# A Comprehensive Study on Tetraaryltetrabenzoporphyrins

**DOI:** 10.1002/chem.201904718

**Published:** 2020-02-06

**Authors:** Michael Ruppel, Dominik Lungerich, Sabrina Sturm, Rainer Lippert, Frank Hampel, Norbert Jux

**Affiliations:** ^1^ Department of Chemistry and Pharmacy & Interdisciplinary Center for, Molecular Materials (ICMM) Organic Chemistry II Friedrich-Alexander University Erlangen–Nuernberg Nikolaus-Fiebiger-Str. 10 91058 Erlangen Germany; ^2^ Department of Chemistry and Pharmacy, Bioinorganic Chemistry Friedrich-Alexander University Erlangen-Nuernberg Egerlandstr. 1 91058 Erlangen Germany

**Keywords:** crystal engineering, electrochemistry, photochemistry, porphyrinoids, UV/Vis spectroscopy

## Abstract

Tetraaryltetrabenzoporphyrins (TATBPs) show, due to their optoelectronic properties, rising potential as dyes in various fields of physical and biomedical sciences. However, unlike in the case of porphyrins, the potential structural diversity of TATBPs has been explored only to little extent, owed mainly to synthetic hurdles. Herein, we prepared a comprehensive library of 30 TATBPs and investigated their fundamental properties. We elucidated structural properties by X‐ray crystallography and found explanations for physical properties such as solubility. Fundamental electronic aspects were studied by optical spectroscopy as well as by electrochemistry and brought in context to the stability of the molecules. Finally, we were able to develop a universal synthetic protocol, utilizing a readily established isoindole synthon, which gives TATBPs in high yields, regardless of the nature of the used arylaldehyde and without meticulous chromatographic purifications steps. This work serves as point of orientation for scientists, that aim to utilize these molecules in materials, nanotechnological, and biomedical applications.

## Graphical abstract

**The underrepresented class** of tetraaryltetrabenzoporphyrins (TATBPs) is investigated thoroughly by DFT calculations, X‐ray diffraction, optical spectroscopy, electro‐ and photochemical studies. Fundamental and unexpected insights were gained about structural and electronic properties of this compound class, which will advance the research around TATPBs based technologies, such as in materials and life sciences.

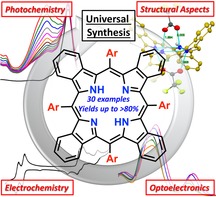

## Introduction

Among the class of nitrogen‐containing macrocycles, porphyrins and phthalocyanines are probably the most exploited representatives, not least because of their biological importance.^1^ With prospective applications in medicinal chemistry^2^, ^3^, ^4^ as well as in materials, including sensors^5^, ^6^ or dye‐sensitized solar cells,^7^, ^8^ literature on porphyrins keeps on growing without any sign of regression. With that said, it is surprising that tetraaryltetrabenzoporphyrins (TATBPs), the artificially prepared benzannulated “bigger brothers” of porphyrins, are found to be rather underrepresented in chemical research. Even though the first synthesis of tetrabenzoporphyrins (TBPs) reaches back to 1938,^9^ their preparation at temperatures above 350 °C limited intrinsically the development of structural variety. Delicate substituents would not “survive” these conditions and the crude reaction mixtures required thorough chromatographic purification. However, within the last three decades, better synthetic routes, mainly based on masked isoindoles, were developed.^10^ Since then, prospective applications in organic near infrared light‐emitting devices,^11^, ^12^, ^13^, ^14^, ^15^, ^16^ in organic photovoltaics,^17^, ^18^, ^19^, ^20^, ^21^, ^22^, ^23^ or as oxygen sensors,^24^, ^25^ have been published. Benefiting from the strong absorption characteristics in the red and NIR region, TATBPs showed good performance in photon up‐conversion (sTTA‐UC) processes,^26^, ^27^, ^28^, ^29^ were investigated as sensitizers for the therapeutic carbon monoxide release^30^ and studied on various metal surfaces,^31^, ^32^, ^33^ showing peculiar self‐assembling behavior, which could find potential applications in organic electronics or catalysis. Despite these promising results, the research around TATBPs is still in its infancy and lacks in structural diversity. To date, the focus was set to a large portion on common derivatives, for example phenyl‐substituted TBPs,^11^, ^14^, ^27^, ^30^, ^34^ which neglects the steric and electronic fine‐tuning properties of the aryl moieties. Subsequently, a “black box” of numerous unexplored properties and applications is left behind.

**Figure 1:**
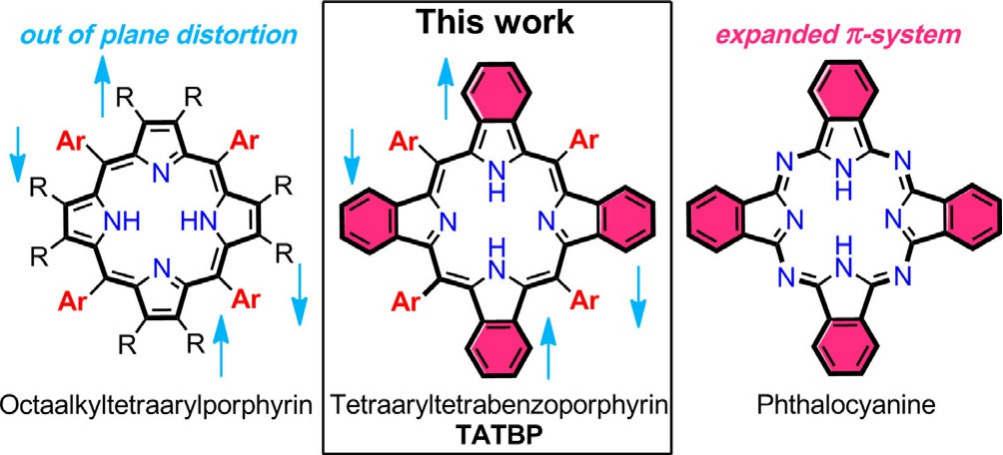
The general structure of TATBP, the molecule class that this work focusses on.

Herein, we bridge the gap of fundamental aspects of TATBPs (Figure 1) by systematically investigating aryl‐substituent effects on the optoelectronics, electrochemistry, photostability as well as the influence on the geometrical constitution in the solid state by means of X‐ray diffraction analysis (XRDA). Furthermore, we improved and simplified the synthetic protocol to such an extent that the application of TATBPs becomes feasible and attractive to fields apart from synthetic organic chemistry, which aims to encourage the exploration of TATBPs in novel applications.^35^

## Results and Discussion

### Design and synthesis

We prepared 30 TATBPs, of which the majority were unknown (see Table 1). During the exploration of suitable isoindole synthons, we found 4,7‐dihydro‐4,7‐ethano‐2*H*‐isoindole **1** (compare reaction Scheme in Table 1), introduced by Jeong et al.,^36^ to be the most facile reagent. Even though condensations of **1**, and derivatives thereof, to TBPs were published before,^37^, ^38^ we discovered that the combination of the reaction details, some of which are reaction time, concentration of the reagents, and the nature of the catalytic acid had a tremendous impact on the reaction outcome. Summarized, we found a condensation time of 18 h, a typical overnight reaction, was necessary to trap the thermodynamically favored tetrapyrrolic structure. Shorter reaction periods (<12 h) resulted in a complicated mixture, containing higher macrocyclic structures and lower yields of the TATBPs. Importantly, this observation was also true for fluorinated benzaldehydes, which are prone for the formation of larger macrocyclic structures (compare yields of structures **5**–**10** in Table 1).^38^ In our protocol, we increased the reagents molarity from 10 mm (Lindsey conditions)^39^ to 17 mm and found that under these conditions, BF_3_⋅OEt_2_ as Lewis acid, served equally well, regardless of the electronic nature of the arylaldehyde. Another crucial detail revealed the work‐up of the obtained bicyclooctadiene (BCOD) porphyrin intermediate. An aqueous work‐up with Na_2_SO_3_ and Na_2_CO_3_ is sufficient, before the BCOD‐porphyrin is applied to the thermal retro‐Diels–Alder transformation to the TATBP. Final purification is obtained by a short silica gel plug filtration, followed by precipitation. To our delight, all isolated yields exceeded reported yields often by a factor of two, giving the target compounds in good to very good yields (compare yields of e.g., **2**, **4**, **10**, **19**, **21** and **26** in Table 1). Metalation of TATBPs can be carried out under standard conditions elaborated for porphyrins. A standard method for Pd^II^ complexation, which was used to prepare **12Pd** can be extracted from the Supporting Information.

**Table 1:** Herein prepared and investigated TATBPs.

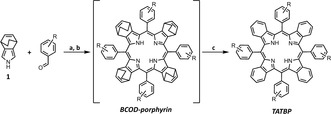				
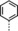	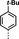	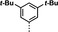	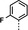	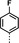
**2** (75 %) 35 %^37^	**3** (58 %)^a^	**4** (40 %) 20 %^12^	**5** (80 %)	**6** (86 %)^a^
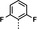	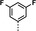	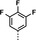	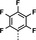	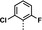
**7** (58 %)	**8** (70 %)^a^	**9** (59 %)^a^	**10** (57 %) 18 %^38^	**11** (52 %)
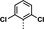	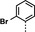	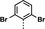	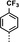	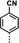
**12** (57 %)	**13** (76 %)	**14** (44 %)	**15** (66 %)^a^	**16** (68 %)
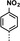	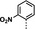	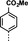	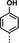	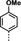
**17** (30 %)^a^	**18** (35 %)	**19** (67 %) 39 %^46^	**20** (50 %)^a^	**21** (51 %) 23 %^47^
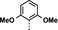	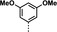	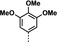	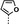	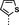
**22** (55 %)	**23** (69 %)	**24** (56 %)	**25** (20 %)	**26** (50 %) 25 %^37^
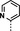	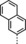	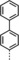	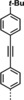	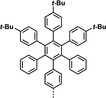
**27** (15 %)	**28** (35 %)	**29** (59 %)^a^	**30** (65 %)^16^	**31** (60 %)^48^

a) CH_2_Cl_2_, arylaldehyde, **1**, cat. BF_3_⋅OEt_2_, N_2_, rt, 18 h; b) DDQ, N_2_, reflux, 2 h; c) vacuum, 205 °C, 1 h; isolated yields for the various arylaldehydes are listed in the table below in brackets; yields reported in literature are displayed without brackets. [a] compound reported in literature, but without sufficient synthetic details and yields.

### Structural analysis

**Figure 2:**
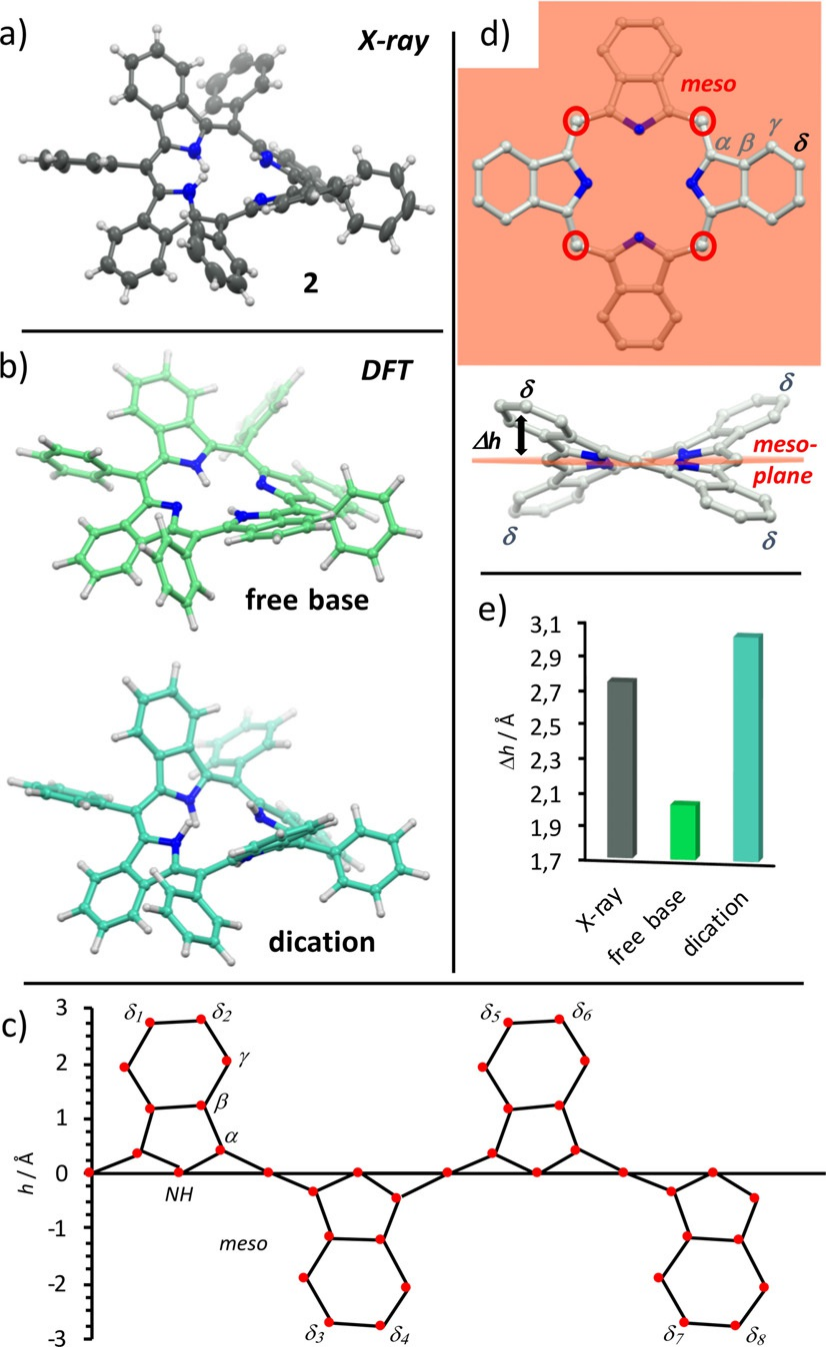
TBP‐skeleton analysis: a) single crystal X‐ray structure of **2**; ellipsoids are drawn at 50 % probability; b) geometry optimized structure of **2** as free‐base and diprotonated dication at the DFT B3LYP 6–311G** level of theory; c) skeletal deviation of **2** from XRDA; d) definition of Δ*h*; e) Δ*h* for **2**, derived from XRDA and DFT.^80^

Constitutionally, TATBPs can be regarded as hybrids between sterically congested and thus distorted porphyrins, and π‐expanded but planar phthalocyanines.^40^ As demonstrated in the single‐crystal X‐ray structure of **2** displayed in Figure 2 a, the saddle‐shape geometry reveals a critical distinction from planar tetraarylporphyrins, which is a result of the steric repulsion between the aryl moieties and the annulated benzo moieties. Similar distortions are known for dodeca substituted porphyrins,^38^, ^39^ such as octaethyltetraphenylporphyrin.^41^, ^42^ Furthermore, the curvature of the saddle‐shaped TBP‐skeleton can be reversibly increased by protonation of the pyrrolic inner nitrogen, as depicted in the density functional theory (DFT) geometry optimized structures of **2** (free base) and H_2_ **2** ^2+^ (dication) in Figure 2 b. This is in sound agreement with the protonation,^43^ or the irreversible N‐methylation of porphyrins.^44^, ^45^ Unfolded, the structure of a TATBP can be well described in a skeletal deviation diagram, as it is depicted for the crystal structure of **2** in Figure 2 c. However, for comparative studies, the flap‐height Δ*h*, which is defined as the distance between the *δ*‐carbon and the plane generated through all *meso*‐carbons (compare Figure 2 d), is more suitable for multiple structures. In Figure 2 e, the Δ*h* values derived from the DFT calculations in the gas‐phase show a deviation of 1.0 Å between the free‐base (Δ*h*=2.02 Å) and dication (Δ*h*=3.02 Å) form. The latter matches well with the value of the obtained crystal structure of **2** (Δ*h*=2.74 Å), which was crystalized as dication. The proton induced flapping is a useful tool to solubilize hardly soluble free‐base TATBPs like **10**, which exhibits otherwise pigment‐like behavior.

**Figure 3:**
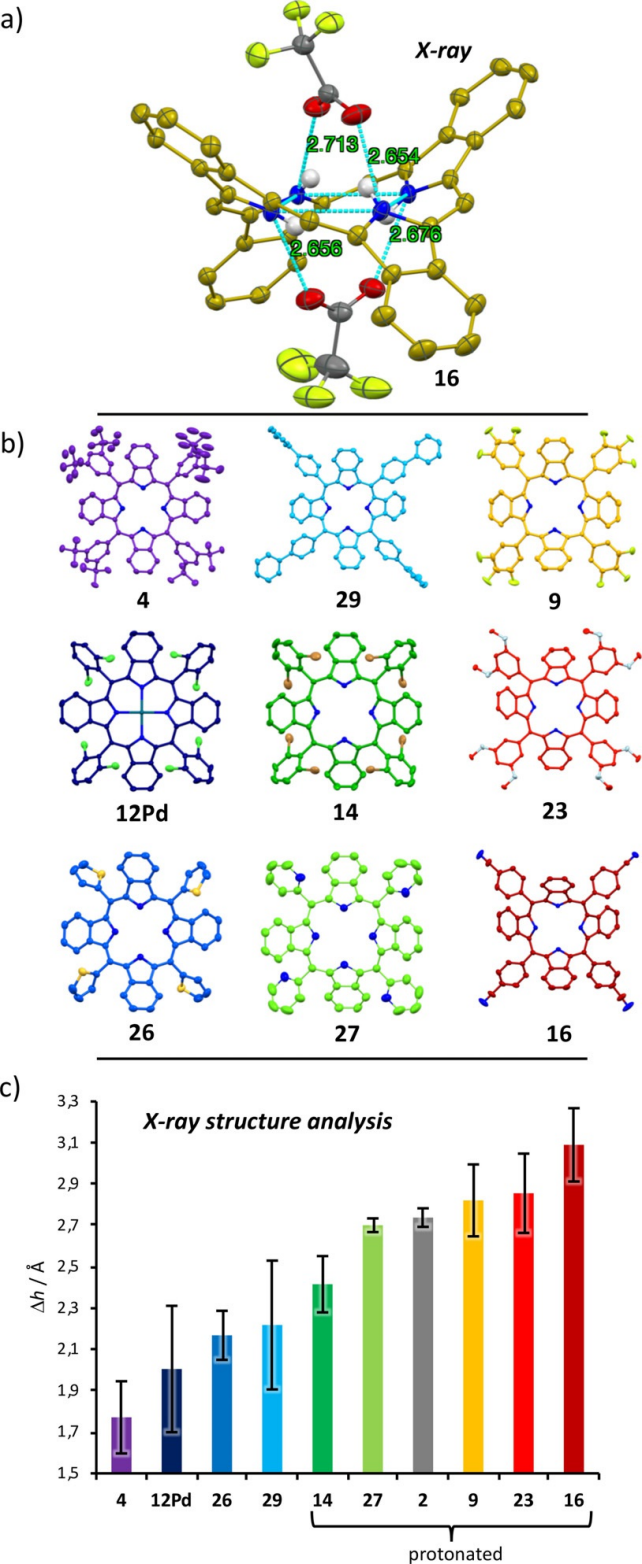
XRDA analysis of saddle‐shape distortion in TATBPs: a) tight‐ion pair binding of TFA molecules with the inner NH; aryl moieties and residual H‐atoms are omitted for clarity; ellipsoids are drawn at 50 % probability; b) XRDA structures of analyzed TATBPs; c) Δ*h* analysis.^80^

In particular, trifluoroacetic acid (TFA) undergoes a tight ion pair binding with the TATBPs (TFA‐O**⋅⋅⋅**NH=2.7 Å), as it is highlighted in Figure 3 a, which even allows the purification on silica gel. Consequently, the negative curvature of the aromatic π‐system has an impact on the properties of the TATBPs. These include solubility, aggregation behavior and morphology. Derived from single crystal X‐ray structures of various TATBPs (**2**, **4**, **9**, **12Pd**, **14**, **16**, **23**, **26**, **27** and **29**), the impact of the aryl moiety on the distortion and its manipulation is depicted in Figure 3 b and 3 c. While the saddle‐shape distortion of TATBPs is expressed with Δ*h* in the bar diagram in Figure 3 c, the other types of distortions are reflected in the respective error bars (compare NSD analysis in Figure 5). Even though the degree of distortion is also determined by crystal packing forces, a trend can be rationalized. The highest degree of saddle‐shape bending was observed by protonation of the inner nitrogen positions, as already described in Figure 2. As it can be derived from XRDA of protonated **27**, **2**, **9**, **23** and **16**, the Δ*h* values are located around 2.9 Å (2.71–3.09 Å), whereas free‐base species (**4**, **26** and **29**) are found around 2.0 Å (1.77–2.22 Å).

The combination of induced severe distortion and the intercalated anion bulk (CF_3_COO^−^) leads to a largely suppressed packing interaction in the solid‐state. Especially in the case of fluorinated TATBPs, which show pigment‐like behavior, the very poor solubility in common solvents can be overcome by protonation with trifluoroacetic acid (TFA). In that sense, the processability of TATBPs can be switched by acid/base treatment. However, despite the protonation with TFA, the *ortho*‐dibrominated derivative **14** shows, due to the steric repulsion of the bromo substituents, only a Δ*h*=2.42 Å, which reflects a decreased solubility compared to its chlorinated or fluorinated analogues. Increased solubility of neutral TATBPs is observed for highly solubilizing substituents such as *m‐t*Bu‐moieties as in case of **4**, or if the symmetry is disturbed due to the presence of rotamers as in the case of **5**, **11**, **13**, **18** and **25**–**28**. Metallated species show bending degrees comparable to free‐base species (compare **12Pd**, Δ*h*=2.01 Å).

**Figure 4:**
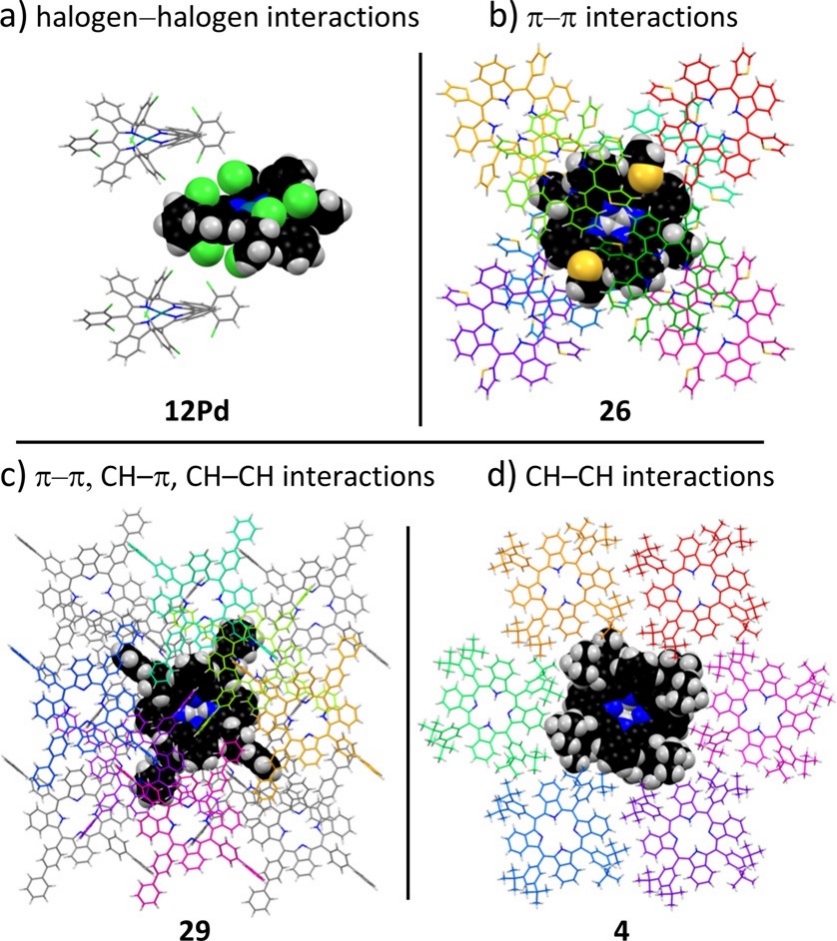
Single crystal packing motifs: a) Cl–Cl interactions in **12Pd**; b) π–π interactions in **26**; c) multiple interaction types in **29**; d) CH–CH interactions in **4**.^80^

On the other hand, with a decreasing Δ*h* value the “comfortable zone” for distinct intermolecular interactions are empirically found to begin with Δ*h*<2.3 Å. In Figure 4, we highlighted four different solid‐state binding motifs, obtained from **4**, **12Pd**, **26** and **29**. All four species show packing interactions between the TATBPs that propagate through the crystal, without being isolated by intercalated solvent molecules. However, the nature of the packing interactions varies strongly with the aryl substituents. In Figure 4 a, *trans*‐type I halogen–halogen interactions^49^ with Cl–Cl distances of 3.35 Å were found for **12Pd**, forming one‐dimensional rods with molecules above and beneath its molecular plane. Thiophenyl substituted TATBP **26** depicted in Figure 4 b, is surrounded by eight neighboring molecules, which undergo π–π interactions of the TBP‐core in distances of 3.21 and 3.74 Å, which facilitates the charge transport in solar cells.^19^ In contrast, biphenyl substituted TBP **29** shows a large variety of CH–CH, CH–π and π–π interactions originated solely at the biphenyl moieties, leading to a tightly interwoven architecture (Figure 4 c). Hereby, one molecule interacts with up to sixteen surrounding molecules. Lastly, the crystal packing of *t*Bu substituted TATBP **4** is displayed in Figure 4 d, which is largely dominated by dispersive CH–CH interactions, originating from the *t*Bu groups. These CH–CH interactions lead to an in‐plane hexagonal surrounding of one molecule. While **4** forms nicely crystals, its solubility remains still very high in CH_2_Cl_2_, which is characteristic for London dispersion interactions.^50^, ^51^, ^52^, ^53^ Even though the TBP‐skeletons show a similar distortion in all four structures, the examples show that *meso*‐substituents have a distinct influence on the crystal‐packing, which allows for manifold manipulation.

**Figure 5:**
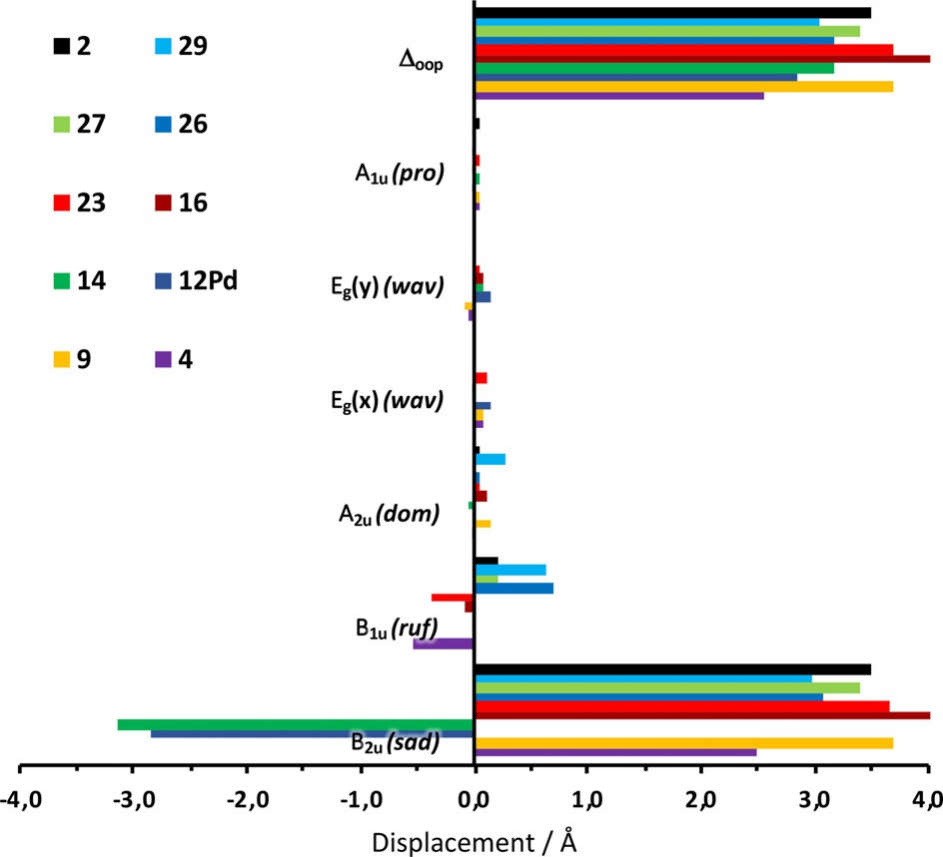
NSD analysis for out of plane distortions of **2**, **4**, **9**, **12Pd**, **14**, **16**, **23**, **26**, **27**, and **29**.^80^

An in‐depth analysis of the skeletal distortion of TATBPs illustrated in Figure 3 b, can be carried out by the normal‐coordinate structural decomposition (NSD) method introduced by Shelnutt and co‐workers,^54^, ^55^, ^56^, ^57^ which breaks down the various normal modes of vibration of the porphyrin macrocycle. As illustrated in Figure 5 for the six most common lowest‐frequency out of plane modes,^58^, ^59^ the overall out of plane distortion (Δ_oop_) goes hand in hand with the earlier provided Δ*h* analysis from Figure 2 and 3. Here the major contribution for distortion arises for all analyzed TATBPs from the B_2u_ mode, which stands for the saddling distortion. The other vibrations, such as ruffling (B_1u_), doming (A_2u_), the degenerate waving modes (E_g_(*x*), E_g_(*y*)), or propelling (A_1u_) play only a minor role in TATBPs. The full NSD analysis, including the in‐plane distortions, is provided in the Supporting Information.

### Optoelectronics

**Figure 6:**
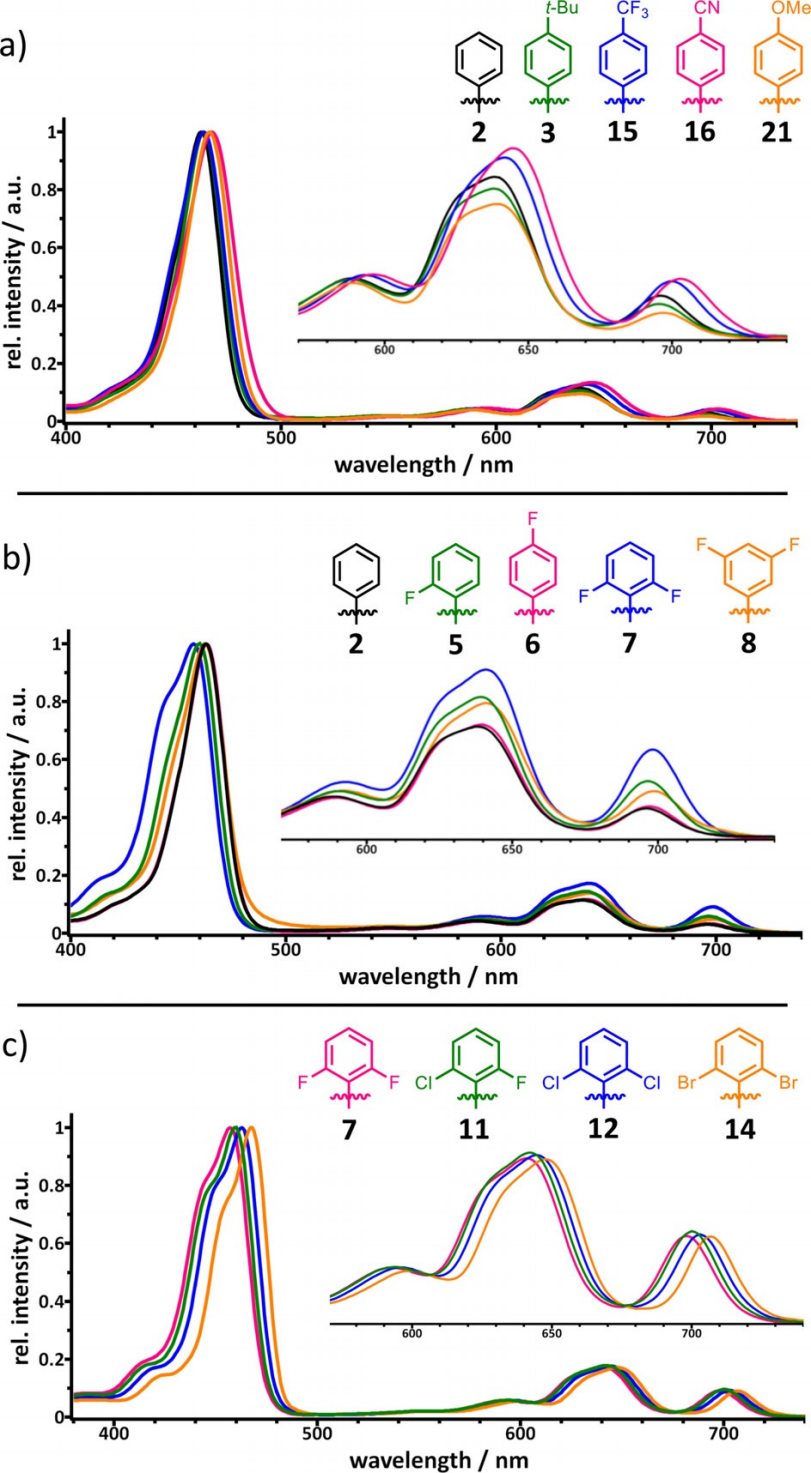
Changes in normalized absorption spectra of different TATBPs correlated to: a) the electronic nature of the substituent; b) the position of the substituent; c) the element.

All TATBPs synthesized in this work show the typical absorption and emission features (see Supporting Information), which have already been described in literature.^60^, ^61^ However, the majority of publications describes the characteristics of metallated species.^12^, ^13^, ^15^, ^24^, ^26^, ^62^, ^63^, ^64^, ^65^ In the following, the influence of the substituents in the aryl group with respect to their positions and electronic nature is highlighted by means of absorption spectroscopy. A table summarizing the relative oscillator strength of all compounds is shown in the Supporting Information (Table S6). First, the electronic nature of the substituents within the aryl moieties and its effect on the absorption features is described. Therefore, the spectra of **3**, **15**, **16** and **21** are compared with respect to **2** (shown in Figure 6 a). The introduction of substituents with inductive effects like **3** (+I effect) or **15** (−I effect) exhibits a slight redshift of the Soret band of about 1 nm, while substituents with mesomeric effects like **16** (−M effect) or **21** (+M effect) cause a bathochromic shift of about 5 nm compared to **2**. Furthermore, it can be stated that electron‐donating groups in *para* position decrease the relative intensities of the Q‐bands accompanied by a slight redshift of the absorption maxima. In contrast, electron‐withdrawing groups cause a bathochromic shift of 4 to 8 nm with a simultaneously increase in the oscillator strength of the Q‐bands.

However, not only the electronic nature but also the position of the substituent has an influence on the absorption properties. Therefore, the absorption spectra of **2** and different fluorinated TATBPs are compared and depicted in Figure 6 b). Due to the short range of the −I effect, no change in the absorption features is observed in the case of **6**, in which the fluorine substituent is located in the *para* position. In contrast, placing a fluorine in *ortho* position (**5**), a hypsochromic shift of 3 nm for the Soret band and a bathochromic shift of 3 nm for the first and second Q band Is observed. In addition, the relative intensities of the Q‐bands are increased by 1 % for the first one and 3 % for the latter two. A second fluorine substituent in *ortho* position **7** leads to a further hypsochromic shift of 3 nm for the Soret band and bathochromatic shifts of 2–3 nm for the Q bands. In the case of **8**, in which the fluorine substituents are situated in *meta* positions, the described effects are not so strongly pronounced as in **7**. Lastly, the “element effect” is highlighted in Figure 6 c for 2,6‐dihalogenated TATBPs **7**, **11**, **12** and **14**. Hereby **7**, which has two fluorine substituents in *ortho* position, exhibits absorption features at 457, 593, 641 and 699 nm. The gradual exchange of fluorine for chlorine atoms (**11**) causes a bathochromic shift to absorption maxima at 460, 593, 642 and 700 nm, and to 463, 596, 645 and 703 nm for dichlorinated species **12**, respectively. The most red‐shifted absorption bands at 468, 600, 647 and 706 nm are observed for 2,6‐dibromophenyl compound **14**. Furthermore, there is no change in oscillator strength of the Q‐bands. A similar trend is also found for the literature reported values of the corresponding 2,6‐dihalogenated TPPs.^66^, ^67^, ^68^ A correlation between the location of the absorption maxima and the electronic nature (electronegativity, steric demand) was not successful, but we believe the observed “element effect” is the result of a non‐linear interference between electronic and steric aspects of the substituents.

### Electrochemistry

The electrochemical characteristics were studied by cyclic voltammetry and differential pulse voltammetry in the potential range from −1.8 to 1.8 V in CH_2_Cl_2_ containing 0.1 m TBAPF_6_. The potentials are summarized in Table 2. All examined TBPs showed two reductions and depending on the aryl substituent one to three oxidations. The potential for oxidation and reduction vary with the nature, the position and the number of substituents within the aryl rings, whereas the highest occupied molecular orbital—lowest unoccupied molecular orbital (HOMO–LUMO) gap is only slightly affected. For some of the TBPs, irreversible signals are observed as either anodic or cathodic currents. These signals are believed to originate from side products that are formed during oxidation/reduction of the TBP.^69^

**Table 2:** Electrochemical characteristics (V vs. Ag/AgCl) for the TBPs in CH_2_Cl_2_ containing 0.1 m TBAPF_6_.

N	*E*_red2_ [V]^[a]^	*E*_red1_ [V]^[a]^	*E*(LUMO) [eV]^[b]^	*E*_ox1_ [V]^[a]^	*E*_ox2_ [V]^[a]^	*E*_ox3_ [V]^[a]^	*E*(HOMO) [eV]^[b]^	*E*_g_^elect^ [eV]	*E*_g_^opt^ [eV]^[c]^
2	−1.40	−1.19	−3.35	0.62	0.75	1.21	−5.16	1.81	1.73
3	−1.47	−1.26	−3.28	0.57	0.65	1.14	−5.11	1.83	1.73
4	−1.55	−1.25	−3.29	0.54	0.72	–	−5.08	1.79	1.73
6	−1.38	−1.21	−3.33	0.65	0.76	1.26	−5.19	1.86	1.73
7	−1.35	−1.05	−3.49	0.73	0.96	–	−5.27	1.78	1.73
8	−1.45	−1.21	−3.33	0.63	0.87	–	−5.17	1.84	1.72
10	−1.12	−0.88	−3.66	0.90	–	–	−5.44	1.78	1.71
16	−1.25	−1.09	−3.45	0.76	–	–	−5.30	1.85	1.71
19	−1.27	−1.14	−3.40	0.70	0.81	1.25	−5.24	1.84	1.72
21	−1.45	−1.23	−3.31	0.58	0.85	1.13	−5.12	1.81	1.74
22	−1.72	−1.44	−3.10	0.40	0.65	–	−4.94	1.84	1.73
23	−1.44	−1.22	−3.32	0.61	0.75	1.18	−5.15	1.83	1.74
24	−1.43	−1.23	−3.31	0.62		1.14	−5.16	1.85	1.74
29	−1.39	−1.21	−3.33	0.61	0.71	1.19	−5.15	1.82	1.72
H_2_TPP^[d]^	−1.54	−1.19	−3.35	1.02	1.37	–	−5.56	2.21	–

[a] Potential values are taken from differential pulse voltammograms. [b] *E*(LUMO)=−(*E*_red_+4.54) eV; *E*(HOMO)=−(*E*_ox_+4.54) eV.^78^ [c] *E*_g_^opt^=1240/*λ*_a.e_.^79^ [d] Reference values.^78^

**Figure 7:**
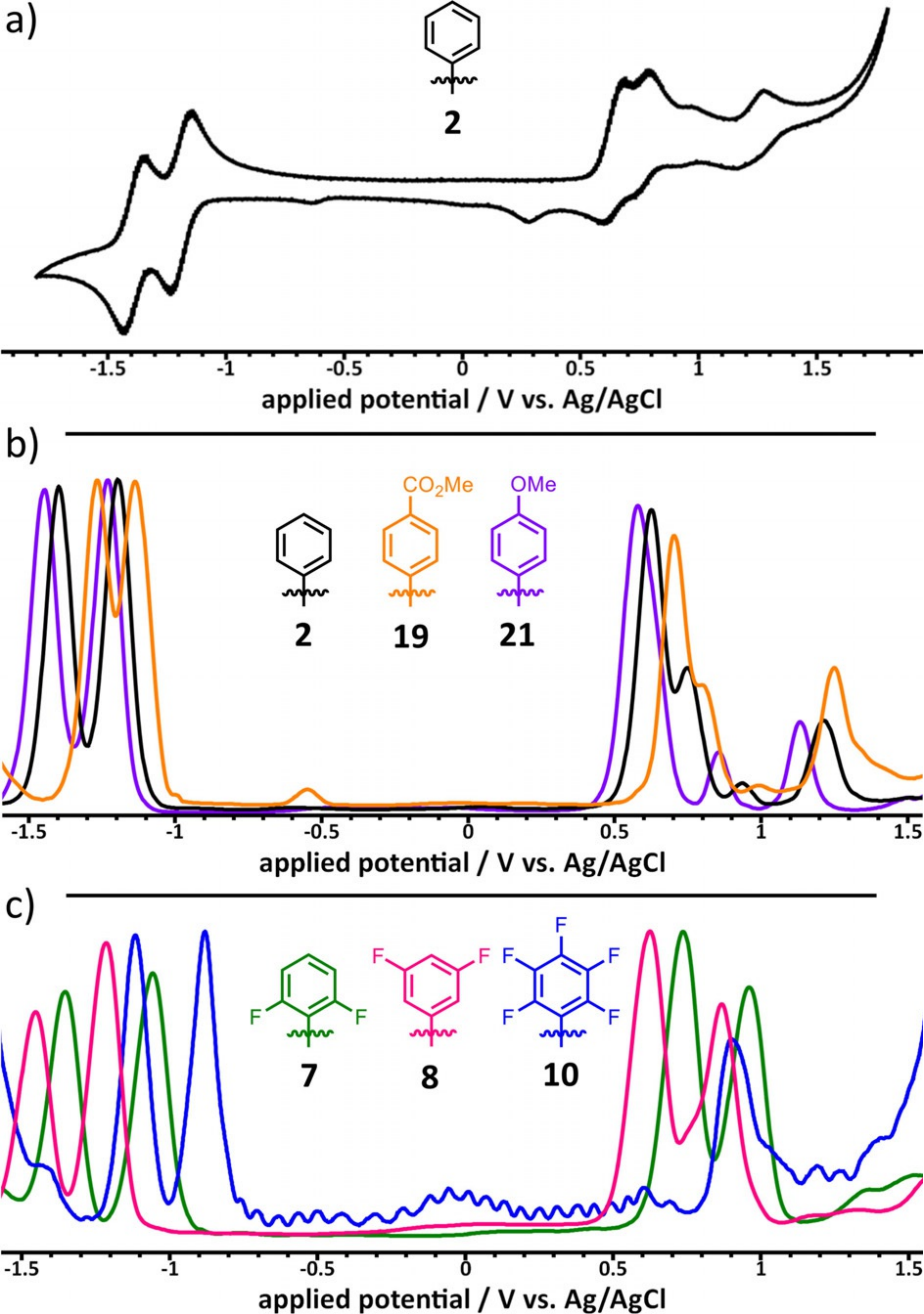
Electrochemical characteristics of different TATBPs, measured in CH_2_Cl_2_, containing 0.1 m TBAPF_6_: a) cyclic voltammogram of **2** at a scan rate of 100 mVs^−1^; b) the influence of the electronic nature: normalized different pulse voltammogram of **2**, **19**, and **21** at a scan rate of 10 mVs^−1^; c) influence of the position and the amount of fluorine atoms within the aryl moiety: normalized different pulse voltammogram of **7**, **8**, and **10** at a scan rate of 10 mVs^−1^.

A comparison of electrochemical properties of **2** with its “smaller brother” **H_2_TPP** shows that the oxidations of **2** occur at much lower potential than for **H_2_TPP** (see Figure 7 a and Table 2). This behavior is consistent with the literature, where the narrowing of the HOMO–LUMO gap is described by destabilization of the porphyrins HOMO upon π‐extension.^64^, ^65^, ^70^ In addition, no shift of the first reduction was observed comparing **2** with **H_2_TPP**. The second reduction, however, shifts about 0.15 V to more positive values. The destabilization energy of the HOMO was estimated from the difference of HOMO energies of **H_2_TPP** and **2** and has a value of 0.4 eV. To demonstrate the influence of the electronic nature of the aryl substituents, the voltammograms of **2**, **19** and **21** are compared in Figure 7 b. Compound **2** exhibits two reversible reductions at −1.19 and −1.40 V and three quasi reversible oxidations at 0.63, 0.75 and 1.21 V. The electron donating methoxy groups in **21** shift the potential for both the reductions (−1.23 and −1.45 V) and the oxidations to more negative values (0.58, 0.85 and 1.13 V). The broad shape of the first oxidation of **21** indicates the superposition with another oxidation. In contrast, the potentials shift to more positive values by introduction of an electron withdrawing substituent as in **19**. Here, two reversible reductions at −1.14 and −1.27 V and three quasi reversible oxidations at 0.7, 0.81 and 1.25 V are observed. The additional reduction at −0.55 V can be attributed to the dication of **19**, that was formed by protonation during the measurement.

Figure 7 c shows the dependence of electrochemical characteristics on the position and the number of substituents within the aryl rings. For **8**, which bears two fluorine substituents in *meta*‐position, two quasi reversible reductions at −1.21 and −1.45 V and two quasi reversible oxidations at 0.63 and 0.87 V are observed. In contrast, situating the two electronegative fluorine substituents in *ortho*‐positions (**7**) leads to a shift of both, the reductions (−1.05 and −1.35 V) and the oxidations (0.73 and 0.96 V), to more positive values. An even larger shift to positive values is observed for the penta‐fluorophenyl TBP **10**, which shows two quasi reversible reductions at −0.88 and −1.12 V and one quasi reversible oxidation at 0.9 V. Due to the electron deficiency of **10**, a second oxidation could not be observed in the potential limit of CH_2_Cl_2_.

### Photochemistry

#### Photostability

**Figure 8:**
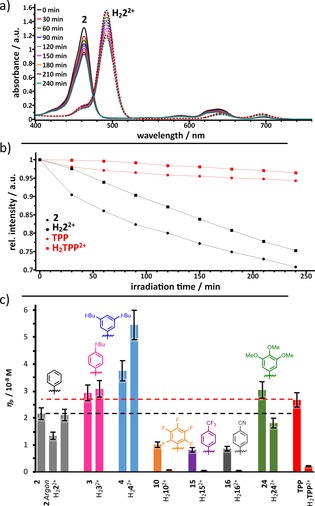
Photobleaching characteristics of TATBPs: a) Photodegradation of free base and diprotonated species of **2** in CH_2_Cl_2_ +1 % NEt_3_ or 1 % TFA, monitored by absorption spectroscopy; b) absorption decrease of the B‐band over irradiation time of **2** (black) and **TPP** (red) under basic (dots) and acidic conditions (squares); c) photobleaching efficiencies *η*_p_ for different TATBPs; 10 % error bars; the photobleaching efficiency is defined as: $\eta_{P}=\;\frac{r_{P}\;\times \;S}{\epsilon_{abs\;}\times \;V}$ , in which V is the reaction volume (3 cm^3^) and S the irradiated area of the cell (0.32 cm^2^) and r_P_ rate of photobleaching after 4 h of irradiation.

In order to evaluate photochemical processes of TATBPs, we studied first the stability upon continuous light irradiation in the region of 620–800 nm, using a cut‐off filter. The photodegradation (bleaching) was determined by following the intensity decrease of the Soret band (B‐band) of the TATBP (compare Figure 8 a), dissolved in CH_2_Cl_2_ upon excitation in the region of the Q‐bands. The photobleaching rate over four hours, exemplified for **2** and **TPP**, is depicted in Figure 8 b. The photobleaching efficiency *η*_p_ of **2**, **3**, **4**, **10**, **15**, **16**, **24** and **TPP**, which considers the integral of the molar extinction coefficients ∫*ϵ* between 620–800 nm of the respective compounds, is shown in Figure 8 c. All data are summarized in the Supporting Information. A comparison of the photobleaching efficiencies of free base TATBPs shows that the photostability is increased for molecules with electron‐withdrawing substituents, which is consistent with earlier reports on porphyrins.^71^, ^72^, ^73^ Interestingly, the nature (inductive or mesomeric effect) of the electron‐withdrawing groups seems to play only a minor role. Both types of substituents lead to an approximately 2.5 times increased photostability compared to reference **2**. In case of electron‐donating substituents, a decrease of photostability by the factor of 1.4 is found for the *para*‐*t*Bu and the methoxy substituted TATBP **3** and **24**, while the 3,5‐di‐*t*Bu TATBP **4** shows, to our surprise, the strongest photobleaching characteristics of all investigated compounds. For all investigated samples except for **3** and **4**, protonation of the inner nitrogen atoms of the macrocycle, leads to an increased photostability. In particular, the protonated species of the electron‐withdrawing substituents (**H_2_10^2+^**, **H_2_15^2+^**, **H_2_16^2+^**), lead to an almost fifteen times higher photostability compared to **H_2_2^2+^**. This observation is in contradiction to previously reported data on porphyrins, which showed that diprotonated species are more prone towards photobleaching.^74^ Through the comparison of an aerated and deaerated (argon‐purged) solution of **2**, we found that photobleaching of TATBPs is an oxidative process. The deaerated sample exhibits a 60 % higher photostability than the aerated solution. Comparing the photobleaching efficiency of **2** with its “smaller brother” **TPP**, compound **2** shows a 1.2‐times higher photostability, while in form of the dication, **H_2_TPP^2+^** exhibits an almost 13‐times higher photostability than **H_2_2^2+^**.

As described in the electrochemistry section (vide supra), π‐expansion leads to a destabilization of the porphyrin HOMO, which results in smaller oxidation potentials (see Table 2). However, our observations are in contradiction with earlier reported theoretical conclusions, which describe an increase in oxidative photobleaching upon π‐expansion.^70^, ^75^ In our case, all investigated TATBPs exhibit a smaller oxidation potential than **TPP**, but only the electron rich compounds **3**, **4** and **24** show higher photobleaching efficiencies. However, under acidic conditions, only the electron poor TATBPs are less prone to photobleaching than **TPP**.

#### Singlet oxygen formation

**Figure 9:**
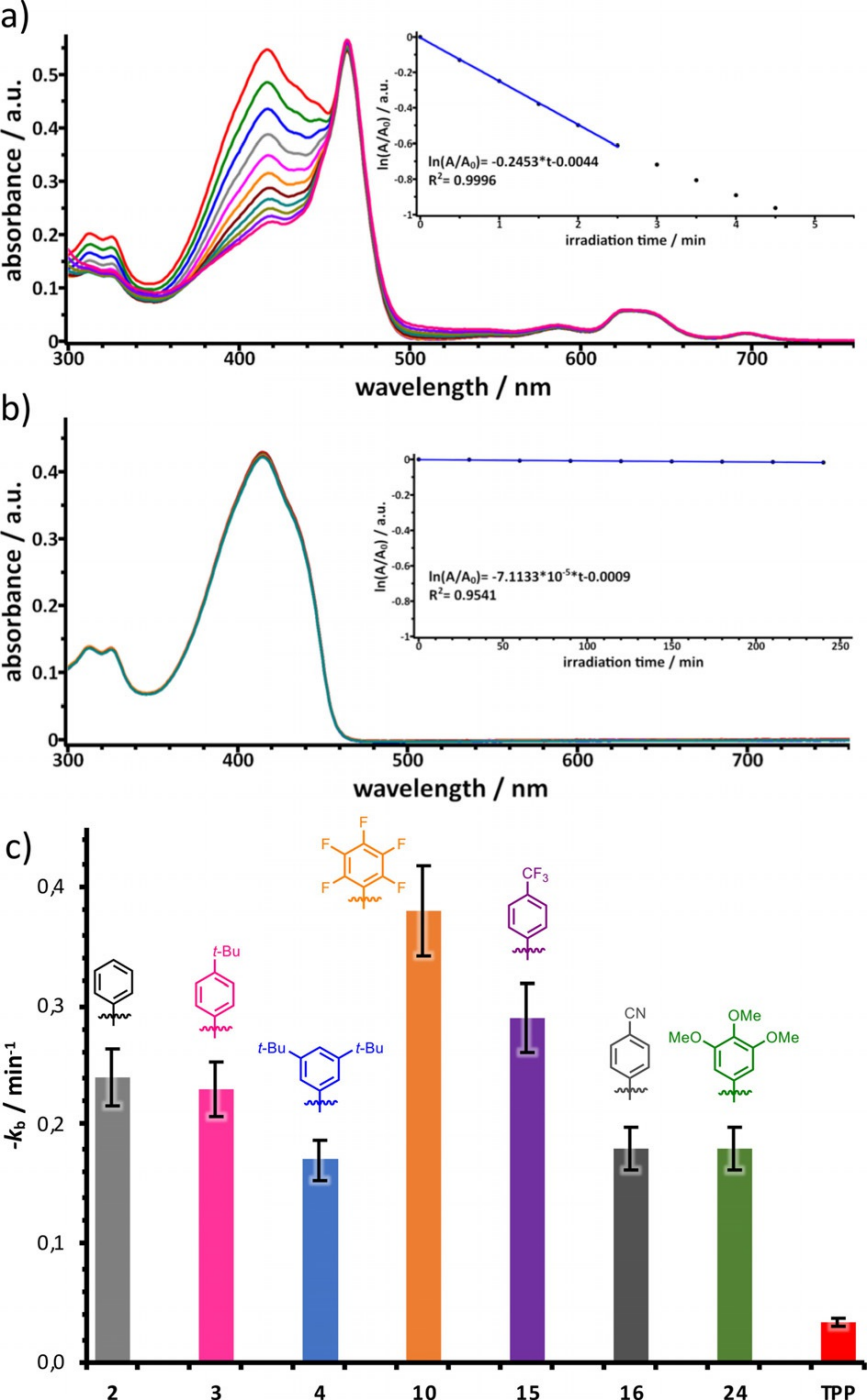
Singlet oxygen generation upon irradiation with red light (620–800 nm) monitored by absorption spectroscopy using **DPBF** as singlet oxygen scavenger: a) decrease in absorption intensity of **DPBF** at 415 nm after different irradiation periods in the presence of **2**; inset: kinetic curve of **DPBF** bleaching versus irradiation time; b) **DPBF** bleaching without any sensitizer; c) the obtained DPBF‐bleaching rate constants *k*_b_ for different TATBPs and **TPP**; 10 % error bars.

The ^1^O_2_ production ability of different TATBPs was determined, using the 1,3‐diphenylisobenzofuran (**DPBF**) photo‐oxidation method.^76^, ^77^ Figure 9 a shows the first‐order kinetics of the **DPBF** oxidation in the presence of **2** upon irradiation in DMF, using the same setup as for the photostability measurements (620–800 nm). The slope of the plot ln(*A*/*A_0_*) against the irradiation time *t*, defines the DPBF‐bleaching rate constant *k*_b_, which is proportional to the amount of produced singlet oxygen ^1^O_2_. The photobleaching of **DPBF** in DMF was found to be negligible for the calculation of *k*_b_, due to a low photobleaching rate of less than 2 % after four hours of irradiation, as depicted in Figure 9 b. All determined values for the investigated TATBPs are summarized in table S8. Overall, the relative ^1^O_2_ quantum yields Δ_rel_(^1^O_2_), are found to be lower for TATBP than for **TPP** (compare Table S8). This observation is consistent with literature, which demonstrated that π‐extension enhances the relaxation through internal conversion.^61^, ^64^ However, in perspective of biomedical relevance, these Δ_rel_(^1^O_2_) values are misleading. Earlier reports showed that π‐extension has a positive effect on the photodynamic activity.^34^, ^61^ As depicted in Figure 9 c, the DPBF‐bleaching rate constant *k*_b_ is significantly larger for the investigated TATBP compared to **TPP**, and thus more ^1^O_2_ is produced in the same time period.^31^ This is due to the higher oscillator strengths of the TATBPs in the red to near‐infrared (NIR) region, in which the samples were irradiated (620–800 nm). Overall, the trend shows that substituents with a −I effect like **10** and **15** have the highest *k*_b_ values, whereas electron‐rich substituents show poorer performance. Surprisingly, **16**, which has an electron‐withdrawing CN‐group with a −M effect performs as poor as **24**, which bears three OMe groups with a strong +M effect. It appears that substituents with −I effects have a beneficial effect on the formation of ^1^O_2_. Categorizing and evaluating the characteristics of the substituents is important for the design of compounds, if biological tissue is targeted for treatment of, for example, cancer. The therapeutic optical window (650–800 nm) for the formation of ^1^O_2_ is limited by the absorption of water and tissue material, which narrows down the utilization of ^1^O_2_‐sensitizers, and makes TATBPs more promising candidates than respective porphyrin derivatives. As a result, a lower dose of the photosensitizer with the same oxidative stress to cancer cells, and a deeper tissue penetration is to be expected for TATBPs.

## Conclusions

Even though the research around tetrapyrroles has accompanied us now for more than a century, several sub‐members of this family, such as the tetraaryltertrabenzoporphyrins (TATBPs), have been investigated only to a little extend. With the preparation of 30 derivatives, we elaborated a comprehensive and systematic study, which describes the fundamental properties of A_4_‐symmetrical TATBPs. For this purpose, we developed a universal and feasible synthetic protocol, which allows us to isolate TATBPs with electron‐rich or poor, with heteroatoms, or sterically demanding substituents, in good to excellent yields. Very often, the obtained amounts outperform by far the typically low yields of macrocyclizations to porphyrins. This optimized methodology, which requires no meticulous column chromatography makes TATBPs truly attractive candidates for the implementation into commercial applications.

Demonstrated by single crystal X‐ray diffraction, unlike planar porphyrins or phthalocyanines, the most significant structural difference of TATBPs represents the inherent saddle‐shape of the core, which can be tuned by the aryl moieties, as well as reversibly switched by the protonation of the inner nitrogen atoms. This negative curvature of the core opens novel design strategies of materials, which utilize the convex–concave intermolecular interactions. Further, we showed that crystal packing motifs are strongly influenced by the available diversity of aryl moieties and allows for a precise crystal‐engineering with various packing motifs.

Absorption and emission spectroscopy revealed rather consistent characteristics, which show moderate changes to the nature of the substituents. However, this propensity is not reflected in the electrochemistry, which shows significant shifts of the potentials with respect to the aryl moieties. Hence, electronic fine‐tuning for the application in solar cells, in order to optimize charge‐extraction properties without changing the broad absorptions over the Vis–NIR region, can be easily envisioned.

Our photochemical explorations resulted in several unexpected findings, which stand in contrast to theoretically predicted properties. For example, the high oxidative photostability of several TATBPs cannot be directly associated with a reduced HOMO–LUMO gap, as the gap remains virtually the same for all investigated species. Lastly, the high singlet oxygen formation ability within the therapeutic optical window, makes these compounds attractive candidates for the utilization in biomedical application, such as in photodynamic therapy.

With our presented library, we aim to advance and to accelerate the research around TATBPs, which represent promising building blocks for novel materials, as well as powerful candidates in nano‐ and biomedical applications.

## Experimental Section

General procedure to tetraaryltetrabenzoporphyrins: A 500 mL Schlenk round bottom flask equipped with a magnetic stir bar and protected from daylight was charged with 4,7‐dihydro‐2*H*‐ethanoisoindole **1** (613 mg, 4.22 mmol), the respective benzaldehyde (4.25 mmol) and dissolved in CH_2_Cl_2_ (250 mL). The mixture was deoxygenated by passing a moderate stream of N_2_ through the solution for 15 min. Then, BF_3_⋅OEt_2_ (150 μL, 173 mg, 1.22 mmol) was added, and the mixture was stirred for 16–18 hours at rt. After the addition of DDQ (950 mg, 4.19 mmol), the mixture was brought to reflux for 2 h. The dark solution was washed with 10 % aqueous Na_2_SO_3_ (250 mL) and 10 % aqueous Na_2_CO_3_ (250 mL), the solvent was removed and the resulting purple residue was heated to 205 °C under reduced pressure for 1 h. *(All reaction steps were carried out in the same reaction flask)*. The purification involved typically a plug filtration (SiO_2_, 3×8 cm) and recrystallization. Details to each purification can be extracted from the supporting information. The products were usually dark green in solution, and dark green or dark blue in the solid state.

## Conflict of interest

The authors declare no conflict of interest.

## Supporting information

As a service to our authors and readers, this journal provides supporting information supplied by the authors. Such materials are peer reviewed and may be re‐organized for online delivery, but are not copy‐edited or typeset. Technical support issues arising from supporting information (other than missing files) should be addressed to the authors.

Supplementary
