# Supplementary material for: A Comprehensive Study on Tetraaryltetrabenzoporphyrins
Source: Chemistry. 2020 Feb 6;26(15):3287–96. doi: 10.1002/chem.201904718 (PMC7154557; doi:10.1002/chem.201904718)
Supplement: Supplementary file 1 — Supplementary [file CHEM-26-3287-s001.pdf]

CHEMISTRY
A European Journal

Supporting Information

A Comprehensive Study on Tetraaryltetrabenzoporphyrins

Michael Ruppel,[a] Dominik Lungerich,*[a] Sabrina Sturm,[b] Rainer Lippert,[b] Frank Hampel,[a] and Norbert Jux*[a]

chem_201904718_sm_miscellaneous_information.pdf

Author Contributions

M.R. Conceptualization: Supporting; Data curation: Equal; Formal analysis: Lead; Investigation: Lead; Methodology: Equal; Visualization: Supporting; Writing - Original Draft: Supporting; Writing - Review & Editing: Supporting
D.L. Conceptualization: Lead; Data curation: Lead; Formal analysis: Supporting; Investigation: Equal; Methodology: Lead; Project administration: Equal; Supervision: Equal; Validation: Equal; Visualization: Equal; Writing - Original Draft: Lead; Writing - Review & Editing: Lead
S.S. Electrochemistry: Supporting
R.L. Electrochemistry: Supporting
F.H. X-ray diffraction: Lead
N.J. Conceptualization: Supporting; Funding acquisition: Lead; Project administration: Equal; Resources: Lead; Supervision: Equal; Validation: Equal; Writing - Review & Editing: Supporting.

Content

1 GENERAL INFORMATION	S2
2 EXPERIMENTAL SECTION	S4
3 X-RAY CRYSTALLOGRAPHIC DATA	S20
4 NSD ANALYSIS	S35
5 ∆H ANALYSIS	S39
6 SPECTRAL APPENDIX (NMR, HRMS, UV/VIS, FLUO)	S40
7 SPECTRAL APPENDIX (CYCLIC VOLTAMMOGRAMS, DPV)	S139
8 PHOTOSTABILITY MEASUREMENTS	S153
9 SINGLET OXYGEN PRODUCTION	S158
10 COMPUTATIONAL DATA	S167
11 REFERENCES	S169

1 GENERAL INFORMATION

All chemicals were purchased from Sigma-Aldrich and used without any further purification. Solvents were distilled prior to usage. Dichloromethane and chloroform were neutralized with K_2CO_3 before distillation. Thin layer chromatography (TLC) was performed on Merck silica gel 60 F524, detected by UV-light (254nm, 366nm). Column chromatography was performed on Macherey-Nagel silica gel 60 M (230-400 mesh, 0.04–0.063 mm). NMR spectroscopy was performed on a Bruker Avance Neo CryoProbe DCH (^{1}H: 600 MHz, ^{13}C: 150 MHz), Bruker Avance 400 (^{1}H: 400 MHz, ^{13}C: 100 MHz), Bruker Avance 300 (^{1}H: 300 MHz, ^{13}C: 75 MHz) or Jeol EX400 (^{1}H: 400 MHz, ^{13}C: 100 MHz). Deuterated solvents were purchased from Sigma Aldrich and used as received. ^{1}H NMR and ^{13}C NMR chemical shifts δ are given in parts per million [ppm] and are referenced to residual protic impurities in the solvent (^{1}H NMR), or to the deuterated solvent itself (^{13}C NMR). The resonance multiplicities are indicated as "s" (singlet), "d" (doublet), "t" (triplet), "q" (quartet) and "m" (multiplet). Signals referred to as "bs" (broad singlet) are not clearly resolved or significantly broadened. LDI/MALDI-ToF (nitrogen UV-laser, 337 nm) mass spectra were obtained by using a Bruker ultrafleXreme spectrometer with 2,5-dihydroxybenzoic acid (DHB) or (*E*)-2-(3-(4-(*tert*-butyl)phenyl)-2-methylallylidene)malononitrile (dctb) as matrices. ESI/APPI-ToF mass spectrometry was carried out on a Bruker maXis 4G UHR TOF MS/MS-spectromter or a Bruker micrOTOF II focus TOF MS-spectrometer. X-ray diffraction analysis was conducted on a Super Nova Dual Wavelength Platform diffractometer from Agilent Technologies GmbH. A suitable crystal was selected and mounted on a mylar loop in perfluoro ether oil on a SuperNova, Dual, Cu at zero, Atlas diffractometer. The crystal was kept at a steady T = 153 K or 173 K during data collection. The structure was solved with the ShelXS 2014/4 solution program using direct methods and by using Olex2 as the graphical interface. The model was refined with ShelXL 2018/3 using full matrix least squares minimization on F^2.[1-5] IR spectroscopy was performed on a Bruker FT-IR Tensor 27 and Pike MIRacle ATR unit. The ATR unit was equipped with a diamond crystal plate and high-pressure clamp. Spectra were recorded as solid samples directly from the diamond crystal. All absorptions $\tilde{\nu}$ are given in wave numbers [cm^{-1}]. UV/vis spectroscopy was carried out on a Varian Cary 5000 UV-Vis-NIR spectrometer. Spectra were recorded at room temperature using quartz cuvettes with a path length of 1 cm. Fluorescence spectra were recorded on a Shimadzu RF–5301PC spectrofluorophotometer. Electrochemical measurements

were conducted in a classical three electrode cell from Deutsche Metrohm GmbH & Co. KG, which was connected to Metrohm Autolab PGSTAT 101, controlled by NOVA 1.6 software, running on a personal computer. As working electrode, a motionless platinum disc electrode (0.07 cm^2) was used combined with a platinum wire that served as counter electrode. All potentials are presented relative to an Ag/AgCl (2 M lithium chloride in ethanol) reference electrode with a potential of 0.164 V *vs.* SHE at 21 ± 1 °C. Spectra were recorded in methylene chloride (HPLC grade) at 21 ± 1 °C with 0.1 M n-Bu_4NPF_6 as supporting electrolyte. For cyclic voltammetry three different scan rates of v = 50, 100 and 500 mVs^{-1} were chosen whereas differential pulse voltammetry was conducted with a scan rate of v = 1 mVs^{-1}. Methylene chloride was degassed with nitrogen (1 min/mL) prior to each measurement. The nitrogen atmosphere was maintained during all measurements. Photostability and singlet oxygen production experiments were carried out in a Hellma® fluorescence standard cell of 1 cm pathlength and equipped with PTFE stopper. For photostability measurements, Ar_4TBP solution (3ml, 3 μM in CH_2Cl_2 + 1% TFA or 1% NEt_3) was introduced to the cell and irradiated in the Q-band region with a cold light source KL1500 LCD, containing a cool beam halogen reflector lamp (150 W, 3050 K) equipped with a 620 nm glass cut-off filter (Schott). Photobleaching experiments were monitored by the absorption decay of the Soret band after certain irradiation periods. For the evaluation of the singlet oxygen production, 1,3-diphenylisobenzofuran (DPBF) was used as scavenger for singlet oxygen. DPBF/Ar_4TBP solution (3 ml, DPBF 20 μM and Ar_4TBP 2 μM in DMF + 1% NEt_3) was added to the cell and irradiated as mentioned above for photostability measurements. Singlet oxygen production was followed by the DPBF absorption decay at 415 nm after certain irradiation periods. The reaction volume for photostability and singlet oxygen production was V= 3 cm^3 and the irradiated area of the cell S= 0.3 cm^2.

2 EXPERIMENTAL SECTION

4,7-dihydro-2*H*-ethanoisoindole **1** was synthesized according to a modified literature procedure. The spectroscopic data are consistent with the literature values.[6]

4-Bromo-1,1-diethoxybut-2-yne S1

A mixture of triethyl orthoformate (100 mL, 89.2 g, 600 mmol), propargyl bromide (80% in toluene, 94.0 mL, 100 g, 840 mmol) and ZnI_2 (4.80 g, 15.0 mmol) was heated to 110 °C, and ethanol was removed by slow distillation over 18 h. Then the mixture was combined with brine (100 mL) and extracted with CH_2Cl_2 (4x50 mL). The organic layer was dried over $MgSO_4$ and the solvent was removed under reduced pressure. The crude product was purified by distillation to obtain a pale-yellow oil (42.5 g, 192 mmol, 32%). **^{1}H NMR** (400 MHz, $CDCl_3$, rt) δ [ppm] 5.27 (t, $^5J_{HH}$= 1.5 Hz, 1H), 3.91 (d, $^5J_{HH}$= 1.5 Hz, 2H), 3.74–3.52 (dq, $^2J_{HH}$= 9.4 Hz, $^3J_{HH}$= 7.1 Hz, 4H), 1.21 (t, $^3J_{HH}$= 7.1 Hz, 6H). **^{13}C NMR** (100 MHz, $CDCl_3$, rt) δ [ppm] 91.1, 81.9, 80.3, 61.0, 15.0, 13.4.

3-(Bromomethyl)bicyclo[2.2.2]octa-2,5-diene-2-carbaldehyde S2

A solution of 4-bromo-1,1-diethoxybut-2-yne **S1** (53.6 g, 242 mmol) in formic acid (58.0 mL) was stirred under nitrogen atmosphere for 3 h at 45 °C. Then CH_2Cl_2 (200 mL) and 1,3-cyclohexadiene (33.0 mL, 27.7 g, 346 mmol) were added and the resulting mixture was stirred for 68 h at 45 °C. The mixture was combined with brine (100 mL) and extracted with CH_2Cl_2 (4x50 mL). The combined organic layers were washed with saturated aqueous $NaHCO_3$ solution (2x100 mL) and water (2x100 mL), and dried over $MgSO_4$. The solvent was removed under reduced pressure and the crude product was purified by silica gel plug filtration (CH_2Cl_2, Ø= 12 cm, h= 7 cm) to obtain a yellow, highly viscous oil (45.6 g, 201 mmol, 83%). **^{1}H NMR** (400 MHz, $CDCl_3$, rt) δ [ppm] 9.86 (s, 1H), 6.36–6.31 (m, 2H), 4.45 (m, 2H), 4.27–4.24 (m, 1H), 3.76–3.73 (m, 1H), 1.50–1.26 (m, 4H). **^{13}C NMR** (100 MHz, $CDCl_3$, rt) δ [ppm] 184.3, 158.9, 142.1, 134.6, 132.8, 43.4, 34.2, 26.2, 25.2, 25.1.

4,7-Dihydro-2*H*-ethanoisoindole 1

To a solution of 3-(bromomethyl)bicyclo[2.2.2]octa-2,5-diene-2-carbaldehyde **S2** (45.3 g, 199 mmol) in 1,2-dimethoxyethane (170 mL) was added a 28-30% aqueous NH_4OH solution (90 mL). The mixture was stirred for 3 h at room temperature with exclusion of light. Then the mixture was combined with brine (250 mL) and extracted with CH_2Cl_2 (4x150 mL). The combined organic layers were dried over $MgSO_4$ and the solvent was removed under reduced pressure. The crude product was purified by column chromatography (silica gel, CH_2Cl_2, Ø = 15 cm, h = 20 cm, exclusion of light) and subsequent sublimation (80°C at 0.21 mbar) to obtain a colorless, spicy-smelling, crystalline solid (14.2 g, 97.5 mmol, 49%). **^{1}H NMR** (400 MHz, $CDCl_3$, rt) δ [ppm] 7.49 (br, 1H), 6.53 (dd, $^3J_{HH}$= 4.4 Hz, $^4J_{HH}$= 2.8 Hz, 2H), 6.46 (d, $^3J_{HH}$= 2.0 Hz, 2H), 3.89–3.86 (m, 2H), 1.60–1.50 (m, 4H). **^{13}C NMR** (100 MHz, $CDCl_3$, rt) δ [ppm] 136.3, 129.3, 108.0, 33.1, 27.6.

General procedure for the synthesis of TATBPs:

4,7-Dihydro-2*H*-ethanoisoindole **1** (613 mg, 4.22 mmol) and the corresponding benzaldehyde (4.25 mmol) were dissolved in CH_2Cl_2 (250 mL), and degassed by passing nitrogen through the solution for 15 min. Then BF_3 diehtyl etherate (150 µL, 173 mg, 1.22 mmol) was added, and the mixture was stirred under light exclusion for 16 h at room temperature. After the addition of DDQ (950 mg, 4.19 mmol), the mixture was heated at reflux for 2 h. The solution was washed with 10% aqueous Na_2SO_3 (250 mL) and 10% aqueous Na_2CO_3 (250 mL), the solvent was removed and the resulting purple residue was heated to 205 °C under reduced pressure for 1 h. The purification differs depending on the functional groups of the benzaldehyde-derivatives and is described in the following.

***meso*-Tetraphenyltetrabenzoporphyrin 2**

Purified by filtration over silica gel (CH_2Cl_2) and subsequent recrystallization from CH_2Cl_2/NEt_3 with MeOH, 645 mg, 75%. **^{1}H NMR** (400 MHz, CD_2Cl_2/TFA-d_1, rt) δ [ppm] 8.56–8.54 (m, 8H), 8.07–8.02 (m, 4H), 7.99–7.95 (m, 8H), 7.48 (ps, 16H). **^{13}C NMR** (100 MHz, CD_2Cl_2/TFA-d_1, rt) δ [ppm] 141.3, 141.1, 139.1, 136.1, 131.59, 131.55, 131.3, 130.20, 130.17, 124.7, 115.34, 115.28. **HRMS** (APPI, toluene) m/z calcd for

$C_{60}H_{39}N_4$ $[M+H]^+$ 815.3169, found 815.3184. **UV/Vis** (CH_2Cl_2 + 1% NEt_3) λ [nm] (ε [$M^{-1}cm^{-1}$]) 418 (28100), 463 (310000), 588 (12900), 624 (29700), 637 (34800), 696 (9400). **Fluorescence** (CH_2Cl_2 + 1% NEt_3, λ_{exc} = 463 nm) λ [nm] 654, 702, 779. **IR** (ATR) $\tilde{\nu}$ [cm^{-1}] 3328, 3080, 3052, 2617, 2574, 2361, 1948, 1912, 1894, 1597, 1506, 1439, 1359, 1317, 1174, 1121, 1026, 825, 764, 736, 721, 697, 624.

***meso*-Tetrakis(4-*tert*-butylphenyl)tetrabenzoporphyrin 3**

Purified by filtration over silica gel (CH_2Cl_2) and subsequent recrystallization from CH_2Cl_2/NEt_3 with MeOH, 636 mg, 58%. **^{1}H NMR** (400 MHz, $CDCl_3$/TFA-d_1, rt) δ [ppm] 8.37 (d, $^3J_{HH}$= 8.3 Hz, 8H), 7.93 (d, $^3J_{HH}$= 8.3 Hz, 8H), 7.41 (s, 16H), 1.63 (s, 36H). **^{13}C NMR** (100 MHz, $CDCl_3$/TFA-d_1, rt) δ [ppm] 155.0, 140.9, 135.9, 135.3, 131.0, 129.9, 126.7, 124.1, 114.7, 35.3, 31.5. **HRMS** (ESI, CH_2Cl_2/MeCN) m/z calcd for $C_{76}H_{71}N_4$ $[M+H]^+$ 1039.5673, found 1039.5688. **UV/Vis** (CH_2Cl_2 + 1% NEt_3) λ [nm] (ε [$M^{-1}cm^{-1}$]) 418 (24400), 464 (309000), 588 (12800), 625 (28100), 637 (32300), 696 (7600). **Fluorescence** (CH_2Cl_2 + 1% NEt_3, λ_{exc} = 464 nm) λ [nm] 654, 701, 779. **IR** (ATR) $\tilde{\nu}$ [cm^{-1}] 3324, 3109, 3055, 2963, 2603, 2563, 1954, 1920, 1511, 1447, 1396, 1359, 1339, 1316, 1291, 1267, 1188, 1123, 1022, 945, 880, 865, 850, 804, 769, 760, 742, 720, 638.

***meso*-Tetrakis(3,5-di-*tert*-butylphenyl)tetrabenzoporphyrins 4**

Purified by filtration over silica gel (CH_2Cl_2) and subsequent recrystallization from CH_2Cl_2/NEt_3 with pentane, 533 mg, 40%. **^{1}H NMR** (400 MHz, $CDCl_3$, rt) δ [ppm] 8.25 (d, $^4J_{HH}$= 1.8 Hz, 8H), 7.93 (t, $^4J_{HH}$= 1.8 Hz, 4H), 7.19 (br, 16H), 1.49 (s, 72H). **^{13}C NMR** (100 MHz, $CDCl_3$, rt) δ [ppm] 151.3, 141.0, 129.5, 125.4, 124.4, 121.6, 116.7, 35.3, 31.7. **HRMS** (APPI, toluene) m/z calcd for $C_{92}H_{102}N_4$ $[M]^+$ 1262.8099, found 1262.8105. **UV/Vis** (CH_2Cl_2 + 1% NEt_3) λ [nm] (ε [$M^{-1}cm^{-1}$]) 418 (29000), 462 (317000), 587 (13700), 624 (31900), 634 (36200), 691 (8600). **Fluorescence** (CH_2Cl_2 + 1% NEt_3, λ_{exc} = 462 nm) λ [nm] 654, 699, 777. **IR** (ATR) $\tilde{\nu}$ [cm^{-1}] 3321, 3065, 3047, 2957, 2903, 2866, 2361, 1593, 1476, 1448, 1393, 1362, 1317, 1247, 1223, 1122, 1023, 995, 900, 883, 837, 815, 762, 732.

***meso*-Tetrakis(2-fluorophenyl)tetrabenzoporphyrins 5**

Purified by filtration over silica gel (CH_2Cl_2 + 1% TFA) and subsequent recrystallization from CH_2Cl_2/NEt_3 with MeOH, 749 mg, 80%. **^{1}H NMR** (400 MHz, CD_2Cl_2/TFA-d$_1$, rt) δ [ppm] 8.59–8.45 (m, 4H), 8.10–8.04 (m, 4H), 7.82–7.76 (m, 4H), 7.72–7.66 (m, 4H), 7.62–7.55 (m, 16H). **^{13}C NMR** (100 MHz, CD_2Cl_2/TFA-d$_1$, rt) δ [ppm] 163.82, 163.75, 163.7, 163.64, 163.60, 161.32, 161.25, 161.2, 161.14, 161.10, 142.5, 142.2, 142.0, 141.8, 141.6, 141.4, 141.2, 141.1, 141.0, 140.8, 140.2, 137.5, 137.45, 137.39, 137.3, 137.2, 137.0, 134.6, 134.5, 134.4, 131.7, 131.5, 131.4, 131.3, 131.2, 131.14, 131.06, 131.0, 130.9, 130.8, 126.55, 126.51, 126.4, 124.93, 124.87, 124.8, 124.7, 124.6, 123.8, 123.68, 123.65, 123.60, 123.57, 123.55, 123.49, 123.45, 117.93, 117.89, 117.8, 117.71, 117.68, 117.65, 117.6, 117.5, 108.4, 107.61, 107.56, 107.5, 106.61, 106.55. **^{19}F NMR** (282 MHz, CD_2Cl_2/TFA-d$_1$, rt) δ [ppm] -(111.5–112.2). The NMR data refer to a mixture of 4 atropisomers. **HRMS** (ESI, MeCN/toluene) m/z calcd for $C_{60}H_{35}F_4N_4$ $[M+H]^+$ 887.2792, found 887.2776. **UV/vis** (CH_2Cl_2 + 1% NEt_3) λ [nm] (ε [$M^{-1}cm^{-1}$]) 416 (35800), 448 (191000), 460 (277000), 591 (13200), 625 (32600), 639 (39000), 696 (15800). **Fluorescence** (CH_2Cl_2 + 1% NEt_3, λ_{exc} = 460 nm) λ [nm] 654, 702, 779. **IR** (ATR) $\tilde{\nu}$ [cm^{-1}] 3078, 3001, 2859, 2762, 1777, 1689, 1663, 1610, 1487, 1442, 1400, 1333, 1307, 1191, 1139, 1054, 1029, 943, 863, 835, 807, 756, 725, 697, 620.

***meso*-Tetrakis(4-fluorophenyl)tetrabenzoporphyrins 6**

Purified by filtration over silica gel (CH_2Cl_2 + 1% TFA) and subsequent recrystallization from CH_2Cl_2/NEt_3 with MeOH, 805 mg, 86%. **^{1}H NMR** (600 MHz, $CDCl_3$/TFA-d$_1$, rt) δ [ppm] 8.50–8.48 (m, 8H), 7.66–7.63 (m, 8H), 7.52–7.49 (AA'BB', 8H), 7.48–7.46 (AA'BB', 8H). **^{13}C NMR** (150 MHz, $CDCl_3$/TFA-d$_1$, rt) δ [ppm] 164.9 (d, $^1J_{C\text{-}F}$ = 254.1 Hz), 141.2, 141.0, 137.6 (d, $^3J_{C\text{-}F}$ = 8.2 Hz), 134.8 (d, $^4J_{C\text{-}F}$ = 2.9 Hz), 131.0, 130.4, 124.2, 117.5 (d, $^2J_{C\text{-}F}$ = 21.7 Hz), 113.8, 113.7. **^{19}F NMR** (470 MHz, $CDCl_3$/TFA-d$_1$, rt) δ [ppm] -108.7–(-108.8) (m). **HRMS** (APPI, MeCN/toluene) m/z calcd for $C_{60}H_{34}F_4N_4$ $[M]^+$ 886.2714, found 886.2703. **UV/Vis** (CH_2Cl_2 + 1% NEt_3) λ [nm] (ε [$M^{-1}cm^{-1}$]) 421 (28300), 463 (282000), 589 (11500), 626 (28100), 638 (32200), 697 (9100). **Fluorescence** (CH_2Cl_2 + 1% NEt_3, λ_{exc} = 463 nm) λ [nm] 652, 703, 781. **IR** (ATR) $\tilde{\nu}$ [cm^{-1}] 3111, 3080, 3018, 3000, 2858, 2756, 1904, 1766, 1598, 1509, 1470, 1444, 1334, 1157, 1056, 1014, 810, 761, 724, 698, 623, 606.

***meso*-Tetrakis(2,6-difluorophenyl)tetrabenzoporphyrins 7**

Purified by filtration over silica gel (CH_2Cl_2 + 1% TFA) and subsequent recrystallization from CH_2Cl_2/NEt_3 with MeOH, 588 mg, 58%. **^{1}H NMR** (600 MHz, $CDCl_3$/TFA-d_1, rt) δ [ppm] 8.01–7.97 (m, 8H), 7.71–7.69 (AA'BB', 8H), 7.61–7.60 (AA'BB', 8H), 7.51–7.49 (m, 8H). **^{13}C NMR** (150 MHz, $CDCl_3$/TFA-d_1, rt) δ [ppm] 162.0 (dd, $^1J_{CF}$= 254.4 Hz, $^3J_{CF}$= 4.6 Hz), 140.9, 134.5 (t, $^3J_{CF}$= 10.1 Hz), 131.2, 130.6, 123.1, 115.7 (t, $^2J_{CF}$= 17.5 Hz), 113.2 (overlap with TFA-signal), 99.5. **^{19}F NMR** (470 MHz, $CDCl_3$/TFA-d_1) δ [ppm] -110.7 (s). **HRMS** (APPI, THF) m/z calcd for $C_{60}H_{31}F_8N_4$ $[M+H]^+$ 959.2415, found 959.2412. **UV/Vis** (CH_2Cl_2 + 1% NEt_3) λ [nm] (ε [$M^{-1}cm^{-1}$]) 414 (50000), 446 (220000), 457 (265000), 593 (15700), 627 (38500), 641 (45200), 699 (23800). **Fluorescence** (CH_2Cl_2 + 1% NEt_3, λ_{exc} = 457 nm) λ [nm] 656, 705, 781. **IR** (ATR) $\tilde{\nu}$ [cm^{-1}] 3344, 3320, 3098, 3070, 2958, 2928, 2860, 1725, 1621, 1586, 1561, 1464, 1365, 1318, 1276, 1234, 1122, 1032, 1001, 828, 784, 762, 717.

***meso*-Tetrakis(3,5-difluorophenyl)tetrabenzoporphyrins 8**

Purified by filtration over silica gel (CH_2Cl_2 + 1% TFA) and subsequent recrystallization from CH_2Cl_2/NEt_3 with MeOH, 708 mg, 70%. **^{1}H NMR** (600 MHz, $CDCl_3$/TFA-d_1, rt) δ [ppm] 8.09–8.08 (m, 8H), 7.60–7.58 (AA'BB', 8H), 7.56–7.54 (AA'BB', 8H), 7.50 (tt, $^3J_{HF}$= 8.4 Hz, $^4J_{HH}$= 2.2 Hz, 4H). **^{13}C NMR** (150 MHz, $CDCl_3$/TFA-d_1, rt) δ [ppm] 163.7 (dd, $^1J_{CF}$= 253.5 Hz, $^3J_{CF}$= 12.6 Hz), 140.59, 140.57, 140.5–140.4 (m), 130.9, 130.71, 130.67, 124.4, 118.9–118.7 (m), 112.9, 112.8, 106.9 (t, $^2J_{CF}$= 24.9 Hz). **^{19}F NMR** (470 MHz, $CDCl_3$/TFA-d_1) δ [ppm] -106.7–(-106.7) (m). **HRMS** (APPI, toluene) m/z calcd for $C_{60}H_{30}F_8N_4$ $[M]^+$ 958.2337, found 958.2325. **UV/Vis** (CH_2Cl_2 + 1% NEt_3) λ [nm] (ε [$M^{-1}cm^{-1}$]) 418 (24600), 463 (180000), 593 (8900), 626 (19800), 641 (24600), 698 (8600). **Fluorescence** (CH_2Cl_2 + 1% NEt_3, λ_{exc} = 463 nm) λ [nm] 657, 706, 783. **IR** (ATR) $\tilde{\nu}$ [cm^{-1}] 3337, 3339, 3082, 3050, 1613, 1586, 1446, 1426, 1369, 1307, 1116, 1011, 985, 875, 829, 811, 774, 750, 704, 680.

***meso*-Tetrakis(3,4,5-trifluorophenyl)tetrabenzoporphyrin 9**

Purified by filtration over silica gel (CH_2Cl_2 + 1% TFA) and subsequent recrystallization from CH_2Cl_2/NEt_3 with MeOH, 642 mg, 59%. **^{1}H NMR** (600 MHz, $CDCl_3$/TFA-d_1, rt) δ

[ppm] 8.19 (t, $^{3}J_{HF}$= 6.2 Hz, 8 H), 7.65–7.63 (AA'BB', 8H), 7.55–7.53 (AA'BB', 8H). **^{13}C NMR** (150 MHz, $CDCl_3$/TFA-d_1, rt) δ [ppm] 153.1–151.3 (m), 142.5 (dt, $^{1}J_{CF}$= 260.6 Hz, $^{2}J_{CF}$= 14.8 Hz), 140.9, 140.7, 133.5–133.3 (m), 131.2, 130.64, 130.61, 124.3, 120.0 (dd, $^{2}J_{CF}$= 16.8 Hz, $^{3}J_{CF}$= 4.4 Hz), 112.4, 112.3. **^{19}F NMR** (470 MHz, $CDCl_3$/TFA-d_1, rt) δ [ppm] -130.7 (dd, $^{2}J_{FF}$= 20.0 Hz, $^{3}J_{HF}$= 5.8 Hz), -155.1–(-155.2) (m). **HRMS** (APPI, toluene) m/z calcd for $C_{60}H_{26}F_{12}N_4$ $[M]^+$ 1030.1960, found 1030.1951 **UV/Vis** (CH_2Cl_2 + 1% NEt_3) λ [nm] (ε [$M^{-1}cm^{-1}$]) 419 (28000), 463 (209000), 593 (10500), 626 (23200), 642 (29400), 699 (11400). **Fluorescence** (CH_2Cl_2 + 1% NEt_3, λ_{exc} = 463 nm) λ [nm] 658, 707, 786. **IR** (ATR) $\tilde{\nu}$ [cm^{-1}] 3084, 3002, 2918, 2856, 1776, 1616, 1527, 1430, 1393, 1335, 1208, 1152, 1049, 1009, 826, 822, 762, 701, 638.

meso-Tetrakis(2,3,4,5,6-pentafluorophenyl)tetrabenzoporphyrin 10

Purified by filtration over silica gel (CH_2Cl_2 + 1% TFA) and subsequent recrystallization from CH_2Cl_2/NEt_3 with MeOH, 706 mg, 57%. **^{1}H NMR** (600 MHz, CD_2Cl_2/TFA-d_1, rt) δ [ppm] 7.82–7.79 (m, 16H). **^{13}C NMR** (150 MHz, CD_2Cl_2/TFA-d_1, rt) δ [ppm] 147.1 (m), 145.0 (m), 141.8, 141.6, 139.6 (m), 132.7, 130.6, 123.6, 112.9 (m), 97.6. **^{19}F NMR** (470 MHz, CD_2Cl_2/TFA-d_1, rt) δ [ppm] -138.1–(-131.1) (m), -145.7–(-145.7) (m), -157.6–(-157.7) (m). **HRMS** (APPI, toluene) m/z calcd for $C_{60}H_{18}F_{20}N_4$ $[M]^+$ 1174.1207, found 1174.1218. **UV/Vis** (CH_2Cl_2 + 1% NEt_3) λ [nm] (ε [$M^{-1}cm^{-1}$]) 414 (57300), 447 (224000), 458 (254000), 598 (15900), 632 (44900), 647 (53600), 704 (35500). **Fluorescence** (CH_2Cl_2 + 1% NEt_3, λ_{exc} = 458 nm) λ [nm] 661, 711, 787. **IR** (ATR) $\tilde{\nu}$ [cm^{-1}] 3363, 3334, 3081, 2969, 1648, 1520, 1491, 1445, 1358, 1320, 1166, 1082, 1006, 987, 859, 834, 797, 763, 705, 684.

meso-Tetrakis(2-chloro-6-fluorophenyl)tetrabenzoporphyrin 11

Purified by filtration over silica gel (CH_2Cl_2 + 1% TFA) and subsequent recrystallization from CH_2Cl_2/NEt_3 with MeOH, 562 mg, 52%. **^{1}H NMR** (400 MHz, $CDCl_3$/TFA-d_1, rt) δ [ppm] 7.99–7.93 (m, 4H), 7.84–7.82 (m, 4H), 7.61–7.58 (m, 20H). **^{13}C NMR** (150 MHz, CD_2Cl_2/TFA-d_1, rt) δ [ppm] 163.3–161.6 (m), 141.7, 141.5, 141.3, 141.1, 140.8, 140.6, 140.5, 140.1, 139.07, 139.05, 139.0, 138.93, 138.88, 138.86, 134.19, 134.16, 134.13,

134.10, 131.1, 131.03, 130.98, 130.95, 130.8, 130.7, 130.6, 130.5, 127.32, 127.29, 127.27, 125.63, 125.55, 125.5, 123.4, 123.3, 123.23, 123.19, 123.11, 123.09, 123.07, 123.04, 123.00, 116.09, 116.06, 116.02, 115.98, 115.94, 115.92, 115.88, 103.9, 103.5, 103.2. **^{19}F NMR** (470 MHz, CD_2Cl_2/TFA-d_1, rt) δ [ppm] -107.4–(-107.6) (m). The NMR data refer to a mixture of 4 atropisomers. **HRMS** (APPI, toluene) m/z calcd for $C_{60}H_{30}Cl_4F_4N_4$ $[M]^+$ 1022.1155, found 1022.1149. **UV/Vis** (CH_2Cl_2 + 1% NEt_3) λ [nm] (ε [$M^{-1}cm^{-1}$]) 419 (27100), 449 (120000), 460 (150000), 593 (8700), 626 (20400), 642 (26200), 700 (14100). **Fluorescence** (CH_2Cl_2 + 1% NEt_3, λ_{exc} = 460 nm) λ [nm] 656, 706, 782. **IR** (ATR) $\tilde{\nu}$ [cm^{-1}] 3082, 3031, 3005, 2962, 2906, 2866, 2847, 1783, 1689, 1604, 1566, 1446, 1405, 1332, 1307, 1258, 1138, 1017, 898, 783, 721, 696.

***meso*-Tetrakis(2,6-dichlorophenyl)tetrabenzoporphyrin 12**

Purified by filtration over silica gel (CH_2Cl_2 + 1% TFA) and subsequent recrystallization from CH_2Cl_2/NEt_3 with MeOH, 654 mg, 57%. **^{1}H NMR** (300 MHz, CD_2Cl_2/TFA-d_1, rt) δ [ppm] 8.00–7.90 (m, 12H), 7.67–7.63 (AA'BB', 8H), 7.60–7.56 (AA'BB', 8H). **^{13}C NMR** (100 MHz, $CDCl_3$/TFA-d_1, rt) δ [ppm] 140.4, 139.7, 135.5, 133.2, 131.1, 130.6, 129.8, 123.2, 108.0. **HRMS** (APPI, toluene) m/z calcd for $C_{60}H_{30}Cl_8N_4$ $[M]^+$ 1085.9973, found 1085.9972. **UV/Vis** (CH_2Cl_2 + 1% NEt_3) λ [nm] (ε [$M^{-1}cm^{-1}$]) 418 (37900), 451 (188000), 463 (232000), 596 (13300), 629 (32600), 645 (39800), 703 (21300). **Fluorescence** (CH_2Cl_2 + 1% NEt_3, λ_{exc} = 463 nm) λ [nm] 658, 709, 787. **IR** (ATR) $\tilde{\nu}$ [cm^{-1}] 3366, 3342, 3075, 2922, 2852, 1554, 1516, 1448, 1429, 1360, 1317, 1290, 1194, 1137, 1124, 1087, 1035, 838, 785, 760, 720, 618.

***meso*-Tetrakis(2-bromophenyl)tetrabenzoporphyrin 13**

Purified by filtration over silica gel (CH_2Cl_2 + 1% TFA) and subsequent recrystallization from CH_2Cl_2/NEt_3 with MeOH, 906 mg, 76%. **^{1}H NMR** (400 MHz, $CDCl_3$/TFA-d_1, rt) δ [ppm] 8.57–8.39 (m, 4H), 8.20–8.16 (m, 4H), 7.93–7.85 (m, 8H), 7.58–7.36 (m, 16H). **^{13}C NMR** (100 MHz, $CDCl_3$/TFA-d_1, rt) δ [ppm] 142.2, 142.1, 141.5, 141.3, 141.0, 140.7, 140.5, 140.3, 139.8, 138.9, 138.8, 137.2, 137.1, 136.94, 136.85, 136.8, 136.6, 136.5, 136.3, 134.6, 134.54, 134.49, 134.4, 133.0, 132.7, 131.7, 131.3, 131.24, 131.16, 131.1, 130.9, 130.70, 130.67, 130.6, 130.54, 130.46, 129.14, 129.10, 129.0,

128.94, 128.90, 128.85, 128.73, 128.65, 128.6, 128.5, 124.9, 124.6, 124.54, 124.46, 124.3, 124.2, 123.8, 123.43, 123.41, 123.36, 123.0, 114.1, 113.1, 112.2. The NMR data refer to a mixture of 4 atropisomers. **HRMS** (APPI, toluene) m/z calcd for $C_{60}H_{34}Br_4N_4$ $[M]^+$ 1125.9511, found 1125.9516. **UV/Vis** (CH_2Cl_2 + 1% NEt_3) λ [nm] (ε [$M^{-1}cm^{-1}$]) 424 (37900), 454 (206000), 464 (290000), 593 (14000), 629 (35000), 642 (41800), 700 (17600). **Fluorescence** (CH_2Cl_2 + 1% NEt_3, λ_{exc} = 464 nm) λ [nm] 655, 705, 783. **IR** (ATR) $\tilde{\nu}$ [cm^{-1}] 3369, 3345, 3071, 3046, 1511, 1466, 1447, 1424, 1367, 1319, 1288, 1140, 1124, 1060, 1023, 948, 871, 827, 763, 746, 721, 648, 616.

meso-Tetrakis(2,6-dibromophenyl)tetrabenzoporphyrin 14

Purified by filtration over silica gel (CH_2Cl_2 + 1% TFA) and subsequent recrystallization from CH_2Cl_2/NEt_3 with MeOH, 664 mg, 44%. **^{1}H NMR** (400 MHz, aceton-d_6/TFA-d_1/CS_2, rt) δ [ppm] 8.31 (d, $^3J_{HH}$= 8.1 Hz, 8H), 7.93 (t, $^3J_{HH}$= 8.1 Hz, 4H), 7.82–7.80 (AA'BB', 8H), 7.69–7.66 (AA'BB', 8H). **^{13}C NMR** (100 MHz, CD_2Cl_2/TFA-d_1, rt) δ [ppm] 140.7, 140.5, 139.3, 134.2, 134.1, 131.3, 131.2, 130.1, 123.8, 113.3. **HRMS** (ESI) m/z calcd for $C_{60}H_{31}Br_8N_4$ $[M+H]^+$ 1438.6010, found 1438.6022. **UV/vis** (CH_2Cl_2 + 1% NEt_3) λ [nm] (ε [$M^{-1}cm^{-1}$]) 425 (35500), 457 (184000), 468 (246000), 600 (13100), 632 (32700), 647 (41000), 706 (22200). **Fluorescence** (CH_2Cl_2 + 1% NEt_3, λ_{exc} = 468 nm) λ [nm] 501, 545, 660, 712, 793. **IR** (ATR) $\tilde{\nu}$ [cm^{-1}] 3078, 1738, 1681, 1546, 1513, 1448, 1425, 1365, 1319, 1290, 1185, 1124, 1070, 1032, 838, 830, 777, 761, 729, 705, 694, 629, 618.

meso-Tetrakis(4-trifluoromethylphenyl)tetrabenzoporphyrin 15

Purified by filtration over silica gel (CH_2Cl_2 + 1% TFA) and subsequent recrystallization from CH_2Cl_2/NEt_3 with MeOH, 660 mg, 66%. **^{1}H NMR** (400 MHz, CD_2Cl_2/TFA-d_1, rt) δ [ppm] 8.73 (d, $^3J_{HH}$= 8.2 Hz, 8H), 8.27 (d, $^3J_{HH}$= 8.2 Hz, 8H), 7.55–7.53 (AA'BB', 8H), 7.46–7.44 (AA'BB', 8H). **^{13}C NMR** (100 MHz, CD_2Cl_2/TFA-d_1, rt) δ [ppm] 141.5, 141.2, 141.0, 136.5, 133.4 (q, $^2J_{CF}$= 33.0 Hz), 131.38, 131.35, 131.0, 127.3 (m), 124.9, 124.7 (q, $^1J_{CF}$= 272.5 Hz), 114.3, 114.2. **^{19}F NMR** (282 MHz, CD_2Cl_2/TFA-d_1, rt) δ [ppm] -55.7 (s). **HRMS** (APPI, toluene) m/z calcd for $C_{64}H_{34}F_{12}N_4$ $[M]^+$ 1086.2586, found 1086.2562. **UV/Vis** (CH_2Cl_2 + 1% NEt_3) λ [nm] (ε [$M^{-1}cm^{-1}$]) 422 (34800), 464 (302000),

593 (12900), 629 (31500), 642 (37400), 700 (11800). **Fluorescence** (CH_2Cl_2 + 1% NEt_3, λ_{exc} = 464 nm) λ [nm] 659, 707, 784. **IR** (ATR) $\tilde{\nu}$ [cm^{-1}] 3086, 3006, 2937, 2866, 2767, 1670, 1613, 1448, 1409, 1322, 1204, 1167, 1107, 1067, 1018, 865, 825, 764, 736, 718, 701, 688.

meso-Tetrakis(4-cyanophenyl)tetrabenzoporphyrin 16

Purified by filtration over silica gel (CH_2Cl_2/THF, 9:1, v:v, + 1% TFA) and subsequent recrystallization from CH_2Cl_2 /NEt_3 with MeOH, 660 mg, 68%. **^{1}H NMR** (600 MHz, CD_2Cl_2/TFA-d_1, rt) δ [ppm] 8.73 (d, $^3J_{HH}$= 8.3 Hz, 8H), 8.29 (d, $^3J_{HH}$= 8.3 Hz, 8H), 7.55–7.53 (m, 8H), 7.43–7.41 (m, 8H). **^{13}C NMR** (150 MHz, CD_2Cl_2/TFA-d_1, rt) δ [ppm] 142.1, 140.9, 140.7, 136.7, 134.0, 131.2, 131.1, 130.9, 124.9, 118.6, 115.1, 114.03, 113.97, 113.9. **HRMS** (APPI, THF/MeCN) m/z calcd for $C_{64}H_{35}N_8$ $[M+H]^+$ 915.2979, found 915.2964. **UV/Vis** (CH_2Cl_2 + 1% NEt_3) λ [nm] (ε [$M^{-1}cm^{-1}$]) 423 (31800), 468 (273000), 596 (12200), 632 (29300), 645 (36200), 702 (11300). **Fluorescence** (CH_2Cl_2 + 1% NEt_3, λ_{exc} = 468 nm) λ [nm] 663, 712, 790. **IR** (ATR) $\tilde{\nu}$ [cm^{-1}] 3090, 2967, 2861, 2763, 2227, 1679, 1602, 1446, 1396, 1334, 1308, 1173, 1126, 1053, 1020, 867, 821, 795, 761, 747, 727, 701, 639.

meso-Tetrakis(4-nitrophenyl)tetrabenzoporphyrin 17

Purified by filtration over silica gel (THF) and subsequent recrystallization from CH_2Cl_2/NEt_3 with MeOH, 315 mg, 30%. **^{1}H NMR** (400 MHz, THF-d_8/TFA-d_1, rt) δ [ppm] 8.89 (d, $^3J_{HH}$= 8.8 Hz, 8H), 8.80 (d, $^3J_{HH}$= 8.8 Hz, 8H), 7.45 (br, 16H). **^{13}C NMR** (100 MHz, THF-d_8/TFA-d_1, rt) δ [ppm] 150.3, 144.6, 141.6, 137.9, 131.9, 130.5, 125.3, 114.3. **HRMS** (APPI, toluene) m/z calcd for $C_{60}H_{35}N_8O_8$ $[M+H]^+$ 995.2572, found 995.2571. **UV/Vis** (CH_2Cl_2 + 1% NEt_3) λ [nm] (ε [$M^{-1}cm^{-1}$]) 470 (112000), 600 (11100), 648 (22000), 707 (10400). **Fluorescence** (CH_2Cl_2 + 1% NEt_3, λ_{exc} = 470 nm) λ [nm] 553, 659, 720, 793. **IR** (ATR) $\tilde{\nu}$ [cm^{-1}] 3092, 3012, 2989, 2937, 2876, 2857, 1668, 1520, 1447, 1436, 1343, 1107, 1053, 1015, 873, 851, 759, 738, 719, 687.

***meso*-Tetrakis(2-nitrophenyl)tetrabenzoporphyrin 18**

Purified by filtration over silica gel (CH_2Cl_2) and subsequent recrystallization from CH_2Cl_2/NEt_3 with MeOH, 368 mg, 35%. **^{1}H NMR** (600 MHz, CD_2Cl_2/TFA-d_1, rt) δ [ppm] 8.63–8.43 (m, 8H), 8.16–8.07 (m, 8H), 7.54–7.37 (m, 12H), 7.09–6.97 (m, 4H). **^{13}C NMR** (150 MHz, CD_2Cl_2/TFA-d_1, rt) δ [ppm] 152.1, 151.9, 151.83, 151.79, 151.7, 141.1, 140.9, 140.7, 140.0, 139.8, 139.3, 139.2, 139.00, 138.96, 138.9, 138.8, 138.6, 138.42, 138.36, 135.2, 135.1, 135.0, 134.9, 134.8, 133.04, 133.01, 132.99, 132.96, 132.49, 132.46, 132.4, 132.3, 132.2, 132.12, 132.09, 131.9, 131.8, 131.7, 131.5, 131.3, 131.2, 131.14, 131.12, 131.09, 131.04, 131.01, 130.95, 130.92, 130.87, 130.8, 130.4, 130.2, 129.81, 129.76, 129.6, 126.9, 126.6, 126.53, 126.47, 126.4, 125.3, 125.2, 125.1, 125.0, 124.9, 123.83, 123.80, 123.7, 123.6, 123.5, 123.3, 111.6, 111.0, 110.7, 110.3, 109.8, 109.7. The NMR data refer to a mixture of 4 atropisomers. **HRMS** (MALDI) m/z calcd for $C_{60}H_{34}N_8O_8$ $[M+H]^+$ 994.2500, found 994.2494. **UV/Vis** (CH_2Cl_2 + 1% NEt_3) λ [nm] (ε [$M^{-1}cm^{-1}$]) 466 (198000), 597 (13800), 632 (30700), 644 (34400), 707 (13800). **Fluorescence** (CH_2Cl_2 + 1% NEt_3, λ_{exc} = 466 nm) λ [nm] 544, 608, 656, 715, 793. **IR** (ATR) $\tilde{\nu}$ [cm^{-1}] 3327, 3056, 1600, 1522, 1436, 1341, 1120, 1032, 946, 881, 835, 773, 715, 536, 494, 477.

***meso*-Tetrakis(4-methyl benzoate)tetrabenzoporphyrin 19**

Purified by filtration over silica gel (THF) and subsequent recrystallization from CH_2Cl_2/NEt_3 with MeOH, 735 mg, 67%. %. **^{1}H NMR** (600 MHz, THF-d_8/TFA-d_1, rt) δ [ppm] 8.74 (d, $^3J_{HH}$= 8.1 Hz, 8H), 8.59 (d, $^3J_{HH}$= 8.1 Hz, 8H), 7.45–7.41 (m, 16H), 4.07 (s, 12 H. **^{13}C NMR** (150 MHz, THF-d_8/TFA-d_1, rt) δ [ppm] 168.8, 143.5, 141.6, 137.0, 132.9, 132.0, 131.3, 130.2, 125.2, 115.0, 52.9. **HRMS** (APPI, toluene) m/z calcd for $C_{68}H_{46}N_4O_8$ $[M]^+$ 1046.3310, found 1046.3312. **UV/Vis** (CH_2Cl_2 + 1% NEt_3) λ [nm] (ε [$M^{-1}cm^{-1}$]) 424 (31500), 467 (254000), 593 (11800), 629 (24700), 643 (30400), 700 (8500). **Fluorescence** (CH_2Cl_2 + 1% NEt_3, λ_{exc} = 467 nm) λ [nm] 650, 707, 786. **IR** (ATR) $\tilde{\nu}$ [cm^{-1}] 3319, 3117, 3061, 3003, 2952, 2924, 2851, 1715, 1604, 1436, 1404, 1357, 1274, 1191, 1172, 1113, 1098, 1019, 962, 941, 870, 825, 769, 728, 715, 633.

meso-Tetrakis(4-hydroxyphenyl)tetrabenzoporphyrin 20

Synthesized according to the general protocol using 4-formylphenyl acetate. The resulting Ar_4TBP was purified by double filtration over silica (1. THF + 1% TFA; 2. THF/CH_2Cl_2, 9:1, v:v) and subsequent recrystallization from THF/TFA with ether, 560 mg, 51%. The solid was dissolved in THF (150 mL) and 10% aqueous sodium hydroxide solution (40 mL) was added. The mixture was heated under reflux for 16 h, before 10% hydrochloric acid was added to neutralize the mixture. After dilution with water, the precipitate was filtered and washed intensively with water. The crude product was purified by recrystallization from THF with pentane, 464 mg, 50%. **^{1}H NMR** (400 MHz, DMSO-d_6/NEt_3, rt) δ [ppm] 8.03 (d, $^3J_{HH}$= 8.3 Hz, 8H), 7.70–7.00 (m, 16H), 7.34 (d, $^3J_{HH}$= 8.3 Hz, 8H), -1.23 (br, 2H). **^{13}C NMR** (100 MHz, DMSO-d_6/NEt_3, rt) δ [ppm] 158.6, 135.1, 132.2, 126.0, 123.7, 116.1, 115.2. **HRMS** (APPI, toluene/DMSO) m/z calcd for $C_{60}H_{39}N_4O_4$ $[M+H]^+$ 879.2966, found 878.2963. **UV/Vis** (CH_2Cl_2 + 1% DMSO + 1% NEt_3) λ [nm] (ε [$M^{-1}cm^{-1}$]) 468 (248000), 588 (8900), 627 (20400), 638 (22500), 696 (3800). **Fluorescence** (CH_2Cl_2 + 1% DMSO + 1% NEt_3, λ_{exc} = 468 nm) λ [nm] 655, 702, 785. **IR** (ATR) $\tilde{\nu}$ [cm^{-1}] 3069, 2977, 2781, 2736, 2665, 2611, 2490, 2088, 1990, 1598, 1507, 1438, 1372, 1273, 1223, 1165, 1112, 1026, 817, 761, 695.

meso-Tetrakis(4-methoxyphenyl)tetrabenzoporphyrin 21

Purified by filtration over silica gel (CH_2Cl_2/THF, 14:1, v:v) and subsequent recrystallization from CH_2Cl_2/NEt_3 with MeOH, 503 mg, 51%. **^{1}H NMR** (400 MHz, acetone-d_6, rt) δ [ppm] 8.55 (d, $^3J_{HH}$= 8.0 Hz, 8H), 7.55 (d, $^3J_{HH}$= 8.0 Hz, 8H), 7.47 (pd, 16H), 4.17 (s, 12H). **^{13}C NMR** (100 MHz, acetone-d_6, rt) δ [ppm] 162.8, 142.7, 138.4, 133.2, 132.0, 129.5, 124.6, 115.8, 115.0. **HRMS** (APPI, toluene) m/z calcd for $C_{64}H_{46}N_4O_4$ $[M]^+$ 934.3514, found 934.3523. **UV/Vis** (CH_2Cl_2 + 1% NEt_3) λ [nm] (ε [$M^{-1}cm^{-1}$]) 424 (24000), 467 (291000), 590 (10500), 626 (23400), 639 (26700), 697 (5100). **Fluorescence** (CH_2Cl_2 + 1% NEt_3, λ_{exc} = 467 nm) λ [nm] 657, 703, 784. **IR** (ATR) $\tilde{\nu}$ [cm^{-1}] 3080, 3008, 2936, 2837, 2361, 2343, 1691, 1664, 1603, 1512, 1467, 1443, 1416, 1383, 1293, 1252, 1173, 1028, 823, 794, 763, 728, 718, 702, 610.

***meso*-Tetrakis(2,6-dimethoxyphenyl)tetrabenzoporphyrin 22**

Purified by filtration over silica gel (THF) and subsequent recrystallization from THF/NEt_3 with MeOH, 612 mg, 55%. **^{1}H NMR** (400 MHz, aceton-d_6/TFA-d_1, rt) δ [ppm] 8.07 (t, $^3J_{HH}$= 8.5 Hz, 4H), 7.90–7.88 (AA'BB', 8H), 7.71–7.69 (AA'BB', 8H), 7.33 (d, $^3J_{HH}$= 8.5 Hz, 8H). **^{13}C NMR** (100 MHz, aceton-d_6/TFA-d_1, rt) δ [ppm] 160.7, 142.0, 135.3, 131.7, 131.4, 123.7, 118.3, 107.1, 105.5, 57.1. **HRMS** (APPI, toluene) m/z calcd for $C_{68}H_{55}N_4O_8$ $[M+H]^+$ 1055.4014, found 1055.4008 **UV/Vis** (CH_2Cl_2 + 1% NEt_3) λ [nm] (ε [$M^{-1}cm^{-1}$]) 416 (29200), 442 (126000), 459 (263000), 588 (12400), 633 (37300), 695 (11000). **Fluorescence** (CH_2Cl_2 + 1% NEt_3, λ_{exc} = 459 nm) λ [nm] 657, 705, 780. **IR** (ATR) $\tilde{\nu}$ [cm^{-1}] 3005, 2935, 2837, 1684, 1667, 1579, 1472, 1431, 1333, 1305, 1254, 1186, 1137, 1106, 1050, 1028, 904, 837, 766, 749, 720, 699, 618.

***meso*-Tetrakis(3,5-dimethoxyphenyl)tetrabenzoporphyrin 23**

Purified by filtration over silica gel (CH_2Cl_2/THF, 1:2, v:v) and subsequent recrystallization from CH_2Cl_2/NEt_3 with MeOH, 193 mg, 69% (reaction batch was ¼ the size as it is described in the general procedure). **^{1}H NMR** (600 MHz, $CDCl_3$/TFA-d_1, rt) δ [ppm] 7.65 (d, $^3J_{HH}$= 2.2 Hz, 8H), 7.64–7.63 (AA'BB', 8H), 7.52–7.51 (AA'BB', 8H), 7.11 (t, $^3J_{HH}$= 2.2 Hz, 4H), 4.02 (s, 24H). **^{13}C NMR** (100 MHz, CD_2Cl_2/TFA-d_1, rt) δ [ppm] 161.4, 140.6, 140.4, 131.0, 130.9, 130.1, 124.4, 114.6, 114.5, 114.3, 102.8, 56.1. **HRMS** (MALDI, DCTB) m/z calcd for $C_{68}H_{54}N_4O_8$ $[M]^+$ 1054.3942, found 1054.3936. **UV/Vis** (CH_2Cl_2 + 1% NEt_3) λ [nm] (ε [$M^{-1}cm^{-1}$]) 420 (30000), 463 (295000), 589 (12100), 625 (30000), 636 (35500), 694 (10200). **Fluorescence** (CH_2Cl_2 + 1% NEt_3, λ_{exc} = 463 nm) λ [nm] 650, 700, 777. **IR** (ATR) $\tilde{\nu}$ [cm^{-1}] 3369, 3343, 3069, 3040, 3004, 2958, 2935, 2836, 1603, 1580, 1442, 1424, 1360, 1306, 1257, 1201, 1155, 1114, 1070, 1040, 943, 860, 818, 765, 746, 716, 688, 623.

***meso*-Tetrakis(3,4,5-trimethoxyphenyl)tetrabenzoporphyrin 24**

Purified by filtration over silica gel (CH_2Cl_2/THF, 1:2, v:v) and subsequent recrystallization from CH_2Cl_2/NEt_3 with MeOH, 702 mg, 56%. **^{1}H NMR** (400 MHz, acetone-d_6/TFA-d_1, rt) δ [ppm] 8.11 (s, 8H), 7.71–7.66 (AA'BB', 8H), 7.57–7.54 (AA'BB', 8H), 4.11 (s, 12H), 3.94 (s, 24H). **^{13}C NMR** (100 MHz, acetone-d_6/TFA-d_1, rt)

δ [ppm] 155.3, 142.5, 142.3, 134.7, 131.8, 130.2, 124.8, 115.7, 61.5, 57.2. **HRMS** (APPI, toluene/CH_2Cl_2) m/z calcd for $C_{72}H_{62}N_4O_{12}$ $[M]^+$ 1174.4359, found 1174.4363. **UV/Vis** (CH_2Cl_2 + 1% NEt_3) λ [nm] (ε [$M^{-1}cm^{-1}$]) 420 (30500), 463 (296000), 588 (13200), 625 (30600), 636 (36100), 693 (9900). **Fluorescence** (CH_2Cl_2 + 1% NEt_3, λ_{exc} = 463 nm) λ [nm] 652, 698, 777. **IR** (ATR) $\tilde{\nu}$ [cm^{-1}] 3333, 3068, 2997, 2932, 2830, 1577, 1503, 1446, 1408, 1369, 1233, 1165, 1118, 1032, 1005, 922, 862, 837, 804, 767, 723, 635.

meso-Tetrakis(2-furanyl)tetrabenzoporphyrin 25

Purified by filtration over silica gel (CH_2Cl_2) and subsequent recrystallization from CH_2Cl_2/NEt_3 with MeOH, 80 mg, 20%. **^{1}H NMR** (400 MHz, CD_2Cl_2/TFA-d_1, rt) δ [ppm] 8.28 (dd, $^3J_{HH}$= 1.7 Hz, $^4J_{HH}$= 0.6 Hz, 4H), 8.14 (br, 5H), 7.79 (d, $^3J_{HH}$= 3.3 Hz, 4H), 7.69 (br, 8H), 7.20 (dd, $^3J_{HH}$= 3.3 Hz, $^3J_{HH}$= 1.7 Hz, 4H), 7.19–6.85 (br, 3H). **^{13}C NMR** (100 MHz, CD_2Cl_2/TFA-d_1, rt) δ [ppm] 154.6, 146.8, 142.9, 142.7, 131.0, 130.9, 130.7, 123.7, 120.5, 114.3, 102.7. **HRMS** (ESI, MeCN) m/z calcd for $C_{52}H_{31}N_4O_4$ $[M+H]^+$ 775.2340, found 775.2345. **UV/Vis** (CH_2Cl_2 + 1% NEt_3) λ [nm] (ε [$M^{-1}cm^{-1}$]) 418 (36500), 461 (231000), 594 (13200), 633 (36200), 643 (41500), 700 (20900). **Fluorescence** (CH_2Cl_2 + 1% NEt_3, λ_{exc} = 461 nm) λ [nm] 656, 707, 787. **IR** (ATR) $\tilde{\nu}$ [cm^{-1}] 3330, 3303, 3150, 3122, 3075, 1592, 1507, 1446, 1353, 1319, 1287, 1263, 1204, 1151, 1139, 1122, 1045, 1015, 954, 885, 879, 872, 807, 749, 716, 616.

meso-Tetrakis(2-thiophenyl)tetrabenzoporphyrin 26

Purified by filtration over silica gel (CH_2Cl_2 +1% TFA) and subsequent recrystallization from CH_2Cl_2/NEt_3 with MeOH, 440 mg, 50%. **^{1}H NMR** (400 MHz, CD_2Cl_2/TFA-d_1, rt) δ [ppm] 8.24–8.21 (m, 8H), 7.73–7.65 (m, 12H), 7.62–7.55 (m, 8H). **^{13}C NMR** (100 MHz, CD_2Cl_2/TFA-d_1, rt) δ [ppm] 142.8, 142.7, 142.6, 142.53, 142.51, 142.4, 136.4, 136.3, 134.0, 133.93, 133.88, 133.86, 131.71, 131.68, 131.66, 131.6, 131.39, 131.35, 131.34, 131.30, 131.23, 131.20, 131.17, 131.1, 130.90, 130.87, 130.85, 130.82, 130.75, 130.7, 129.81, 129.77, 124.3, 124.2, 123.8, 123.7, 107.3, 107.0, 106.7. The NMR data refer to a mixture of 4 atropisomers. **HRMS** (MALDI, DCTB) m/z calcd for $C_{52}H_{30}N_4S_4$ $[M]^+$ 838.1353, found 838.1348. **UV/Vis** (CH_2Cl_2 + 1% NEt_3) λ [nm] (ε [$M^{-1}cm^{-1}$]) 424

(22700), 469 (232000), 596 (10000), 633 (22700), 645 (26500), 706 (9600). **Fluorescence** (CH_2Cl_2 + 1% NEt_3, λ_{exc} = 469 nm) λ [nm] 655, 711, 791. **IR** (ATR) $\tilde{\nu}$ [cm^{-1}] 3336, 3070, 2976, 1664, 1444, 1361, 1339, 1317, 1286, 1264, 1231, 1200, 1175, 1119, 1025, 894, 842, 831, 809, 798, 772, 738, 711, 615.

meso-Tetrakis(2-pyridyl)tetrabenzoporphyrin 27

Purified by double filtration over silica gel (1. CH_2Cl_2/THF, 1:1, v:v, + 1% AcOH; 2. CH_2Cl_2/THF, 4:1, v:v, + 1% NEt_3) and subsequent recrystallization from CH_2Cl_2/NEt_3 with MeOH, 125 mg, 15%. **^{1}H NMR** (400 MHz, DMSO-d_6/TFA-d_1, rt) δ [ppm] 9.37–9.36 (m, 4H), 9.11–8.99 (m, 4H), 8.71–8.65 (m, 4H), 8.31–8.28 (m, 4H), 7.56–7.54 (m, 8H), 7.10 (br, 8H). **^{13}C NMR** (100 MHz, DMSO-d_6/TFA-d_1, rt) δ [ppm] 154.6, 149.3, 141.0, 132.8, 132.0, 129.8, 126.9, 124.2, 112.6. **HRMS** (ESI, toluene/CH_2Cl_2) m/z calcd for $C_{56}H_{34}N_8$ $[M]^+$ 818.2901, found 818.2868. **UV/Vis** (CH_2Cl_2 + 1% NEt_3) λ [nm] (ε [$M^{-1}cm^{-1}$]) 417 (37600), 448 (192000), 460 (277000), 590 (13900), 624 (33600), 638 (42800), 694 (16700). **Fluorescence** (CH_2Cl_2 + 1% NEt_3, λ_{exc} = 460 nm) λ [nm] 652, 700, 777. **IR** (ATR) $\tilde{\nu}$ [cm^{-1}] 3053, 1582, 1561, 1448, 1425, 1361, 1319, 1209, 1123, 1044, 1034, 983, 860, 839, 777, 749, 719, 611.

meso-Tetrakis(2-naphthyl)tetrabenzoporphyrin 28

Purified by filtration over silica gel (CH_2Cl_2) and subsequent recrystallization from CH_2Cl_2/NEt_3 with MeOH, 375 mg, 35%. **^{1}H NMR** (400 MHz, CD_2Cl_2/TFA-d_1, rt) δ [ppm] 9.11 (br, 4H), 8.70 (pd, 4H), 8.49 (br, 4H), 8.32 (pd, 4H), 8.22 (pt, 4H), 7.88–7.81 (m, 8H), 7.40–7.32 (m, 16H). **^{13}C NMR** (100 MHz, CD_2Cl_2/TFA-d_1, rt) δ [ppm] 136.5, 134.7, 134.2, 130.5, 130.4, 130.3, 130.1, 129.7, 128.7, 127.9, 125.3, 125.0, 115.6. The NMR data refer to a mixture of 4 atropisomers. **HRMS** (APPI, toluene) m/z calcd for $C_{76}H_{47}N_4$ $[M+H]^+$ 1015.3795, found 1015.3788. **UV/Vis** (CH_2Cl_2 + 1% NEt_3) λ [nm] (ε [$M^{-1}cm^{-1}$]) 426 (27400), 470 (293000), 593 (12800), 630 (26200), 643 (31500), 700 (7100). **Fluorescence** (CH_2Cl_2 + 1% NEt_3, λ_{exc} = 470 nm) λ [nm] 661, 708, 786. **IR** (ATR) $\tilde{\nu}$ [cm^{-1}] 3051, 3012, 2989, 2927, 2851, 2360, 1682, 1654, 1444, 1333, 1275, 1260, 1187, 1128, 1053, 978, 930, 897, 861, 819, 750, 713, 669.

***meso*-Tetrakis(biphenyl)tetrabenzoporphyrin 29**

Purified by filtration over silica gel (CH_2Cl_2/THF, 24:1, v:v) and subsequent recrystallization from CH_2Cl_2/NEt_3 with MeOH, 700 mg, 59%. **^{1}H NMR** (400 MHz, CD_2Cl_2, rt) δ [ppm] 8.70 (d, $^3J_{HH}$= 8.3 Hz, 8H), 8.24 (d, $^3J_{HH}$= 8.3 Hz, 8H), 8.04 (pdd, 8H), 7.66 (pt, 8H), 7.61–7.58 (AA'BB', 8H), 7.56–7.52 (m, 8H), 7.43–7.40 (AA'BB', 8H), 2.74 (br, 4H). **^{13}C NMR** (100 MHz, CD_2Cl_2, rt) δ [ppm] 143.2, 141.8, 140.4, 138.7, 136.9, 131.6, 129.5, 129.3, 128.6, 128.3, 127.8, 124.6, 114.9. **HRMS** (APPI, toluene) m/z calcd for $C_{84}H_{54}N_4$ $[M]^+$ 1118.4343, found 1118.4333. **UV/Vis** (CH_2Cl_2 + 1% NEt_3) λ [nm] (ε [$M^{-1}cm^{-1}$]) 424 (27900), 468 (316000), 590 (13400), 627 (26400), 641 (33000), 698 (7300). **Fluorescence** (CH_2Cl_2 + 1% NEt_3, λ_{exc} = 468 nm) λ [nm] 658, 706, 786. **IR** (ATR) $\tilde{\nu}$ [cm^{-1}] 3028, 2926, 2849, 2346, 1687, 1654, 1600, 1529, 1485, 1445, 1403, 1335, 1190, 1135, 1056, 1006, 821, 796, 758, 718, 695, 656.

***meso*-Tetrakis(1-(*tert*-butyl)-4-(phenylethynyl)phenyl)tetrabenzoporphyrin 30**

Purified by filtration over silica gel (CH_2Cl_2 + 1% TFA) and subsequent recrystallization from CH_2Cl_2/NEt_3 with MeOH, 729 mg, 48%. **^{1}H NMR** (400 MHz, $CDCl_3$/TFA-d_1, rt) δ [ppm] 8.50 (d, $^3J_{HH}$= 8.2 Hz, 8H), 8.10 (d, $^3J_{HH}$= 8.2 Hz, 8H), 7.66 (d, $^3J_{HH}$= 8.3 Hz, 8H), 7.57–7.48 (m, 24H), 1.39 (s, 36H). **^{13}C NMR** (100 MHz, $CDCl_3$/TFA-d_1, rt) δ [ppm] 152.5, 141.0, 140.8, 137.8, 136.0, 135.7, 132.9, 131.7, 130.9, 130.3, 128.6, 126.7, 126.1, 125.7, 124.3, 119.7, 114.5, 114.4, 93.4, 88.3, 35.0, 31.2. **HRMS** (APPI, toluene) m/z calcd for $C_{108}H_{86}N_4$ $[M]^+$ 1438.6847, found 1438.6859. **UV/Vis** (CH_2Cl_2 + 1% NEt_3) λ [nm] (ε [$M^{-1}cm^{-1}$]) 431 (34500), 471 (267000), 593 (12900), 629 (22300), 643 (27900), 699 (6700). **Fluorescence** (CH_2Cl_2 + 1% NEt_3, λ_{exc} = 471 nm) λ [nm] 661, 708, 789. **IR** (ATR) $\tilde{\nu}$ [cm^{-1}] 3337, 3320, 2960, 2923, 2853, 2213, 1913, 1707, 1600, 1576, 1518, 1448, 1362, 1264, 1123, 1103, 1032, 1018, 940, 857, 833, 801, 749, 727, 652.

***meso*-Tetrakis((3',4',5'-tris(4-(*tert*-butyl)phenyl)-6'-phenyl-[1,1':2',1''-terphenyl]-4-yl))tetrabenzoporphyrin 31**

Purified by filtration over silica gel (CH_2Cl_2 +1% TFA) and subsequent recrystallization from CH_2Cl_2/NEt_3 with MeOH, 1.04 g, 59% (reaction batch was ½ the size as it is descriped in the general procedure). **^{1}H NMR** (600 MHz, CD_2Cl_2/TFA-d_1, rt) δ [ppm]

8.02 (d, $^3J_{HH}$= 8.2 Hz, 8H), 7.49 (d, $^3J_{HH}$= 8.2 Hz, 8H), 7.28–7.26 (AA'BB', 8H), 7.23 (pseudo-d, 8H), 7.19–7.15 (m, 16H), 7.14–7.111 (m, 8H), 7.10–7.08 (m, 8H), 6.95–6.93 (m, 16H), 6.91 (d, $^3J_{HH}$= 8.4 Hz, 8H), 6.88–6.84 (m, 16H), 6.81 (d, $^3J_{HH}$= 8.4 Hz, 8H), 6.80–6.78 (AA'BB', 8H). **^{13}C NMR** (150 MHz, CD_2Cl_2/TFA-d_1, rt) δ [ppm] 148.5, 148.3, 144.8, 141.9, 141.4, 141.3, 141.2, 140.5, 139.4, 138.2, 138.1, 135.1, 135.0, 133.0, 132.2, 131.9, 131.5, 131.4, 130.44, 130.41, 130.0, 127.3, 127.1, 126.1, 123.9, 123.74, 123.71, 123.6, 114.62, 114.61, 34.42, 34.39, 31.32, 31.31. **HRMS** (MALDI, DCTB) m/z calcd for $C_{252}H_{230}N_4$ $[M]^+$ 3311.8115, found 3312.8124. **UV/Vis** (CH_2Cl_2 + 1% NEt_3) λ [nm] (ε [$M^{-1}cm^{-1}$]) 483 (290000), 598 (14800), 642 (20800), 654 (23700), 707 (4700). **Fluorescence** (CH_2Cl_2 + 1% NEt_3, λ_{exc} = 483 nm) λ [nm] 665, 713, 791. **IR** (ATR) $\tilde{\nu}$ [cm^{-1}] 3044, 2957, 2904, 2868, 2365, 2166, 1967, 1795, 1663, 1602, 1509, 1451, 1394, 1267, 1192, 1111, 1018, 941, 916, 821, 748, 617, 511.

General procedure to *meso*-tetraaryltetrabenzoporphyrinato-Pd^{II} complexes:

$PdCl_2$ (20 eq.) was suspended in PhCN (10 ml) and heated to 108 °C under nitrogen atmosphere. After all inorganics were dissolved, the respective TATBP (50 mg) was added and the mixture was reacted at 180 °C for 1 h. The solvent was removed in vacuo and the residue was purified by plug filtration (silica gel, CH_2Cl_2). The bright green fraction was concentrated, and the residue precipitated from CH_2Cl_2 and MeOH. Yields varied from 80% to 95%.

3 X-RAY CRYSTALLOGRAPHIC DATA

X-ray analysis of **2**

Single clear light blue block crystals of **2** crystallized from a mixture of CH_2Cl_2/TFA and methanol by solvent layering. A solvent mask was calculated and 290 electrons were found in a volume of 1115 Å^3 in 1 void per unit cell. This is consistent with the presence of 8[CH_3OH] per asymmetric unit, which account for 288 electrons per unit cell. The value of Z' is 0.25.

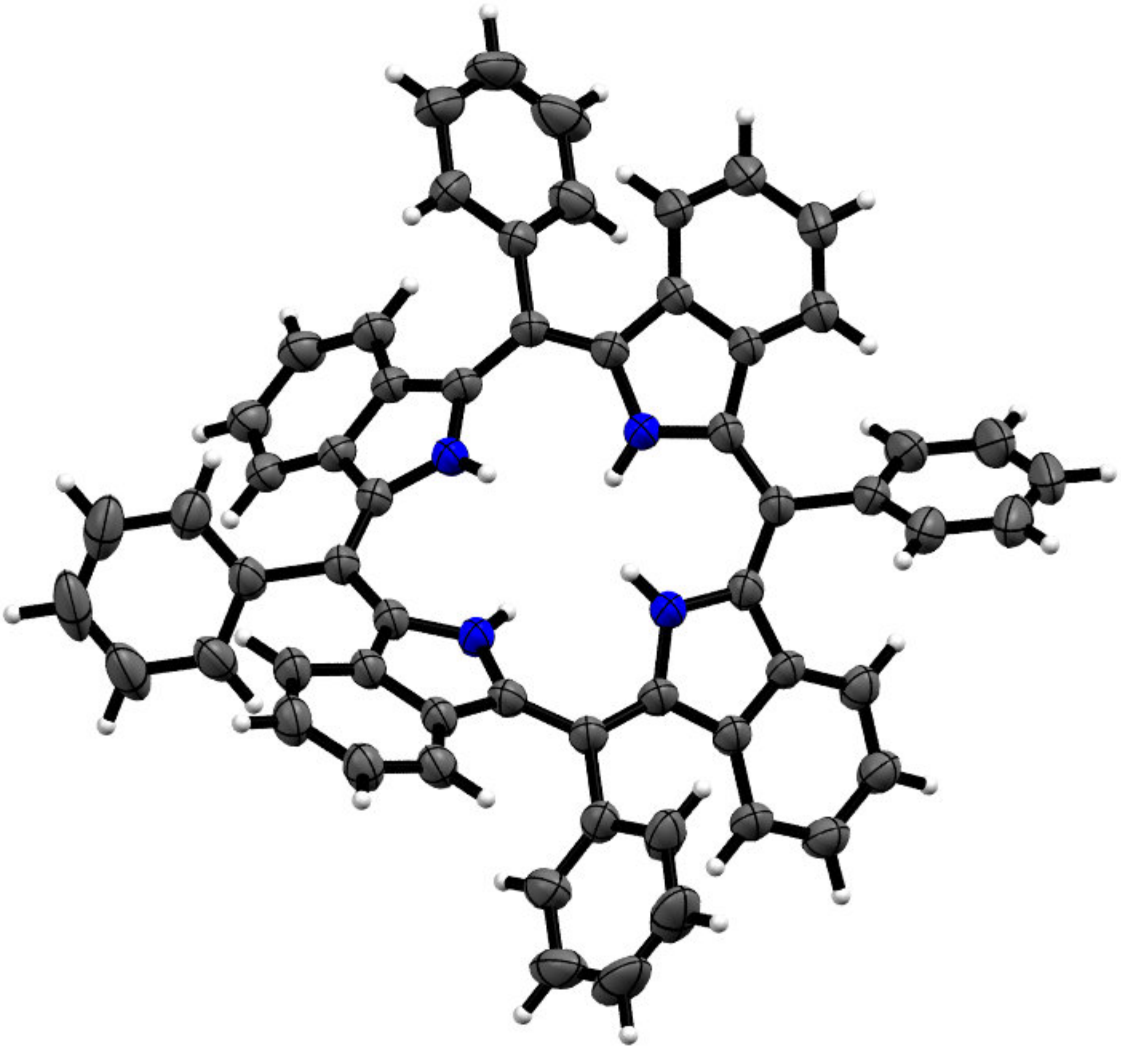

Figure S1. Crystal structure of **2**. Thermal ellipsoids are drawn at 50 % probability level. CCDC: 1950253.

X-ray analysis of **4**

Single metallic dark blue block crystals of **4** crystallized from a mixture of $CHCl_3$ and methanol by solvent layering. Because of a very low diffraction power of the crystal and a related only very average crystal quality the data collection was limited to 0.9 A resolution, to avoid too much collection/accumulation of background noise, but no reflections. 3 molecules (probably partially occupied) of TCM have been found in the asym unit with very bad values and strong undirected disorder. For quality reasons, these areas have been excluded for refinement by the Solvent Mask functionality of Olex2. A solvent mask was calculated and 640 electrons were found in a volume of 2224 $Å^3$ in 3 voids per unit cell. This is consistent with the presence of 3[$CHCl_3$] per asymmetric unit, which account for 696 electrons per unit cell.

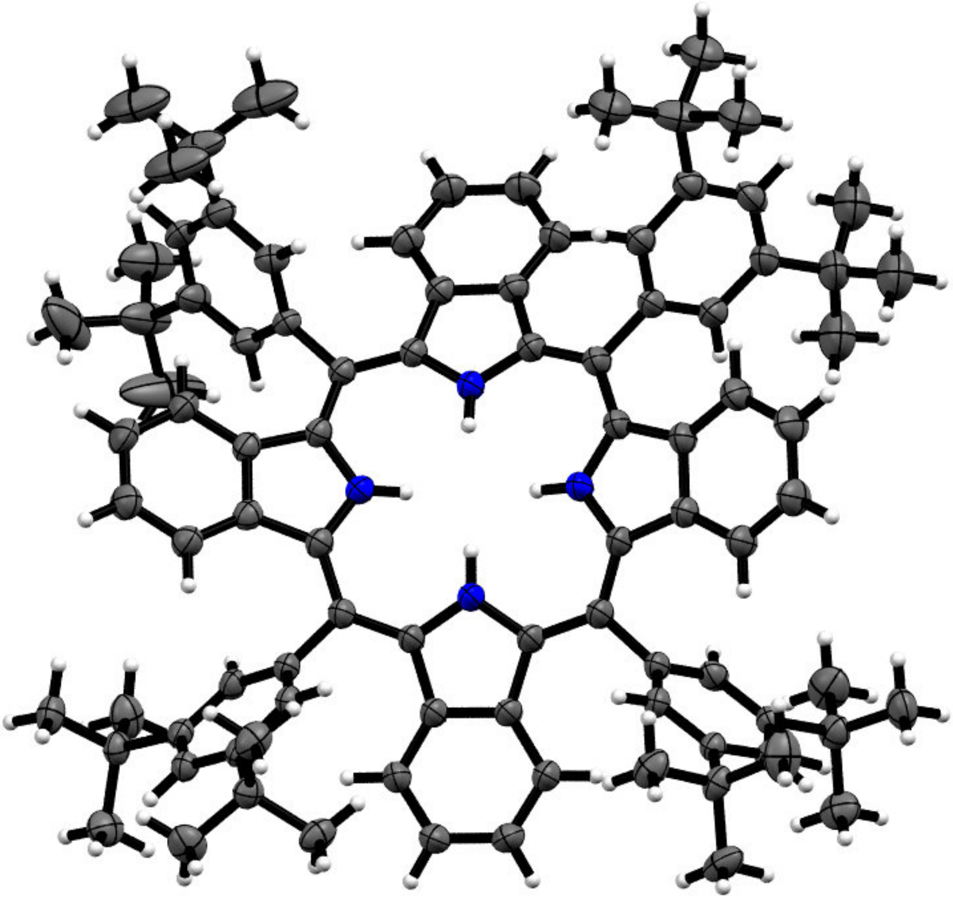

Figure S2. Crystal structure of **4**. Thermal ellipsoids are drawn at 50 % probability level. CCDC: 1950254.

X-ray analysis of **9**

Single clear dark blue needle crystals of **9** crystallized from a mixture of DCM and pentane by solvent layering. Disorder of TFA: F101-F103 : F104-106= 71 : 29% occupation. Structure was refined as racemic twin (BASF= 0.4); Two molecules of disordered, partial occupied DCM molecules with quite weak data have been masked for refinement with Olex2 Solvent mask. A solvent mask was calculated, and 188 electrons were found in a volume of 676 Å^3 in 2 voids per unit cell. This is consistent with the presence of 0.6[CH_2Cl_2], 0.5[CH_2Cl_2] per asymmetric unit, which account for 185 electrons per unit cell. There is a single molecule in the asymmetric unit, which is represented by the reported sum formula. In other words: Z is 4 and Z' is 1.

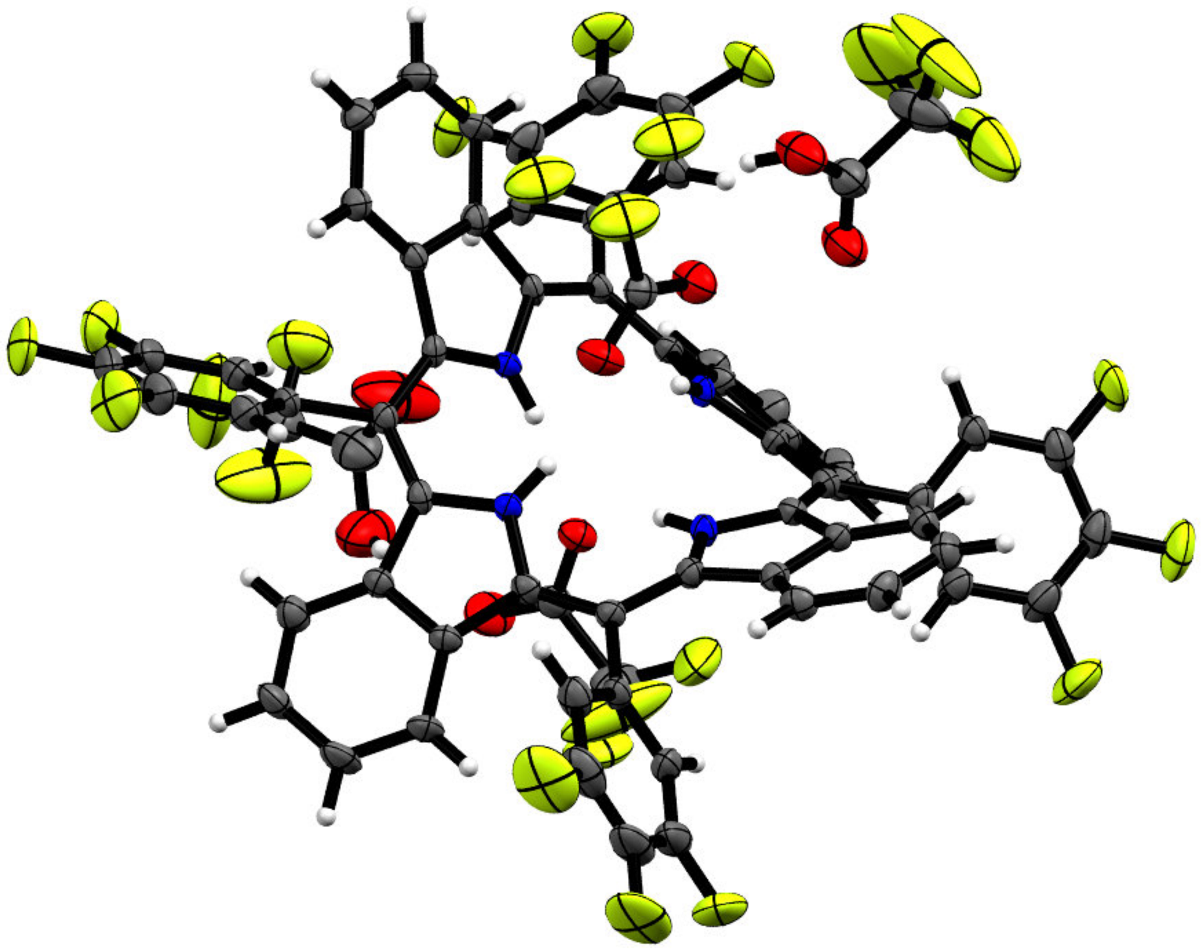

Figure S3. Crystal structure of **9**. Thermal ellipsoids are drawn at 50 % probability level. CCDC: 1950260.

X-ray analysis of **12Pd**

Single clear dark violet block crystals of **12Pd** crystallized from DMAc solution. Because of quite poor diffraction power of the crystal the data collection was limited to 0.9 Å resolution, to avoid too much collection/accumulation of background noise, but no suitable reflections. The following disorder was observed and refined: C103/N101 : N106/C107= 62 : 38%. The value of Z' is 0.5. This means that only half of the formula unit is present in the asymmetric unit, with the other half consisting of symmetry equivalent atoms.

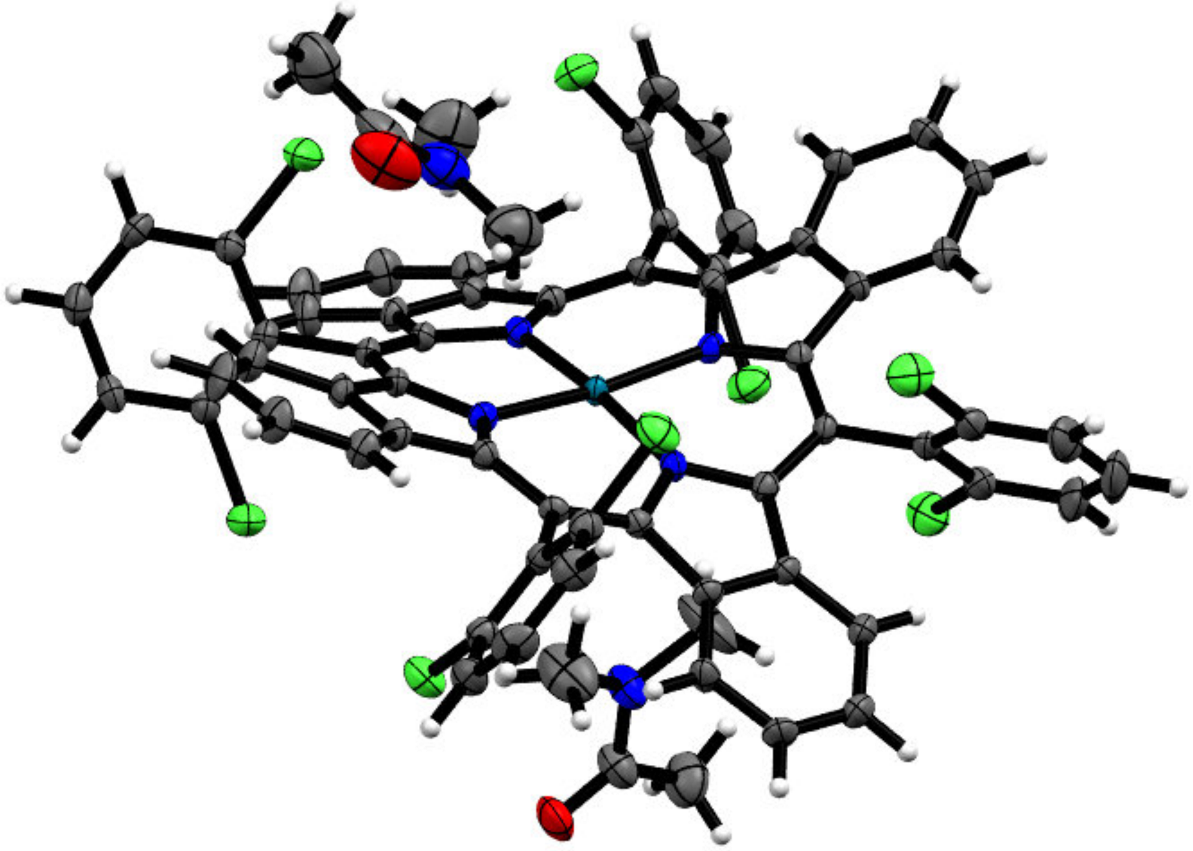

Figure S4. Crystal structure of **12Pd**. Thermal ellipsoids are drawn at 50 % probability level. CCDC: 1950258.

X-ray analysis of **14**

Single clear dark blue block crystals of **14** crystallized from CH_2Cl_2/TFA/Pentane/Et_2O by vapour diffusion. Two CF_3-groups were disordered and refined as follows: F21/22/23 : F21a/22a/23a= 50 : 50%, and F11/12/13 : F11a/12a/13a= 61 : 39% occupation. A solvent mask was calculated, and 832 electrons were found in a volume of 2844 $Å^3$ in 2 voids per unit cell. This is consistent with the presence of 1.5[CH_2Cl_2], 1[$C_4H_{10}O$] per asymmetric unit which account for 840 electrons per unit cell. There is a single molecule in the asymmetric unit, which is represented by the reported sum formula. In other words: Z is 8 and Z' is 1.

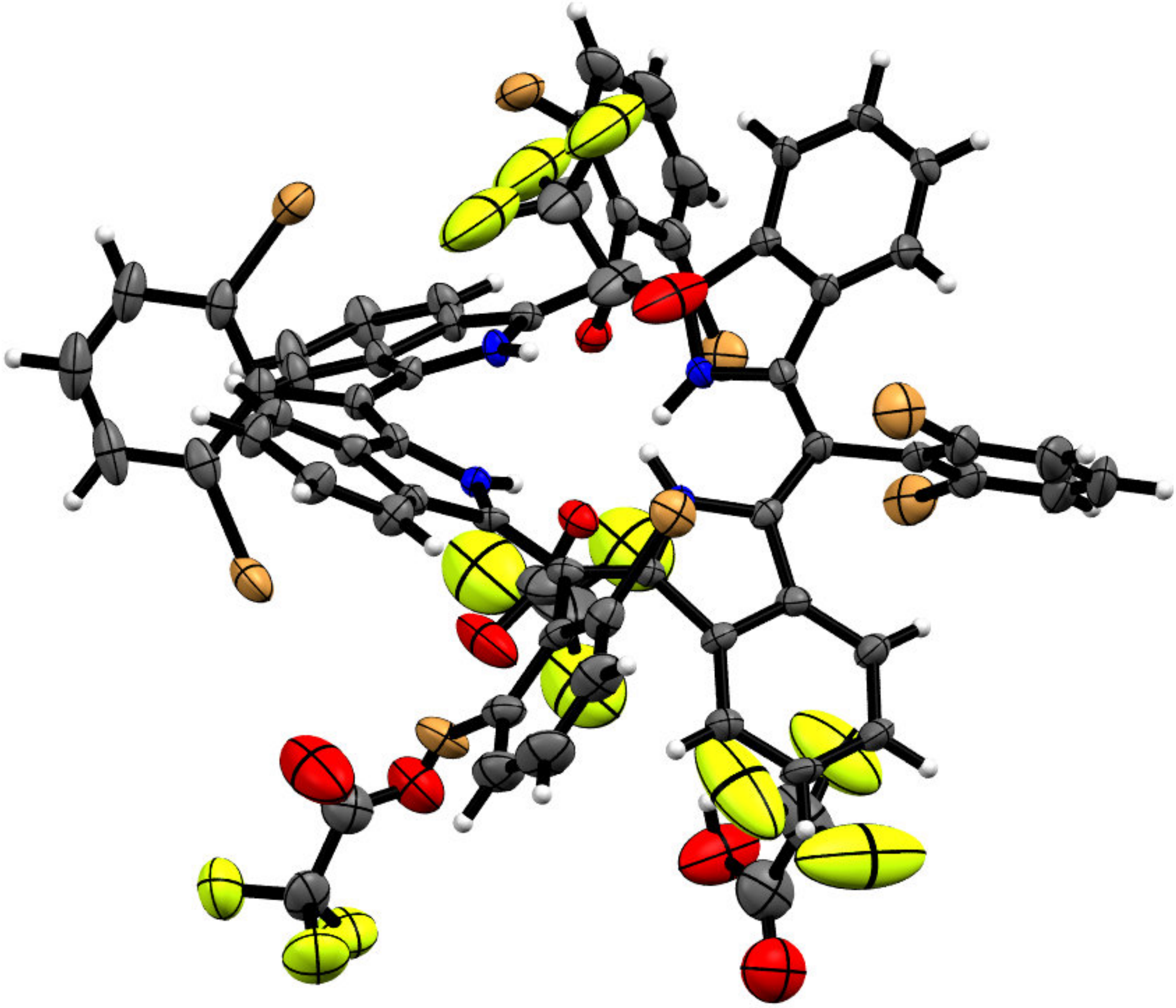

Figure S5. Crystal structure of **14**. Thermal ellipsoids are drawn at 50 % probability level. CCDC: 1950257.

X-ray analysis of **16**

Single clear dark green block crystals of **16** crystallized from a mixture of CH_2Cl_2/TFA and diethylether by solvent layering. A solvent mask was calculated, and 184 electrons were found in a volume of 776 $Å^3$ in 1 void per unit cell. This is consistent with the presence of 1[CH_2Cl_2] per Asymmetric Unit which account for 168 electrons per unit cell. There is a single molecule in the asymmetric unit, which is represented by the reported sum formula. In other words: Z is 4 and Z' is 1.

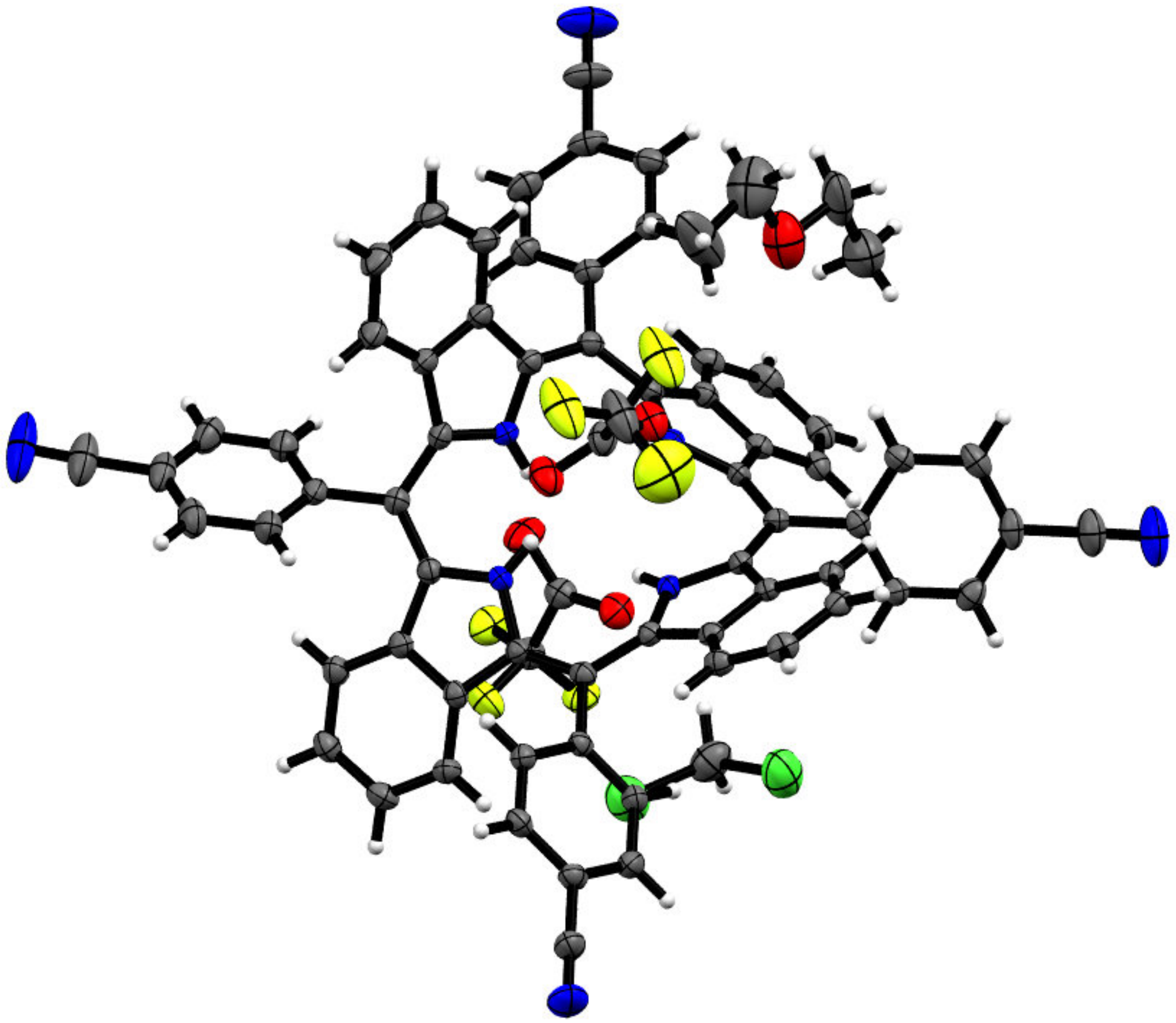

Figure S6. Crystal structure of **16**. Thermal ellipsoids are drawn at 50 % probability level. CCDC: 1950255.

X-ray analysis of **23**

Single clear greenish yellow plate crystals of **23** crystallized from a mixture of $CHCl_3$/TFA and methanol by solvent layering. The CF_3-group of one TFA molecule showed disorder, and was refined to 54 : 46 % occupation. There is a single molecule in the asymmetric unit, which is represented by the reported sum formula. In other words: Z is 2 and Z' is 1.

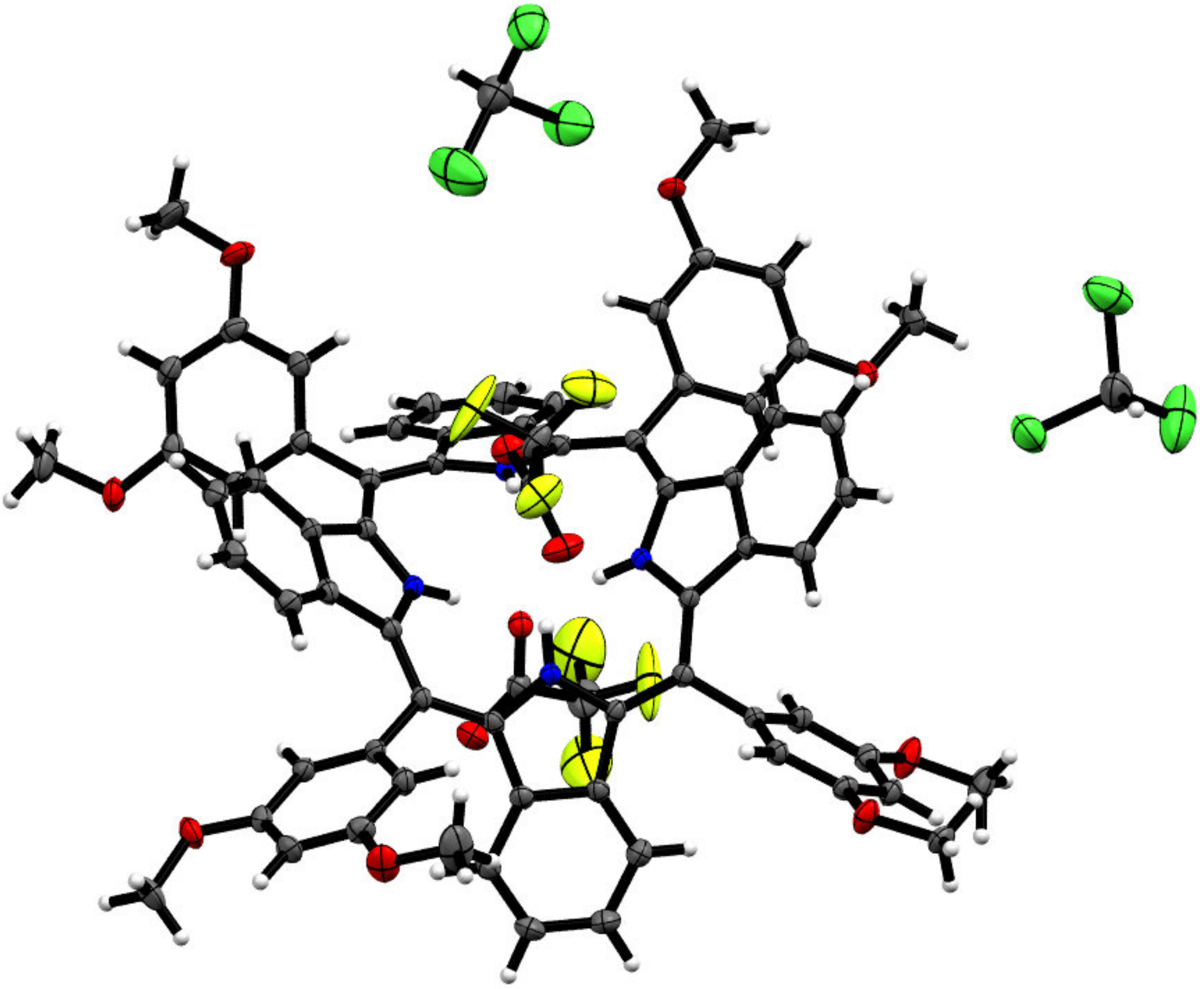

Figure S7. Crystal structure of **23**. Thermal ellipsoids are drawn at 50 % probability level. CCDC: 1950261.

X-ray analysis of **26**

Single metallic dark blue trapezoid crystals of **26** crystallized from a mixture of DCM and MeOH/NEt3 by solvent layering. Disorder refinement of C42/S45 : C42a/S45a= 55 : 45% occupation. Component 2 (twin) is rotated by -103.0763° around [-0.35 -0.03 0.94] (reciprocal) or [-0.58 -0.05 0.81] (direct). A solvent mask was calculated, and 136 electrons were found in a volume of 408 $Å^3$ in 1 void per unit cell. This is consistent with the presence of 2[CH_3OH] per asymmetric unit, which account for 144 electrons per unit cell. The value of Z' is 0.25.

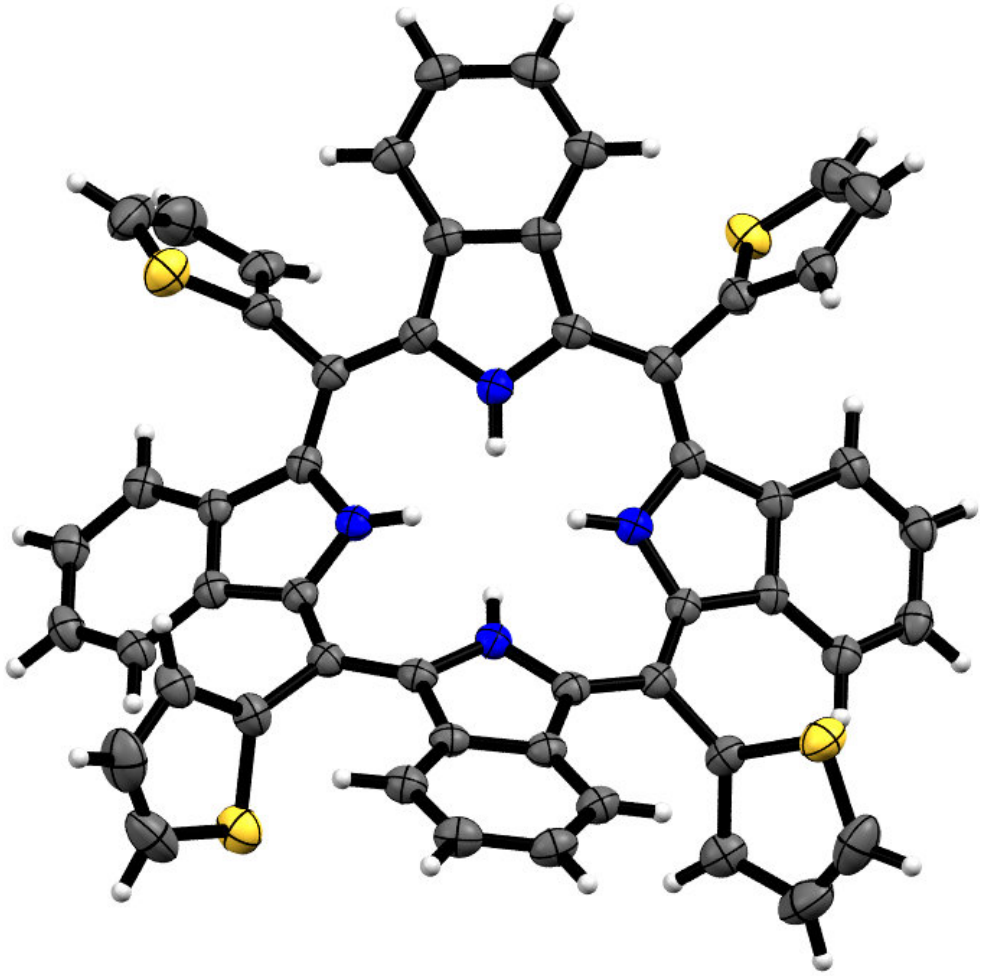

Figure S8. Crystal structure of **26**. Thermal ellipsoids are drawn at 50 % probability level. CCDC: 1950252.

X-ray analysis of **27**

Single clear dark blue prism crystals of **27** crystallized from a mixture of CH_2Cl_2/TFA and ether by solvent layering. Pyridyl-fragment shows disorder at C/N and was refined to 50 : 50% occupation. A solvent mask was calculated, and 313 electrons were found in a volume of 714 $Å^3$ in 1 void per unit cell. This is consistent with the presence of 4[CH_2Cl_2] per asymmetric unit, which account for 336 electrons per unit cell. The value of Z' is 0.25.

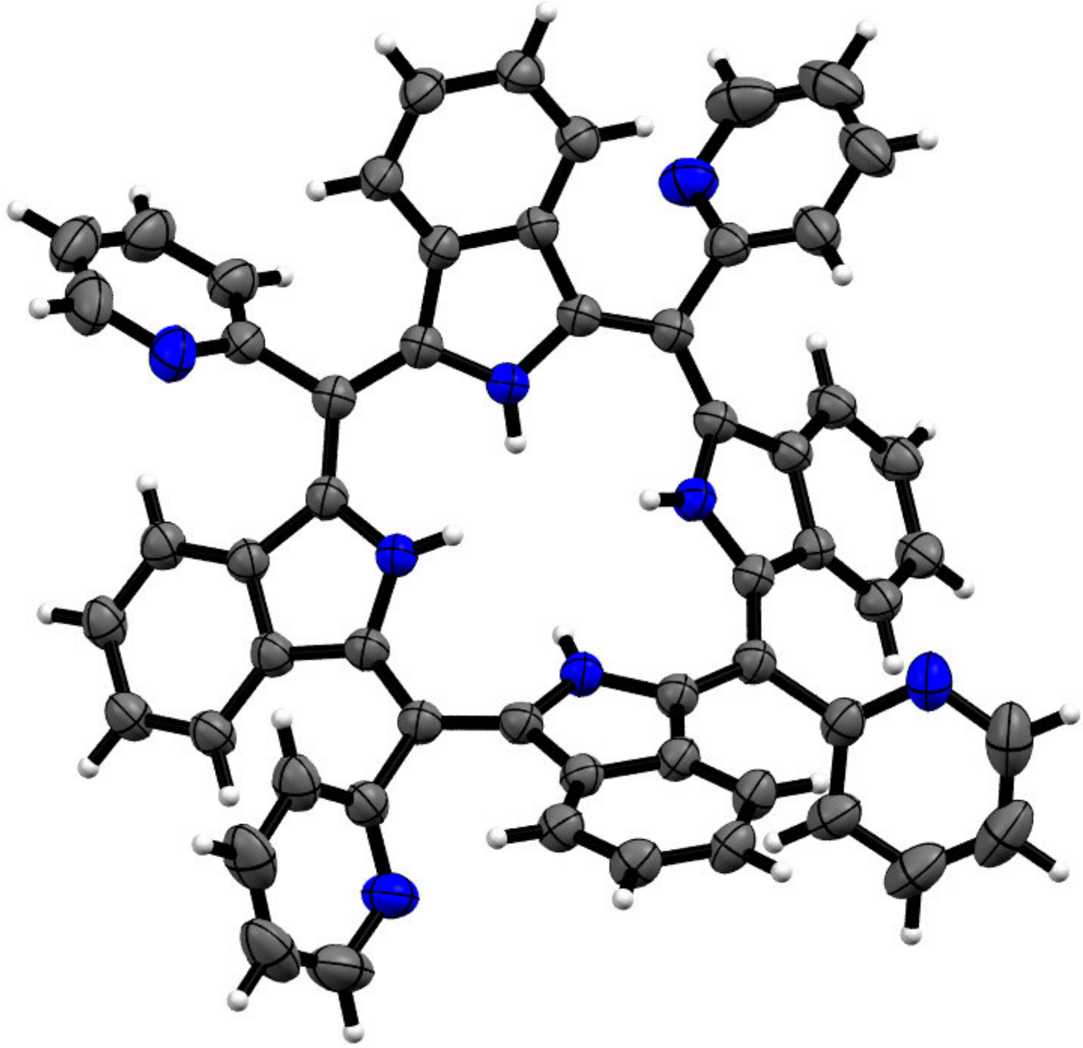

Figure S9. Crystal structure of **27**. Thermal ellipsoids are drawn at 50 % probability level. CCDC: 1950259.

X-ray analysis of **29**

Single clear dark blue octahedral crystals of **29** crystallized from a mixture of CH_2Cl_2/TFA and methanol by solvent layering. Absolute structure could not be determined reliably (Flack = 0.6). Therefore, the structure was refined as racemic twin. The Flack parameter was refined to 0.6(8). Determination of absolute structure using Bayesian statistics on Bijvoet differences using the Olex2 results in 0.4(5). Note: The Flack parameter is used to determine chirality of the crystal studied, the value should be near 0, a value of 1 means that the stereochemistry is wrong, and the model should be inverted. A value of 0.5 means that the crystal consists of a racemic mixture of the two enantiomers. The value of Z' is 0.5. This means that only half of the formula unit is present in the asymmetric unit, with the other half consisting of symmetry equivalent atoms.

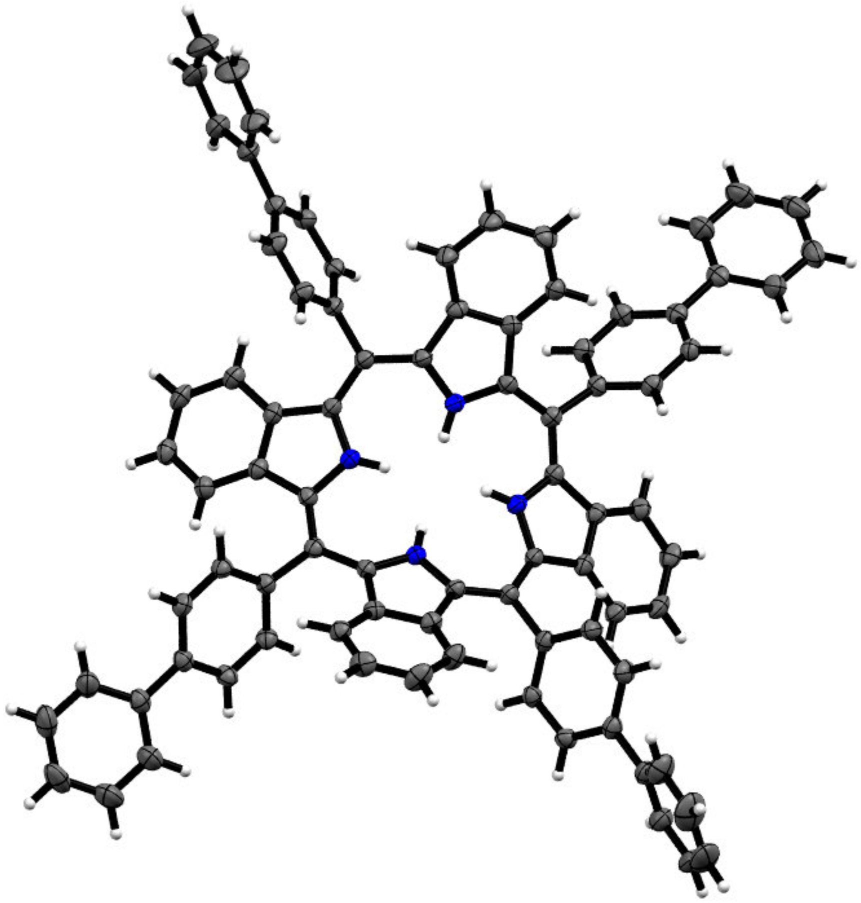

Figure S10. Crystal structure of **29**. Thermal ellipsoids are drawn at 50 % probability level. CCDC: 1950256.

Table S1. Crystal data and structure refinement for **2** (17Jux_MR01) and **4** (DL03).

Compound	**2** (17Jux_MR01)	**4** (DL03)
Formula	$C_{60}H_{38}N_4$	$C_{92}H_{102}N_4$
$D_{calc.}$/ g cm^{-3}	0.931	0.930
μ/mm^{-1}	0.420	0.400
Formula Weight	814.94	1263.77
Colour	clear light blue	metallic dark blue
Shape	block	block
Size/mm^3	0.31×0.27×0.14	0.24×0.22×0.21
T/K	152.90(14)	173.00(10)
Crystal System	tetragonal	monoclinic
Space Group	$P4_2/n$	$P2_1/c$
a/Å	13.9424(3)	30.6465(3)
b/Å	13.9424(3)	17.16260(15)
c/Å	14.9554(8)	17.64490(18)
α/°	90	90
β/°	90	103.4358(10)
γ/°	90	90
V/Å^3	2907.2(2)	9026.75(15)
Z	2	4
Z'	0.25	1
Wavelength/Å	1.54184	1.54184
Radiation type	Cu K_α	Cu K_α
Θ_{min}/°	4.335	3.642
Θ_{max}/°	72.702	61.547
Measured Refl's.	8981	23305
Ind't Refl's	2843	13457
Refl's with I > 2(I)	2027	11389
R_{int}	0.0341	0.0321
Parameters	146	891
Restraints	0	21
Largest Peak	0.433	0.365
Deepest Hole	-0.335	-0.421
GooF	1.097	1.039
wR_2 (all data)	0.2723	0.1556
wR_2	0.2522	0.1470
R_1 (all data)	0.0985	0.0638
R_1	0.0814	0.0559

Table S2. Crystal data and structure refinement for **9** (DL07) and **12Pd** (DL10).

Compound	**9***4(CF_3CO_2H) (DL07)	**12Pd***2(DMA) (DL10)
Formula	$C_{68}H_{30}F_{24}N_4O_8$	$C_{76}H_{64}Cl_8N_8O_4Pd$
$D_{calc.}$/ g cm^{-3}	1.515	1.442
μ/mm^{-1}	1.301	5.323
Formula Weight	1486.96	1543.35
Colour	clear dark blue	clear dark violet
Shape	needle	block
Size/mm^3	0.35×0.05×0.04	0.23×0.13×0.10
T/K	173.0(3)	173.00(10)
Crystal System	orthorhombic	monoclinic
Flack Parameter	0.42(19)	-
Hooft Parameter	0.50(4)	-
Space Group	$P2_12_12$	$C2/c$
a/Å	27.7585(4)	20.4528(2)
b/Å	16.3643(2)	20.2625(2)
c/Å	14.35137(15)	17.2570(2)
α/°	90	90
β/°	90	96.3392(12)
γ/°	90	90
V/Å^3	6519.06(13)	7108.00(15)
Z	4	4
Z'	1	0.5
Wavelength/Å	1.54184	1.54184
Radiation type	Cu K_α	Cu K_α
Θ_{min}/°	3.135	3.859
Θ_{max}/°	76.197	61.512
Measured Refl's.	27784	16336
Ind't Refl's	13033	5457
Refl's with I > 2(I)	11309	4642
R_{int}	0.0486	0.0480
Parameters	938	445
Restraints	15	8
Largest Peak	0.780	0.772
Deepest Hole	-0.588	-0.535
GooF	1.073	1.048
wR_2 (all data)	0.1943	0.1199
wR_2	0.1843	0.1097
R_1 (all data)	0.0854	0.0530
R_1	0.0684	0.0427

Table S3. Crystal data and structure refinement for **14** (MR03) and **16** (18Jux_MR02).

Compound	**14***4(CF_3CO_2H) (MR03)	**16***2(CF_3CO_2H)*(CH_2CL_2)*($C_4H_{10}O$) (18Jux_MR02)
Formula	$C_{68}H_{34}Br_8F_{12}N_4O_8$	$C_{73}H_{48}Cl_2F_6N_8O_5$
$D_{calc.}$/ g cm^{-3}	1.629	1.295
μ/mm^{-1}	5.654	1.496
Formula Weight	1902.27	1302.09
Colour	clear dark blue	clear dark green
Shape	block	block
Size/mm^3	0.20×0.15×0.08	0.29×0.13×0.11
T/K	173.00(14)	153.00(10)
Crystal System	orthorhombic	monoclinic
Space Group	*Pbcn*	$P2_1/c$
a/Å	49.6674(10)	13.07730(10)
b/Å	12.8782(2)	25.4133(3)
c/Å	24.2537(4)	20.6519(3)
α/°	90	90
β/°	90	103.4000(10)
γ/°	90	90
V/Å^3	15513.3(5)	6676.55(14)
Z	8	4
Z'	1	1
Wavelength/Å	1.54184	1.54184
Radiation type	Cu K_α	Cu K_α
Θ_{min}/°	3.752	3.474
Θ_{max}/°	61.592	64.644
Measured Refl's.	33866	20576
Ind't Refl's	11852	10751
Refl's with I > 2(I)	9515	8848
R_{int}	0.0321	0.0289
Parameters	892	843
Restraints	30	49
Largest Peak	1.177	1.199
Deepest Hole	-1.322	-0.860
GooF	1.037	1.053
wR_2 (all data)	0.2076	0.1915
wR_2	0.1956	0.1795
R_1 (all data)	0.0831	0.0743
R_1	0.0713	0.0636

Table S4. Crystal data and structure refinement for **23** (18Jux_MR01) and **26** (16Jux_MR03).

Compound	**23***2(CHCl3)*2(CF_3CO_2H) (18Jux_MR01)	**26** (16Jux_MR03_twin1_hklf4)
Formula	$C_{74}H_{56}Cl_6F_6N_4O_{12}$	$C_{52}H_{30}N_4S_4$
$D_{calc.}$/ g cm^{-3}	1.459	1.314
μ/mm^{-1}	2.974	2.383
Formula Weight	1519.92	839.04
Colour	clear greenish yellow	metallic dark blue
Shape	plate	trapezoid
Size/mm^3	0.19×0.14×0.04	0.37×0.28×0.20
T/K	153.00(10)	153.00(10)
Crystal System	triclinic	tetragonal
Space Group	P-1	$I4_1/a$
a/Å	13.1849(7)	14.50437(16)
b/Å	13.3256(6)	14.50437(16)
c/Å	20.7661(8)	20.1597(3)
α/°	74.789(4)	90
β/°	80.232(4)	90
γ/°	83.442(4)	90
V/Å^3	3460.3(3)	4241.12(11)
Z	2	4
Z'	1	0.25
Wavelength/Å	1.54184	1.54184
Radiation type	Cu K_α	Cu K_α
Θ_{min}/°	3.411	3.754
Θ_{max}/°	64.622	61.553
Measured Refl's.	18902	26371
Ind't Refl's	11119	1653
Refl's with I > 2(I)	9027	1556
R_{int}	0.0286	0.0809
Parameters	953	143
Restraints	10	4
Largest Peak	1.136	0.292
Deepest Hole	-0.744	-0.516
GooF	1.055	1.113
wR_2 (all data)	0.1440	0.1342
wR_2	0.1346	0.1316
R_1 (all data)	0.0644	0.0583
R_1	0.0511	0.0553

Table S5. Crystal data and structure refinement for **27** (16Jux_MR05) and **29** (16Jux_MR04).

Compound	**27** (16Jux_MR05)	**29** (16Jux_MR04_2)
Formula	$C_{56}H_{34}N_8$	$C_{84}H_{54}N_4$
$D_{calc.}$/ g cm^{-3}	0.996	1.309
μ/mm^{-1}	0.472	0.583
Formula Weight	818.91	1119.31
Colour	clear dark blue	clear dark blue
Shape	prism	octahedral
Size/mm^3	0.30×0.22×0.12	0.19×0.18×0.12
T/K	153.00(10)	153.1(5)
Crystal System	tetragonal	tetragonal
Flack Parameter	-	0.6(8)
Hooft Parameter	-	0.4(5)
Space Group	$P4_2/n$	$I4_1$
a/Å	13.7567(3)	20.20796(16)
b/Å	13.7567(3)	20.20796(16)
c/Å	14.4326(4)	13.91237(18)
α/°	90	90
β/°	90	90
γ/°	90	90
V/Å^3	2731.31(12)	5681.28(12)
Z	2	4
Z'	0.25	0.5
Wavelength/Å	1.54184	1.54184
Radiation type	Cu K_α	Cu K_α
Θ_{min}/°	4.440	3.093
Θ_{max}/°	73.489	64.931
Measured Refl's.	8302	7996
Ind't Refl's	2681	4611
Refl's with I > 2(I)	2223	4339
R_{int}	0.0230	0.0400
Parameters	146	398
Restraints	0	1
Largest Peak	1.039	0.177
Deepest Hole	-0.261	-0.211
GooF	1.072	1.046
wR_2 (all data)	0.2449	0.1054
wR_2	0.2348	0.1019
R_1 (all data)	0.0839	0.0432
R_1	0.0753	0.0404

4 NSD ANALYSIS

For Normal Structural Decomposition (NSD) analyses, we used the NSD engine program as provided by J. A. Shelnutt[7], which was handed to us by the Senge group from Dublin. The theory and development of this method have been described by Shelnutt and co-workers.[8-10] NSD employs the decomposition of the conformation of the porphyrin macrocycle by a basis set composed of its various normal modes of vibration. The result affords the quantitative separation of the contributing distortions to the macrocycle conformation.

The normal-modes for the out-of-plane distortions of the minimum basis consist of the lowest energy vibration from each symmetry and comprise the saddle (B_{2u}), ruffle (B_{1u}), domed (A_{2u}), propellered (A_{1u}) and the degenerate wave modes [$E_g(x)$ and $E_g(y)$]. The in-plane modes that compose the minimum basis are the *meso*-stretching (B_{2g}), N-stretching (B_{1g}), pyrrole-translation [$E_u(x)$ and $E_u(y)$], breathing (A_{1g}) and pyrrole-rotation (A_{2g}).[11,12]

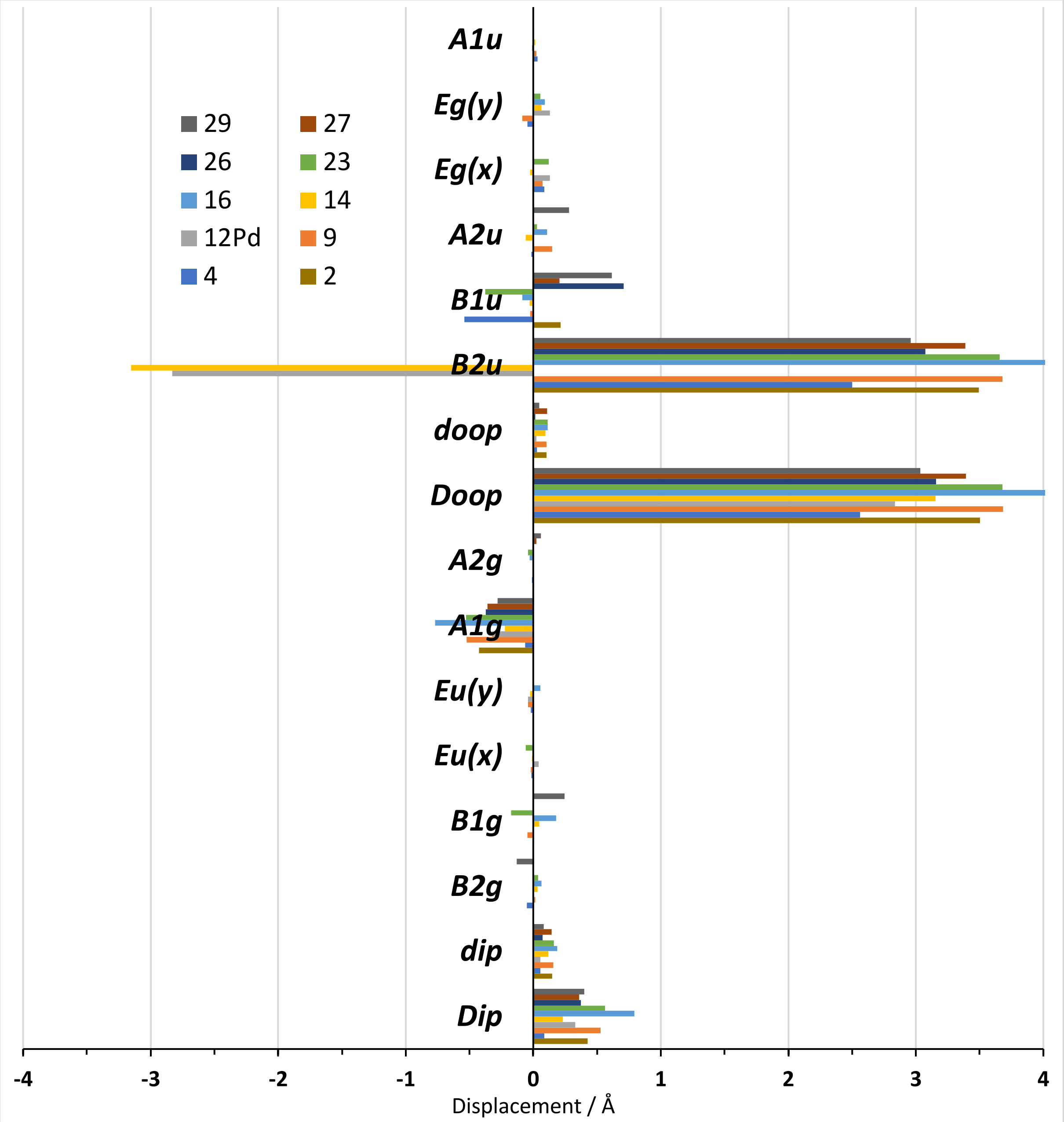

Figure S11. Complete NSD analysis **2**, **4**, **9**, **12Pd**, **14**, **16**, **23**, **26**, **27** and **29**.

2: NSD result generated from file 17jux_mr01.pdb; Summary of the NSD (in Å):

basis	Dip	dip	B2g	B1g	Eu(x)	Eu(y)	A1g	A2g
min.	0.4246	0.1473	-0.0000	0.0000	0.0005	-0.0002	-0.4246	0.0070
ext.	0.5858	0.1211	-0.0000	0.0000	0.0005	-0.0002	-0.4258	0.0077
			0.0000	0.0000	-0.0001	0.0000	-0.3974	0.0702
comp.	0.8874	0.0000	0.0000	0.0000	0.0012	0.0012	0.8843	0.0731
**basis**	**Doop**	**doop**	**B2u**	**B1u**	**A2u**	**Eg(x)**	**Eg(y)**	**A1u**
min.	3.5026	0.1051	3.4960	0.2145	0.0003	0.0000	0.0000	0.0010
ext.	3.5634	0.0103	3.4834	0.2145	0.0003	0.0000	0.0000	0.0010
			-0.6555	-0.0055	0.0006	0.0000	0.0000	0.0000
comp.	3.5639	0.0000	3.5575	0.2147	0.0007	0.0000	0.0000	0.0010

4: NSD result generated from file dl03.pdb; Summary of the NSD (in Å):

basis	Dip	dip	B2g	B1g	Eu(x)	Eu(y)	A1g	A2g
min.	0.0858	0.0560	-0.0486	0.0071	-0.0156	-0.0188	-0.0653	-0.0091
ext.	0.1892	0.0421	-0.0486	0.0071	-0.0156	-0.0188	-0.0657	-0.0102
			-0.0086	-0.0019	0.0012	-0.0019	-0.1241	-0.1138
comp.	0.3059	0.0000	0.0510	0.0098	0.0335	0.0241	0.2752	0.1159
**basis**	**Doop**	**doop**	**B2u**	**B1u**	**A2u**	**Eg(x)**	**Eg(y)**	**A1u**
min.	2.5622	0.0282	2.5024	-0.5394	-0.0155	0.0885	-0.0465	0.0343
ext.	2.5679	0.0038	2.4992	-0.5394	-0.0154	0.0881	-0.0460	0.0343
			-0.1695	0.0118	0.0029	-0.0099	0.0145	-0.0127
comp.	2.5680	0.0000	2.5082	0.5396	0.0158	0.0898	0.0510	0.0366

9: NSD result generated from file dl07.pdb; Summary of the NSD (in Å):

basis	Dip	dip	B2g	B1g	Eu(x)	Eu(y)	A1g	A2g
min.	0.5288	0.1553	0.0170	-0.0463	-0.0185	-0.0403	-0.5246	0.0066
ext.	0.6796	0.1279	0.0170	-0.0469	-0.0188	-0.0411	-0.5259	0.0065
			0.0007	-0.0334	-0.0271	-0.0672	-0.4193	-0.0117
comp.	0.9908	0.0000	0.0237	0.1193	0.0651	0.0921	0.9767	0.0154
**basis**	**Doop**	**doop**	**B2u**	**B1u**	**A2u**	**Eg(x)**	**Eg(y)**	**A1u**
min.	3.6844	0.1032	3.6796	-0.0233	0.1471	0.0748	-0.0836	0.0267
ext.	3.7386	0.0115	3.6675	-0.0233	0.1479	0.0768	-0.0829	0.0267
			-0.6313	0.0035	0.0195	0.0523	0.0180	-0.0079
comp.	3.7391	0.0000	3.7339	0.0238	0.1484	0.0914	0.0873	0.0278

12Pd: NSD result generated from file dl10.pdb; Summary of the NSD (in Å):

basis	Dip	dip	B2g	B1g	Eu(x)	Eu(y)	A1g	A2g
min.	0.3286	0.0571	-0.0054	-0.0001	0.0411	-0.0412	-0.3234	-0.0002
ext.	0.3680	0.0467	-0.0054	-0.0001	0.0413	-0.0413	-0.3239	-0.0002
			0.0016	-0.0001	0.0160	-0.0161	-0.1639	-0.0000
comp.	0.4414	0.0000	0.0090	0.0004	0.0618	0.0621	0.4325	0.0004
**basis**	**Doop**	**doop**	**B2u**	**B1u**	**A2u**	**Eg(x)**	**Eg(y)**	**A1u**
min.	2.8373	0.0233	-2.8313	-0.0002	0.0000	0.1299	0.1303	-0.0090
ext.	2.8413	0.0045	-2.8286	-0.0002	0.0000	0.1287	0.1291	-0.0090
			0.1434	0.0001	-0.0001	-0.0315	-0.0314	0.0057
comp.	2.8414	0.0000	2.8351	0.0003	0.0003	0.1341	0.1344	0.0107

14: NSD result generated from file mr03.pdb; Summary of the NSD (in Å):

**basis**	**Dip**	**dip**	**B2g**	**B1g**	**Eu(x)**	**Eu(y)**	**A1g**	**A2g**
min.	0.2308	0.1180	0.0356	0.0474	-0.0088	-0.0219	-0.2218	0.0019
ext.	0.3783	0.1002	0.0357	0.0474	-0.0089	-0.0223	-0.2227	0.0020
			0.0017	-0.0032	-0.0056	-0.0320	-0.2978	0.0096
comp.	0.6868	0.0000	0.0375	0.0569	0.0324	0.0571	0.6802	0.0103
**basis**	**Doop**	**doop**	**B2u**	**B1u**	**A2u**	**Eg(x)**	**Eg(y)**	**A1u**
min.	3.1556	0.0939	-3.1541	-0.0282	-0.0585	-0.0253	0.0664	0.0141
ext.	3.2106	0.0144	-3.1428	-0.0282	-0.0586	-0.0265	0.0671	0.0141
			0.5903	0.0003	-0.0028	-0.0306	0.0180	0.0003
comp.	3.2116	0.0000	3.2099	0.0289	0.0587	0.0405	0.0713	0.0141

16: NSD result generated from file 18jux_mr02.pdb; Summary of the NSD (in Å):

**basis**	**Dip**	**dip**	**B2g**	**B1g**	**Eu(x)**	**Eu(y)**	**A1g**	**A2g**
min.	0.7936	0.1865	0.0660	0.1773	0.0041	0.0542	-0.7682	-0.0304
ext.	0.9517	0.1508	0.0660	0.1775	0.0043	0.0550	-0.7698	-0.0307
			0.0008	0.0101	0.0173	0.0661	-0.5199	-0.0277
comp.	1.2678	0.0000	0.0663	0.1976	0.0223	0.1066	1.2450	0.0421
**basis**	**Doop**	**doop**	**B2u**	**B1u**	**A2u**	**Eg(x)**	**Eg(y)**	**A1u**
min.	4.0380	0.1115	4.0346	-0.0871	0.1098	-0.0044	0.0916	-0.0005
ext.	4.0960	0.0126	4.0214	-0.0871	0.1089	-0.0048	0.0912	-0.0005
			-0.6862	-0.0094	-0.0203	-0.0095	-0.0119	-0.0026
comp.	4.0966	0.0000	4.0930	0.0877	0.1117	0.0137	0.0953	0.0026

23: NSD result generated from file 18jux_mr01.pdb; Summary of the NSD (in Å):

**basis**	**Dip**	**dip**	**B2g**	**B1g**	**Eu(x)**	**Eu(y)**	**A1g**	**A2g**
min.	0.5618	0.1608	0.0366	-0.1758	-0.0582	0.0045	-0.5276	-0.0414
ext.	0.7239	0.1299	0.0366	-0.1763	-0.0585	0.0050	-0.5289	-0.0426
			0.0014	-0.0249	-0.0279	0.0495	-0.4336	-0.1285
comp.	1.0246	0.0000	0.0376	0.1810	0.0955	0.0706	0.9914	0.1367
**basis**	**Doop**	**doop**	**B2u**	**B1u**	**A2u**	**Eg(x)**	**Eg(y)**	**A1u**
min.	3.6804	0.1113	3.6584	-0.3772	0.0302	0.1232	0.0570	0.0054
ext.	3.7438	0.0123	3.6452	-0.3772	0.0315	0.1222	0.0582	0.0054
			-0.6842	-0.0045	0.0294	-0.0282	0.0334	-0.0022
comp.	3.7445	0.0000	3.7224	0.3773	0.0422	0.1274	0.0675	0.0058

26: NSD result generated from file 16jux_mr03_twin1_hklf4.pdb; Summary of the NSD (in Å):

**basis**	**Dip**	**dip**	**B2g**	**B1g**	**Eu(x)**	**Eu(y)**	**A1g**	**A2g**
min.	0.3740	0.0736	-0.0002	-0.0005	0.0002	0.0001	-0.3740	0.0030
ext.	0.4468	0.0523	-0.0002	-0.0005	0.0002	0.0001	-0.3745	0.0047
			0.0003	-0.0002	0.0003	-0.0001	-0.1617	0.1832
comp.	0.5289	0.0001	0.0008	0.0008	0.0011	0.0011	0.4953	0.1856
**basis**	**Doop**	**doop**	**B2u**	**B1u**	**A2u**	**Eg(x)**	**Eg(y)**	**A1u**
min.	3.1577	0.0172	3.0767	0.7107	0.0002	-0.0000	-0.0000	-0.0005
ext.	3.1594	0.0035	3.0746	0.7107	0.0002	-0.0000	-0.0000	-0.0005
			-0.1050	-0.0132	-0.0009	0.0000	-0.0000	-0.0005
comp.	3.1595	0.0000	3.0785	0.7110	0.0009	0.0000	0.0000	0.0007

27: NSD result generated from file 16jux_mr05.pdb; Summary of the NSD (in Å):

**basis**	**Dip**	**dip**	**B2g**	**B1g**	**Eu(x)**	**Eu(y)**	**A1g**	**A2g**
min.	0.3604	0.1423	-0.0000	-0.0000	-0.0000	0.0000	-0.3597	0.0235
ext.	0.5280	0.1171	-0.0000	-0.0000	-0.0000	0.0000	-0.3609	0.0241
			0.0000	-0.0000	0.0000	-0.0000	-0.3799	0.0672
comp.	0.8394	0.0000	0.0000	0.0000	0.0000	0.0000	0.8363	0.0719
**basis**	**Doop**	**doop**	**B2u**	**B1u**	**A2u**	**Eg(x)**	**Eg(y)**	**A1u**
min.	3.3939	0.1097	3.3875	0.2073	0.0000	-0.0000	0.0000	0.0000
ext.	3.4612	0.0103	3.3745	0.2073	0.0000	-0.0000	0.0000	0.0000
			-0.6793	0.0041	-0.0000	-0.0000	0.0000	-0.0000
comp.	3.4617	0.0000	3.4554	0.2077	0.0000	0.0000	0.0000	0.0000

29: NSD result generated from file 16jux_mr04_2.pdb; Summary of the NSD (in Å):

**basis**	**Dip**	**dip**	**B2g**	**B1g**	**Eu(x)**	**Eu(y)**	**A1g**	**A2g**
min.	0.3982	0.0824	-0.1300	0.2446	-0.0000	0.0000	-0.2800	0.0591
ext.	0.4799	0.0609	-0.1298	0.2462	-0.0000	0.0000	-0.2806	0.0605
			0.0254	0.0883	-0.0000	0.0000	-0.1976	0.1556
comp.	0.5871	0.0000	0.1335	0.2665	0.0000	0.0000	0.4767	0.1691
**basis**	**Doop**	**doop**	**B2u**	**B1u**	**A2u**	**Eg(x)**	**Eg(y)**	**A1u**
min.	3.0353	0.0483	2.9588	0.6175	0.2788	-0.0000	-0.0000	-0.0049
ext.	3.0479	0.0036	2.9535	0.6175	0.2768	-0.0000	-0.0000	-0.0049
			-0.2724	0.0203	-0.0454	-0.0000	-0.0000	-0.0051
comp.	3.0480	0.0000	2.9713	0.6180	0.2829	0.0000	0.0000	0.0071

5 Δh ANALYSIS

Table S5. distances between δcarbons and meso-carbon plane for TATBP. The flap-height Δh is defined as the arithmetic mean of the eight δ values. STEDV= standard devitation

	**4**	**12-Pd**	**26**	**29**	**14**	**27**	**2**	**9**	**23**	**16**
δ_1 / Å	2.047	2.311	2.279	2.571	2.62	2.732	2.696	2.904	3.016	3.054
δ_2 / Å	1.873	2.27	2.057	2.428	2.624	2.67	2.78	2.854	3.115	3.075
δ_3 / Å	1.733	1.702	2.279	2.017	2.406	2.732	2.696	3.045	2.945	2.962
δ_4 / Å	1.644	1.737	2.057	1.856	2.402	2.67	2.78	3.043	3.009	2.977
δ_5 / Å	1.48	1.737	2.279	2.571	2.345	2.67	2.78	2.622	2.59	3.363
δ_6 / Å	1.732	1.702	2.057	2.428	2.362	2.732	2.696	2.581	2.715	3.373
δ_7 / Å	1.74	2.311	2.279	1.856	2.304	2.732	2.78	2.759	2.656	2.947
δ_8 / Å	1.92	2.27	2.057	2.017	2.26	2.67	2.696	2.762	2.8	2.97
Δh / Å	1.771	2.005	2.168	2.218	2.415	2.701	2.738	2.821	2.856	3.090
STDEV / Å	0.175	0.306	0.119	0.311	0.136	0.033	0.045	0.174	0.192	0.177

6 SPECTRAL APPENDIX (NMR, HRMS, UV/VIS, FLUO)

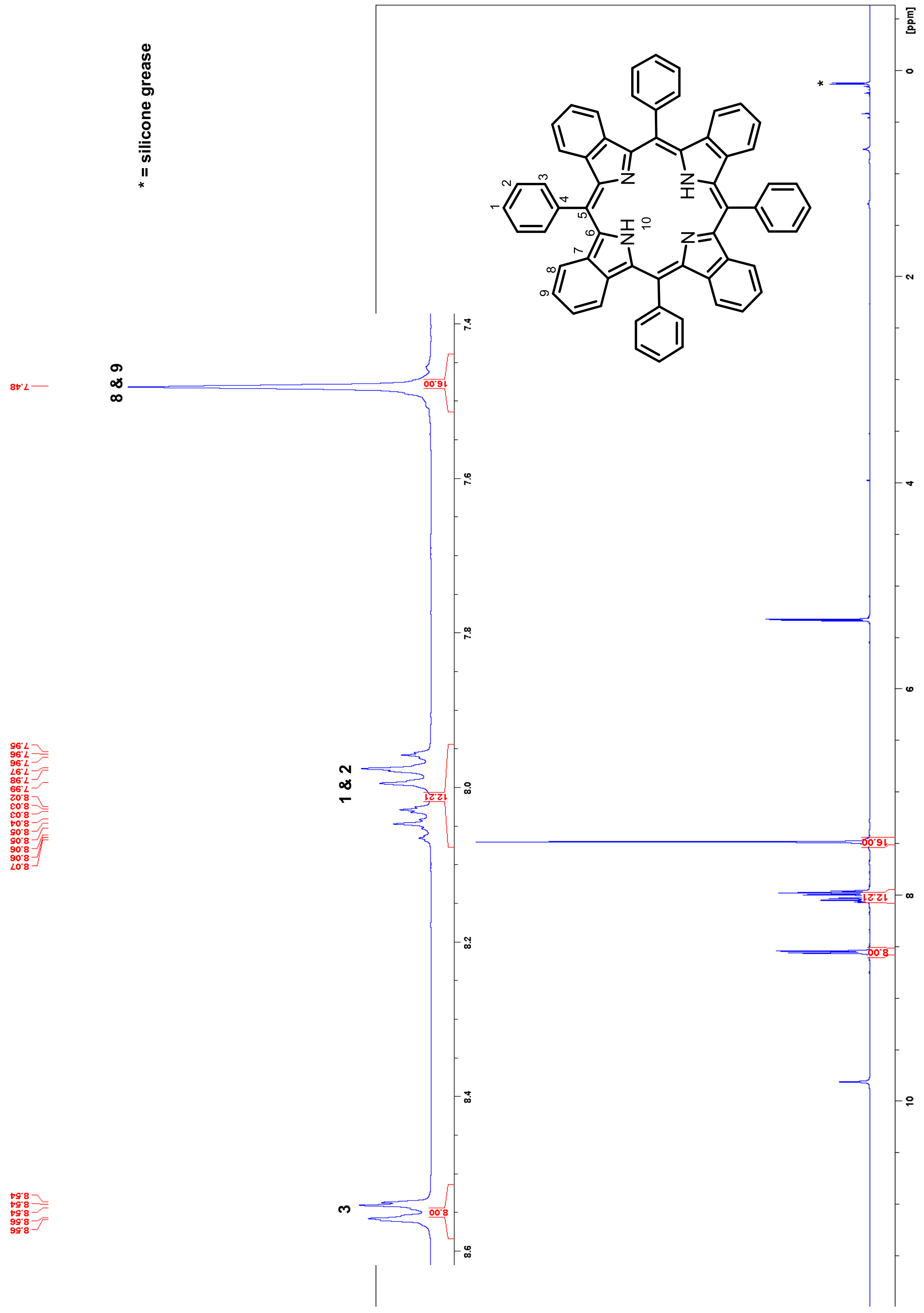

Figure S12. ^{1}H NMR (400 MHz, CD_2Cl_2/TFA-d_1, rt) of **2**.

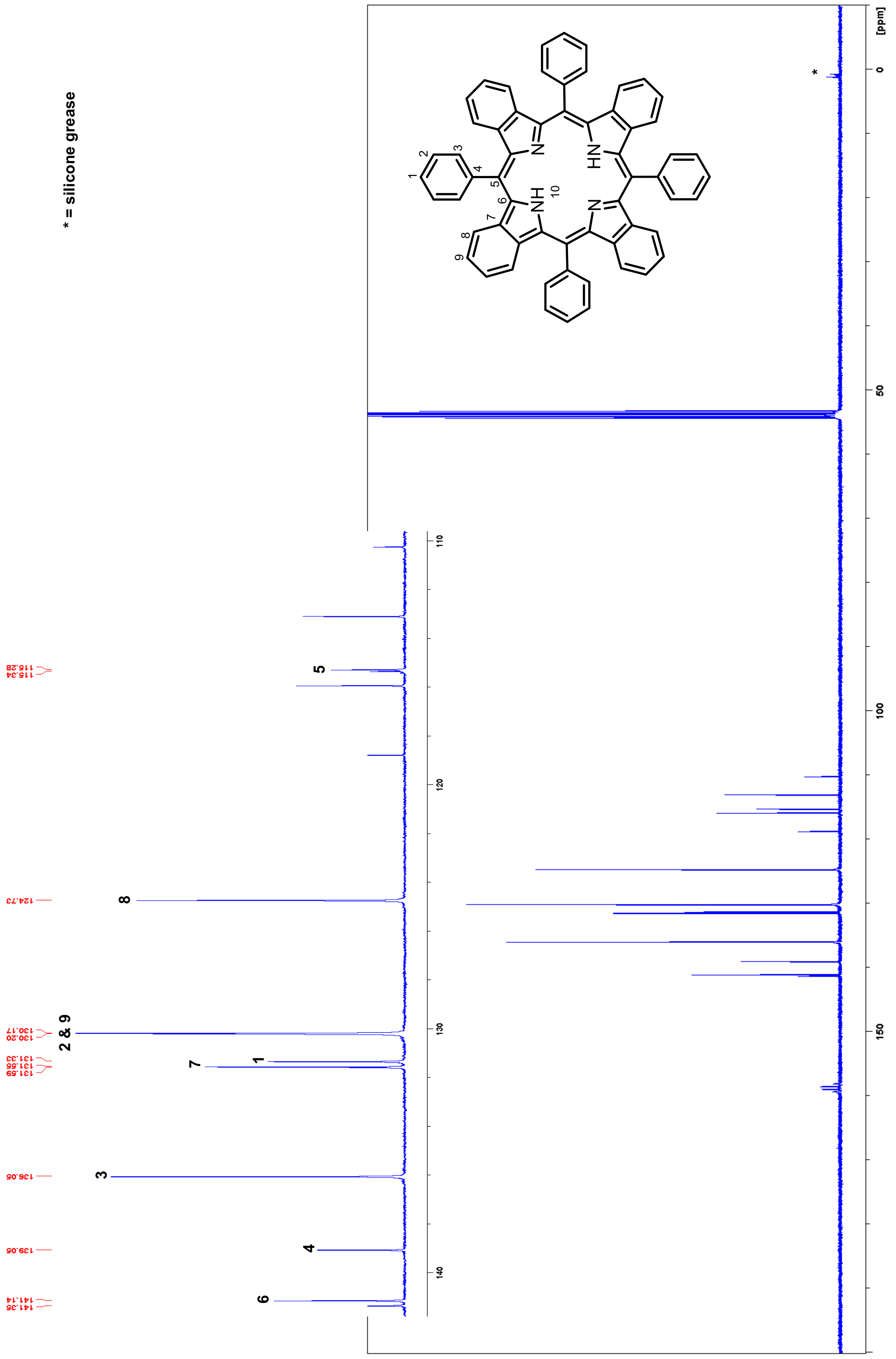

Figure S13. ^{13}C NMR (100 MHz, CD_2Cl_2/TFA-d_1, rt) of **2**.

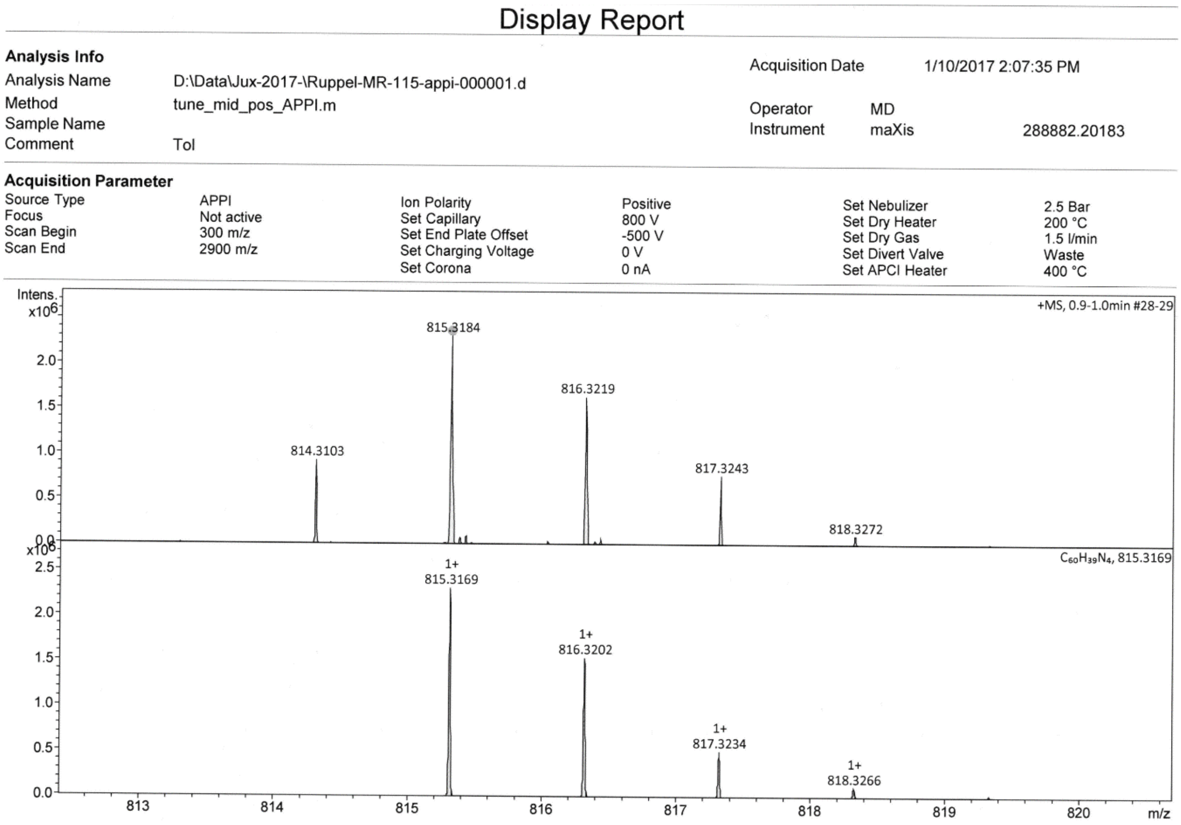

Figure S14. HRMS (APPI, toluene) of **2**.

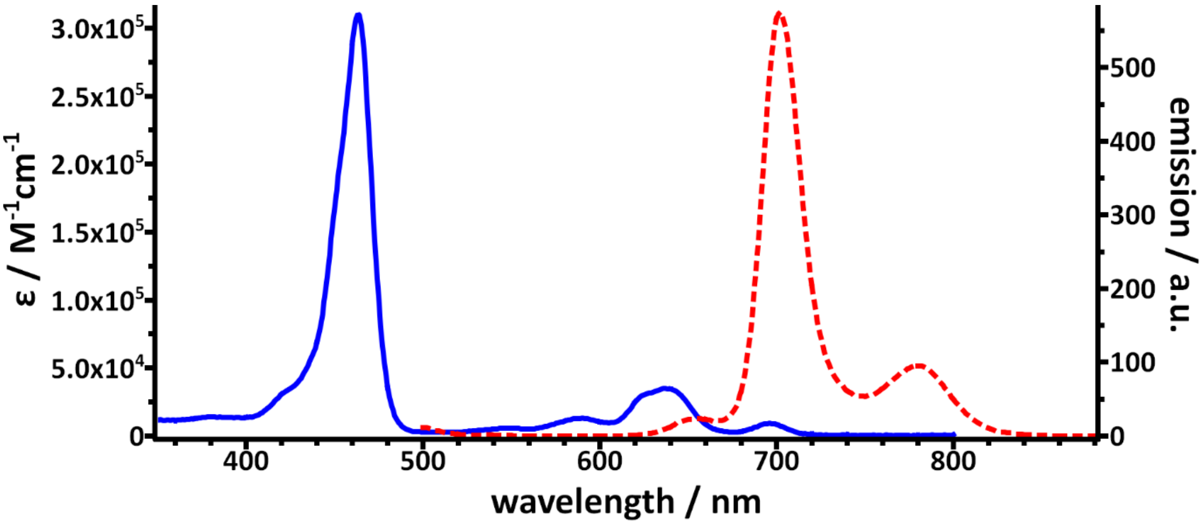

Figure S15. absorption (blue line) and emission spectrum of **2** (dashed red line; excitation at 463 nm) measured in CH_2Cl_2 + 1% NEt_3 at rt.

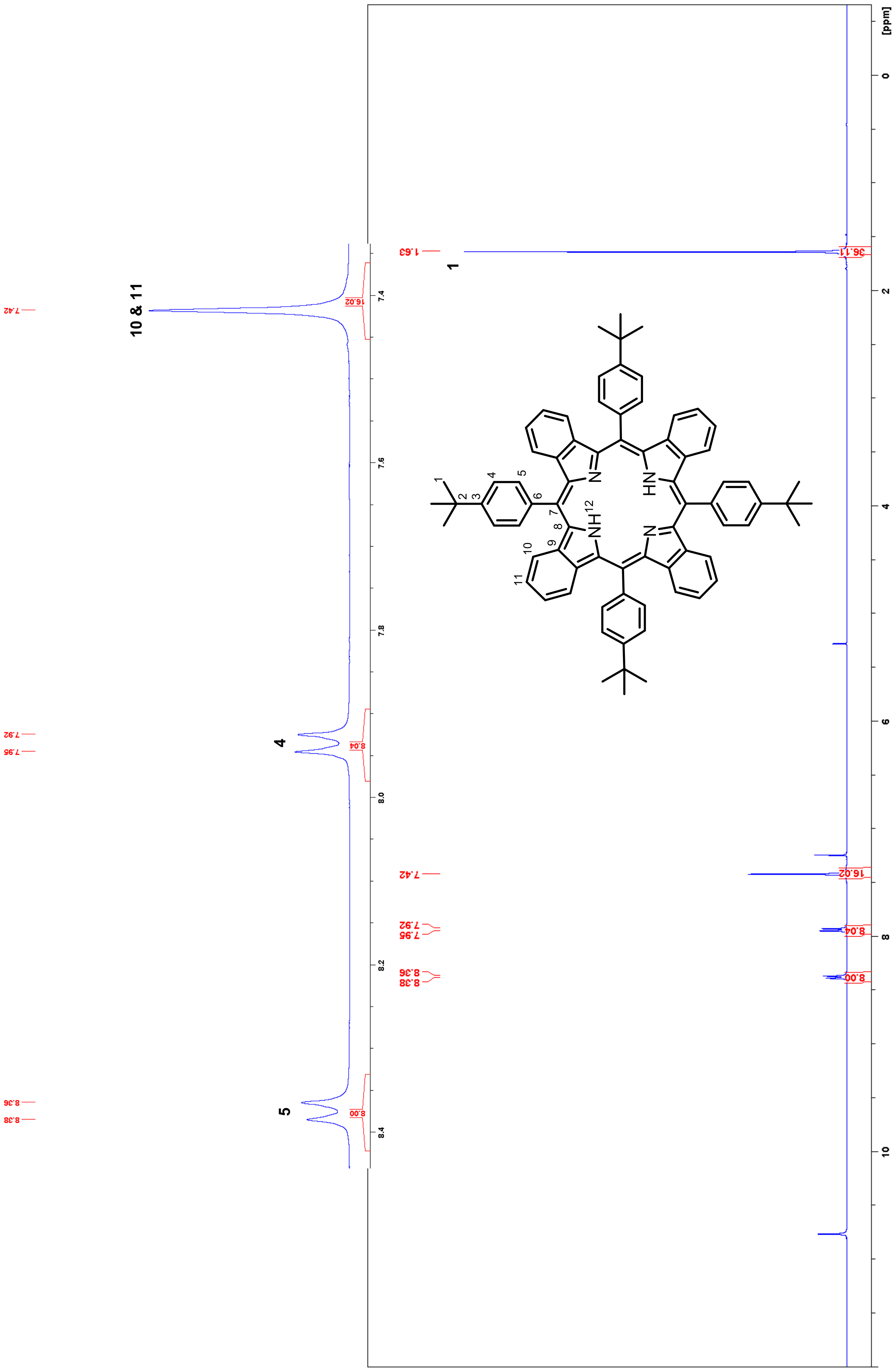

Figure S16. ^{1}H NMR (400 MHz, $CDCl_3$/TFA-d_1, rt) of **3**.

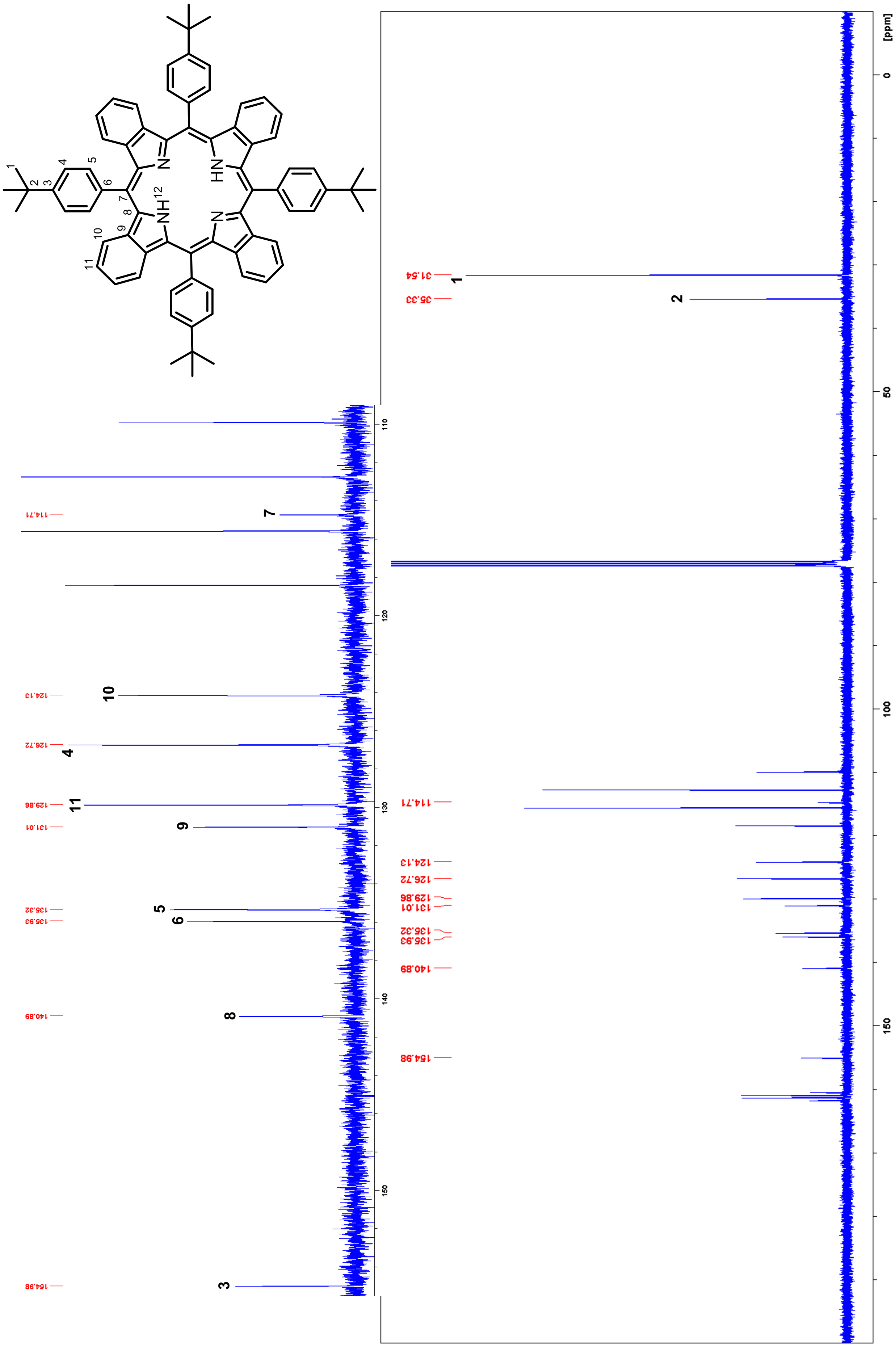

Figure S17. ^{13}C NMR (100 MHz, $CDCl_3$/TFA-d_1, rt) of **3**.

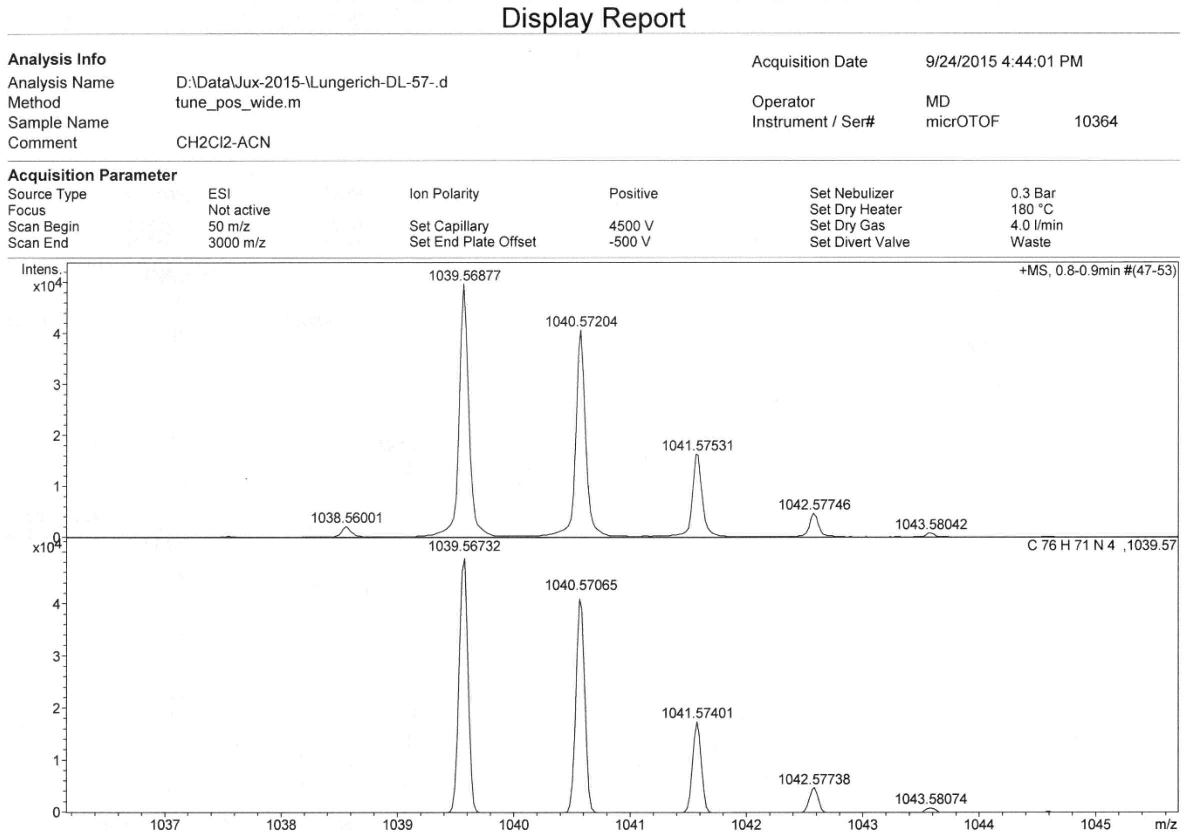

Figure S18. HRMS (ESI, CH_2Cl_2/MeCN) of **3**.

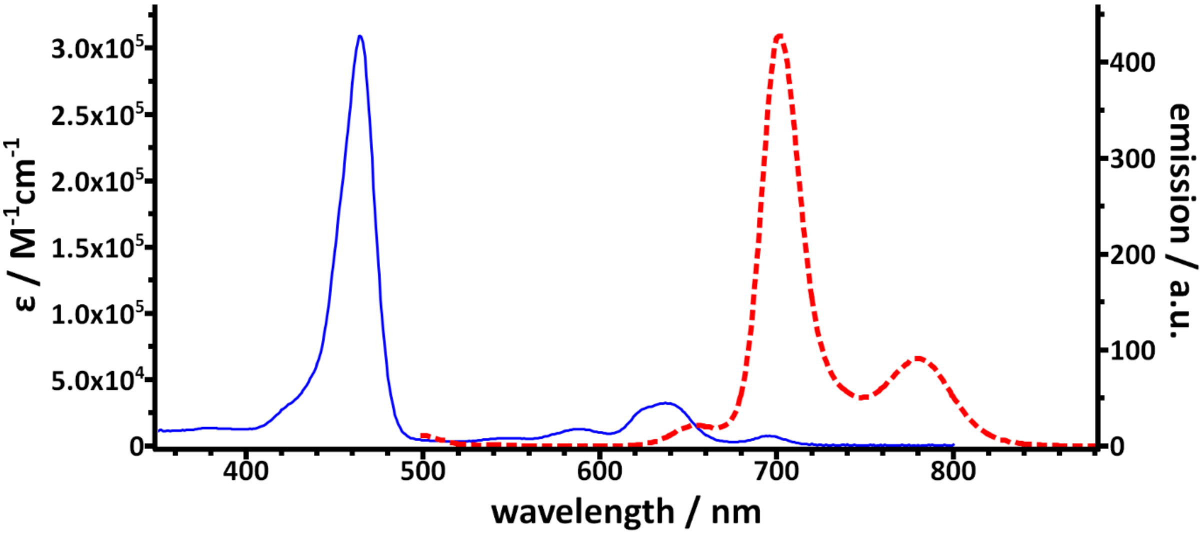

Figure S19. absorption (blue line) and emission spectrum of **3** (dashed red line; excitation at 464 nm) measured in CH_2Cl_2 + 1% NEt_3 at rt.

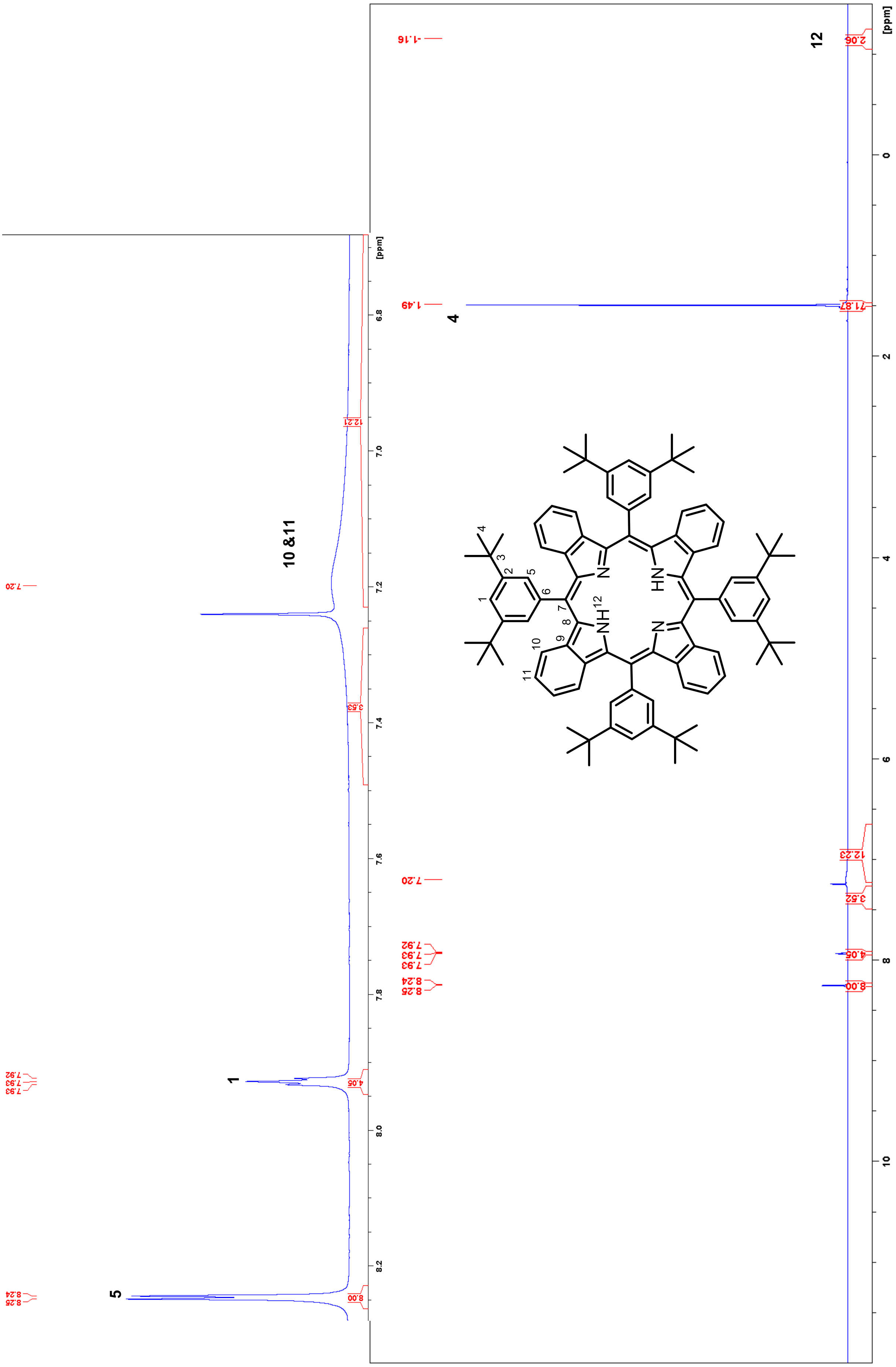

Figure S20. ^{1}H NMR (400 MHz, $CDCl_3$, rt) of **4**.

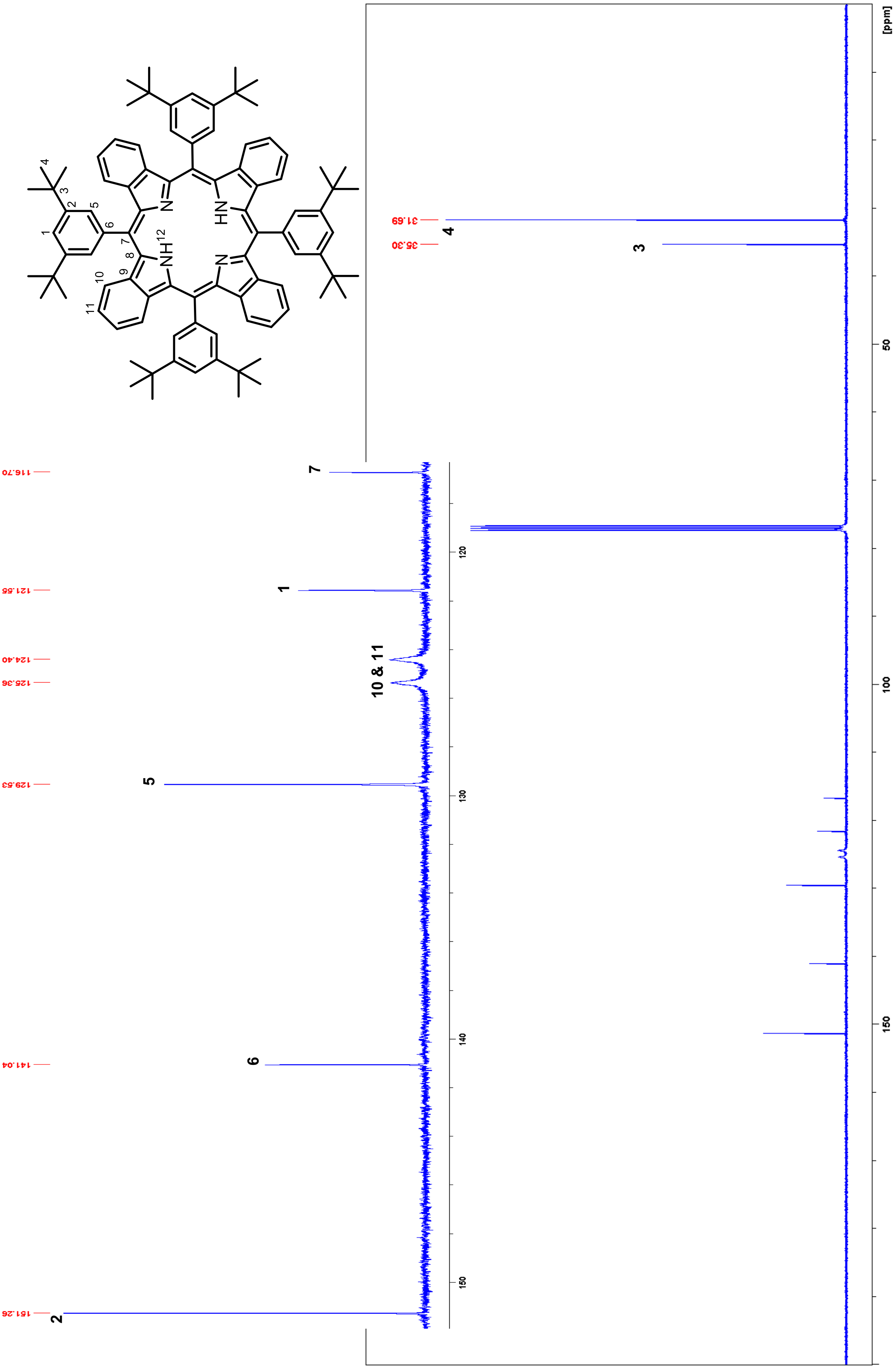

Figure S21. ^{13}C NMR (100 MHz, $CDCl_3$, rt) of **4**.

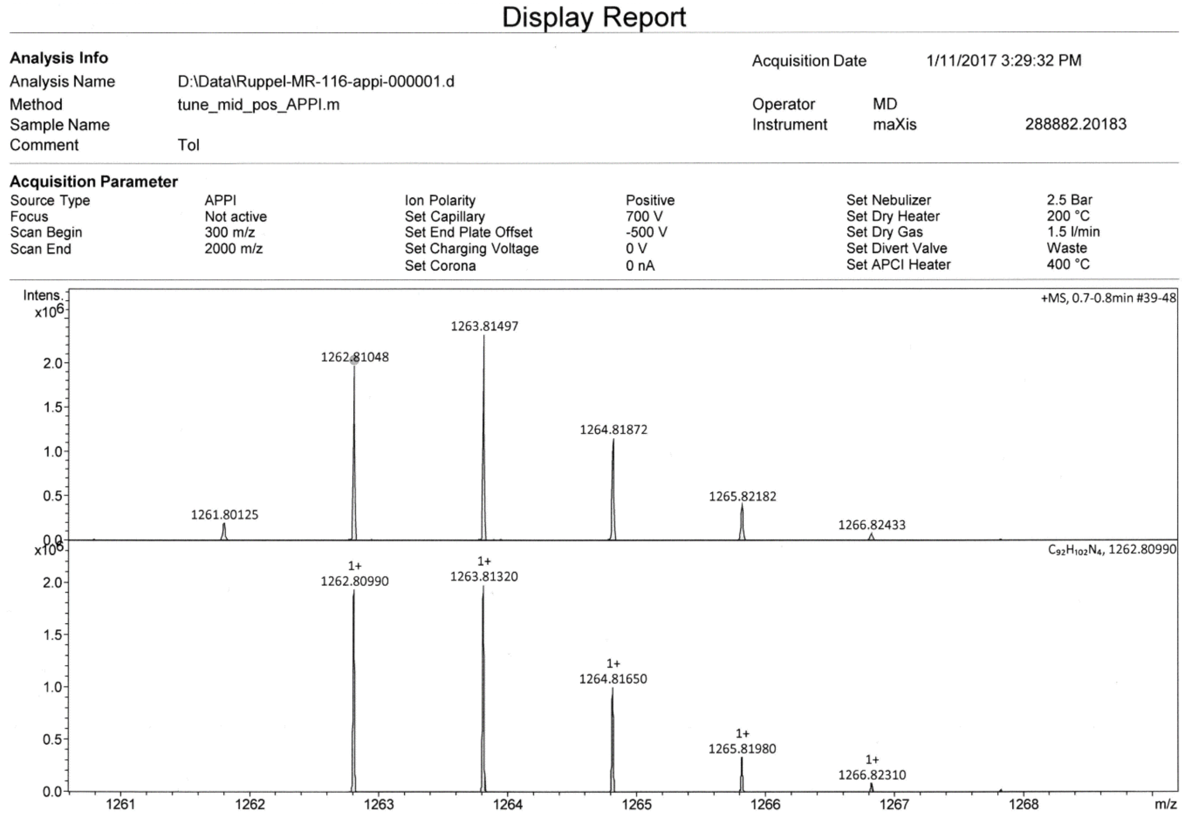

Figure S22. HRMS (APPI, toluene) of **4**.

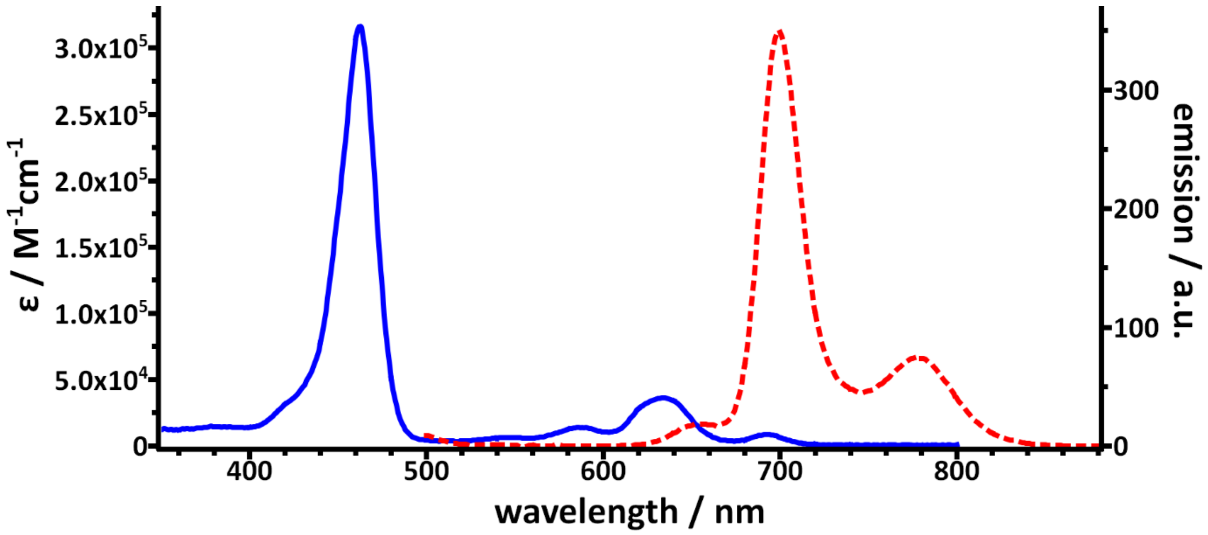

Figure S23. absorption (blue line) and emission spectrum of **4** (dashed red line; excitation at 462 nm) measured in CH_2Cl_2 + 1% NEt_3 at rt.

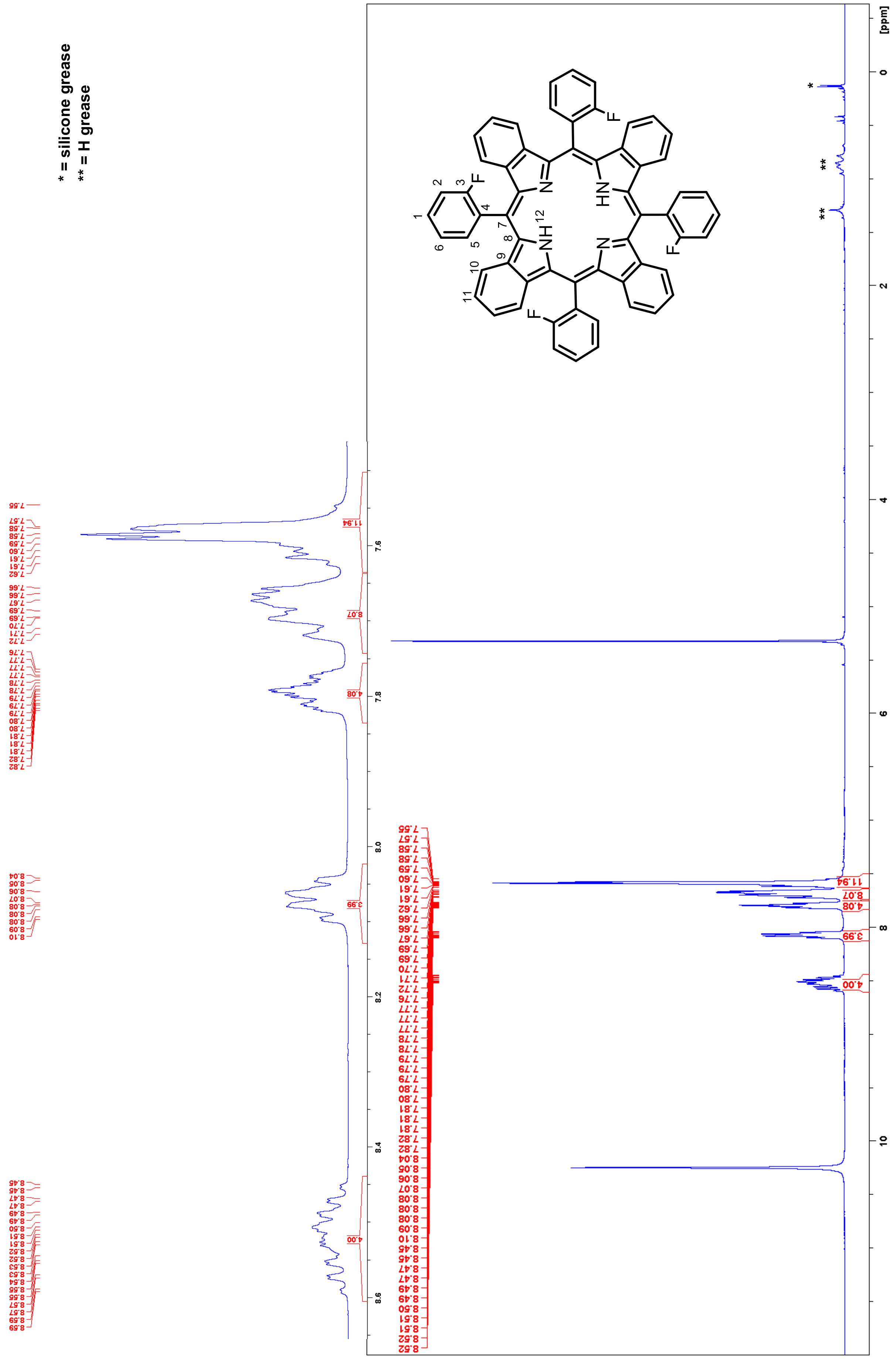

Figure S24. ^{1}H NMR (400 MHz, CD_2Cl_2/TFA-d_1, rt) of **5**.

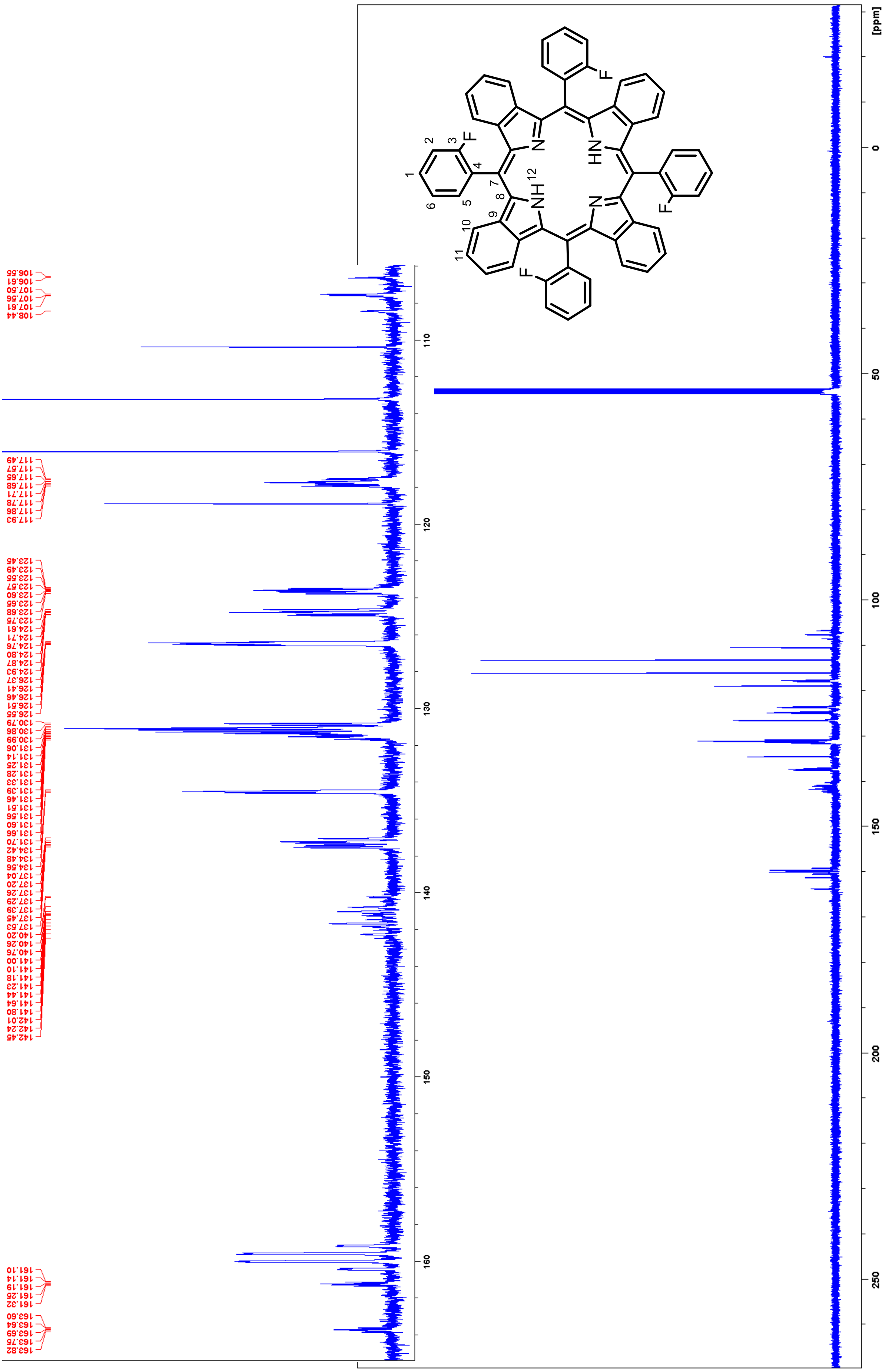

Figure S25. ^{13}C NMR (100 MHz, CD_2Cl_2/TFA-d_1, rt) of **5**.

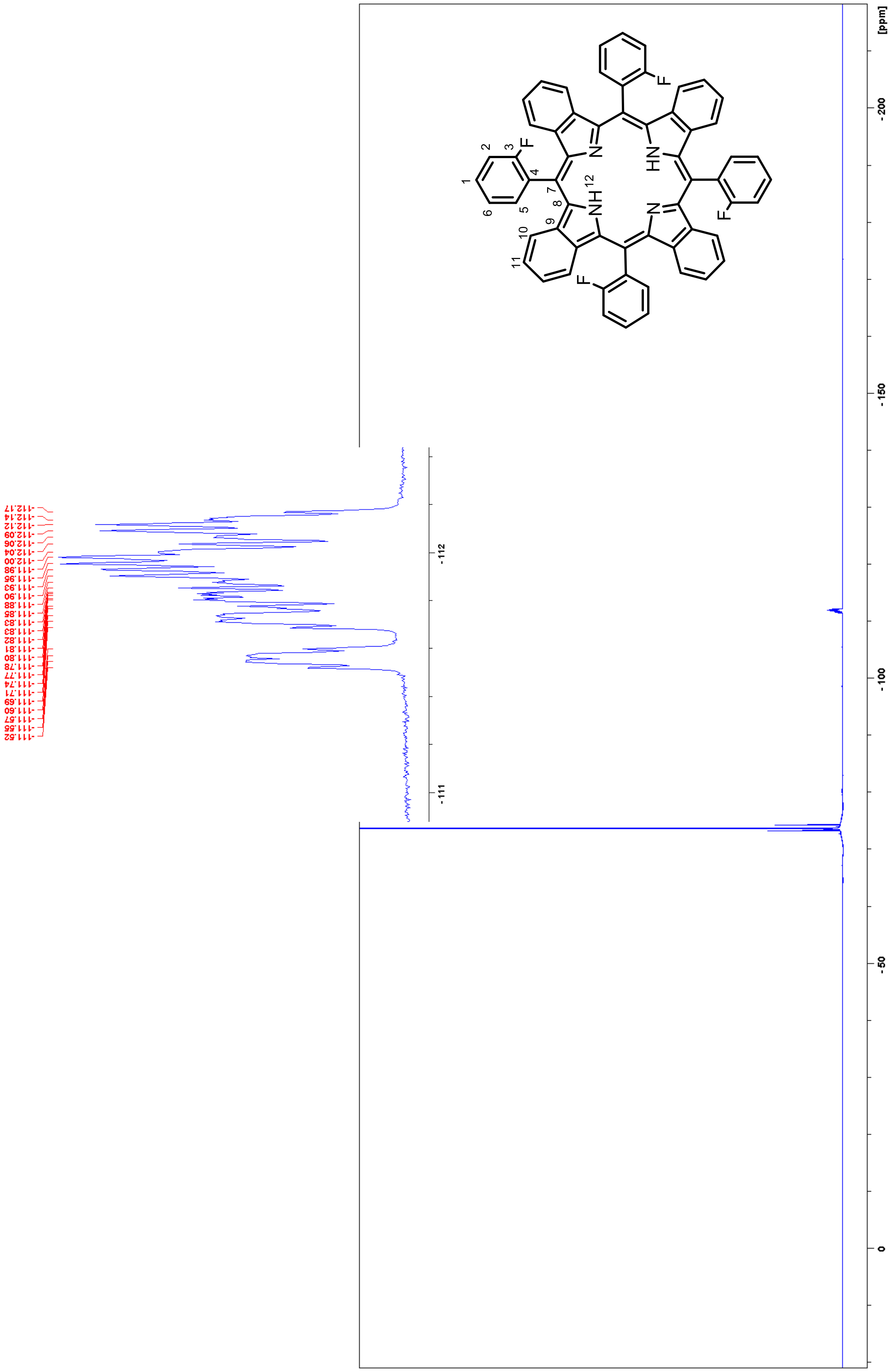

Figure S26. ^{19}F NMR (282 MHz, CD_2Cl_2/TFA-d_1, rt) of **5**.

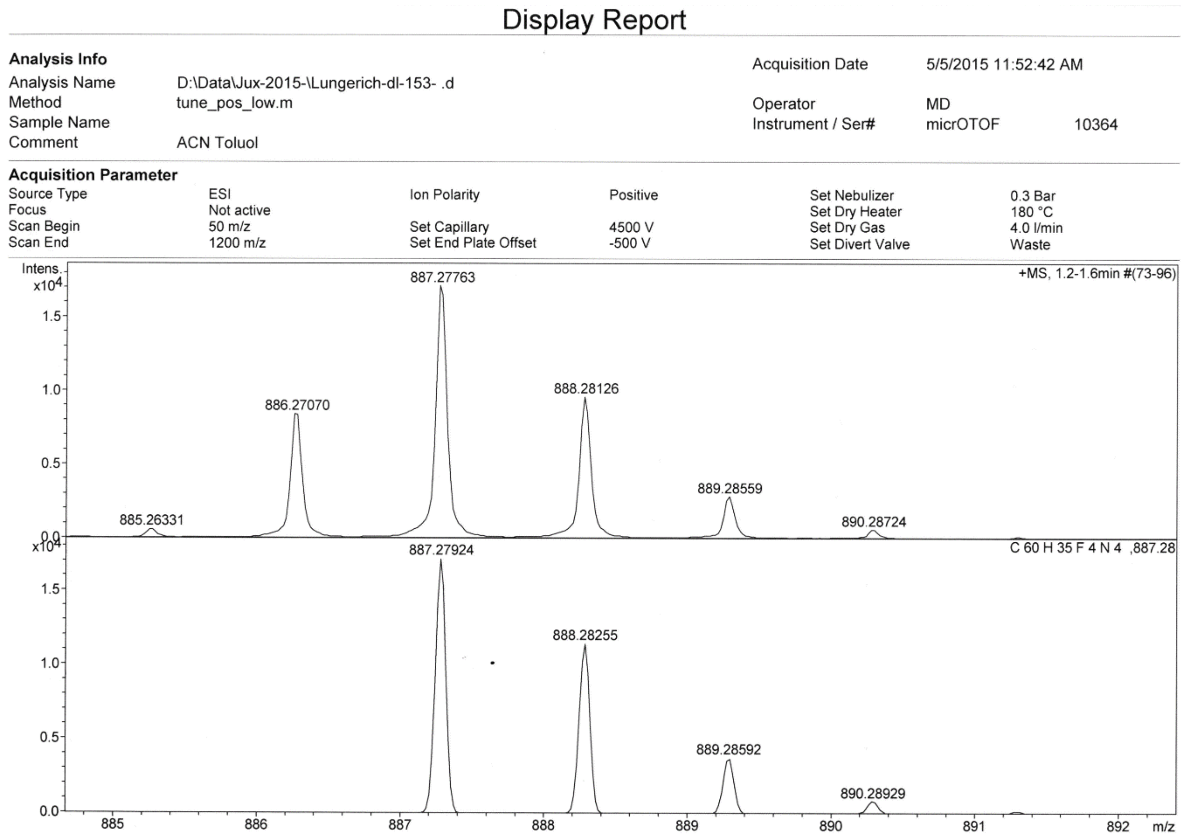

Figure S27. HRMS (ESI, MeCN/toluene) of **5**.

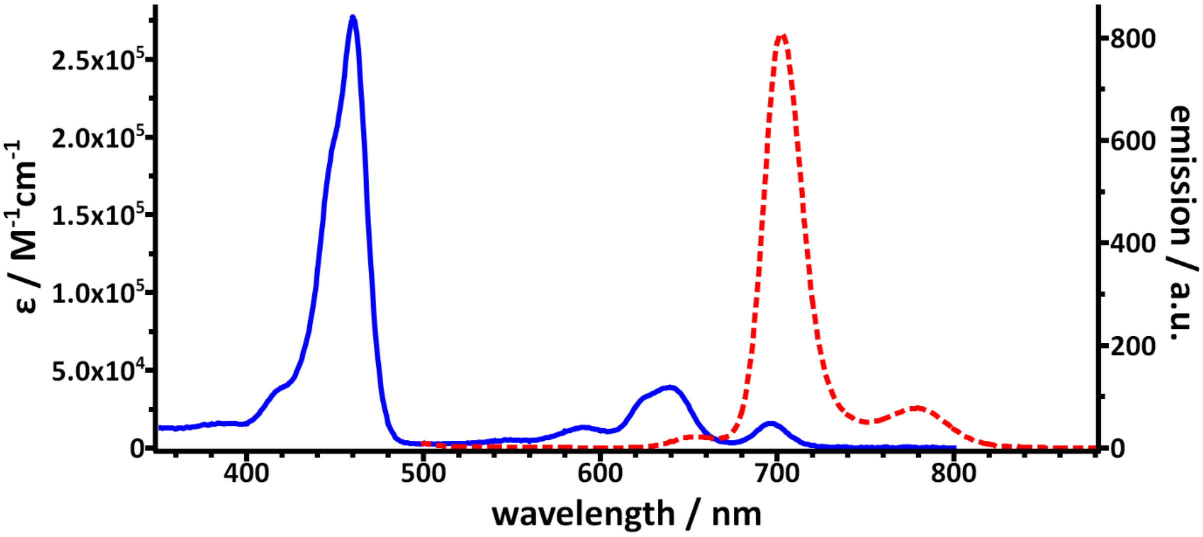

Figure S28. absorption (blue line) and emission spectrum of **5** (dashed red line; excitation at 460 nm) measured in CH_2Cl_2 + 1% NEt_3 at rt.

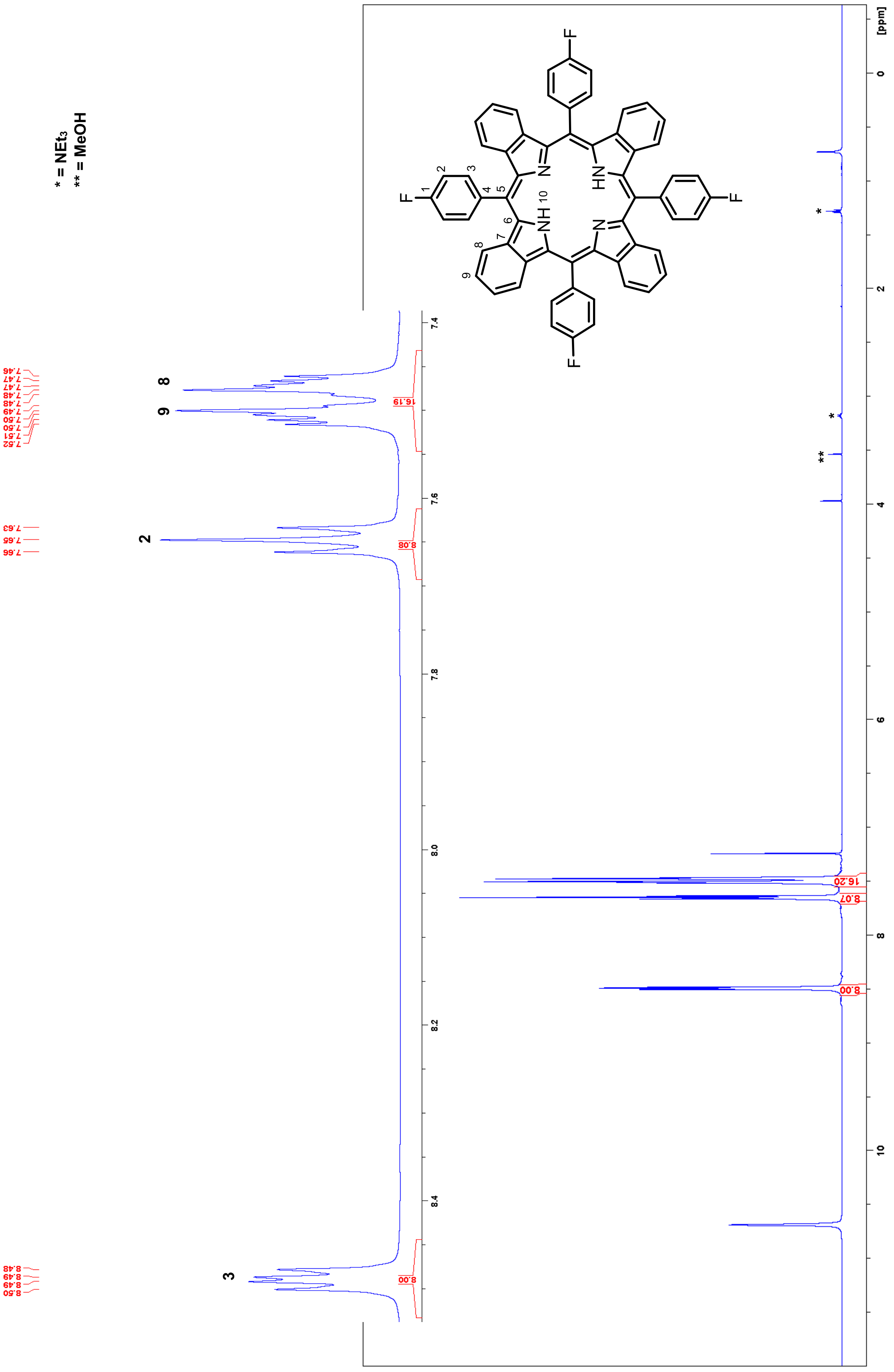

Figure S29. ^{1}H NMR (600 MHz, $CDCl_3$/TFA-d_1, rt) of **6**.

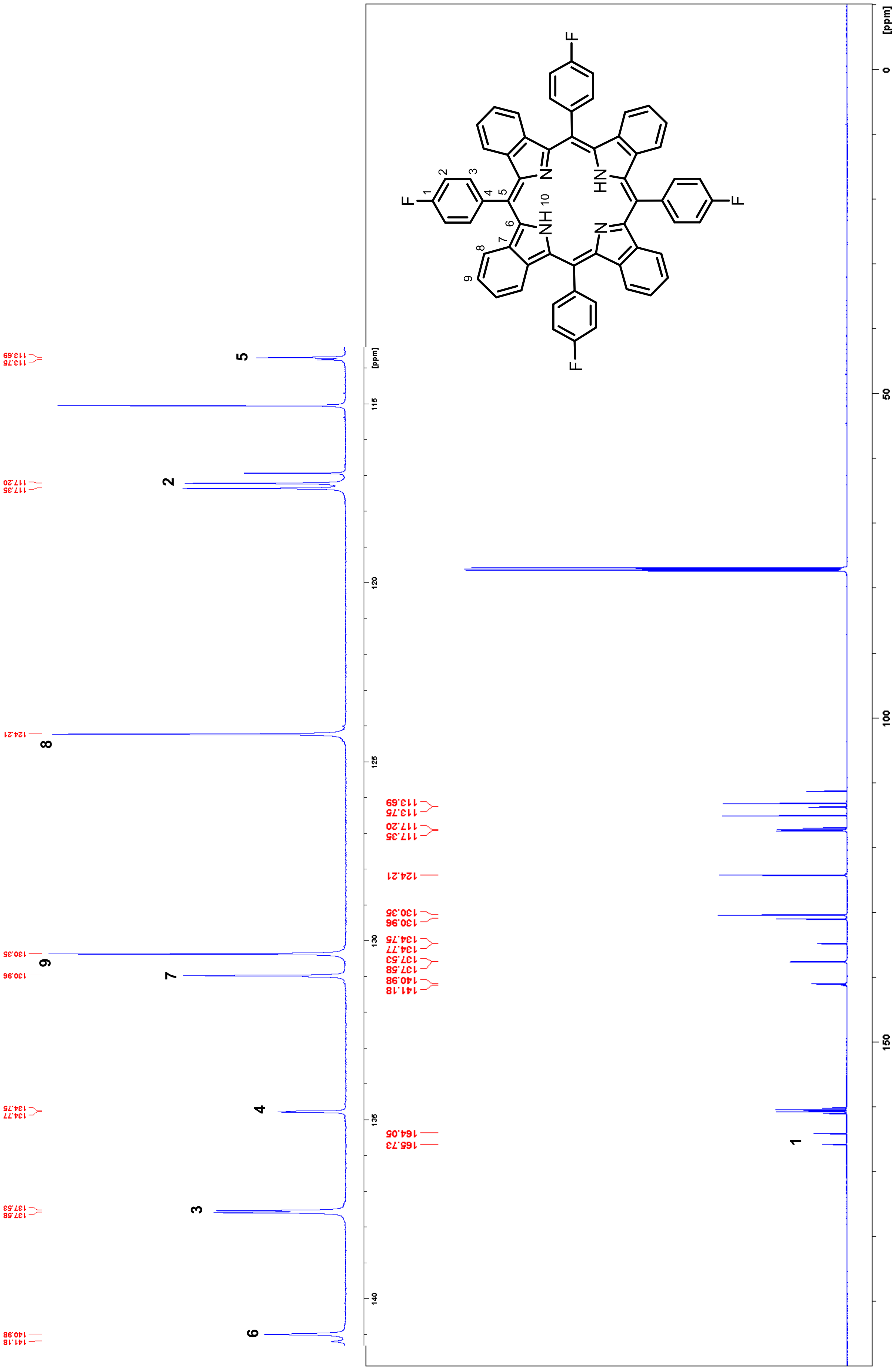

Figure S30. ^{13}C NMR (150 MHz, $CDCl_3$/TFA-d_1, rt) of **6**.

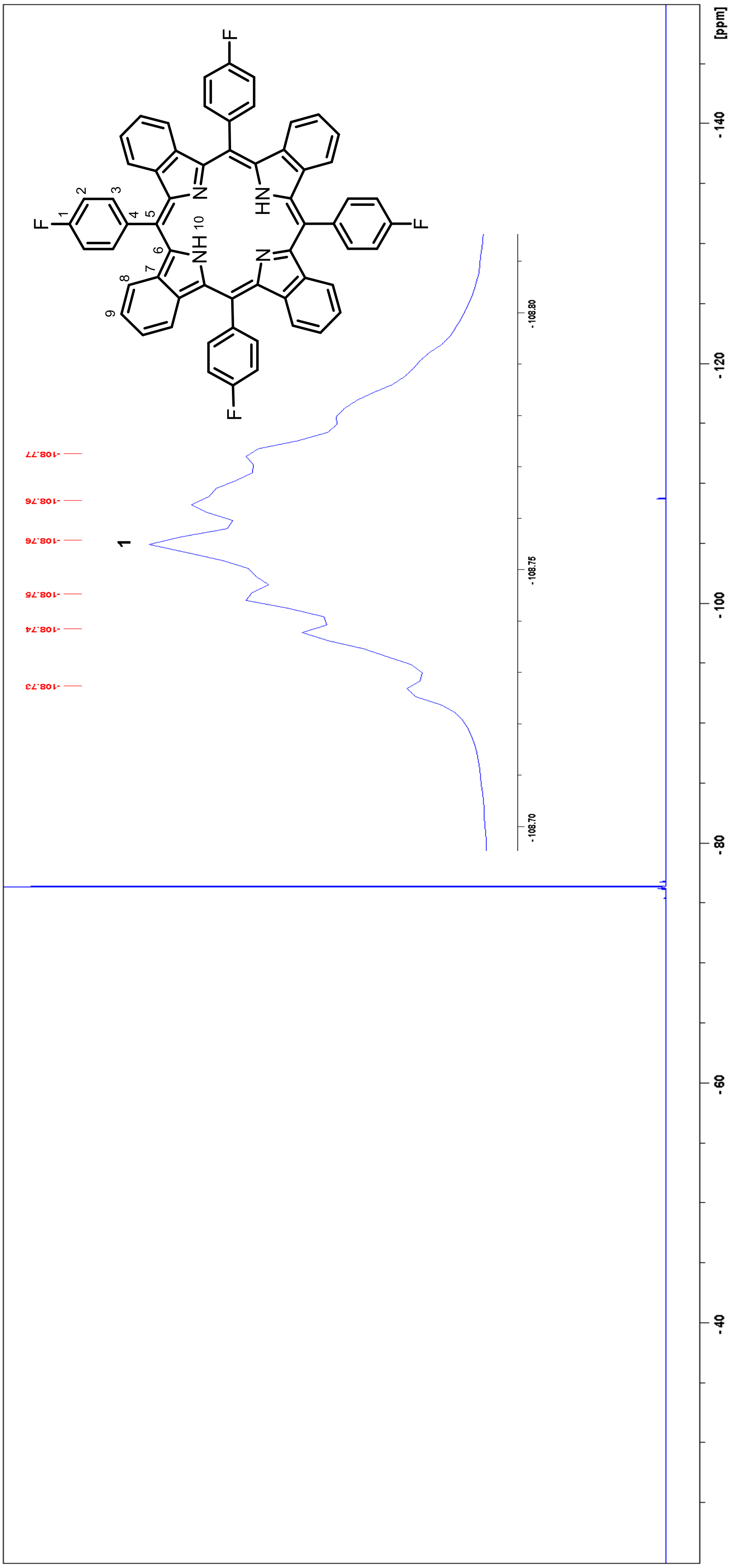

Figure S31. ^{19}F NMR (470 MHz, $CDCl_3$/TFA-d_1, rt) of **6**.

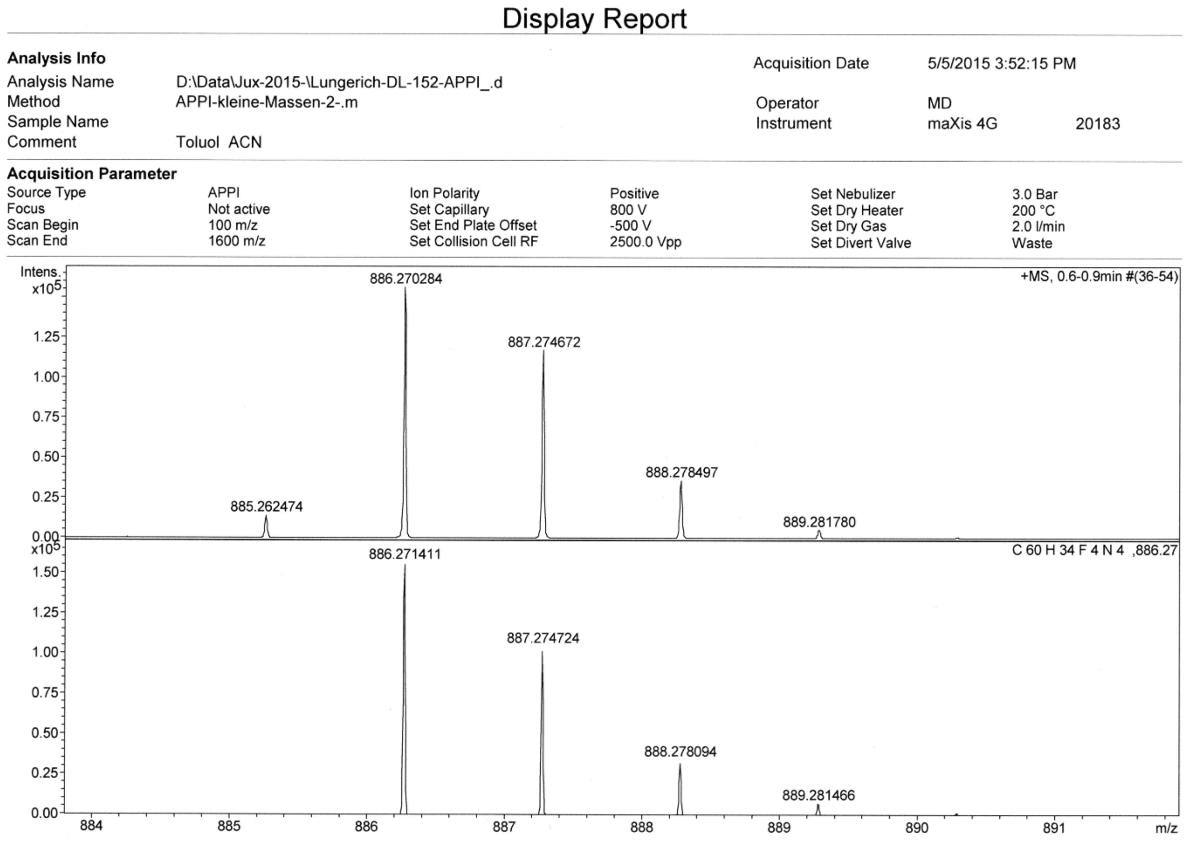

Figure S32. HRMS (APPI, MeCN/toluene) of **6**.

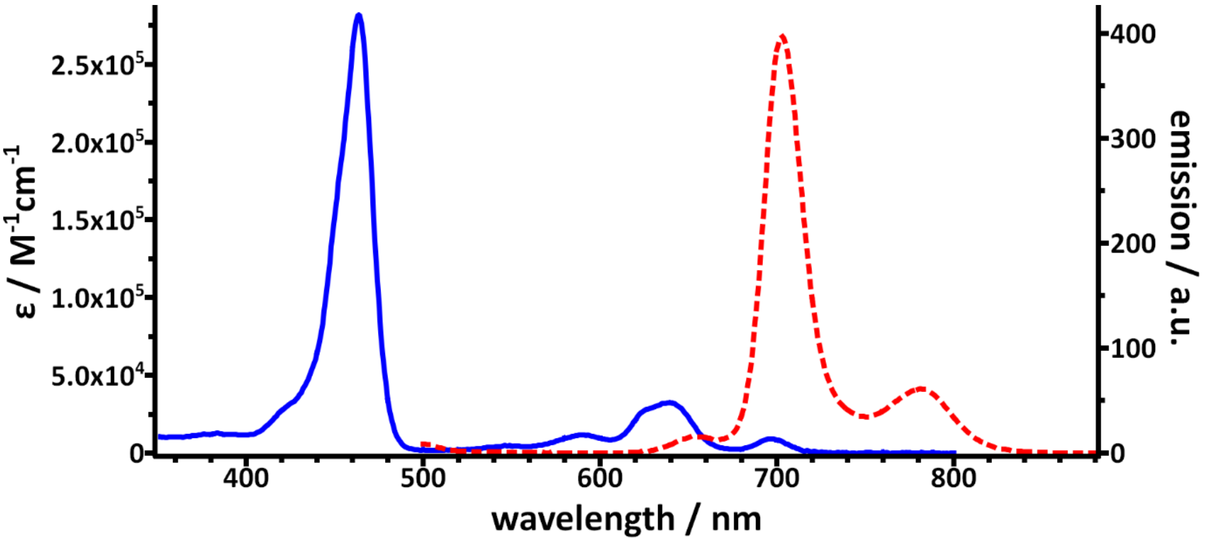

Figure S33. absorption (blue line) and emission spectrum of **6** (dashed red line; excitation at 463 nm) measured in CH_2Cl_2 + 1% NEt_3 at rt.

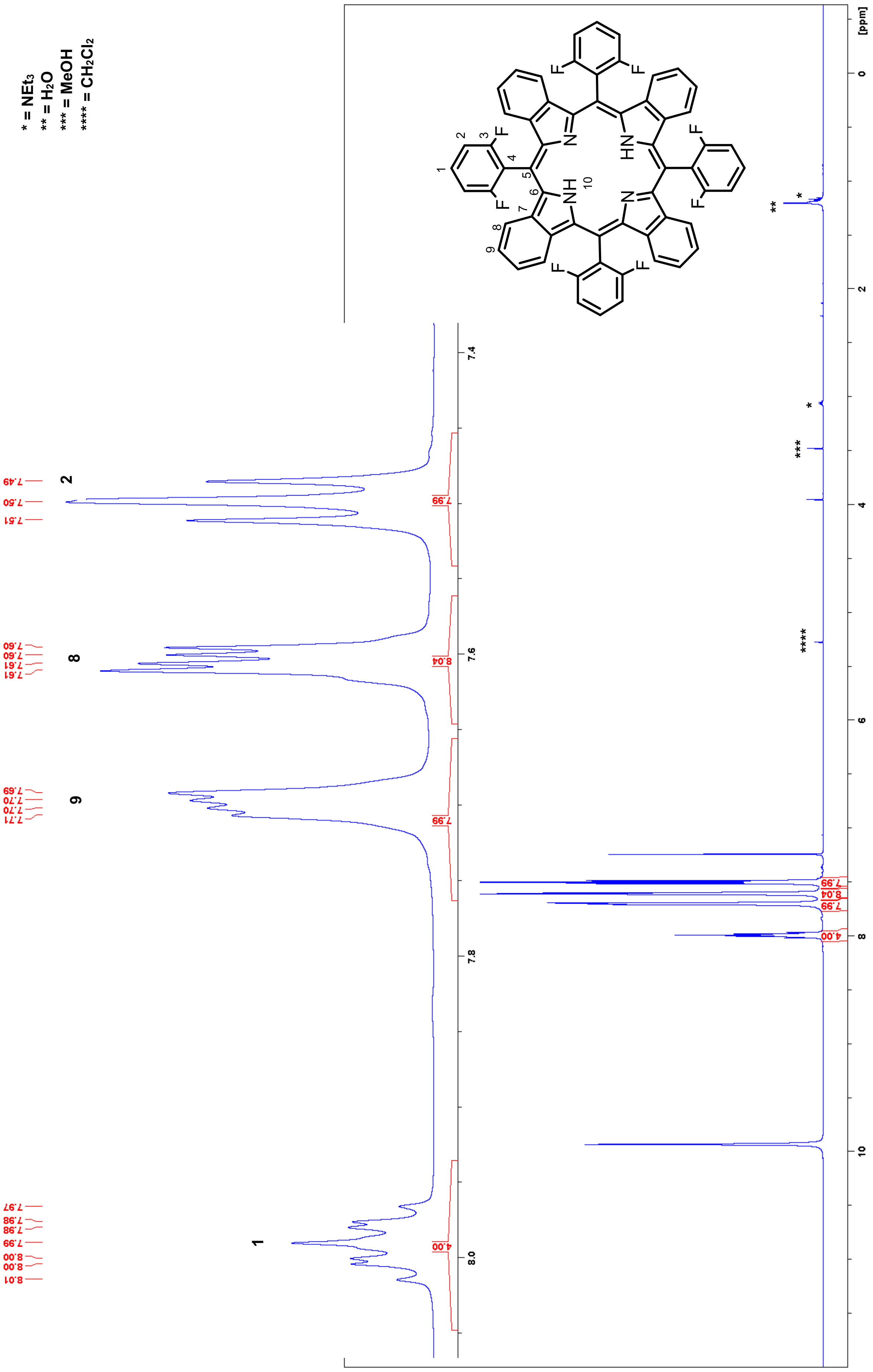

Figure S34. ^{1}H NMR (600 MHz, $CDCl_3$/TFA-d_1, rt) of **7**.

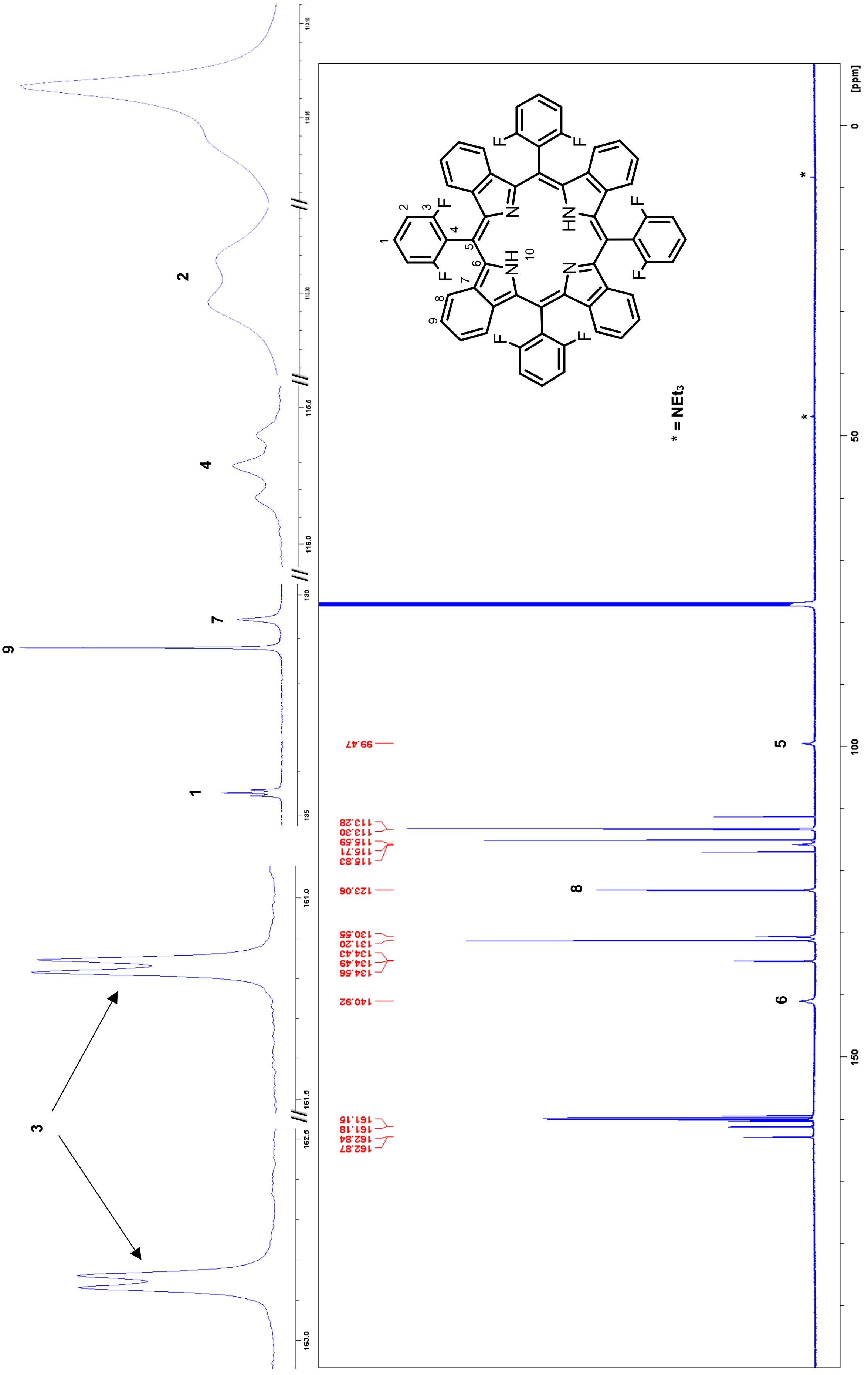

Figure S35. ^{13}C NMR (150 MHz, $CDCl_3$/TFA-d_1, rt) of **7**.

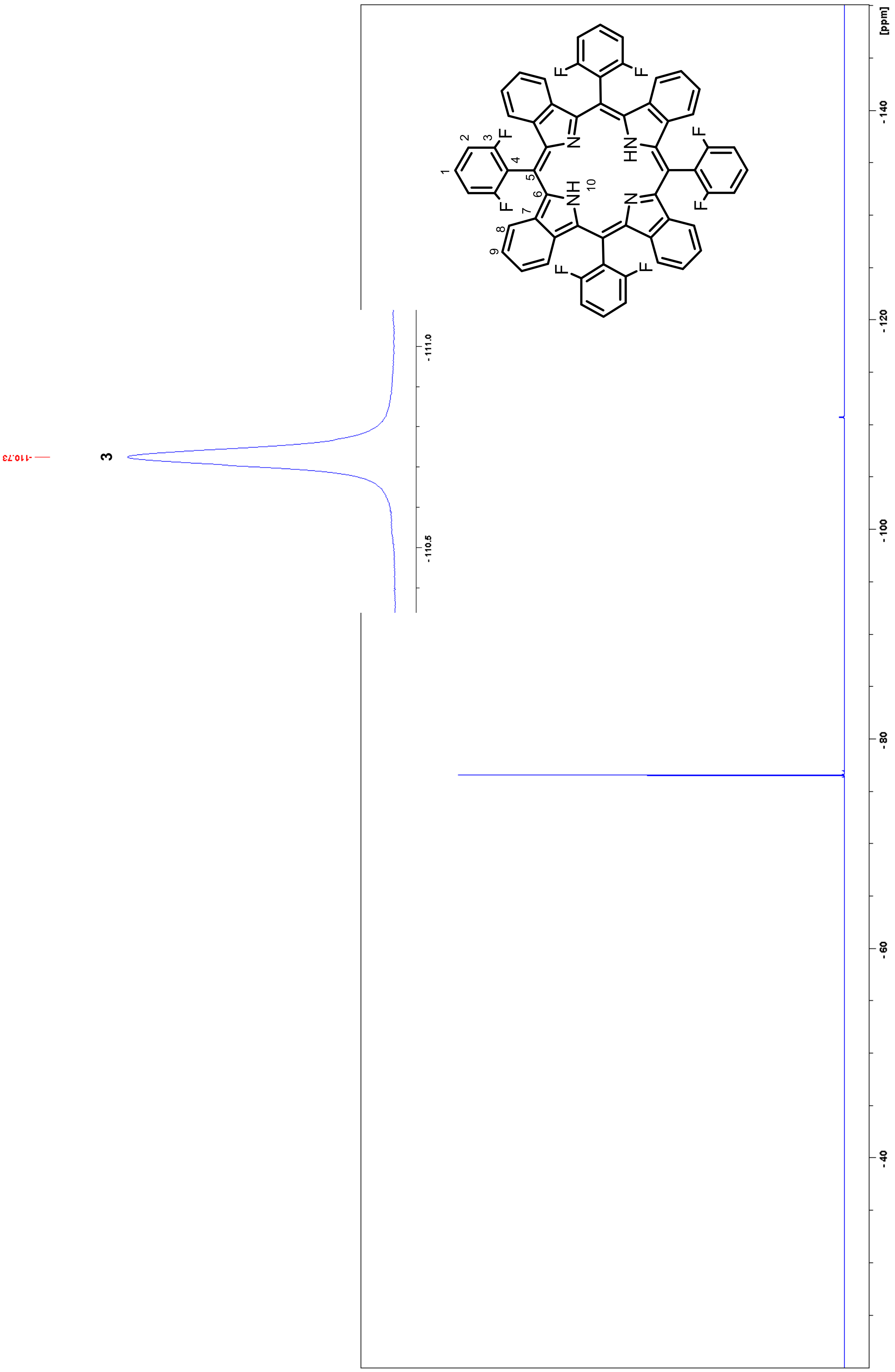

Figure S36. ^{19}F NMR (470 MHz, $CDCl_3$/TFA-d_1, rt) of **7**.

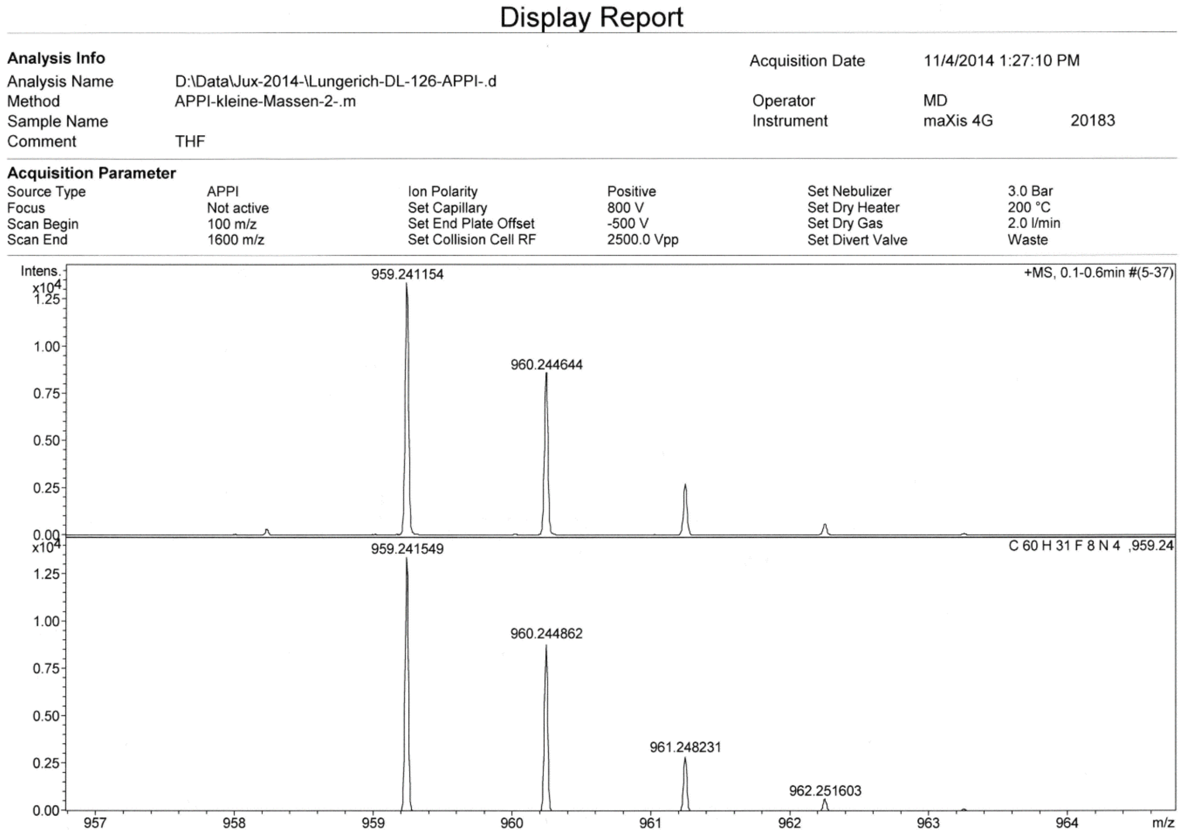

Figure S37. HRMS (APPI, THF) of **7**.

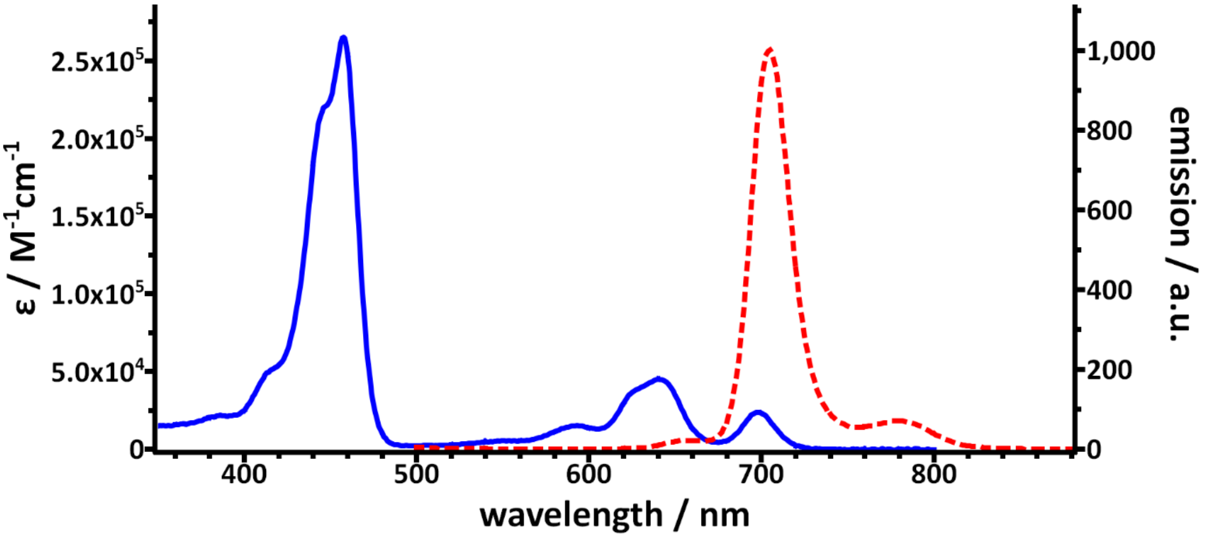

Figure S38. absorption (blue line) and emission spectrum of **7** (dashed red line; excitation at 457 nm) measured in CH_2Cl_2 + 1% NEt_3 at rt.

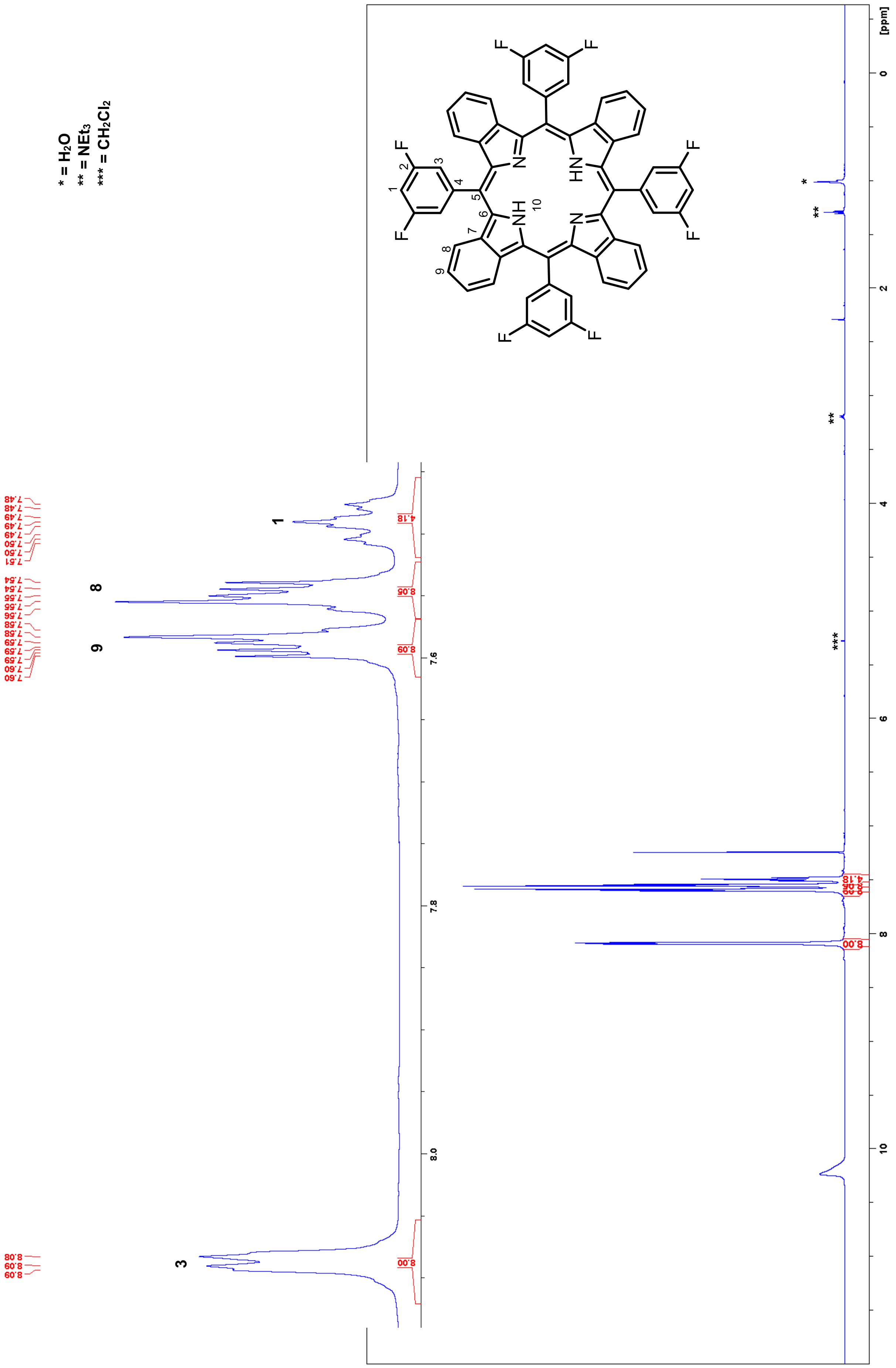

Figure S39. ^{1}H NMR (600 MHz, $CDCl_3$/TFA-d_1, rt) of **8**.

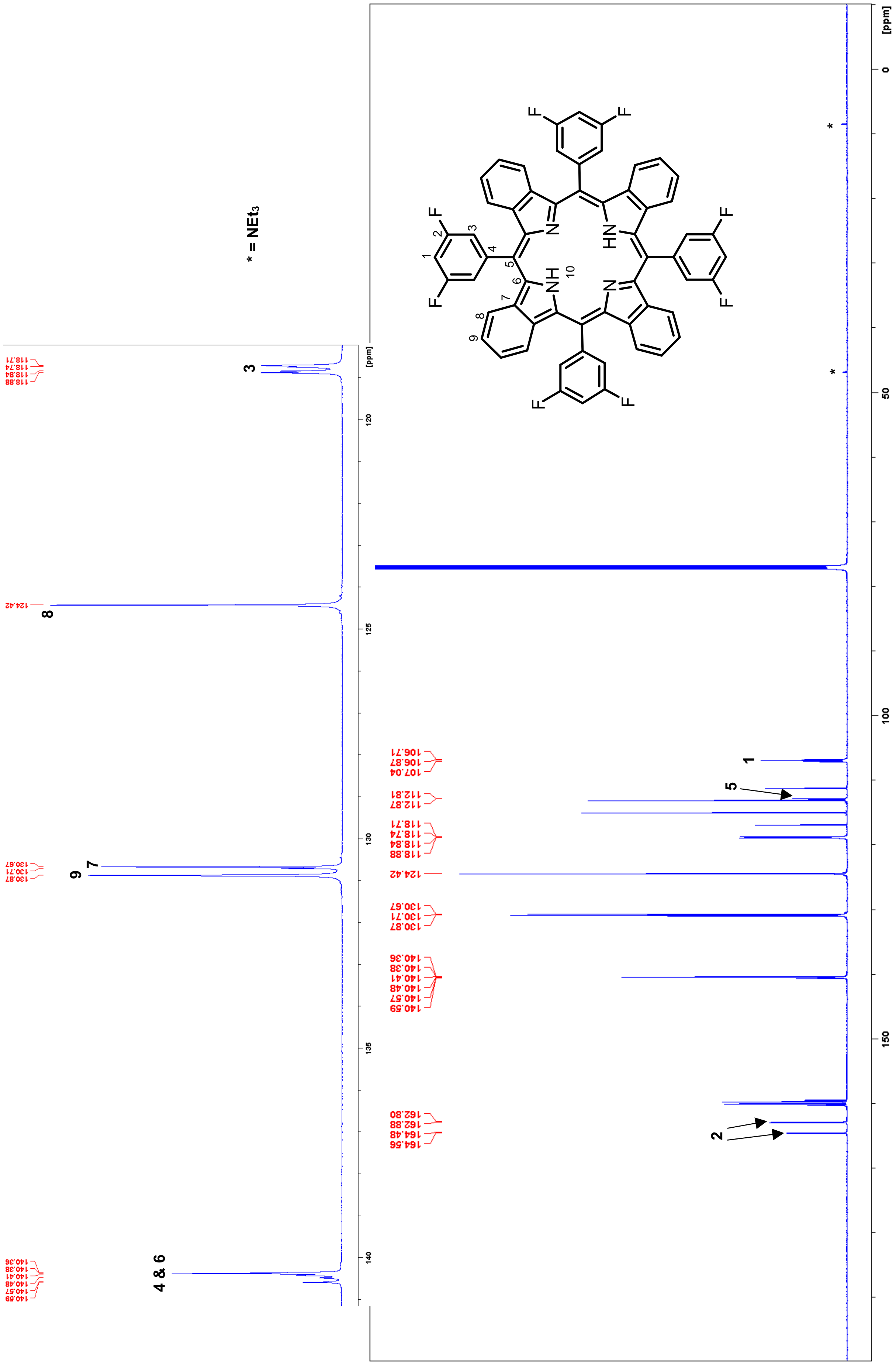

Figure S40. ^{13}C NMR (150 MHz, $CDCl_3$/TFA-d_1, rt) of **8**.

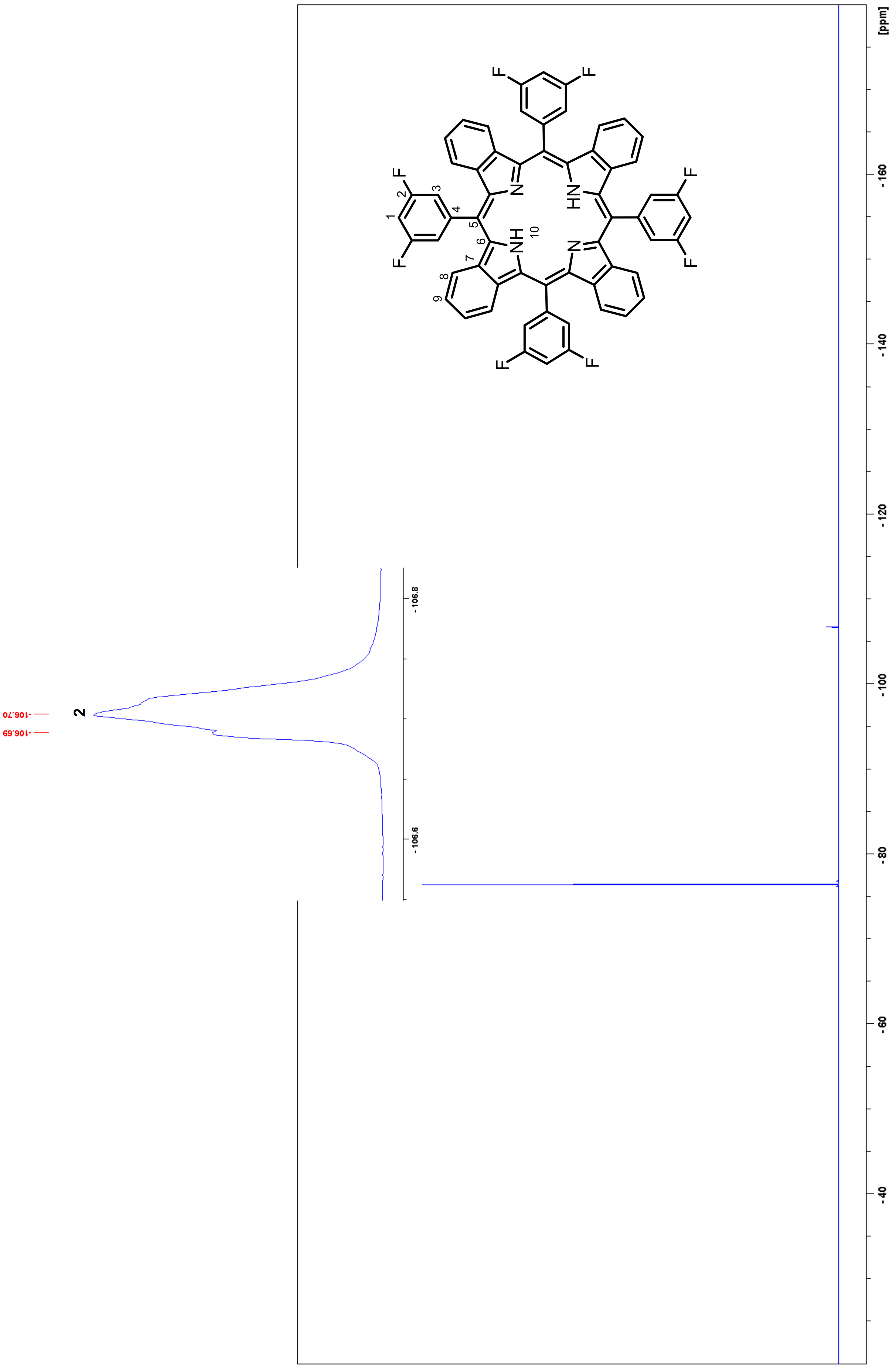

Figure S41. ^{19}F NMR (470 MHz, $CDCl_3$/TFA-d_1, rt) of **8**.

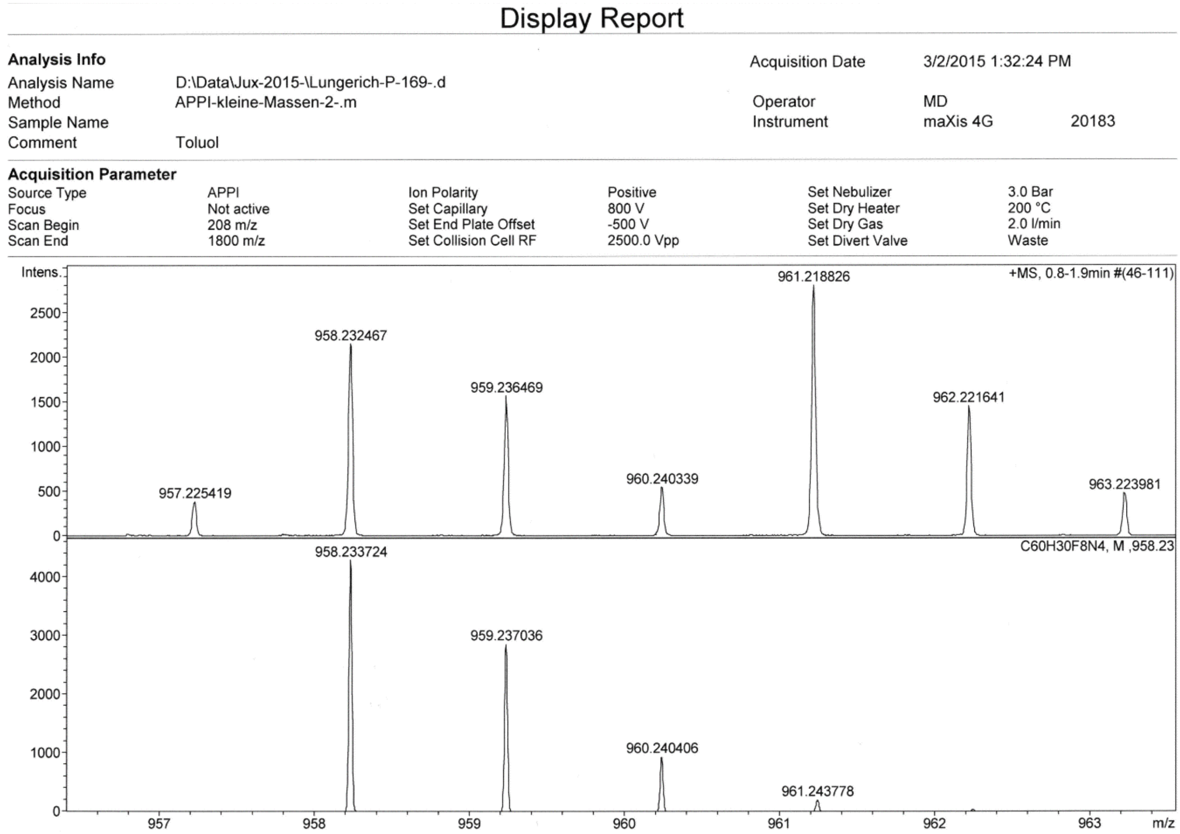

Figure S42. HRMS (APPI, toluene) of **8**.

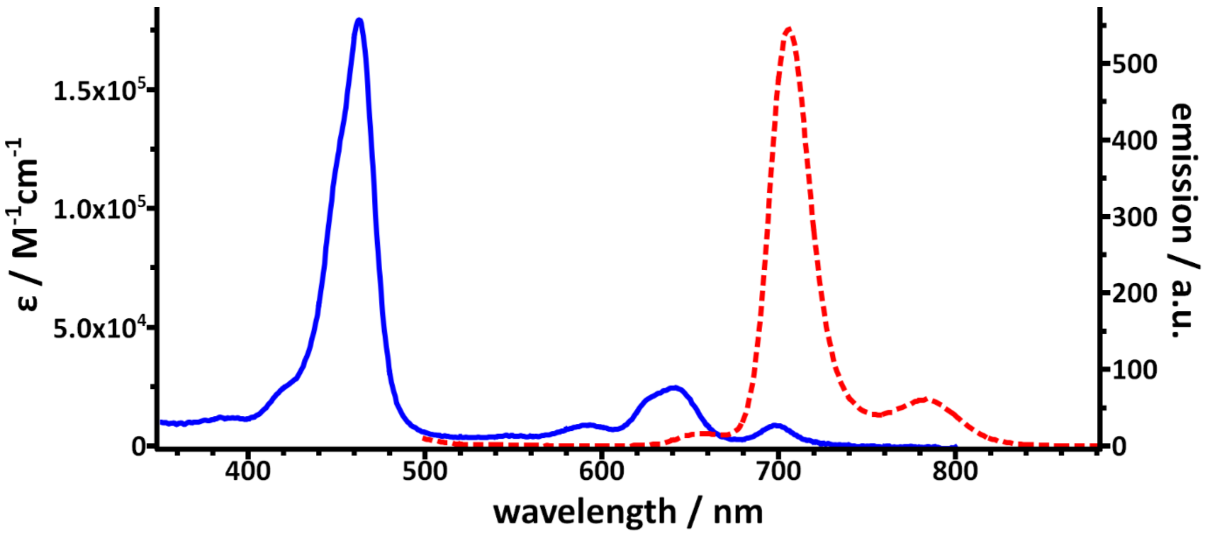

Figure S43. absorption (blue line) and emission spectrum of **8** (dashed red line; excitation at 463 nm) measured in CH_2Cl_2 + 1% NEt_3 at rt.

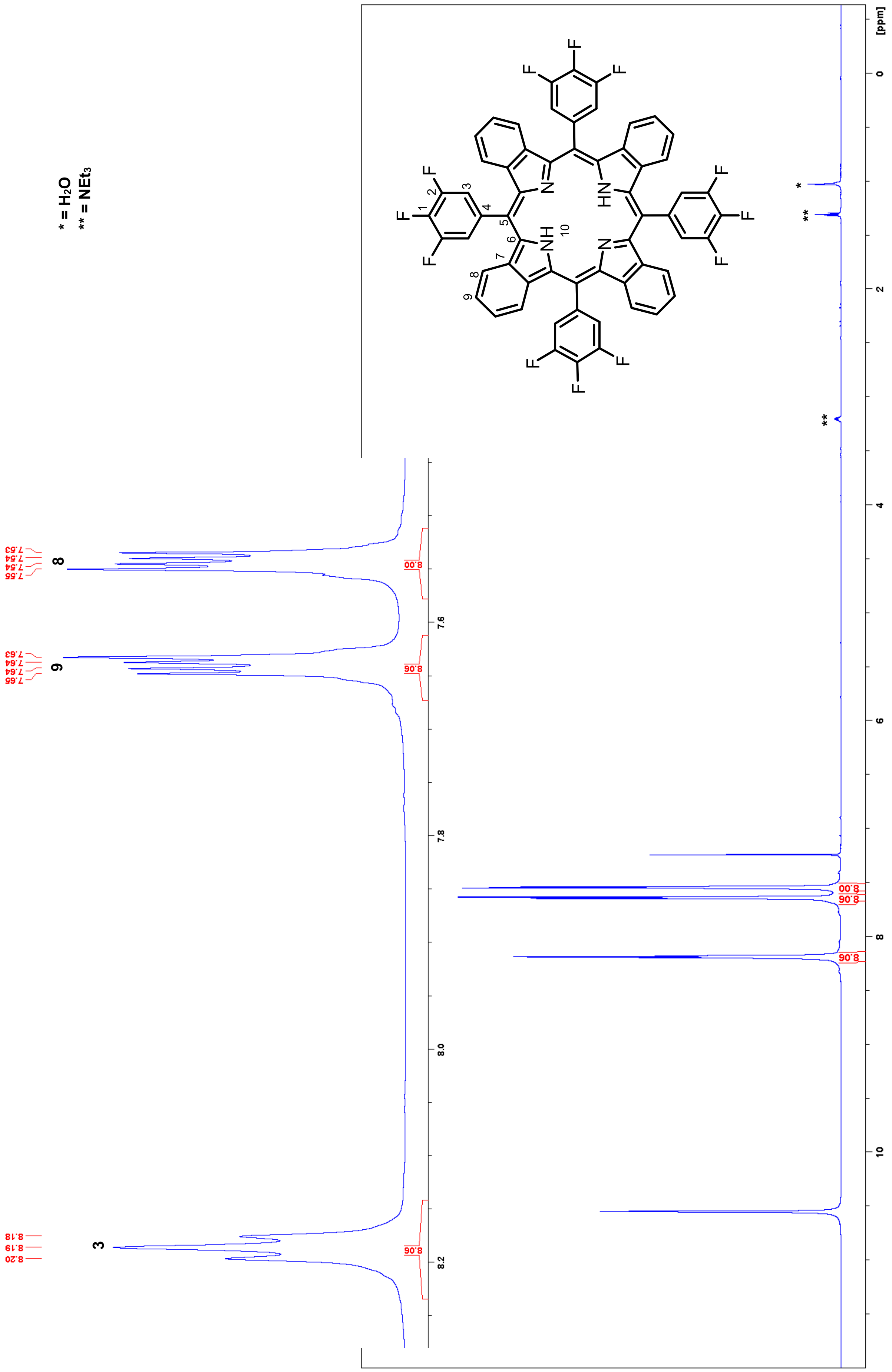

Figure S44. ^{1}H NMR (600 MHz, $CDCl_3$/TFA-d_1, rt) of **9**.

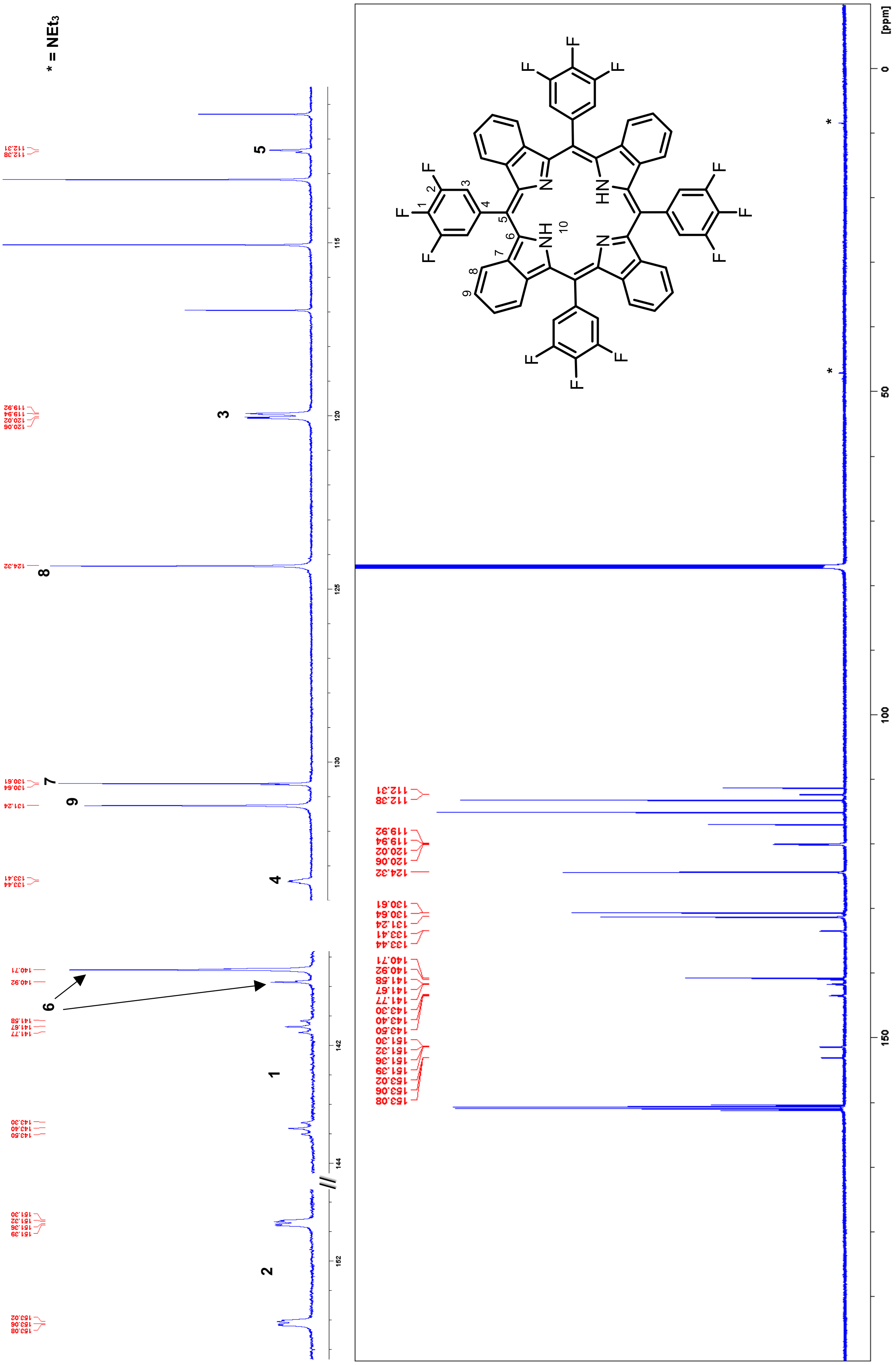

Figure S45. ^{13}C NMR (150 MHz, $CDCl_3$/TFA-d_1, rt) of **9**.

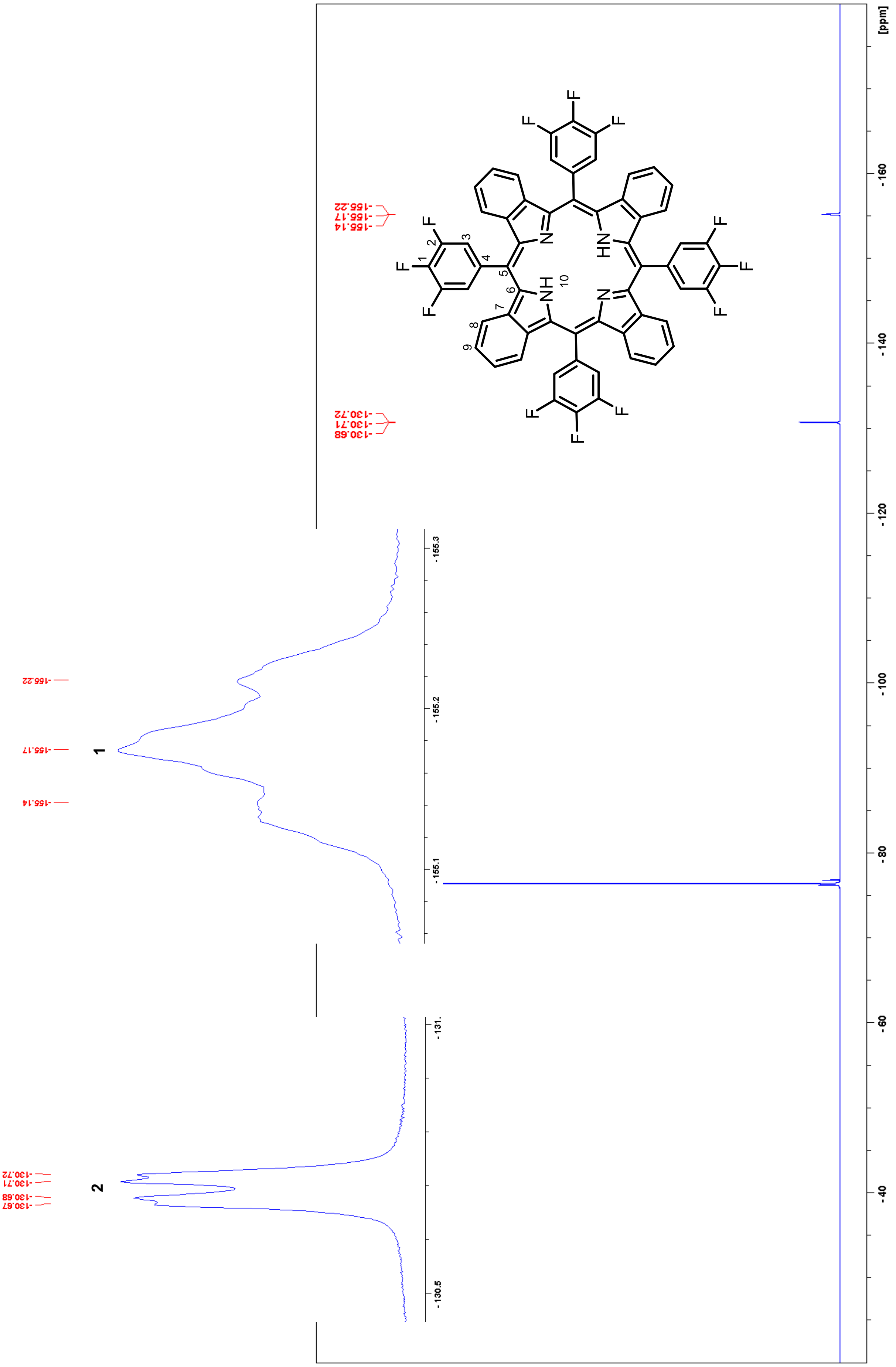

Figure S46. ^{19}F NMR (470 MHz, $CDCl_3$/TFA-d_1, rt) of **9**.

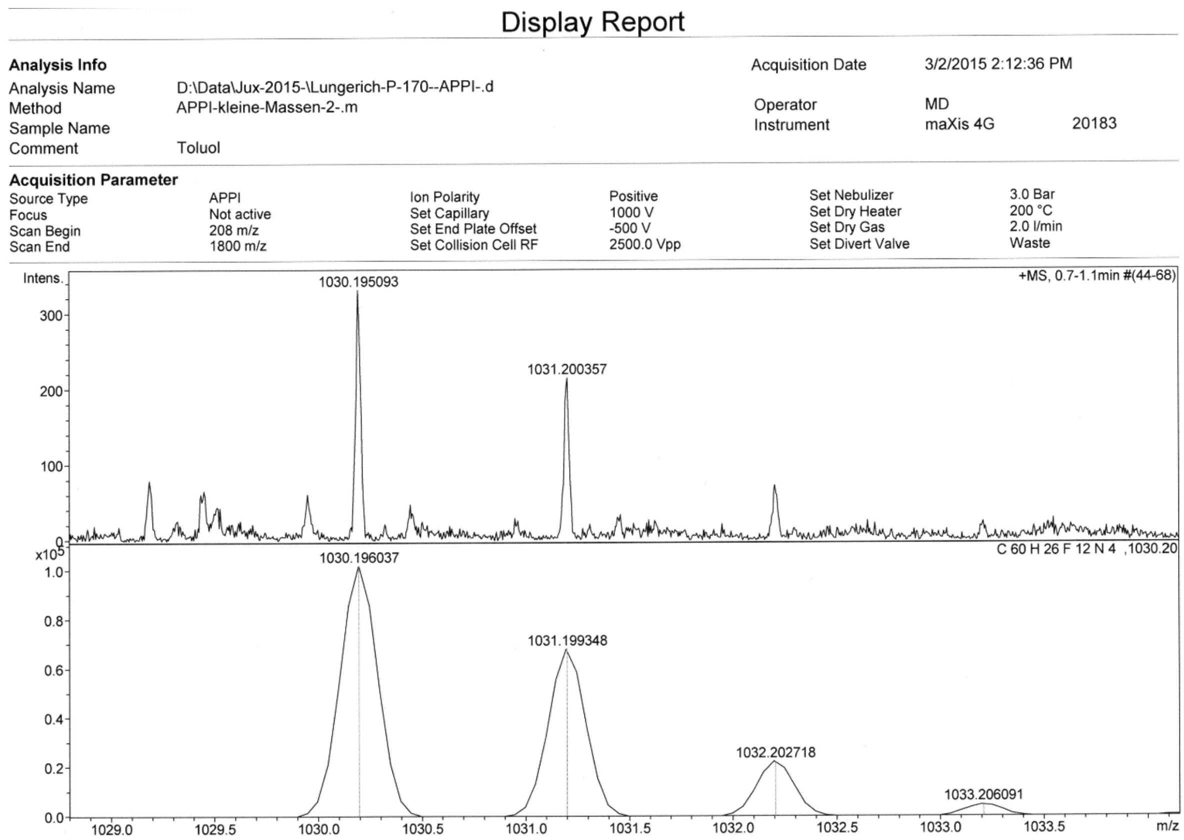

Figure S47. HRMS (APPI, toluene) of **9**.

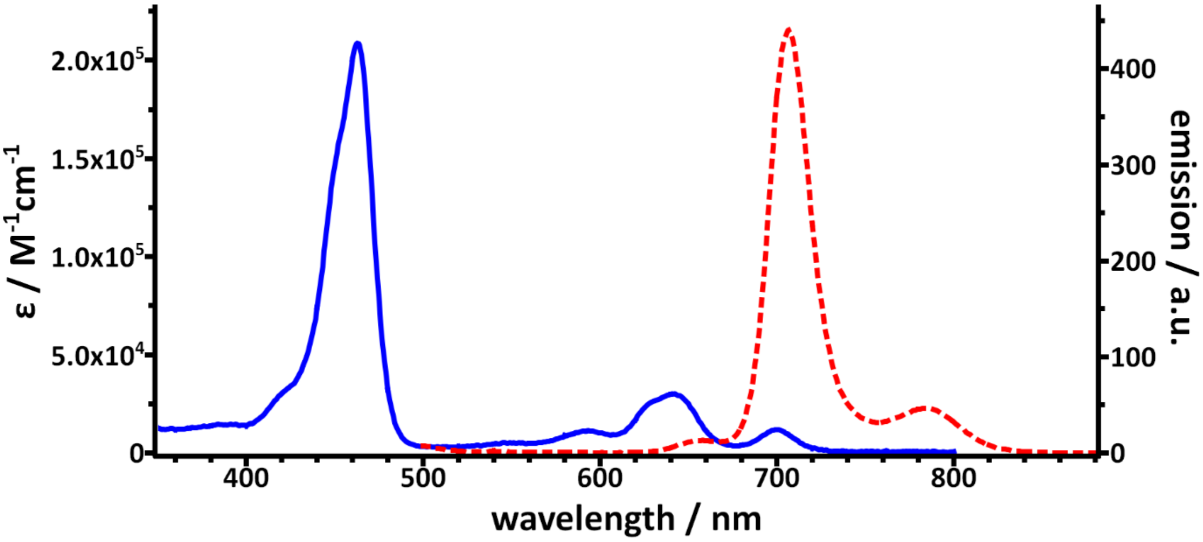

Figure S48. absorption (blue line) and emission spectrum of **9** (dashed red line; excitation at 463 nm) measured in CH_2Cl_2 + 1% NEt_3 at rt.

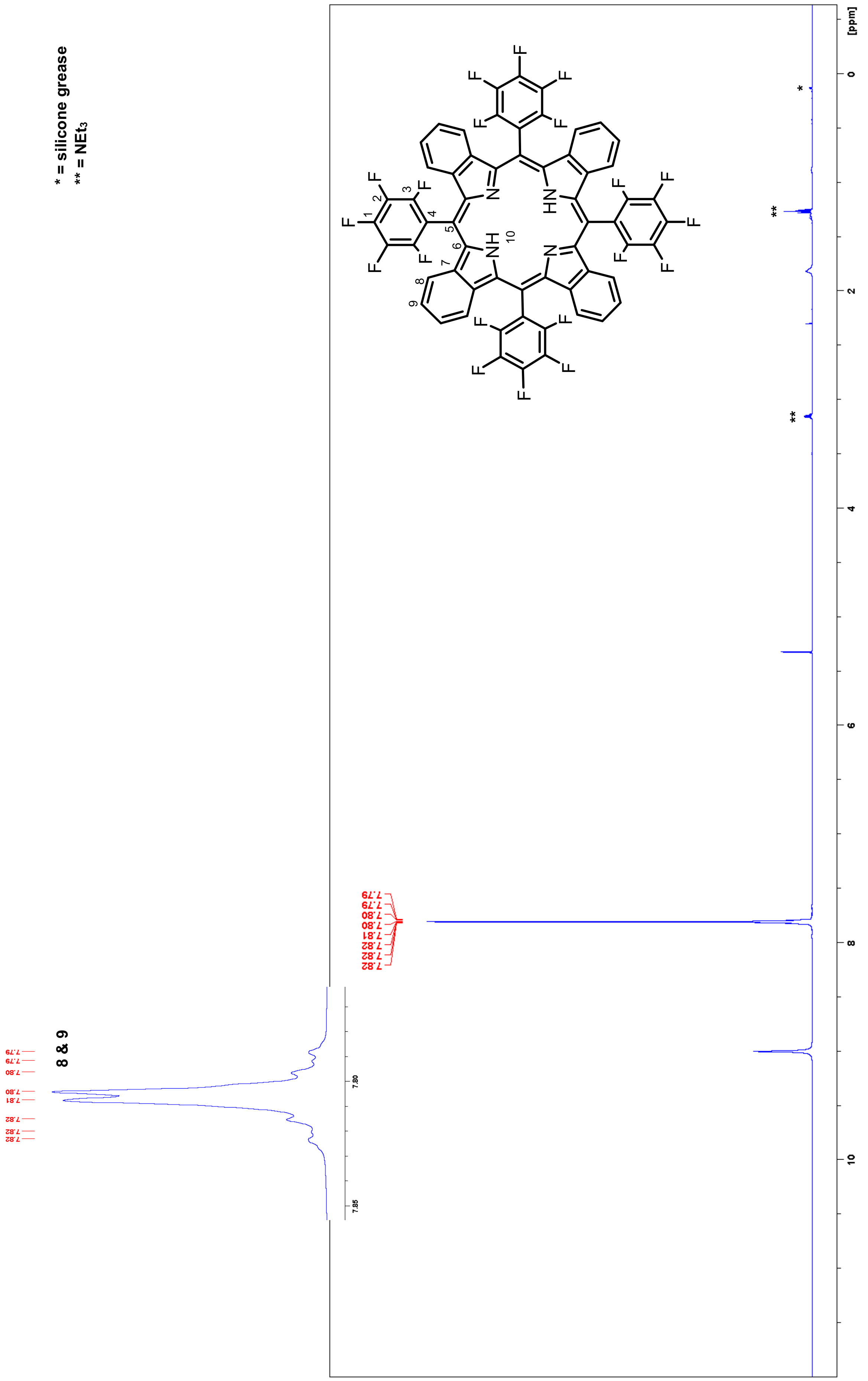

Figure S49. ^{1}H NMR (600 MHz, CD_2Cl_2/TFA-d_1, rt) of **10**.

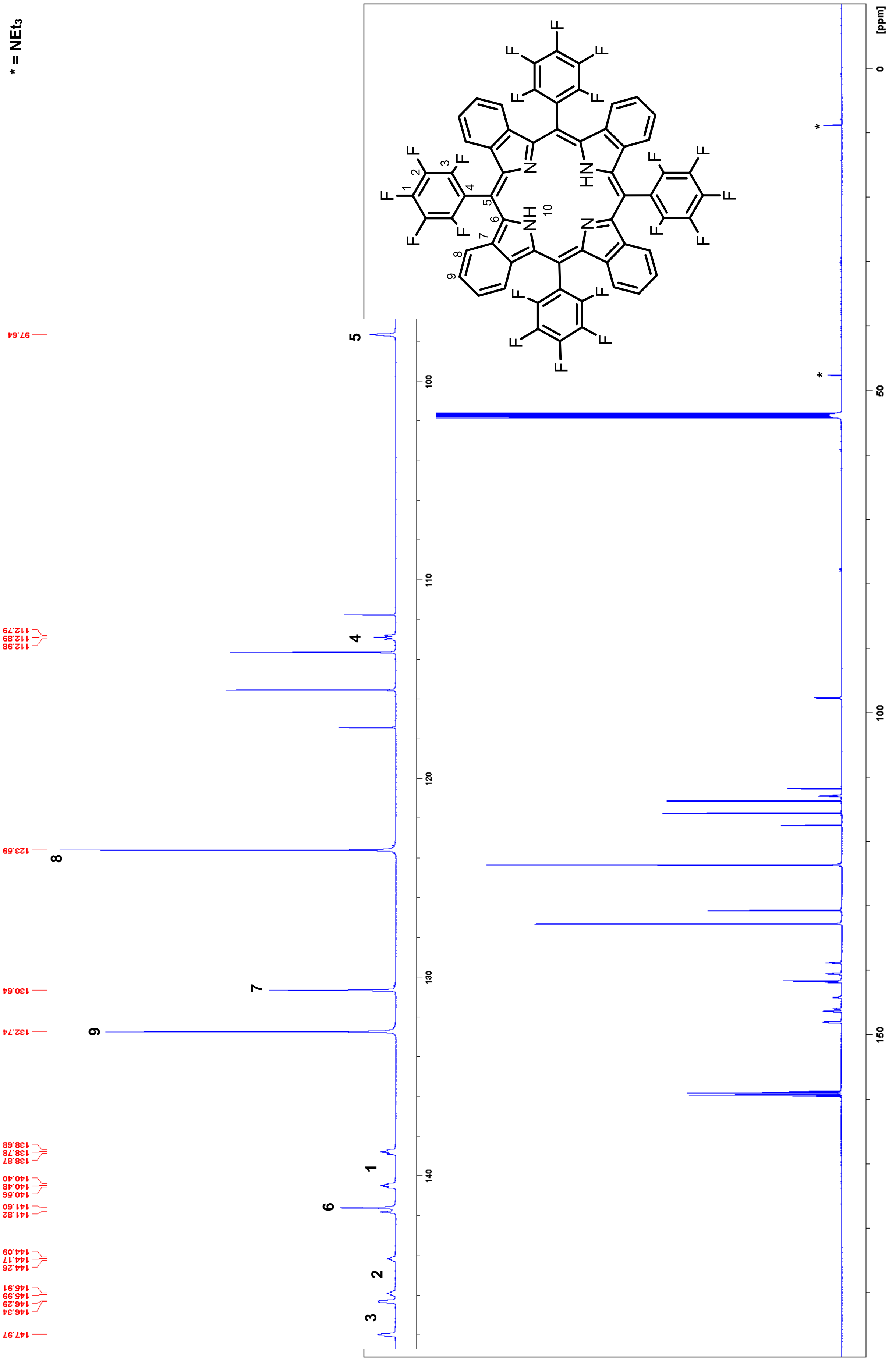

Figure S50. ^{13}C NMR (150 MHz, CD_2Cl_2/TFA-d_1, rt) of **10**.

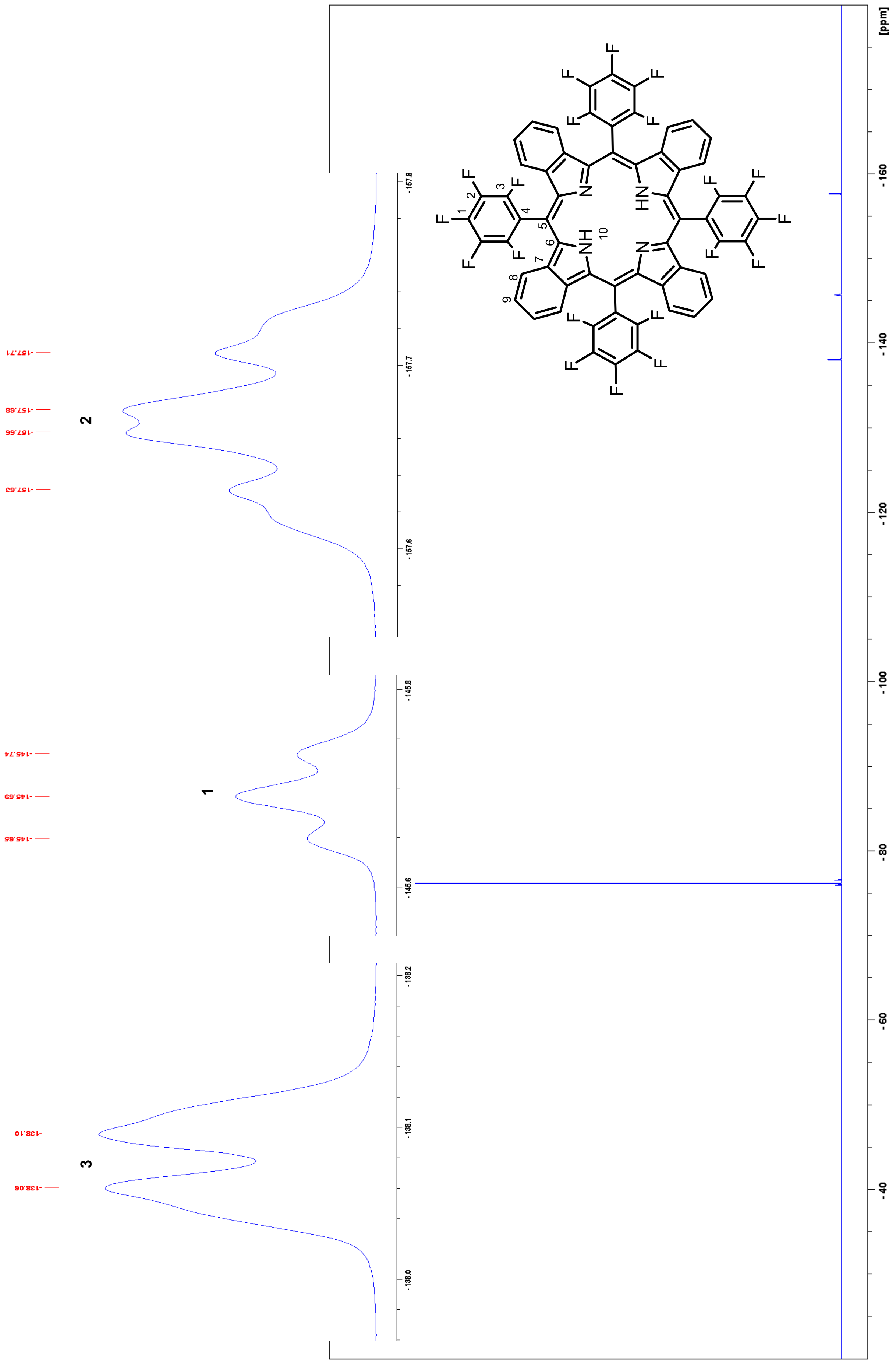

Figure S51. ^{19}F NMR (150 MHz, CD_2Cl_2/TFA-d_1, rt) of **10**.

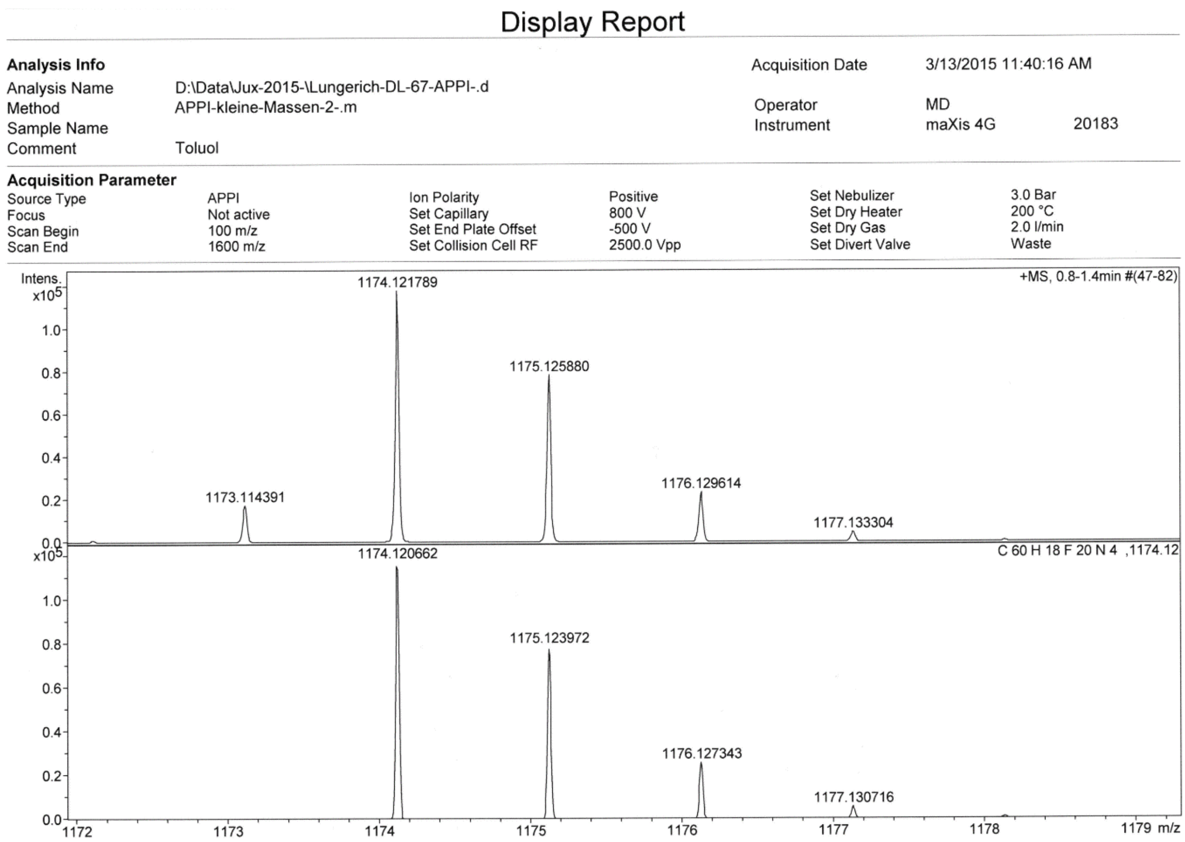

Figure S52. HRMS (APPI, toluene) of **10**.

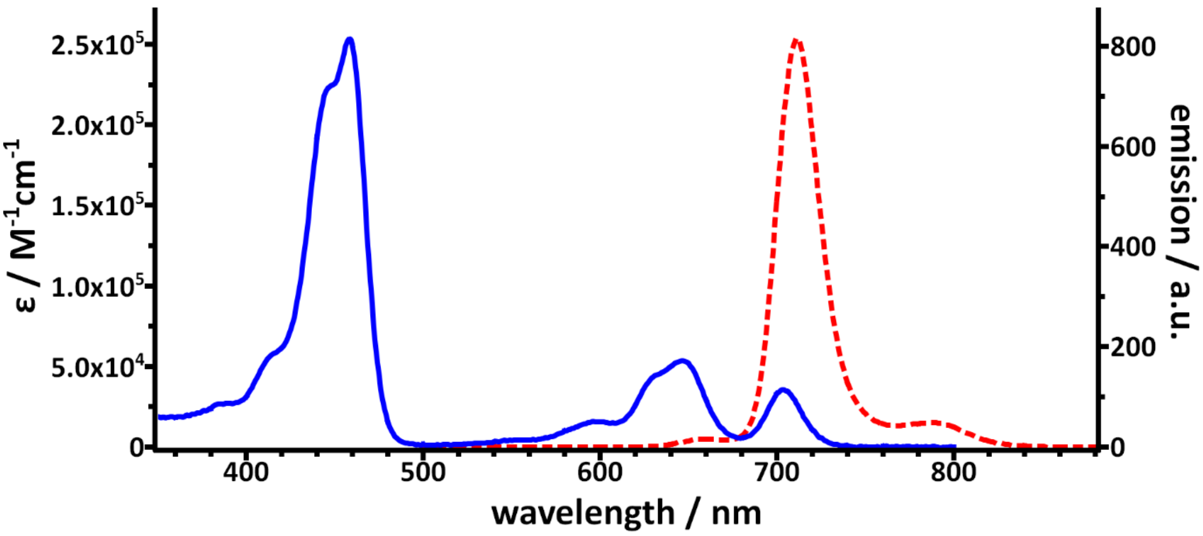

Figure S53. absorption (blue line) and emission spectrum of **10** (dashed red line; excitation at 458 nm) measured in CH_2Cl_2 + 1% NEt_3 at rt.

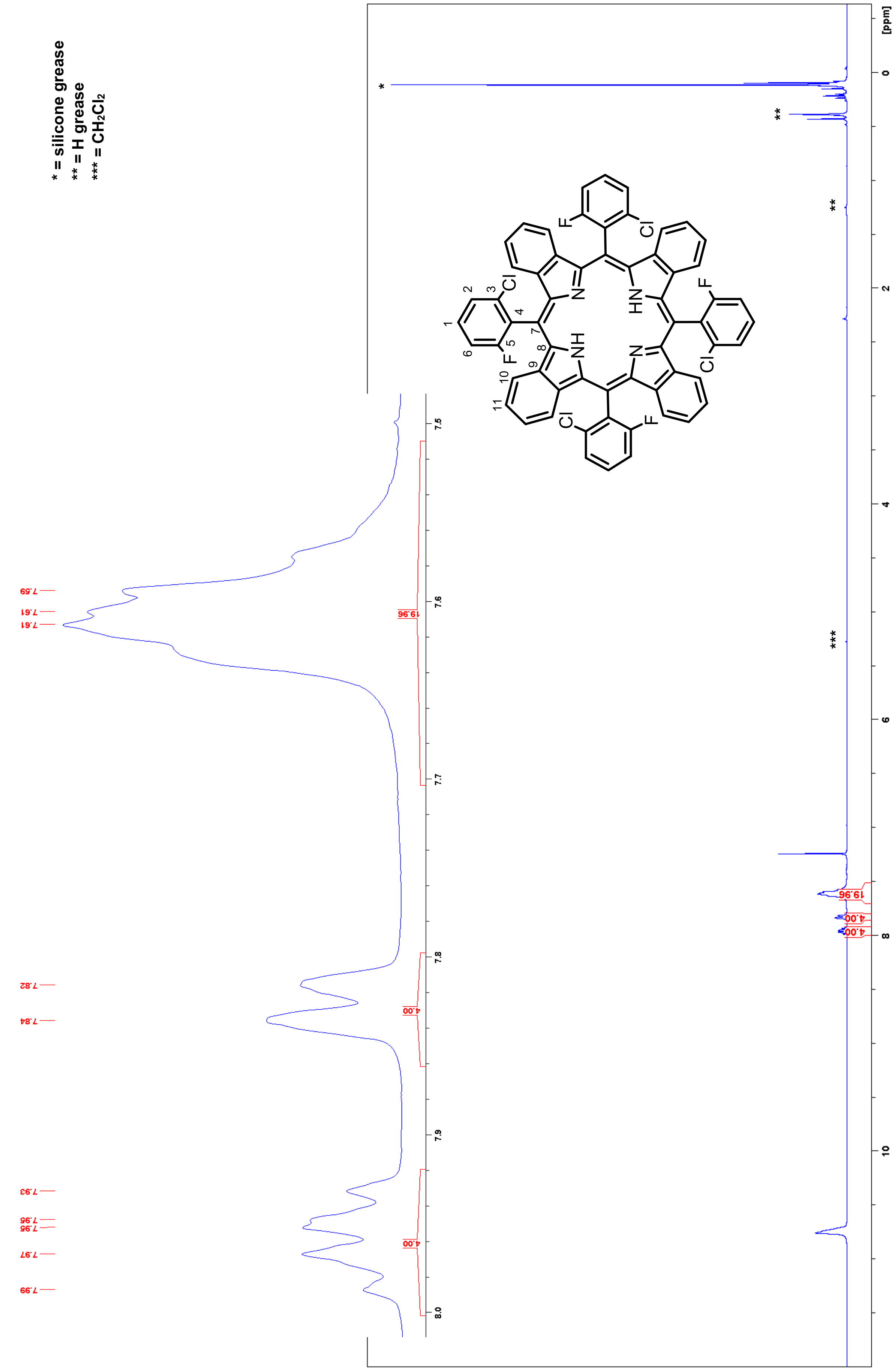

Figure S54. ^{1}H NMR (400 MHz, $CDCl_3$/TFA-d_1, rt) of **11**.

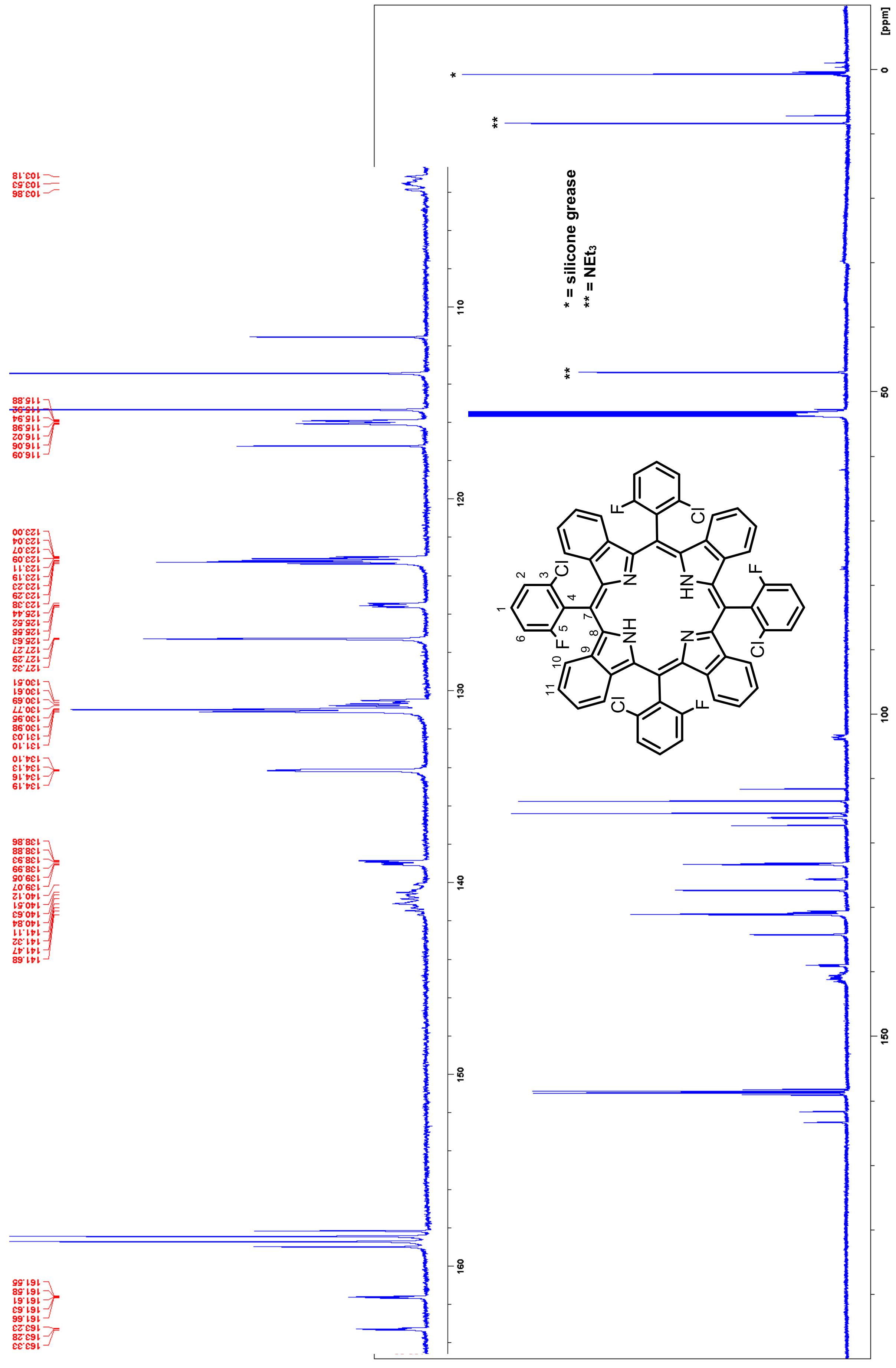

Figure S55. ^{13}C NMR (150 MHz, CD_2Cl_2/TFA-d_1, rt) of **11**.

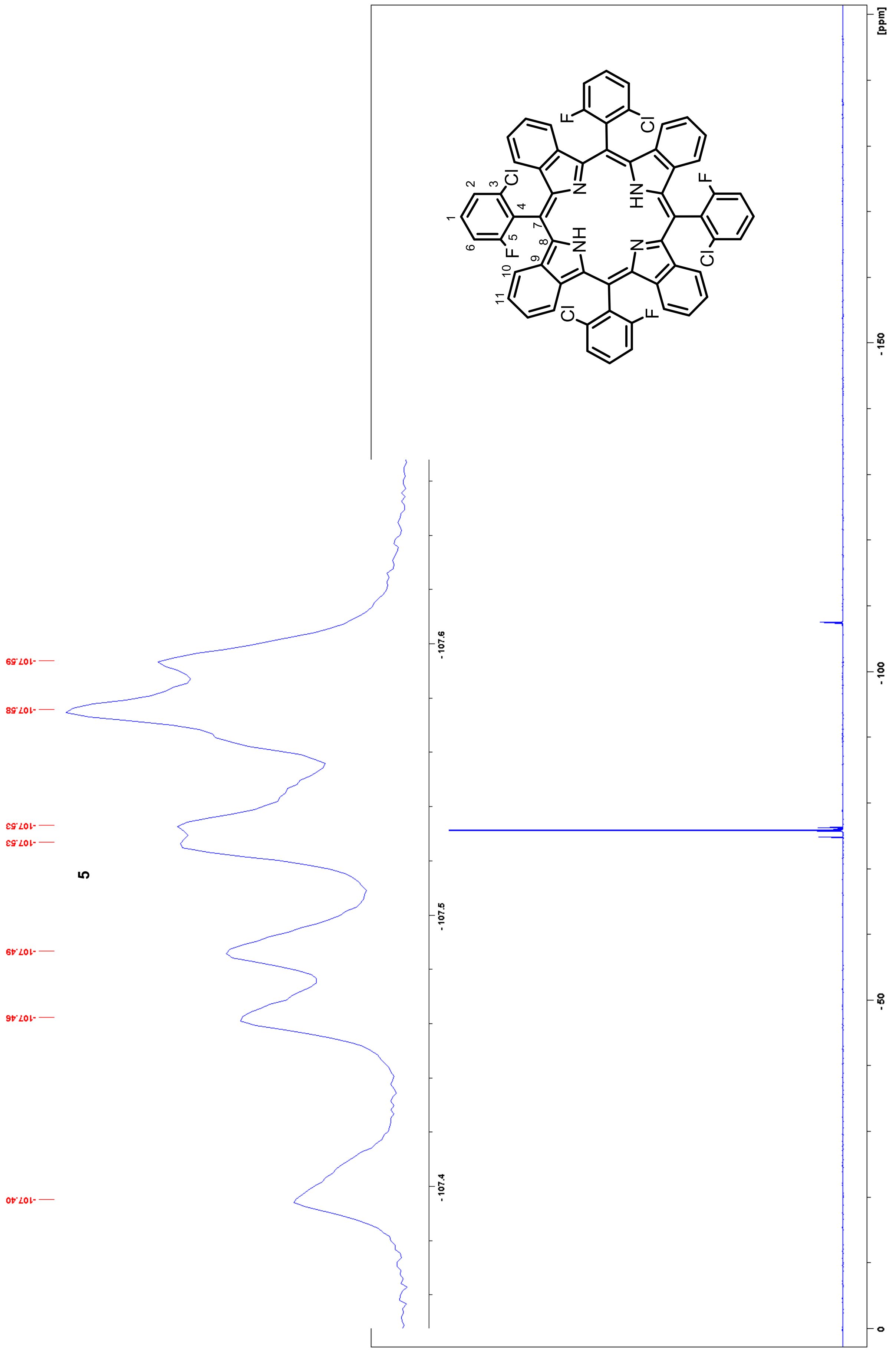

Figure S56. ^{19}F NMR (470 MHz, CD_2Cl_2/TFA-d_1, rt) of **11**.

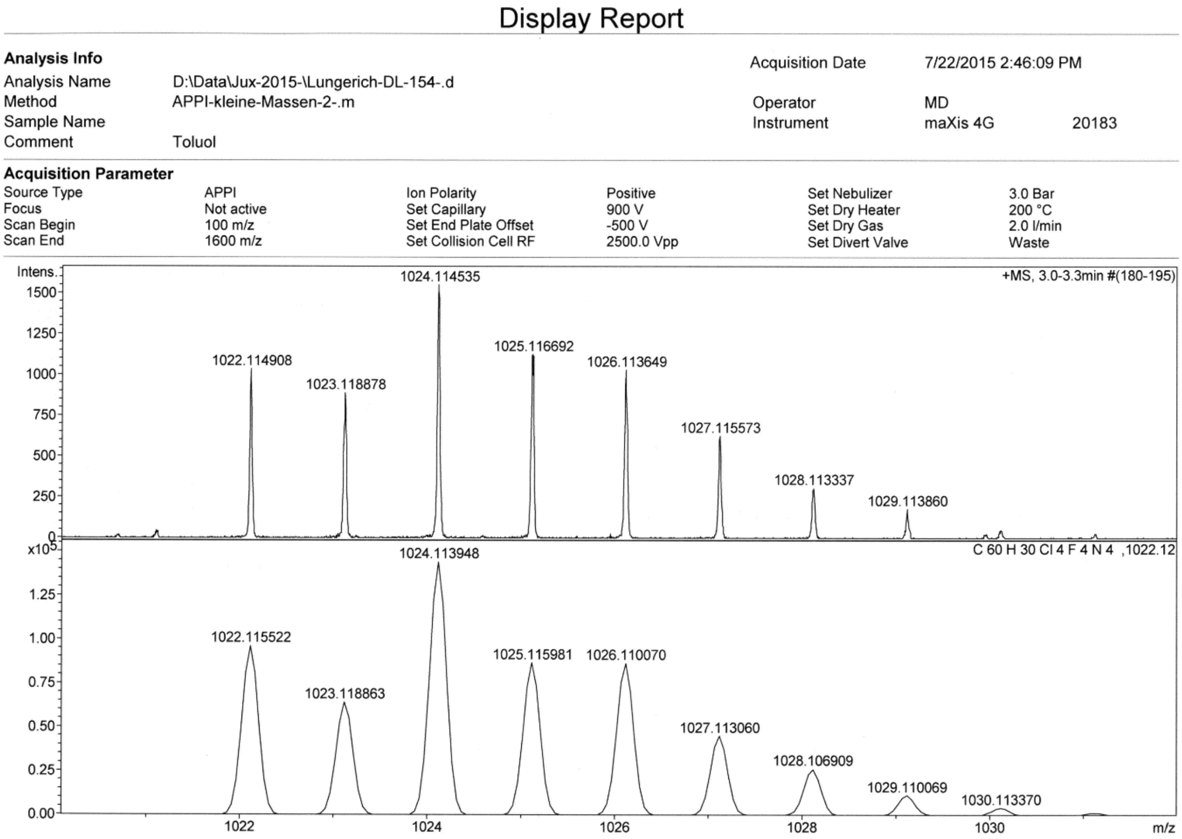

Figure S57. HRMS (APPI, toluene) of **11**.

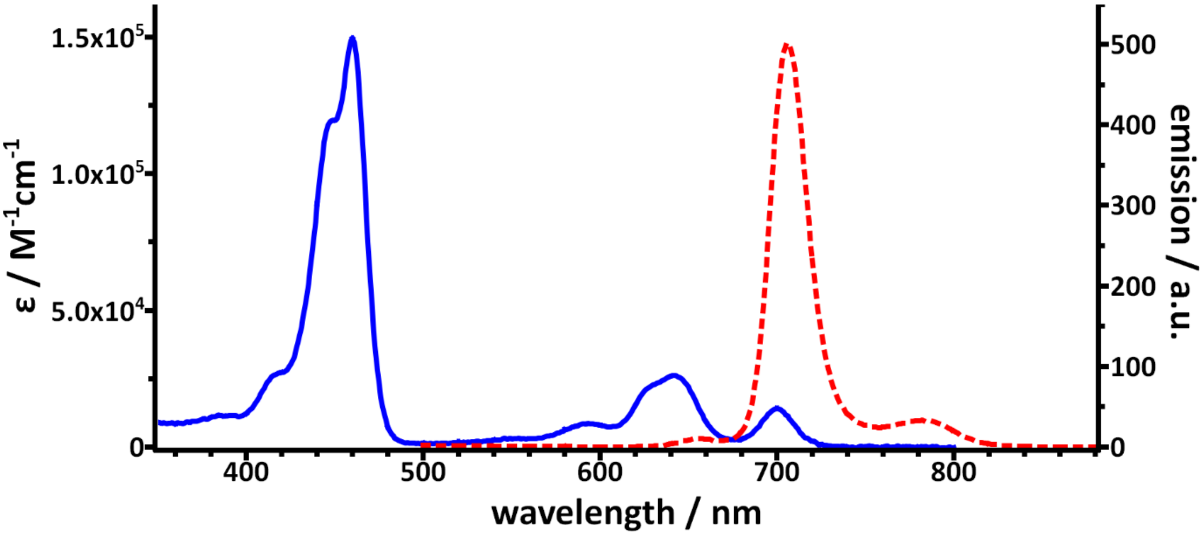

Figure S58. absorption (blue line) and emission spectrum of **11** (dashed red line; excitation at 460 nm) measured in CH_2Cl_2 + 1% NEt_3 at rt.

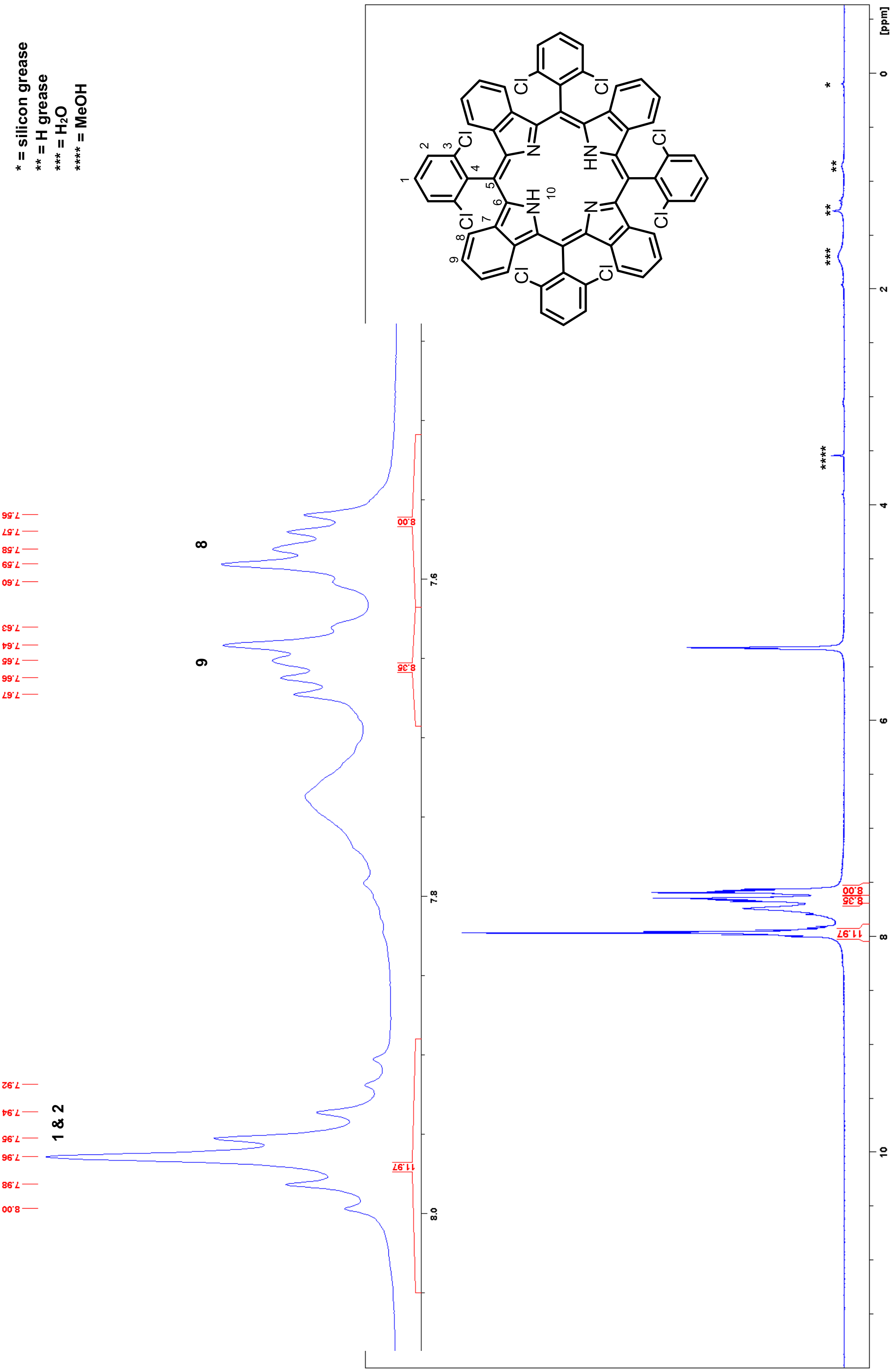

Figure S59. ^{1}H NMR (300 MHz, CD_2Cl_2/TFA-d_1, rt) of **12**.

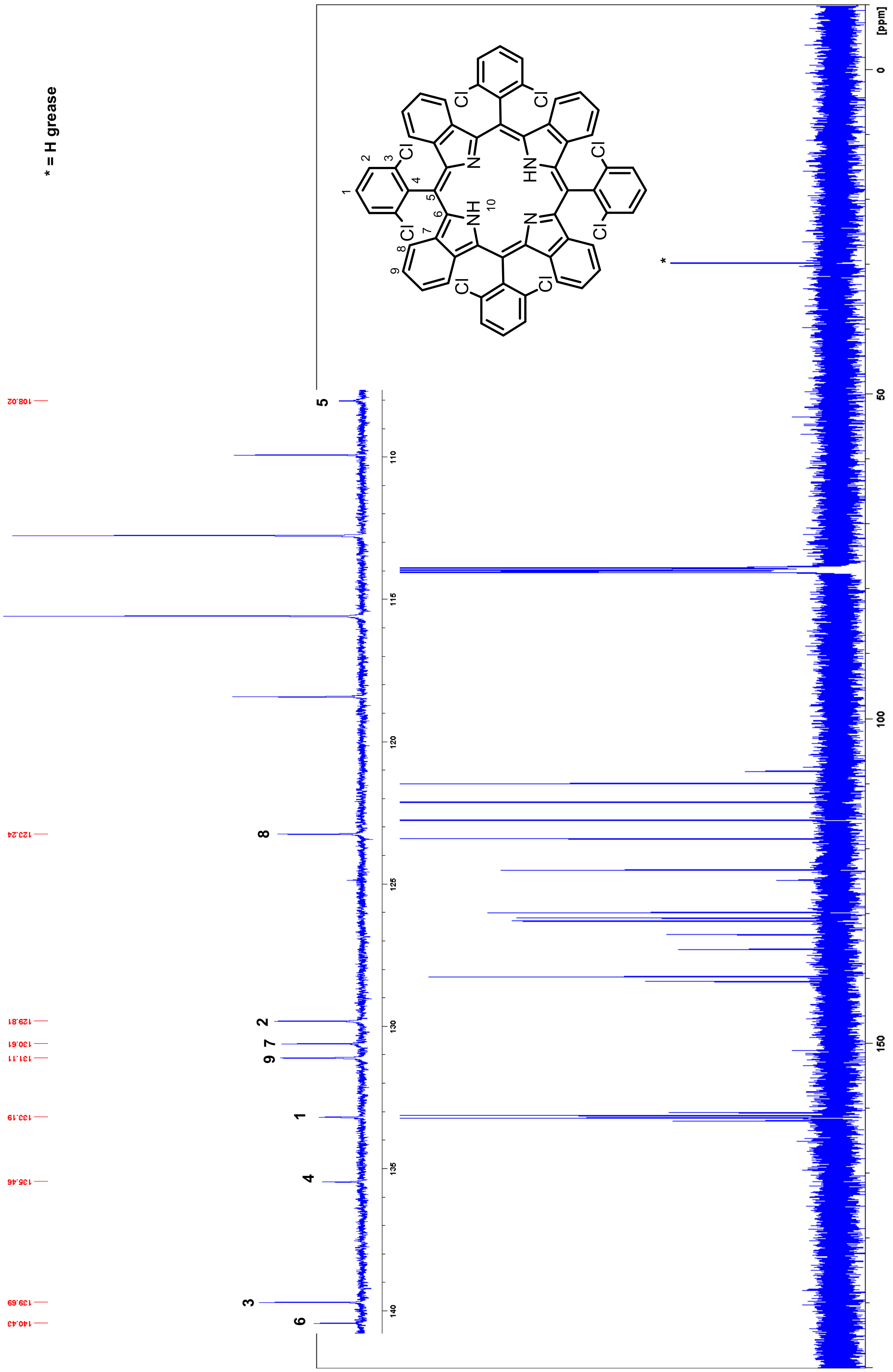

Figure S60. ^{13}C NMR (100 MHz, $CDCl_3$/TFA-d_1, rt) of **12**.

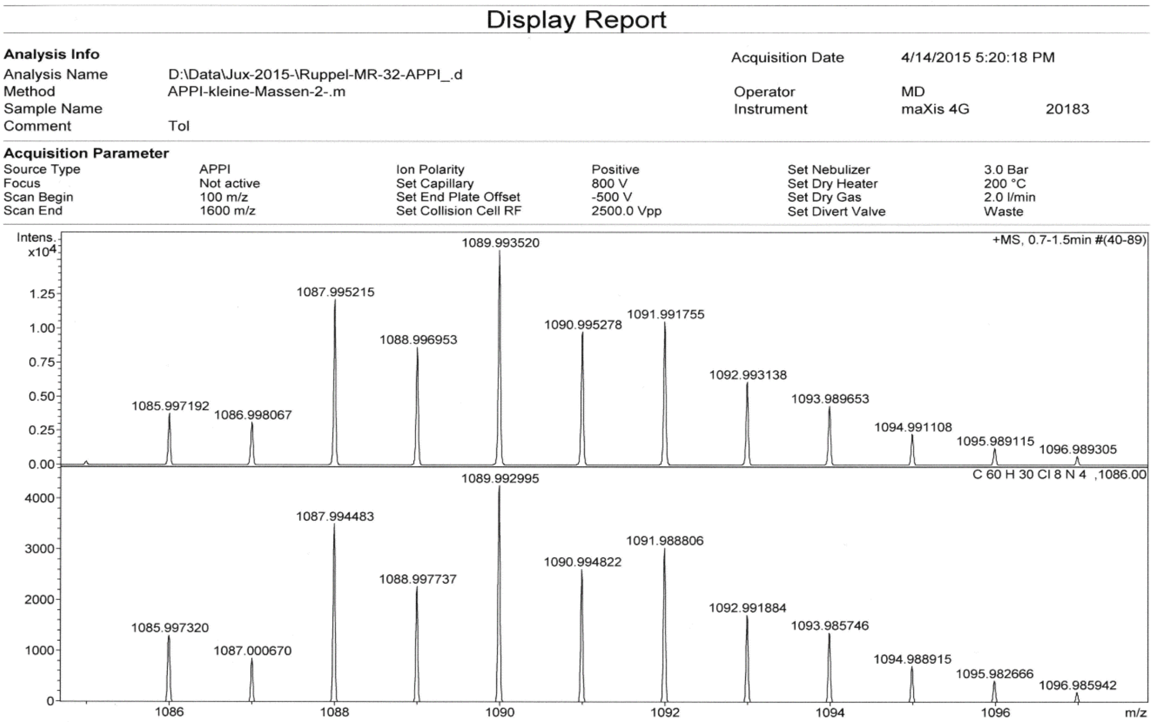

Figure S61. HRMS (APPI, toluene) of 1**2**.

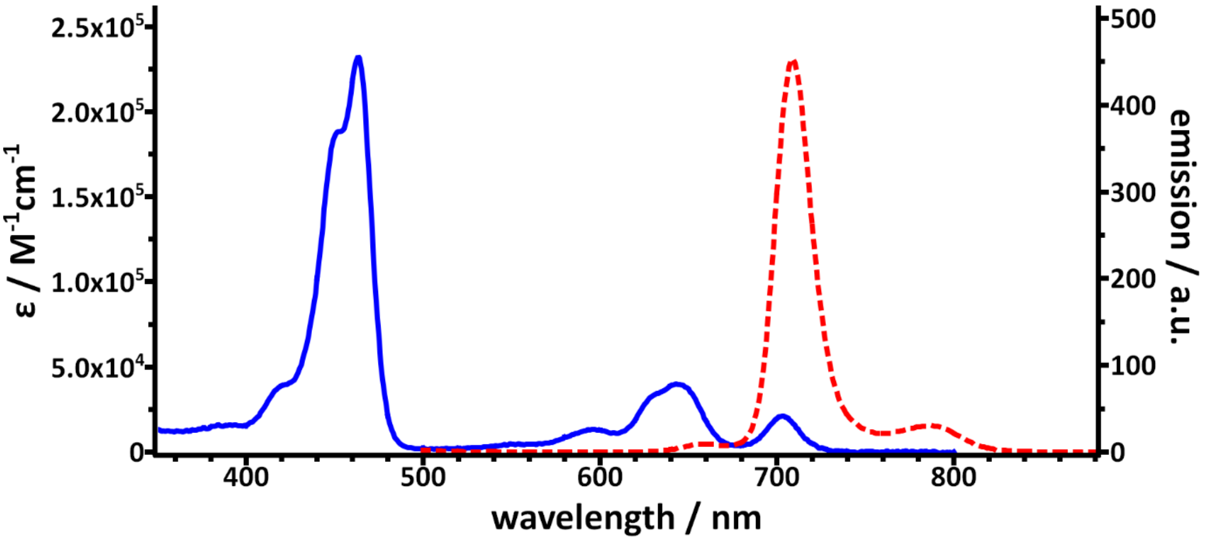

Figure S62. absorption (blue line) and emission spectrum of 1**2** (dashed red line; excitation at 457 nm) measured in CH_2Cl_2 + 1% NEt_3 at rt.

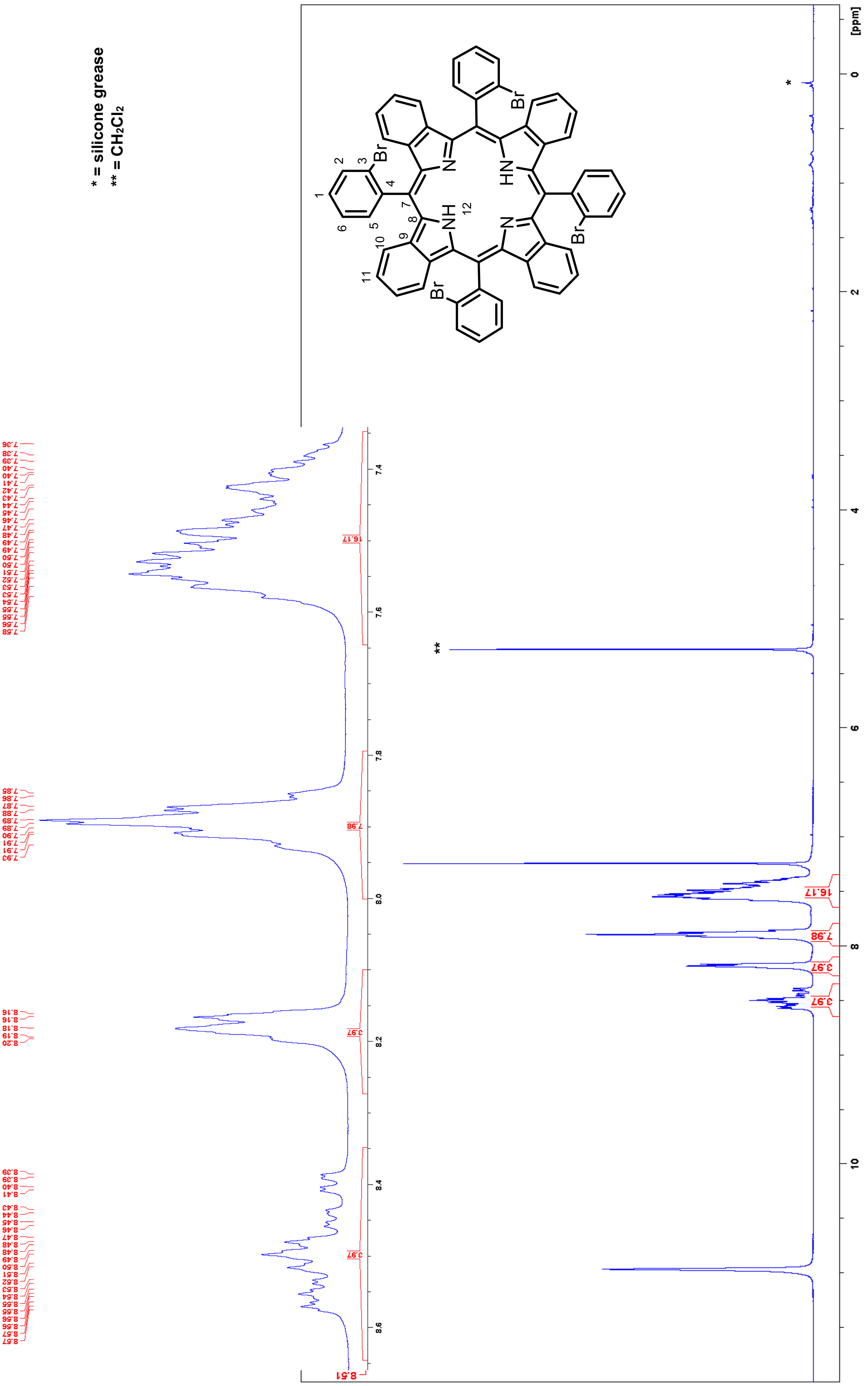

Figure S63. ^{1}H NMR (400 MHz, $CDCl_3$/TFA-d_1, rt) of **13**.

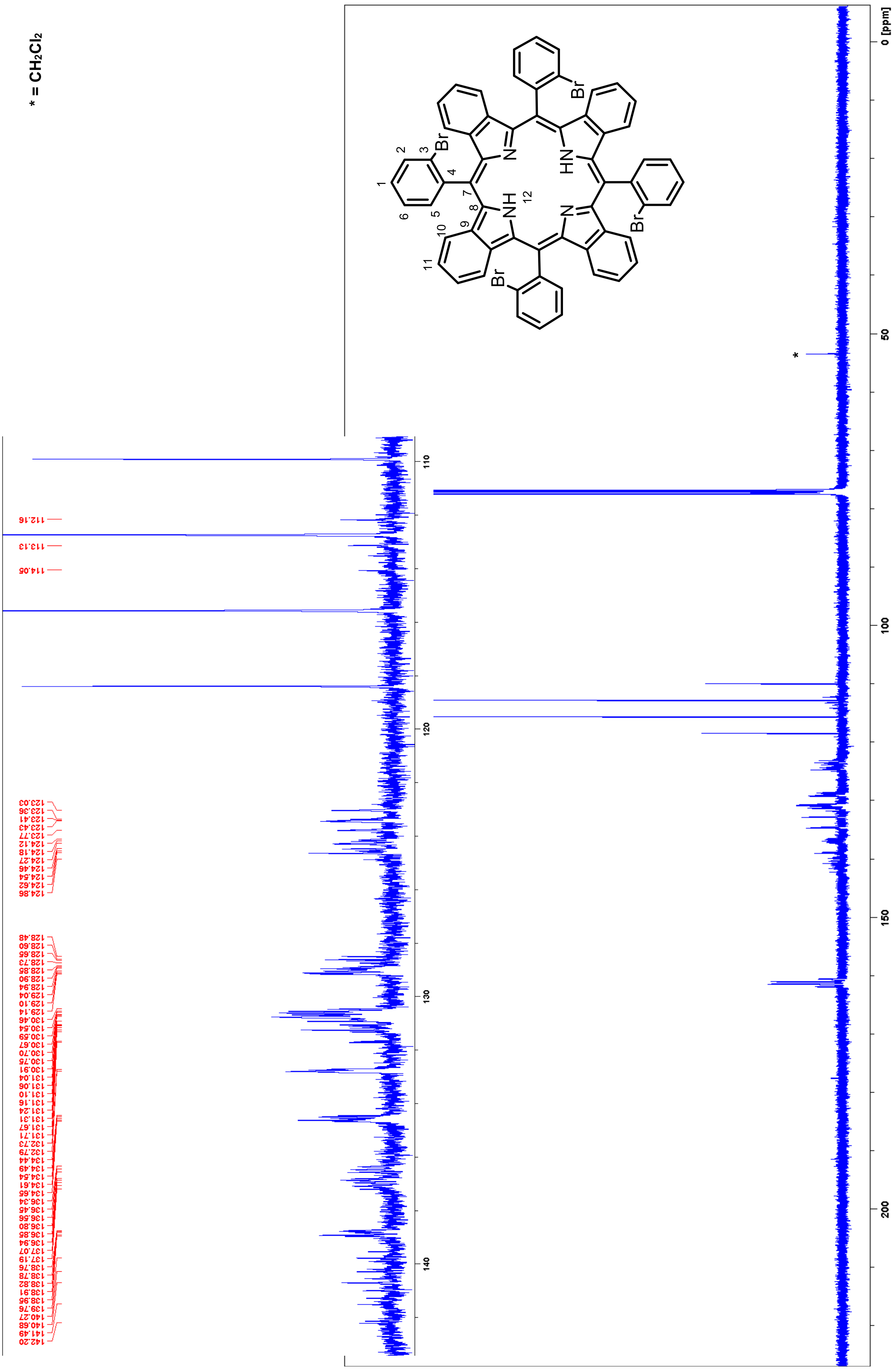

Figure S64. ^{13}C NMR (100 MHz, $CDCl_3$/TFA-d_1, rt) of **13**.

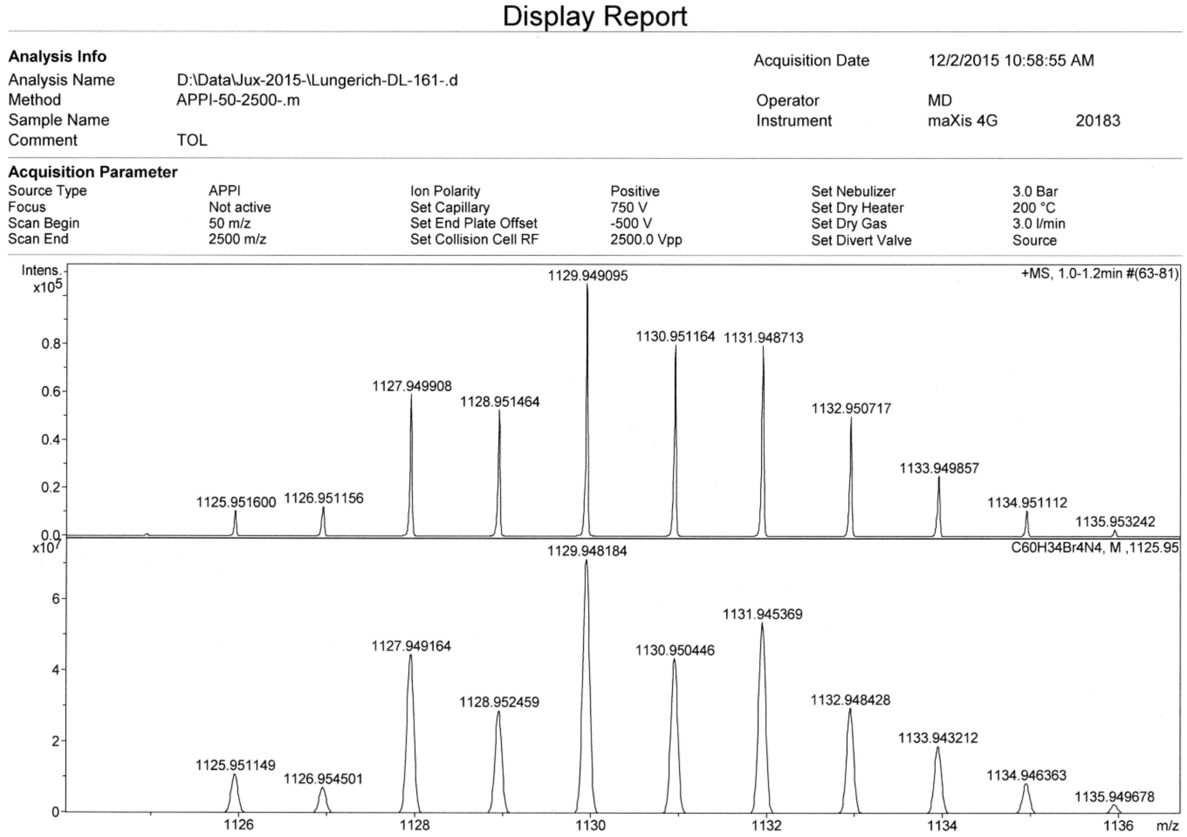

Figure S65. HRMS (APPI, toluene) of **13**.

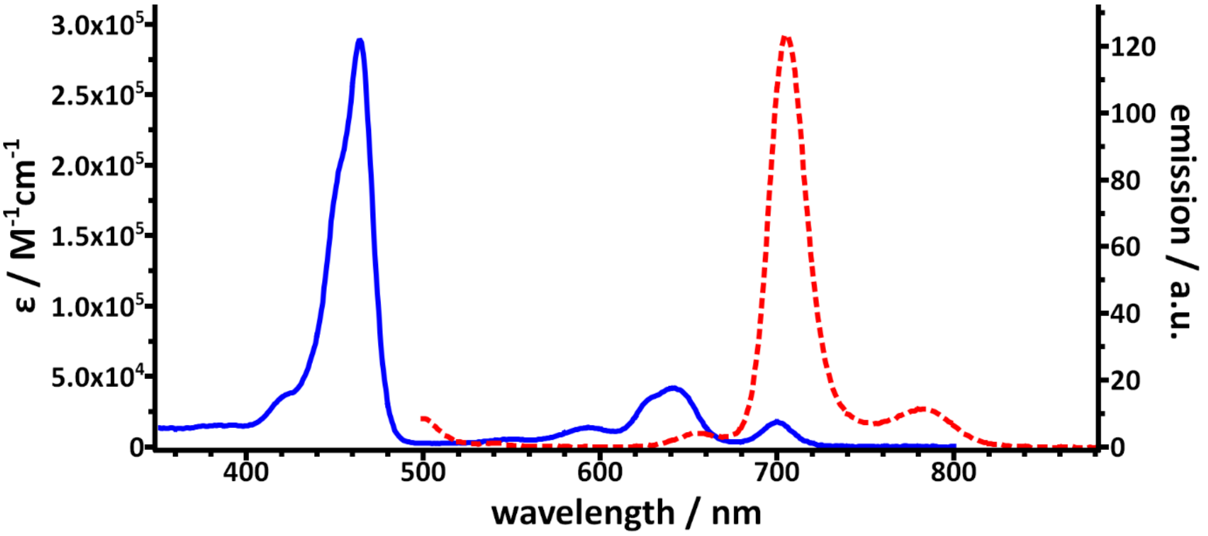

Figure S66. absorption (blue line) and emission spectrum of **13** (dashed red line; excitation at 464 nm) measured in CH_2Cl_2 + 1% NEt_3 at rt.

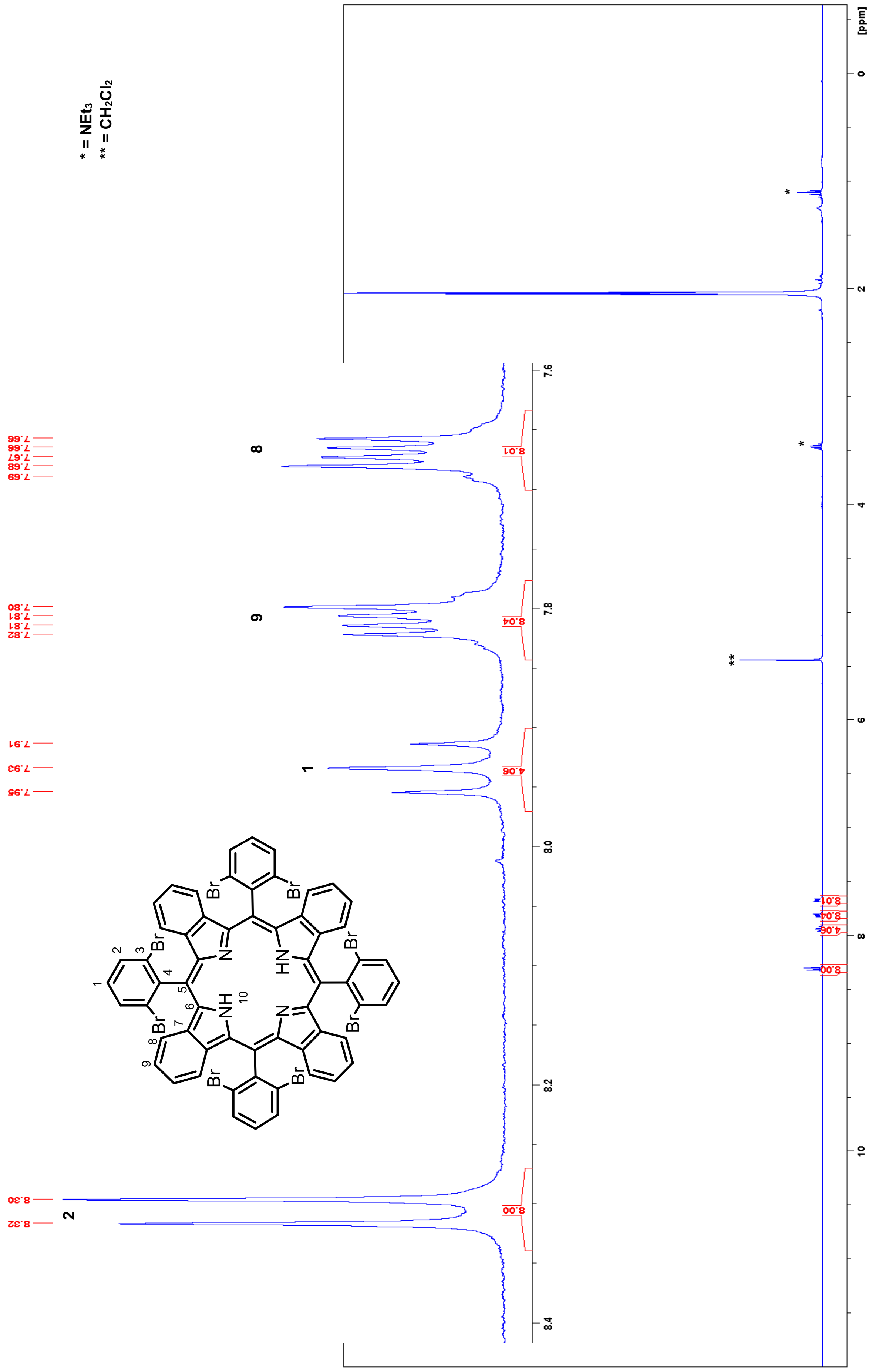

Figure S67. ^{1}H NMR (400 MHz, acetone-d_6/TFA-d_1/CS_2, rt) of **14**.

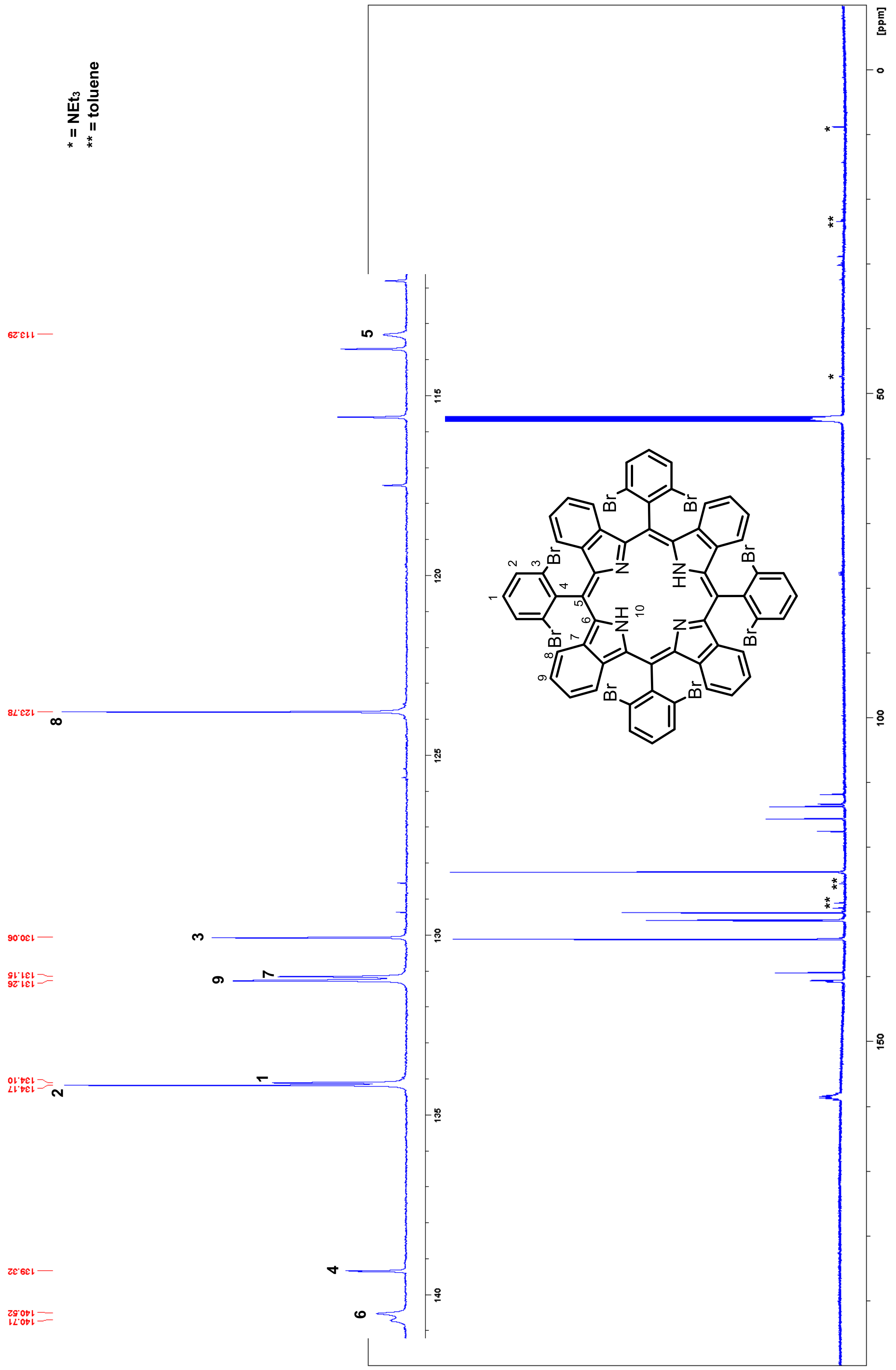

Figure S68. ^{13}C NMR (150 MHz, CD_2Cl_2/TFA-d_1, rt) of **14**.

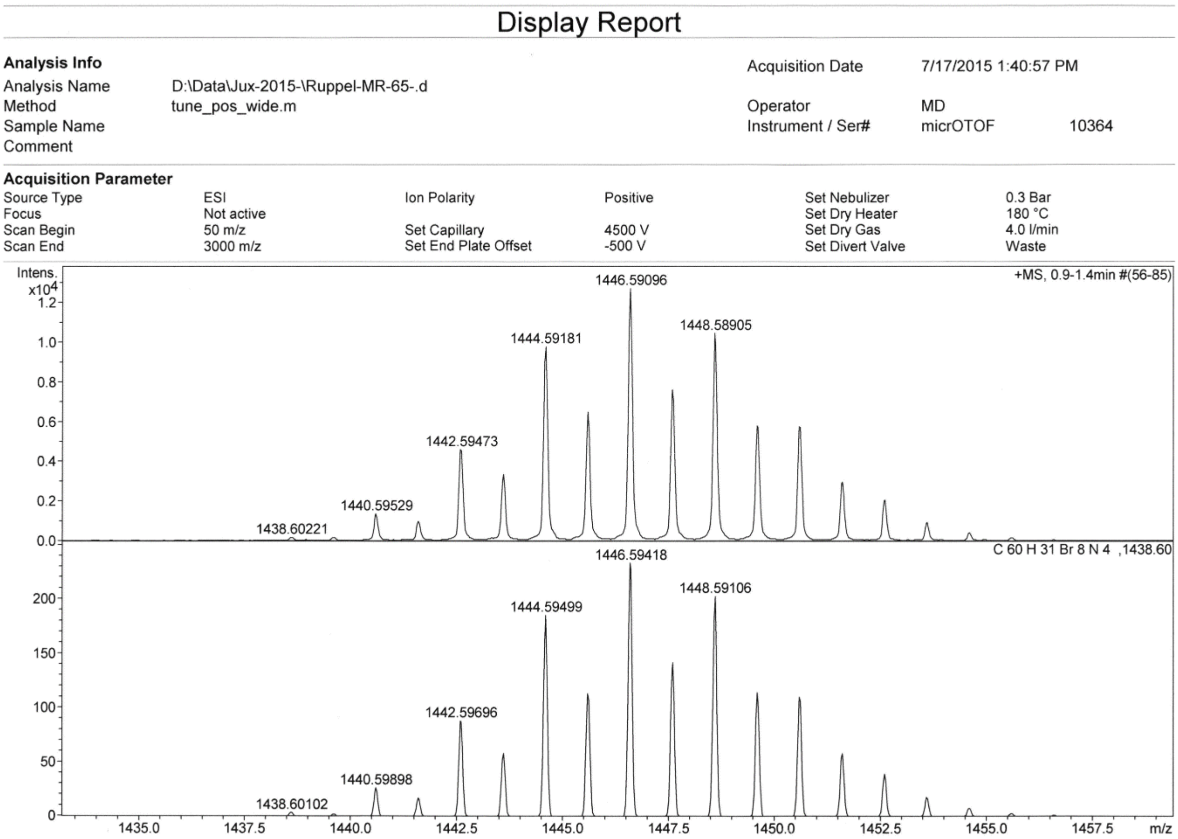

Figure S69. HRMS (ESI) of **14**.

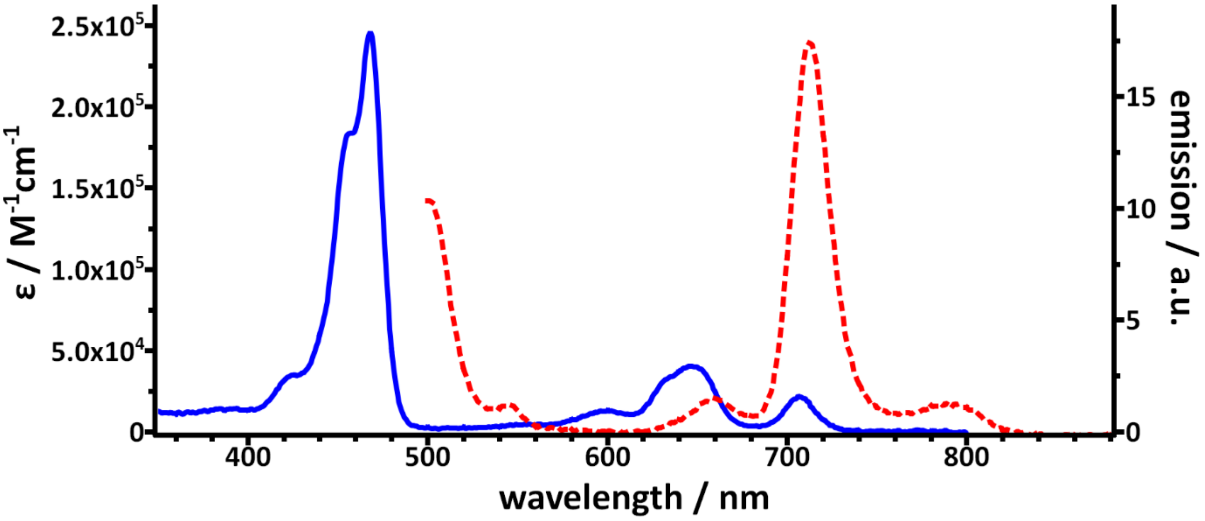

Figure S70. absorption (blue line) and emission spectrum of **14** (dashed red line; excitation at 468 nm) measured in CH_2Cl_2 + 1% NEt_3 at rt.

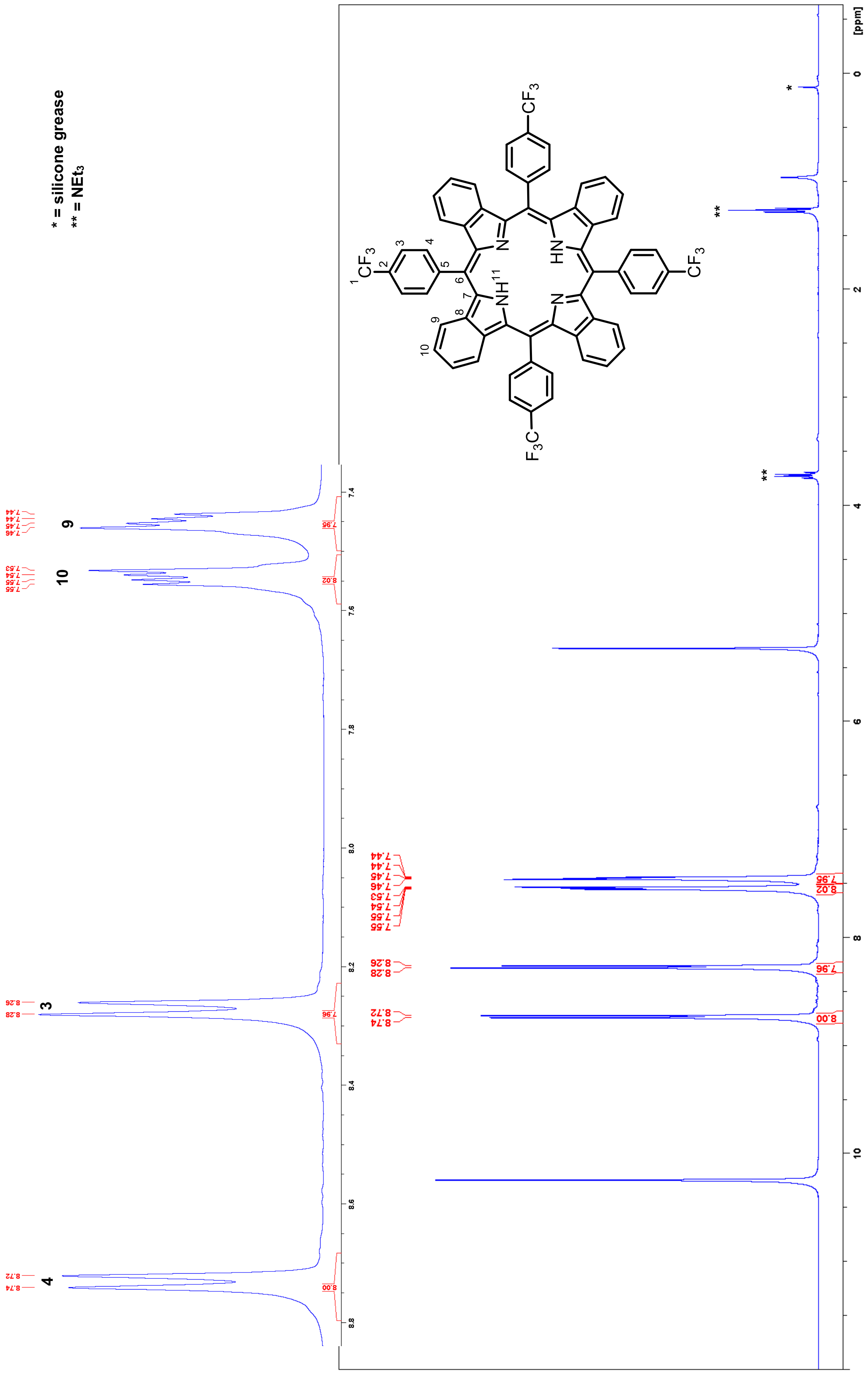

Figure S71. ^{1}H NMR (400 MHz, CD_2Cl_2/TFA-d_1, rt) of **15**.

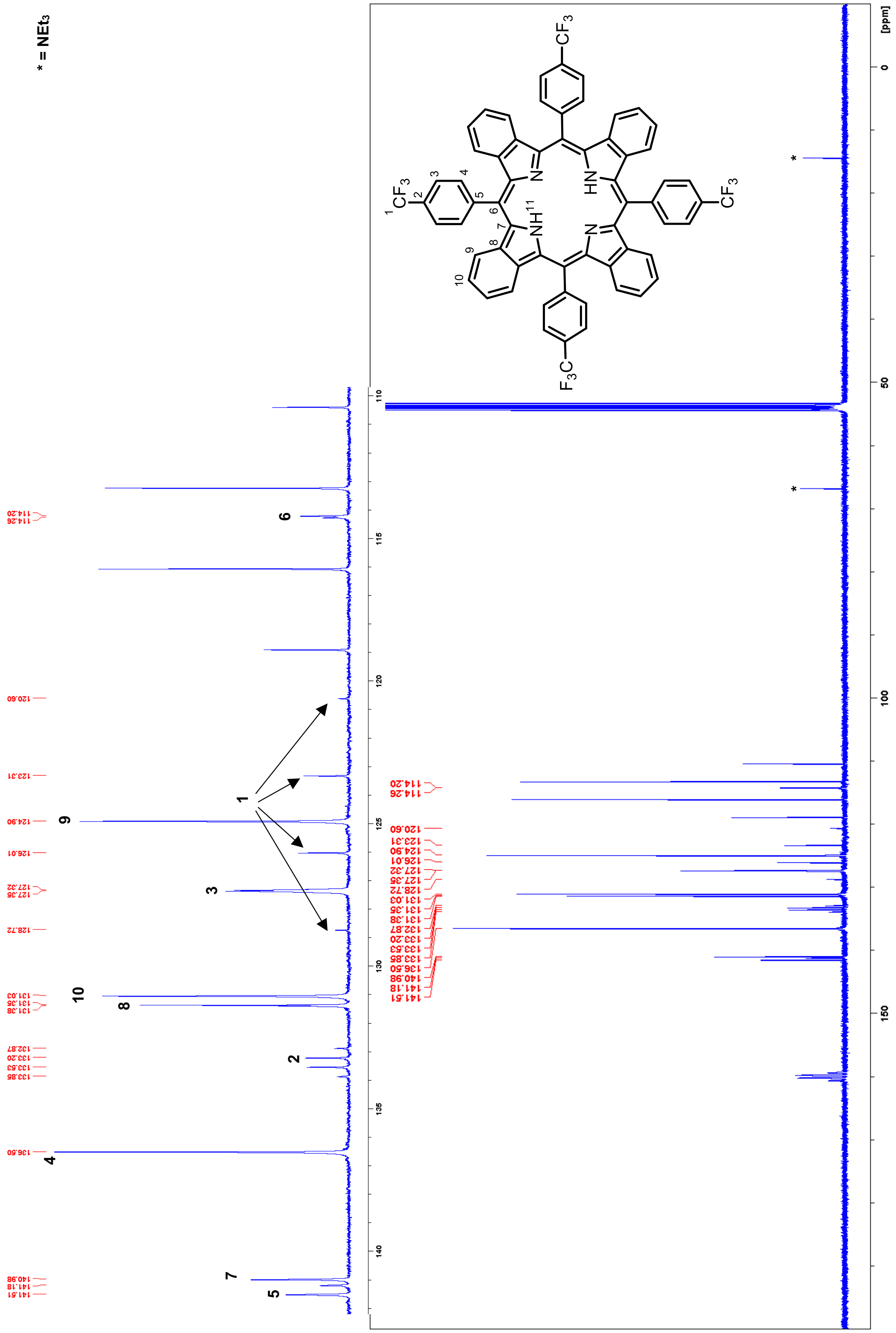

Figure S72. ^{13}C NMR (100 MHz, CD_2Cl_2/TFA-d_1, rt) of **15**.

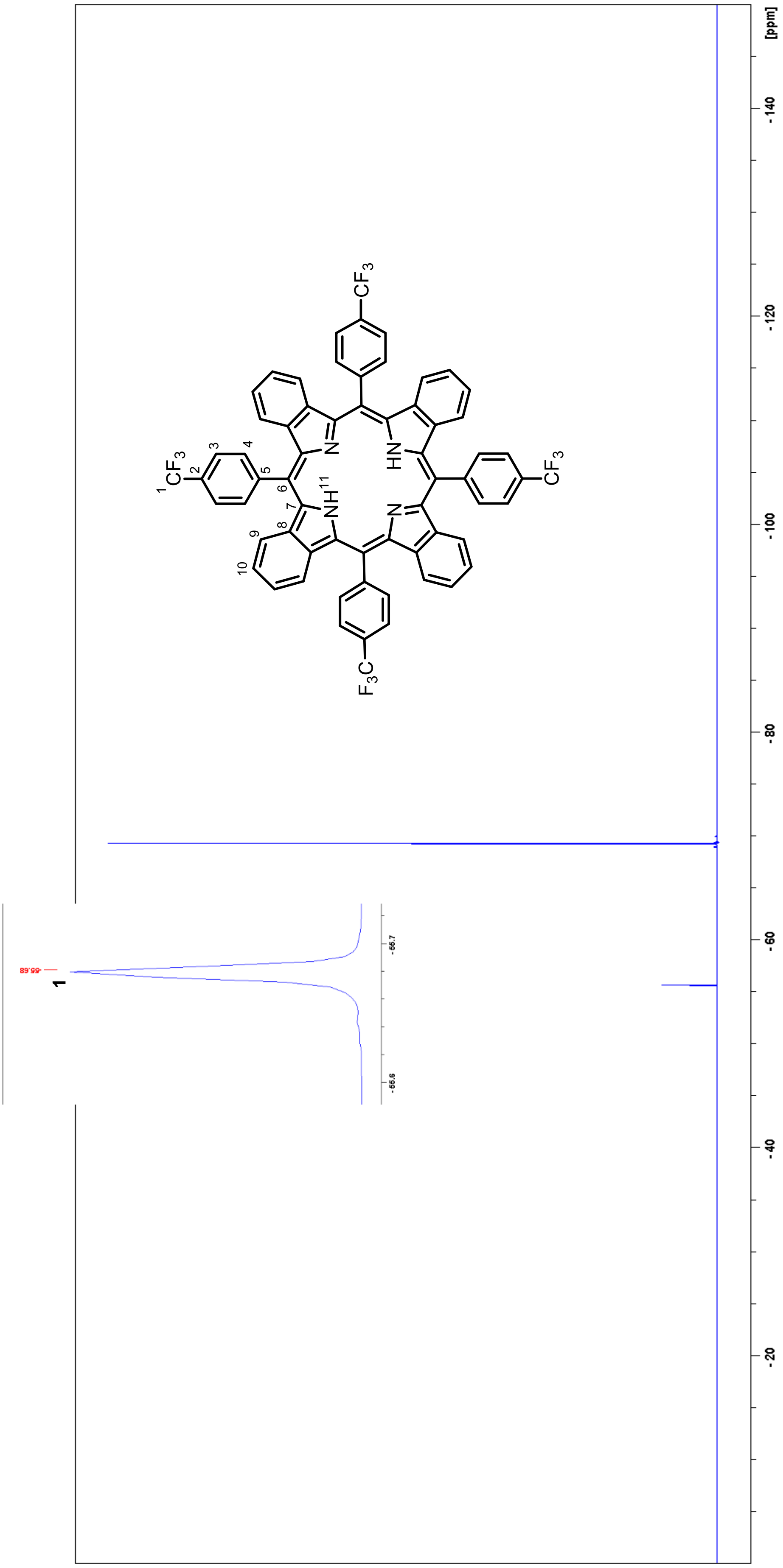

Figure S73. ^{19}F NMR (282 MHz, CD_2Cl_2/TFA-d_1, rt) of **15**.

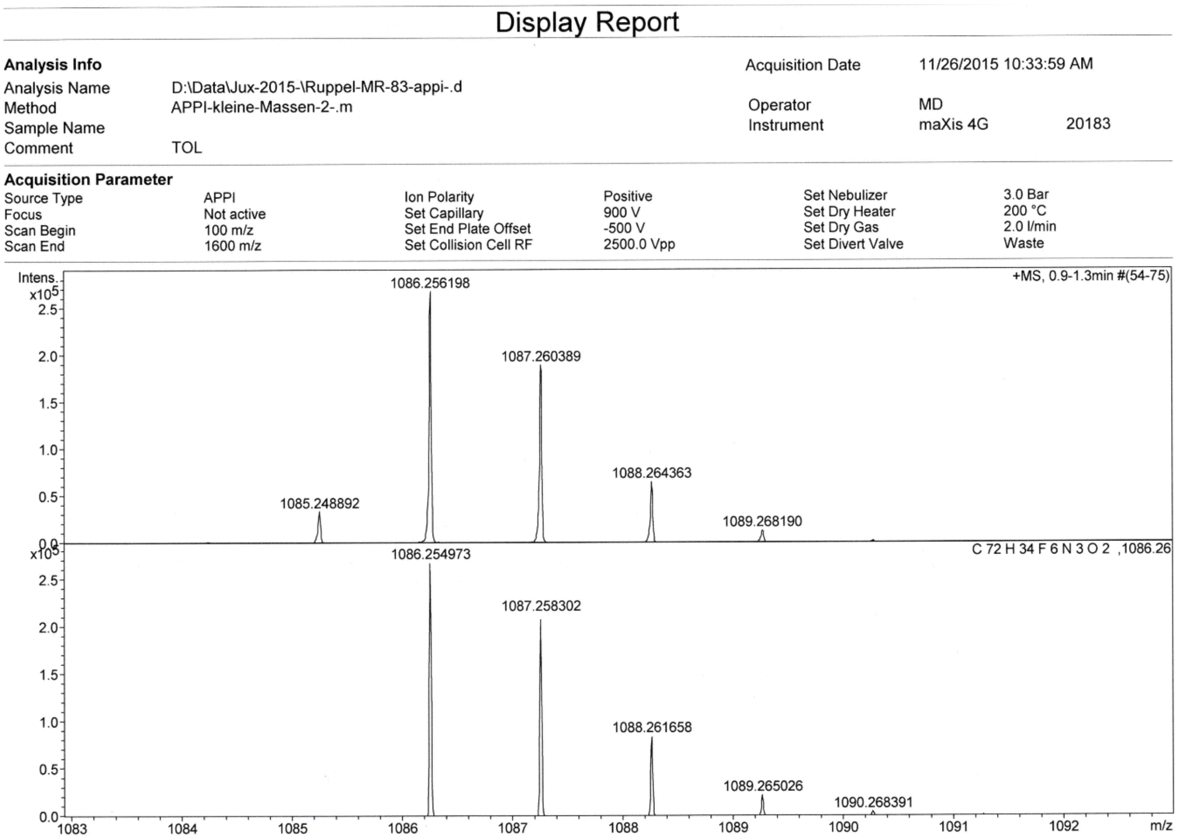

Figure S74. HRMS (APPI) of **15**.

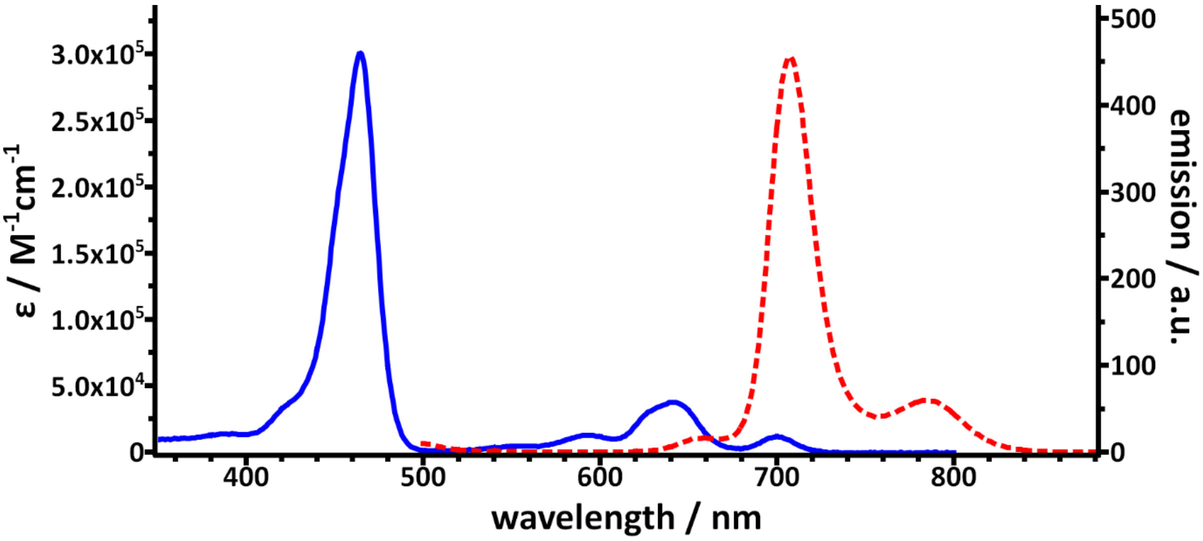

Figure S75. absorption (blue line) and emission spectrum of **15** (dashed red line; excitation at 464 nm) measured in CH_2Cl_2 + 1% NEt_3 at rt.

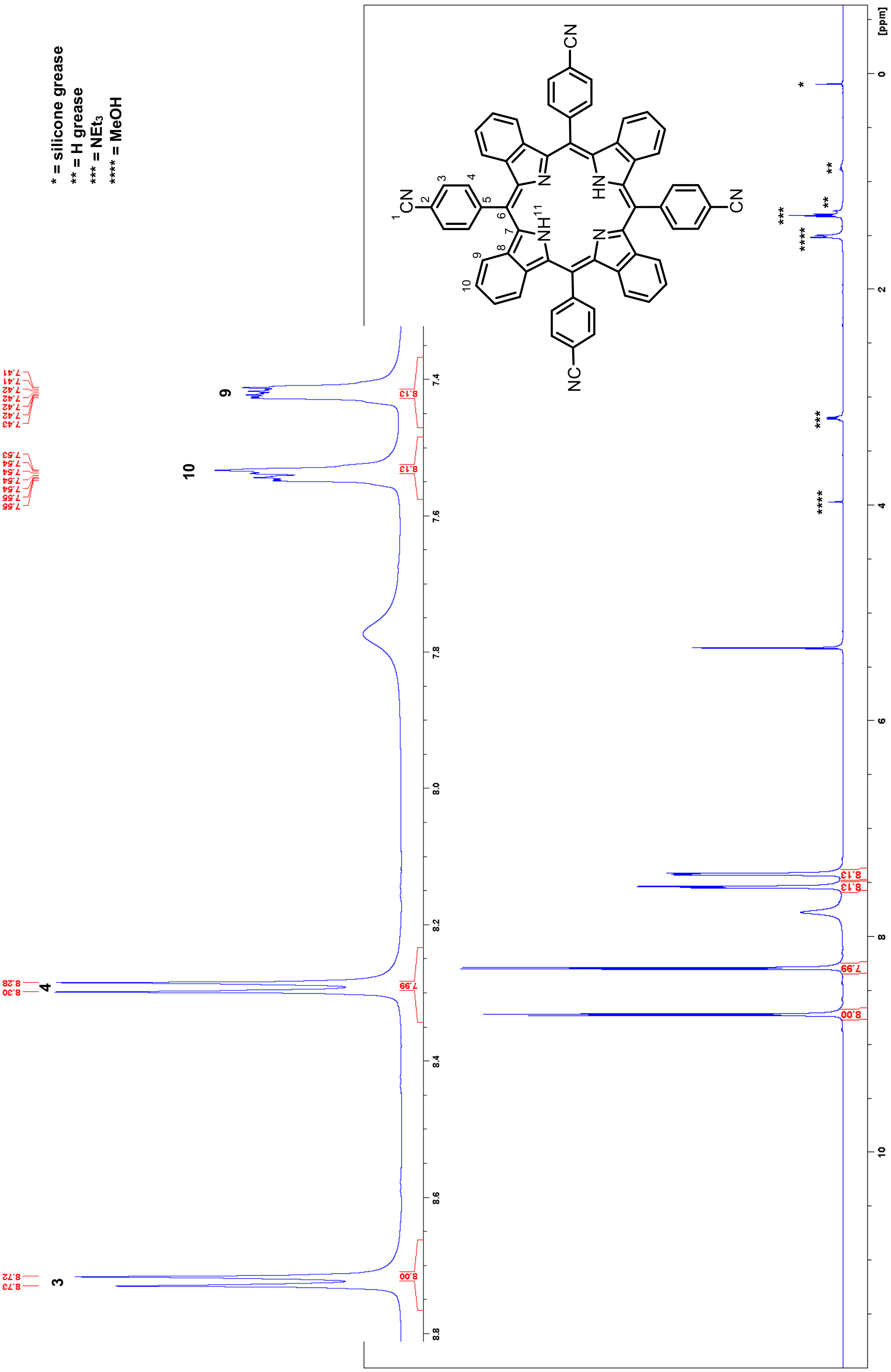

Figure S76. ^{1}H NMR (600 MHz, CD_2Cl_2/TFA-d_1, rt) of **16**.

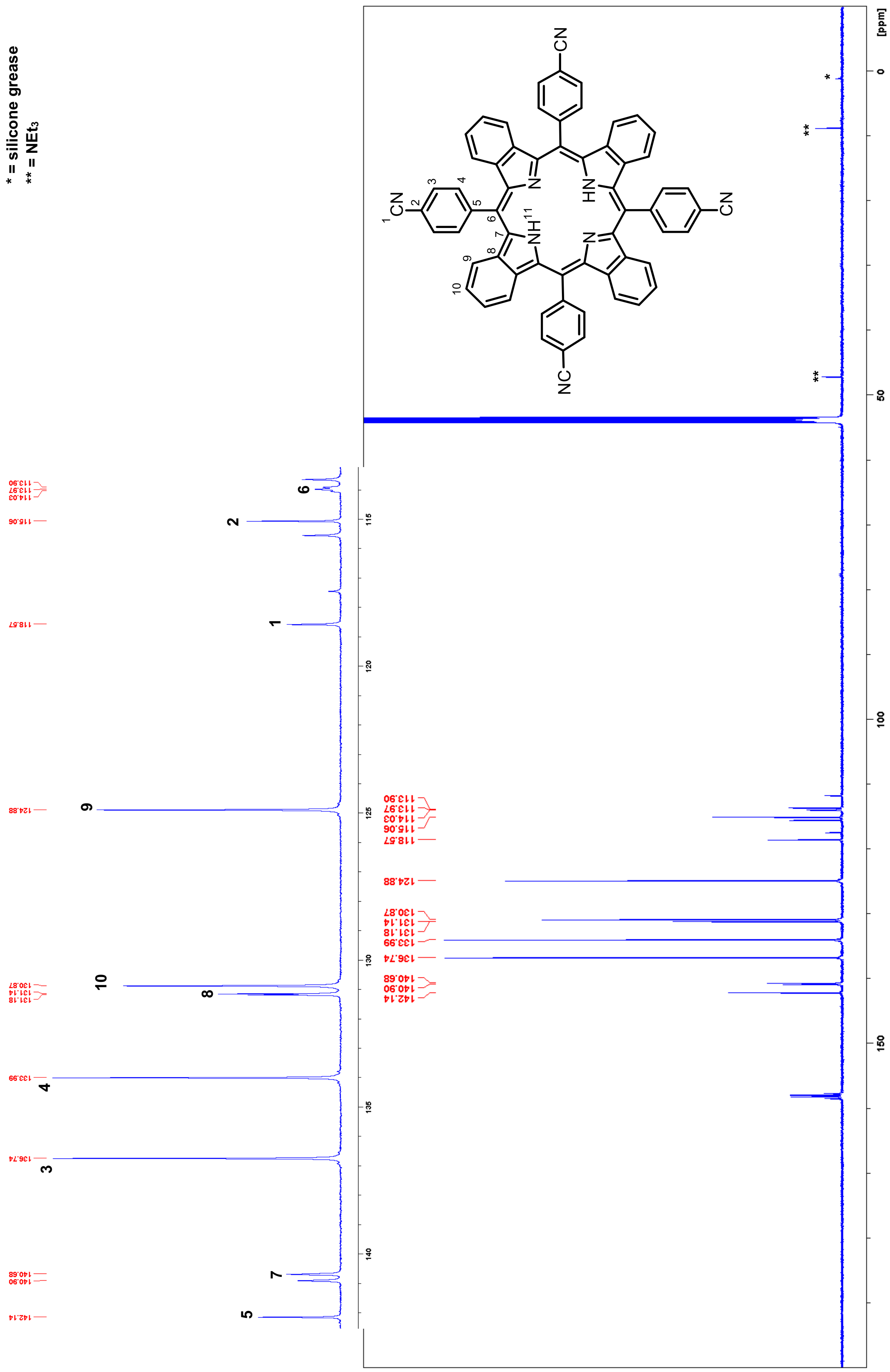

Figure S77. ^{13}C NMR (150 MHz, CD_2Cl_2/TFA-d_1, rt) of **16**.

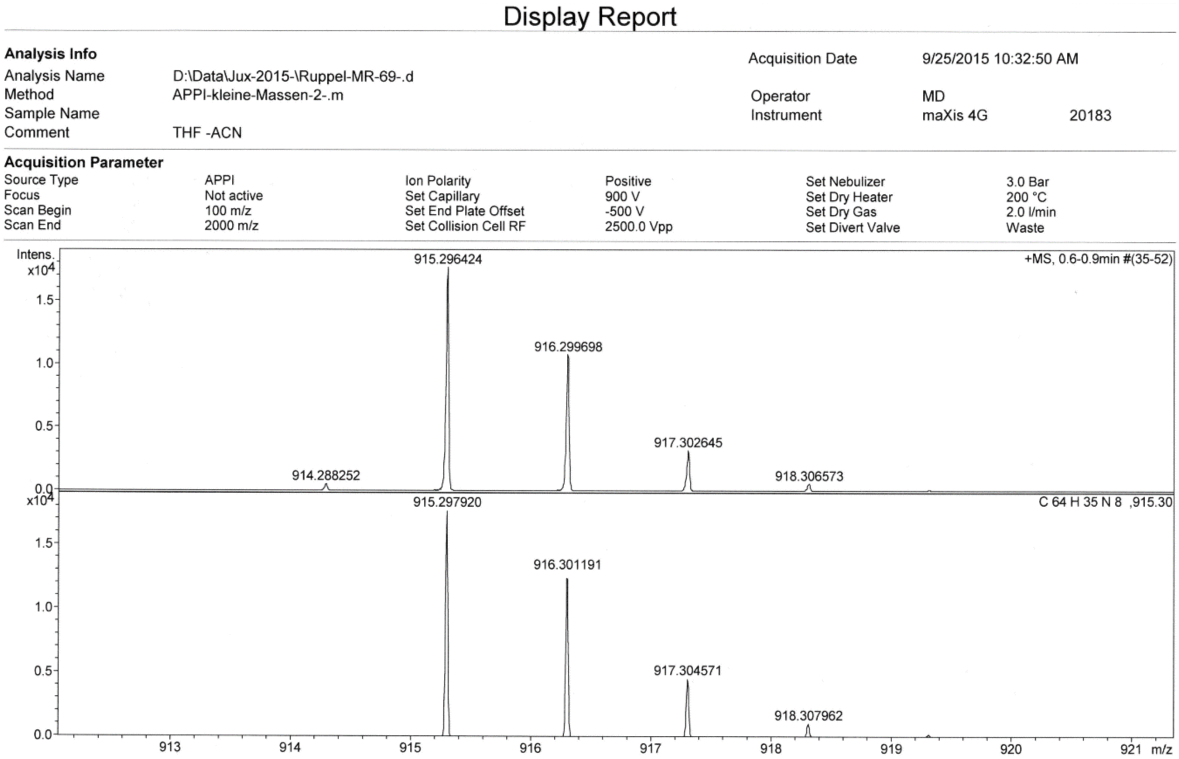

Figure S78. HRMS (APPI, THF/MeCN) of **16**.

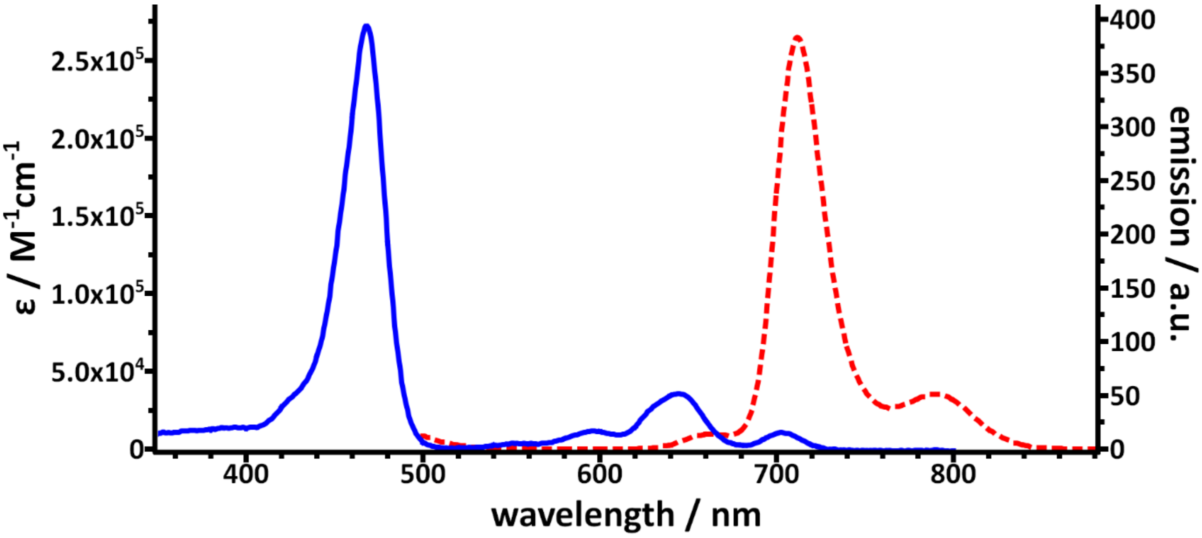

Figure S79. absorption (blue line) and emission spectrum of **16** (dashed red line; excitation at 468 nm) measured in CH_2Cl_2 + 1% NEt_3 at rt.

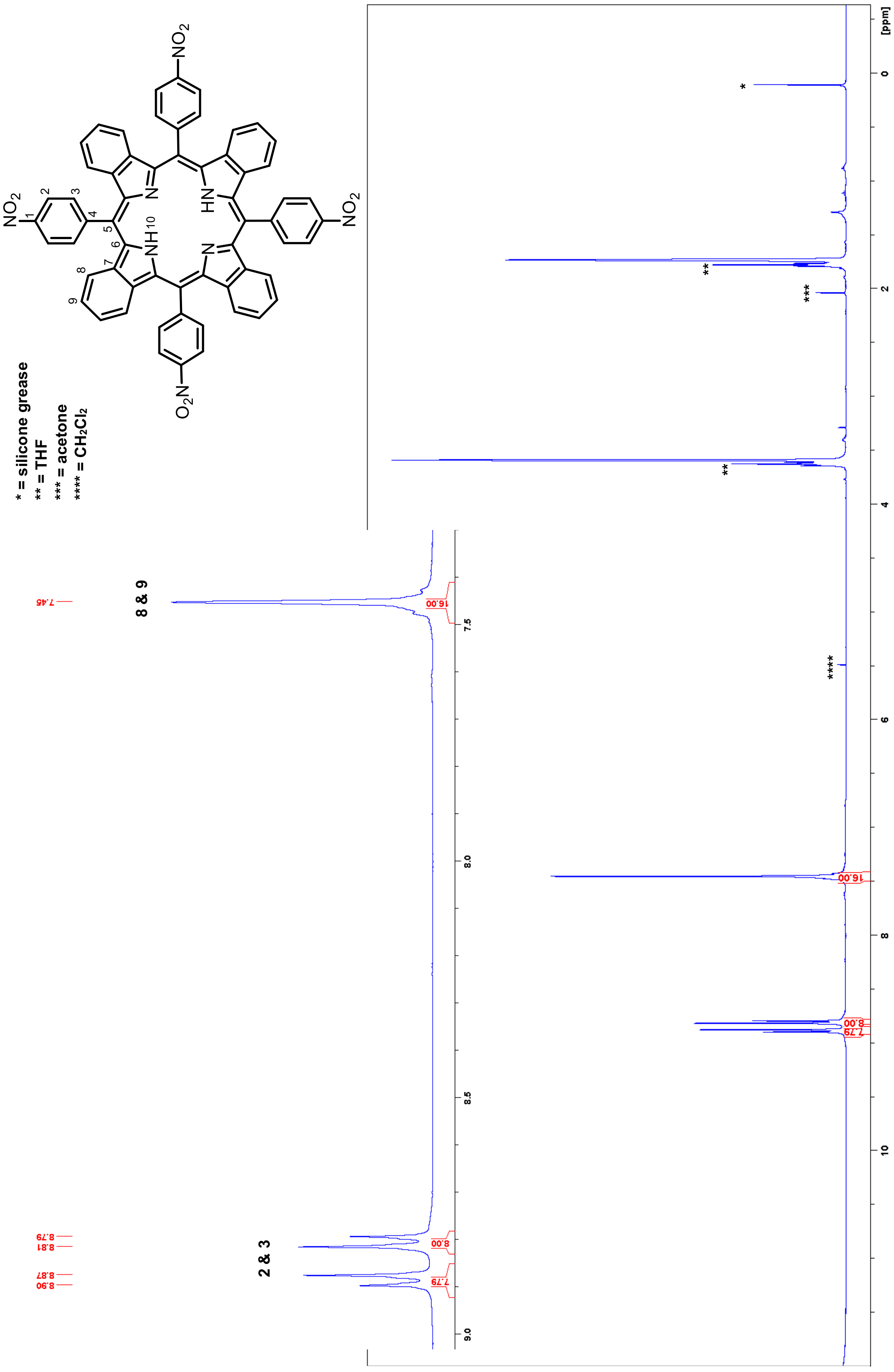

Figure S80. ^{1}H NMR (400 MHz, THF-d_8/TFA-d_1, rt) of **17**.

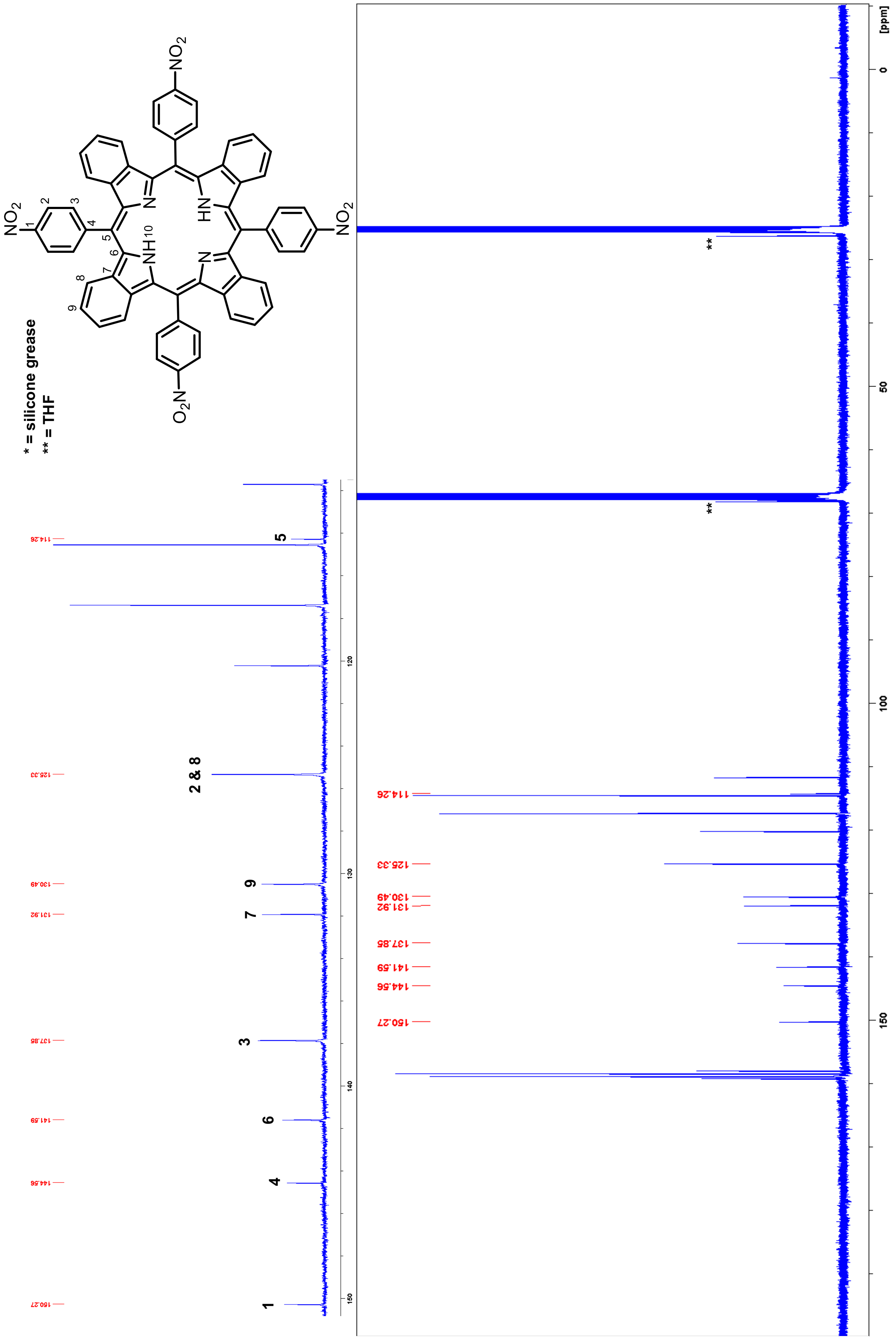

Figure S81. ^{13}C NMR (100 MHz, THF-d_8/TFA-d_1, rt) of **17**.

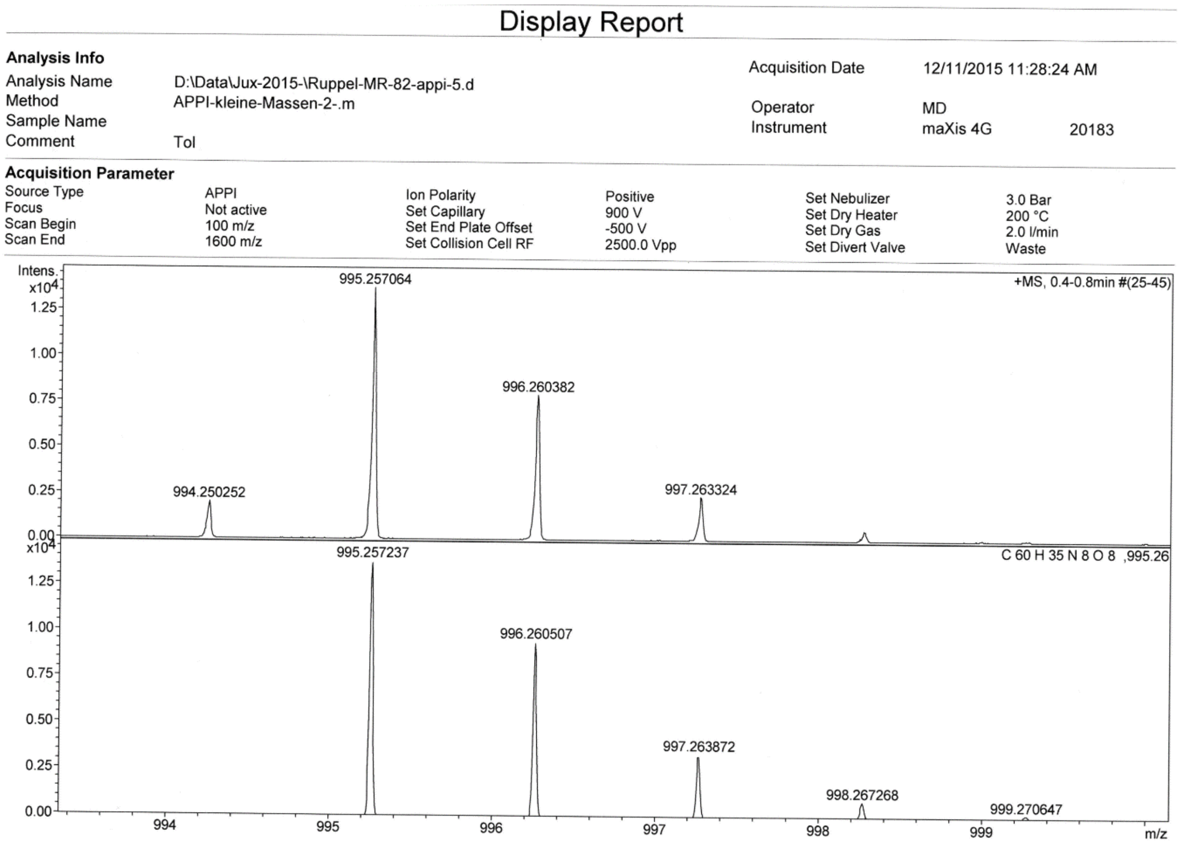

Figure S82. HRMS (APPI, toluene) of **17**.

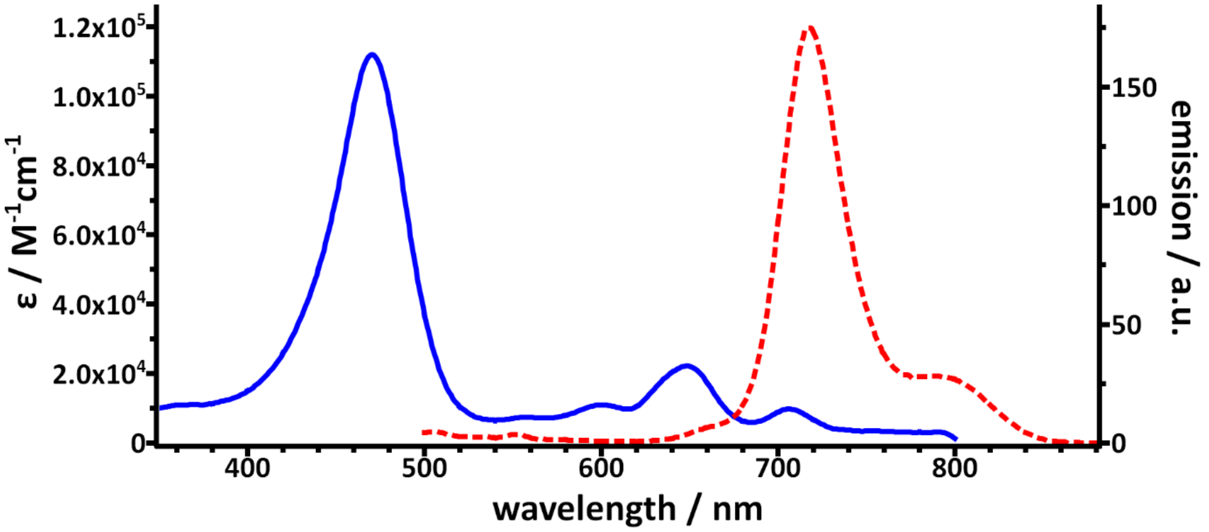

Figure S83. absorption (blue line) and emission spectrum of **17** (dashed red line; excitation at 470 nm) measured in CH_2Cl_2 + 1% NEt_3 at rt.

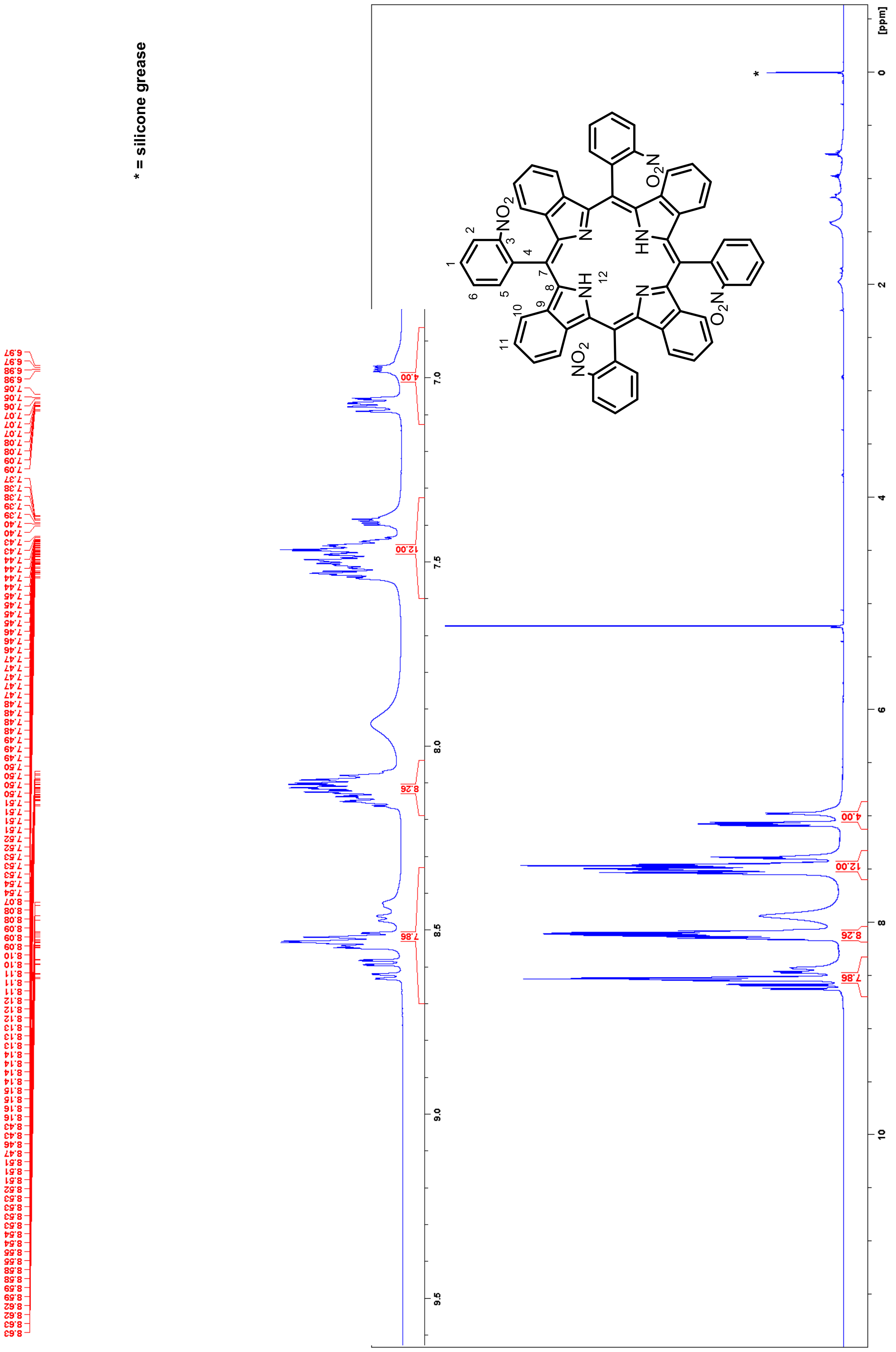

Figure S84. ^{1}H NMR (600 MHz, CD_2Cl_2/TFA-d_1, rt) of **18**.

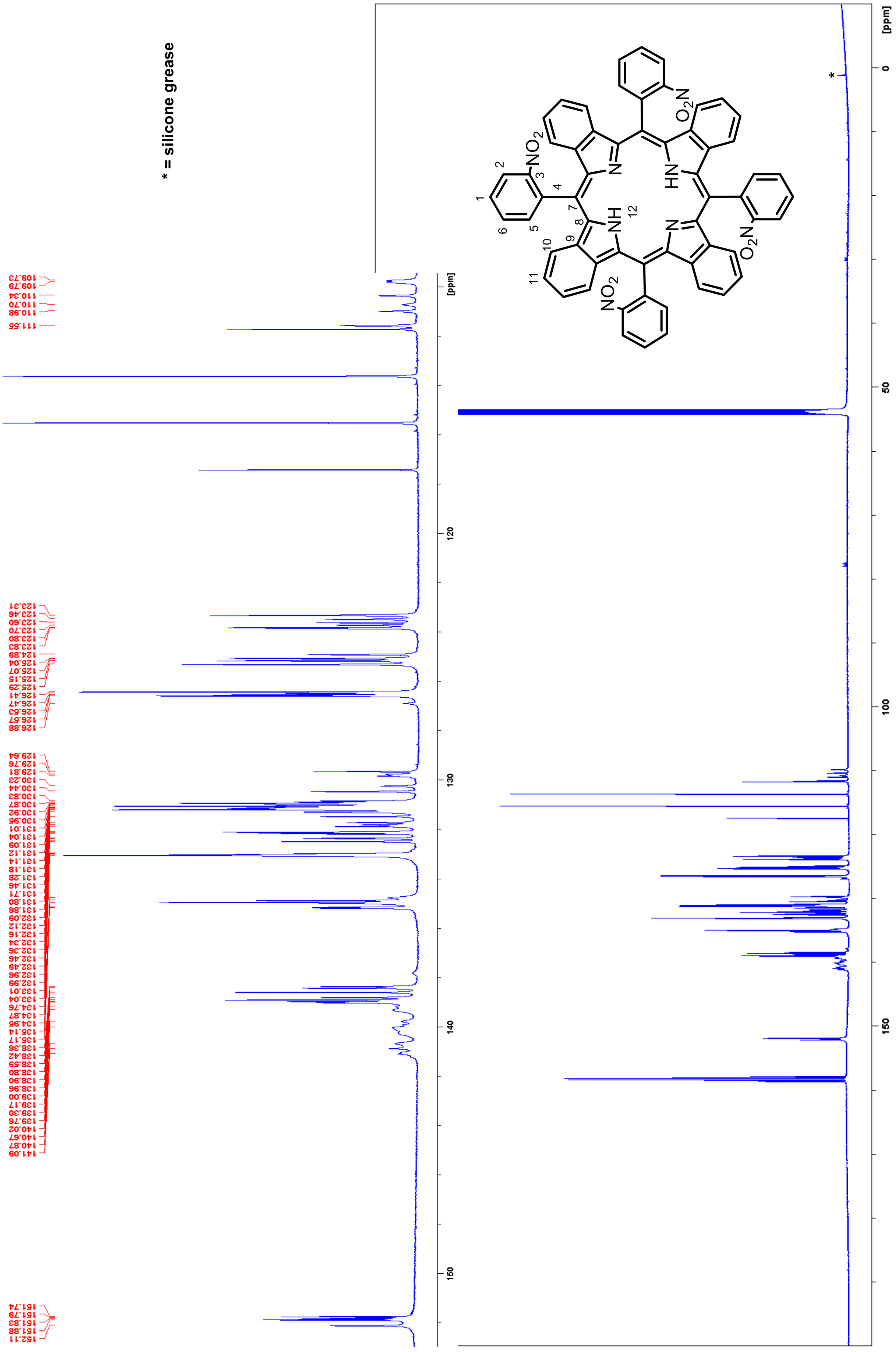

Figure S85. ^{13}C NMR (150 MHz, CD_2Cl_2/TFA-d_1, rt) of **18**.

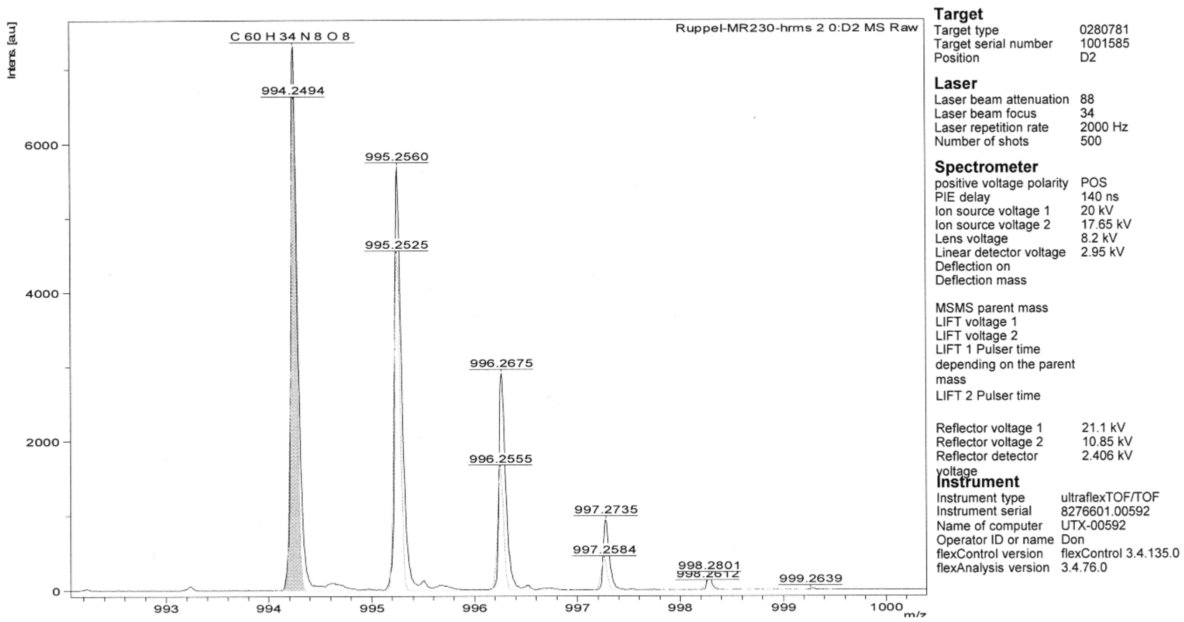

Figure S86. HRMS (MALDI) of **18**.

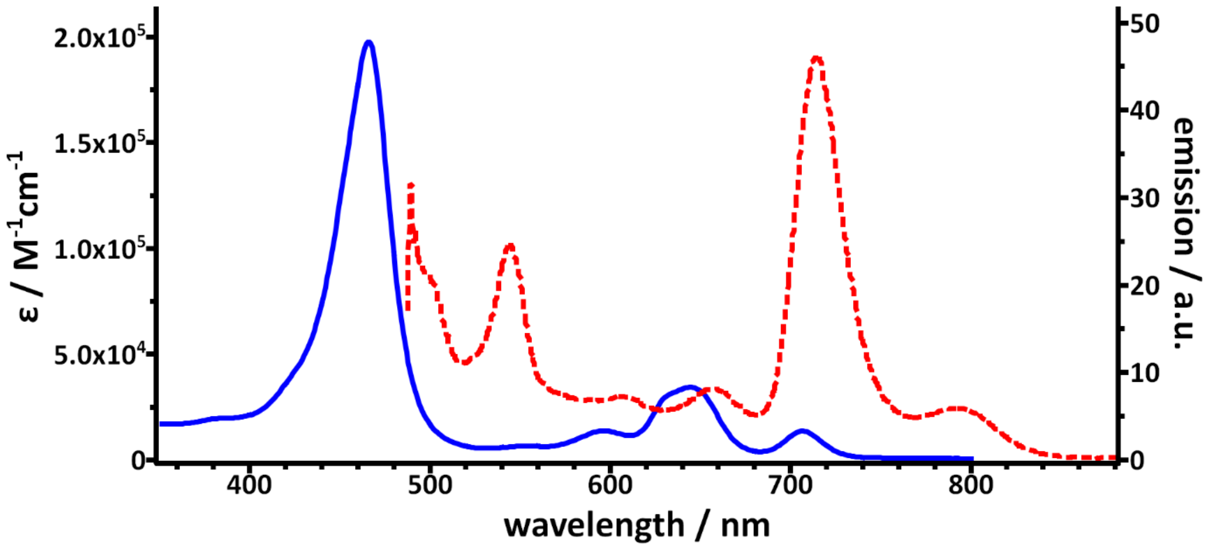

Figure S87. absorption (blue line) and emission spectrum of **18** (dashed red line; excitation at 466 nm) measured in CH_2Cl_2 + 1% NEt_3 at rt.

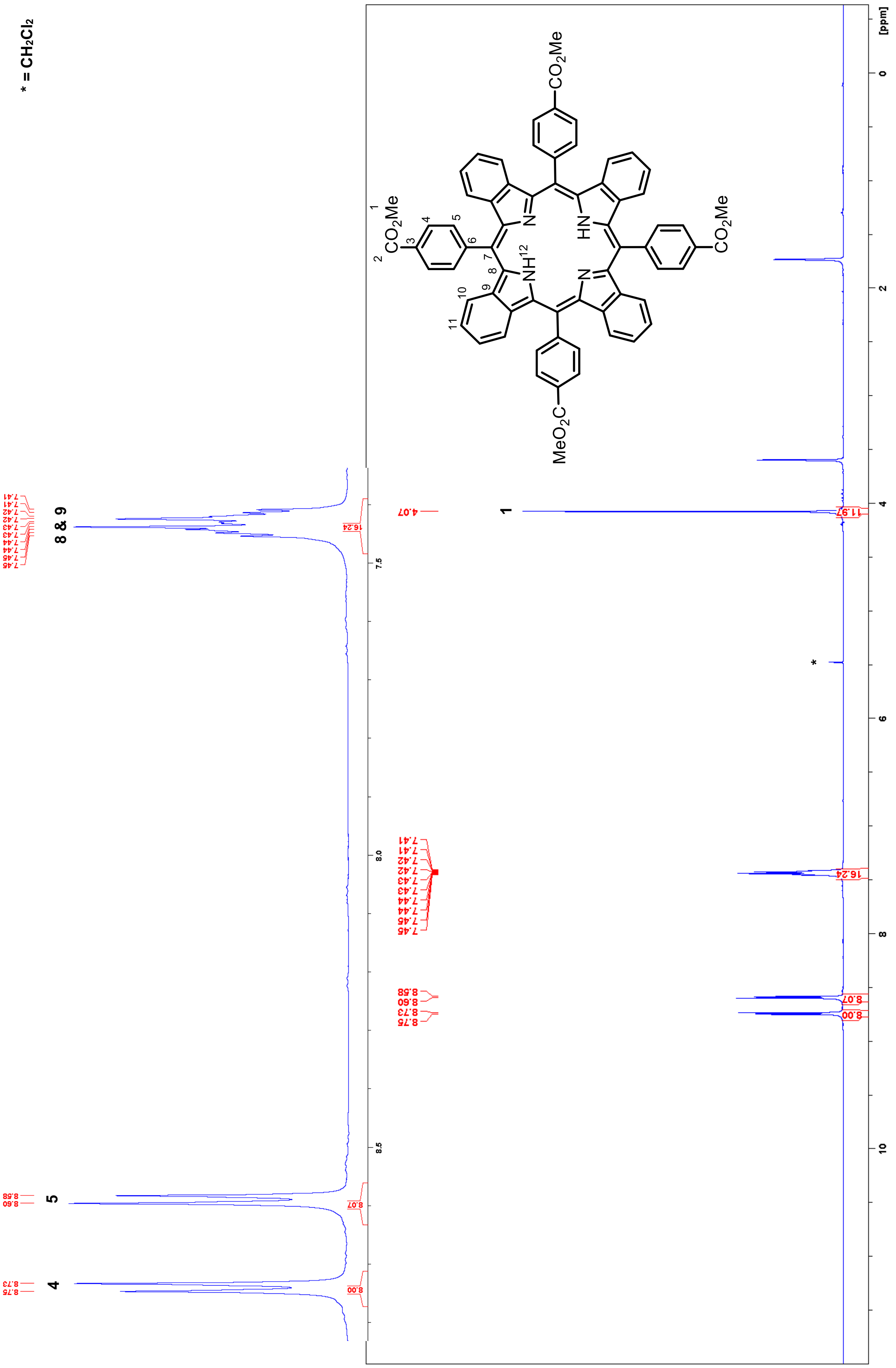

Figure S88. ^{1}H NMR (600 MHz, THF-d_8/TFA-d_1, rt) of **19**.

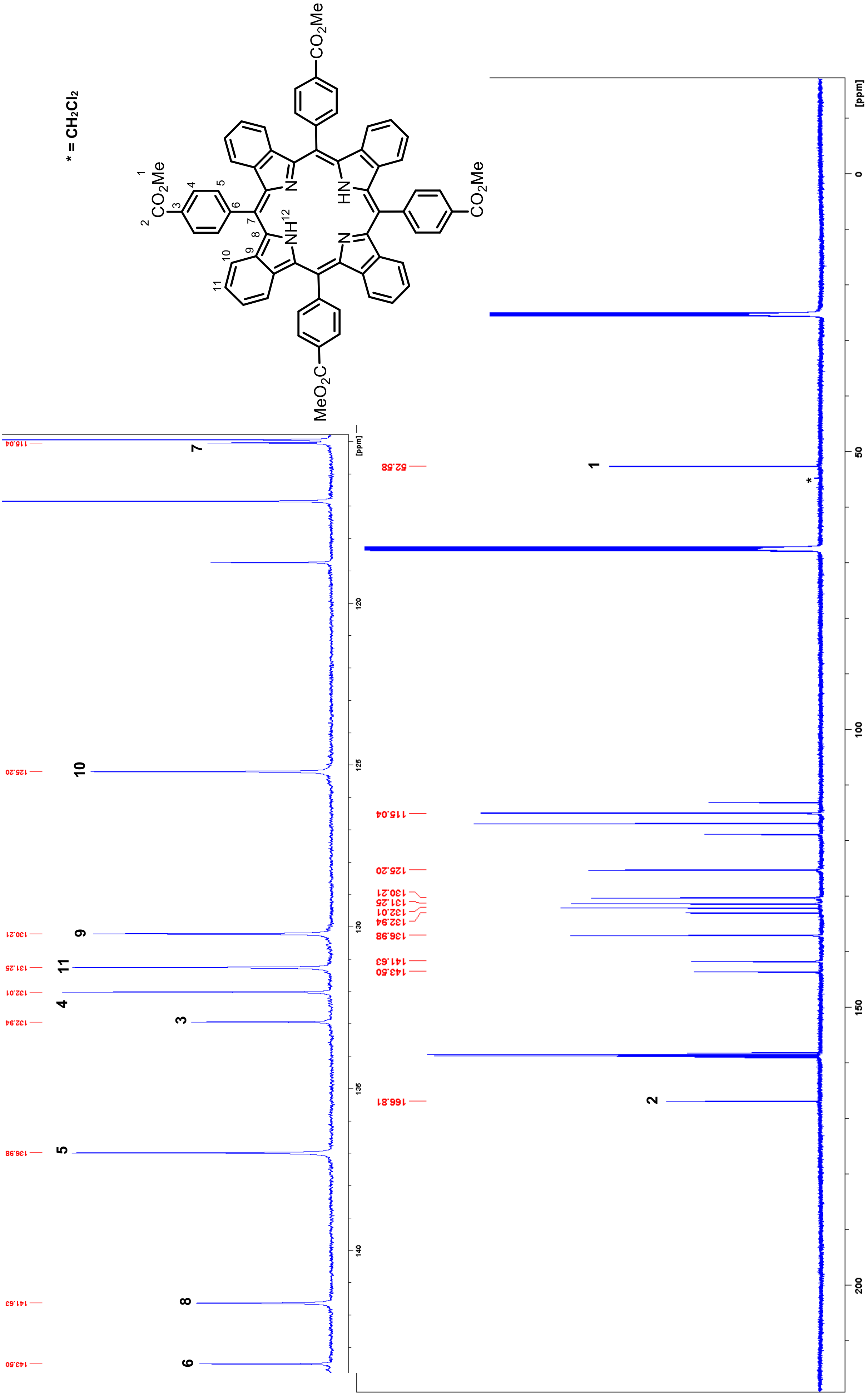

Figure S89. ^{13}C NMR (150 MHz, THF-d_8/TFA-d_1, rt) of **19**.

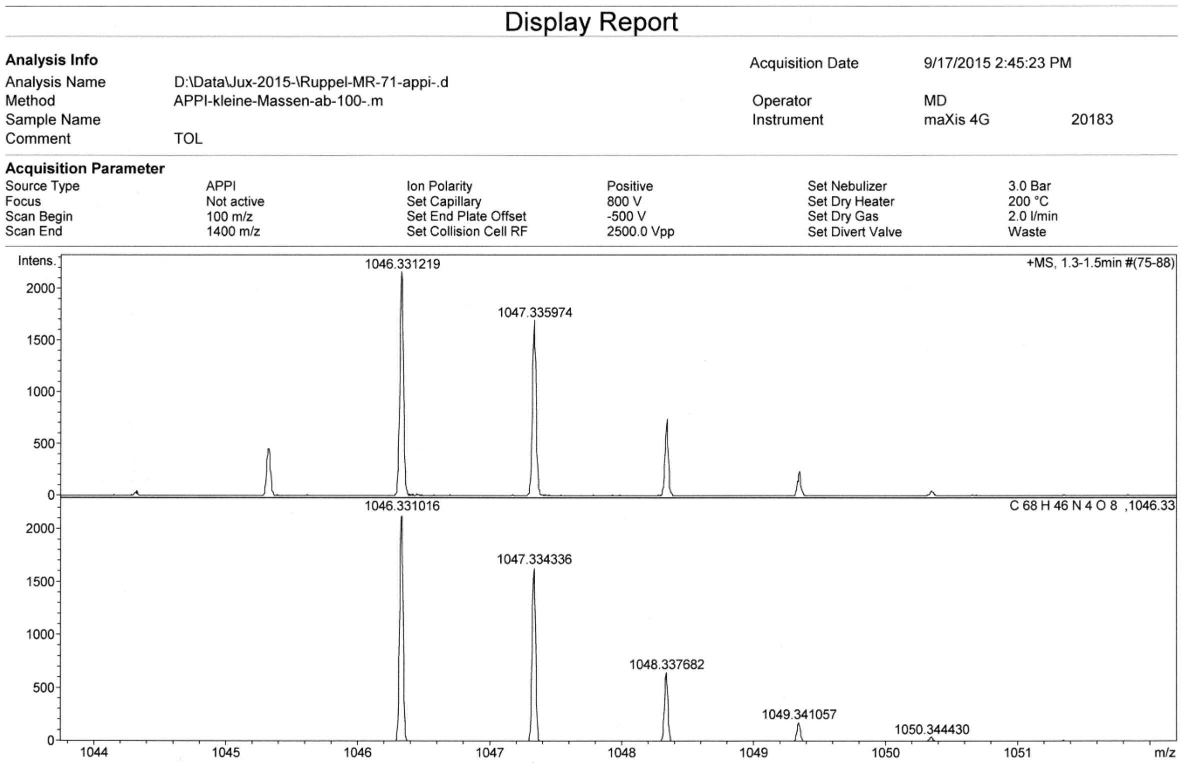

Figure S90. HRMS (APPI, toluene) of **19**.

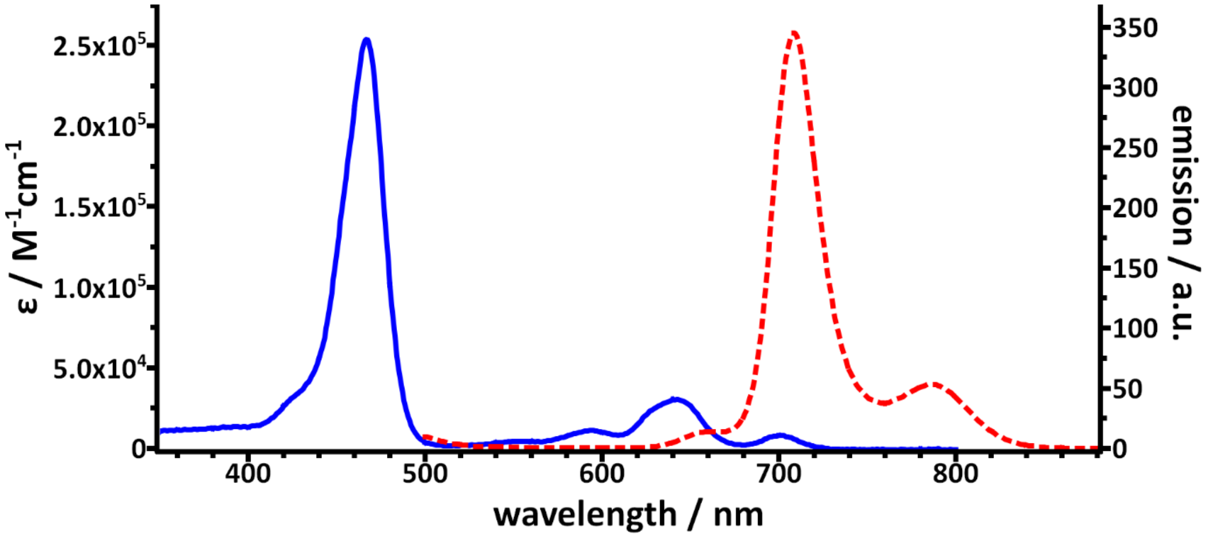

Figure S91. absorption (blue line) and emission spectrum of **19** (dashed red line; excitation at 467 nm) measured in CH_2Cl_2 + 1% NEt_3 at rt.

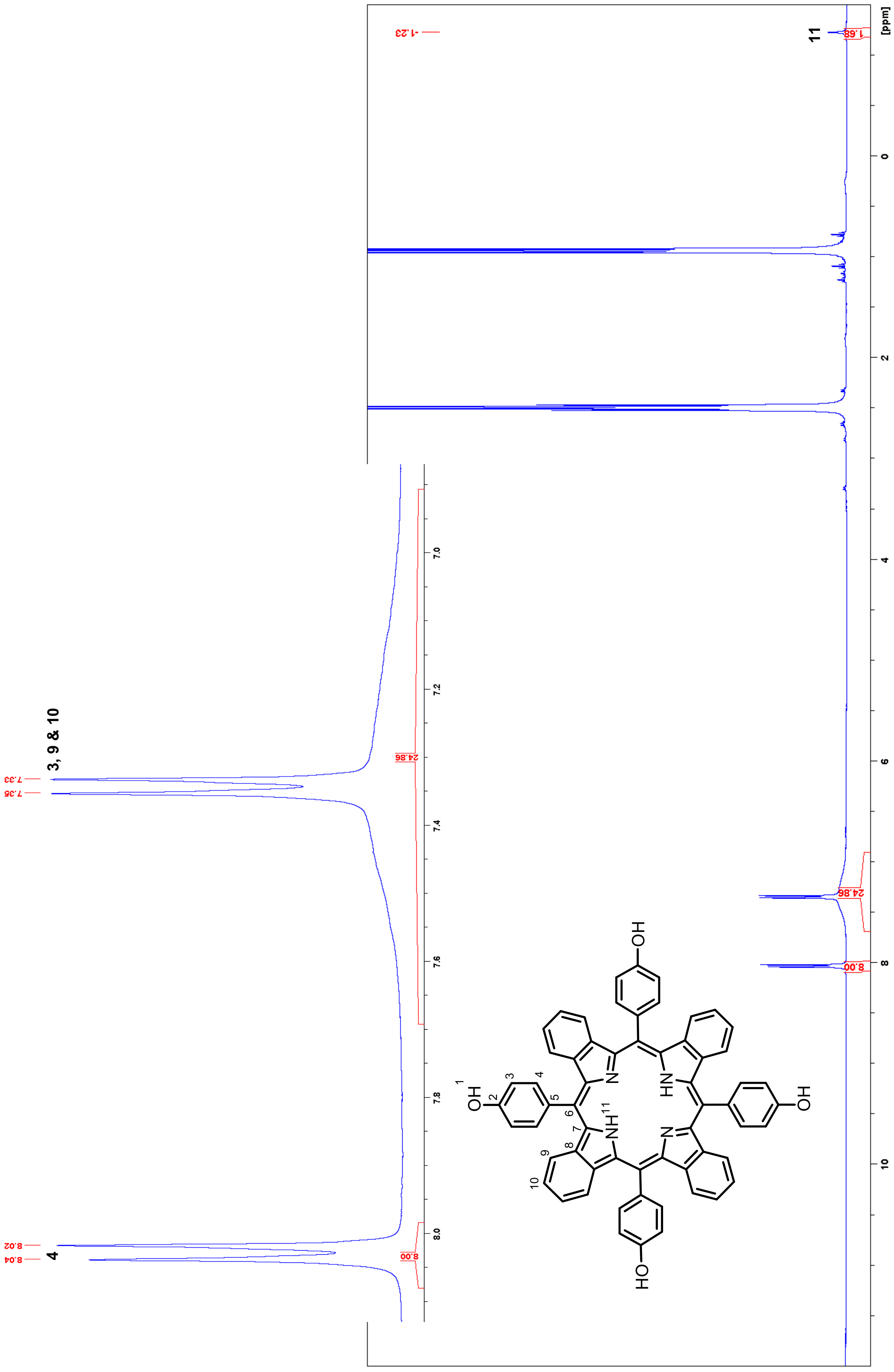

Figure S92. ^{1}H NMR (400 MHz, DMSO-d$_6$/NEt$_3$, rt) of **20**.

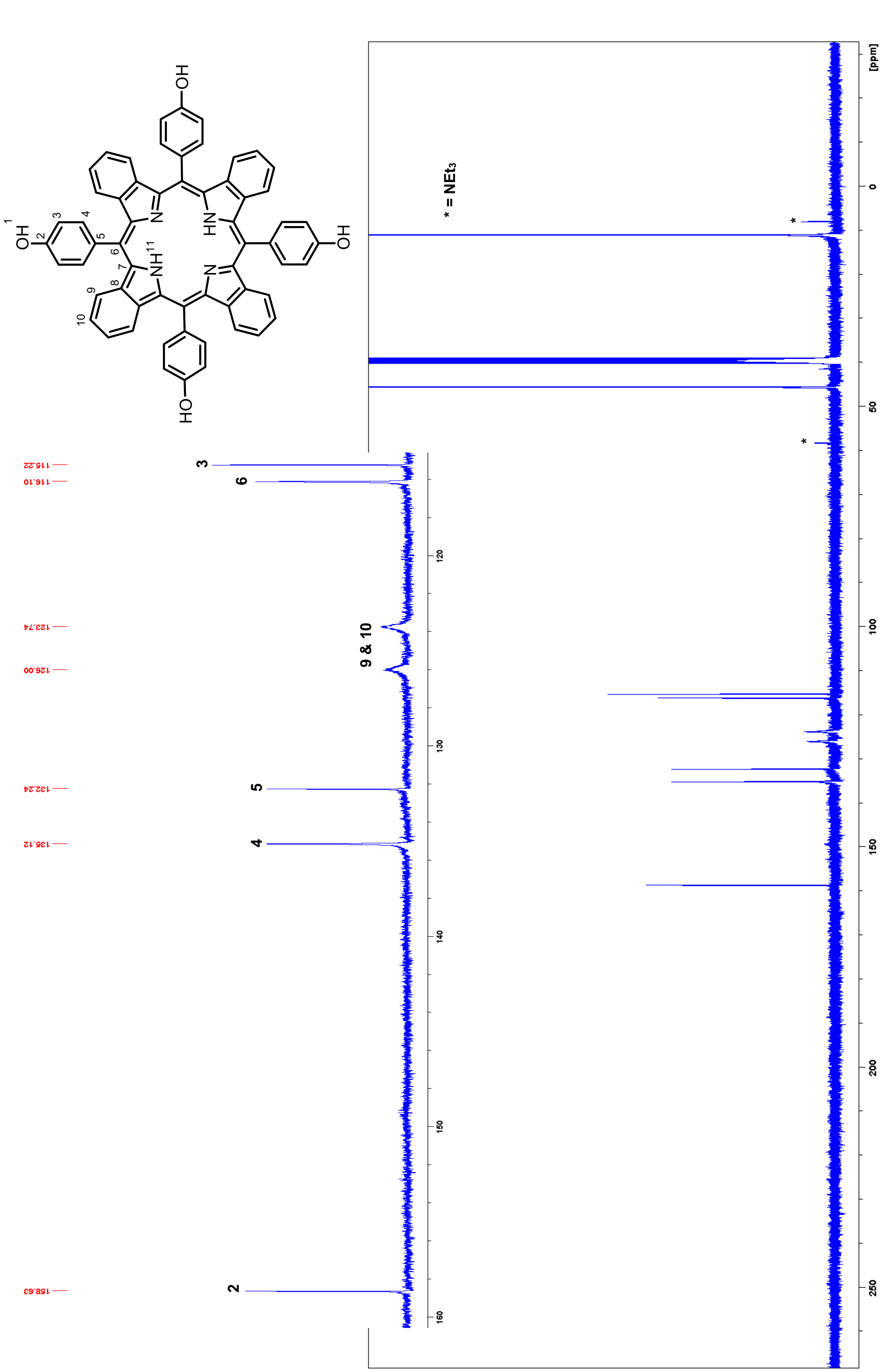

Figure S93. ^{13}C NMR (100 MHz, DMSO-d_6/NEt_3, rt) of **20**.

Display Report

Analysis Info

Analysis Name	D:\Data\Jux-2017-\Ruppel-MR-79-2appi-000001.d	Acquisition Date	1/10/2017 1:55:35 PM
Method	tune_mid_pos_APPI.m	Operator	MD
Sample Name		Instrument	maXis 288882.20183
Comment	Tol DMSO		

Acquisition Parameter

Source Type	APPI	Ion Polarity	Positive	Set Nebulizer	2.5 Bar
Focus	Not active	Set Capillary	800 V	Set Dry Heater	200 °C
Scan Begin	300 m/z	Set End Plate Offset	-500 V	Set Dry Gas	1.5 l/min
Scan End	2900 m/z	Set Charging Voltage	0 V	Set Divert Valve	Waste
		Set Corona	0 nA	Set APCI Heater	400 °C

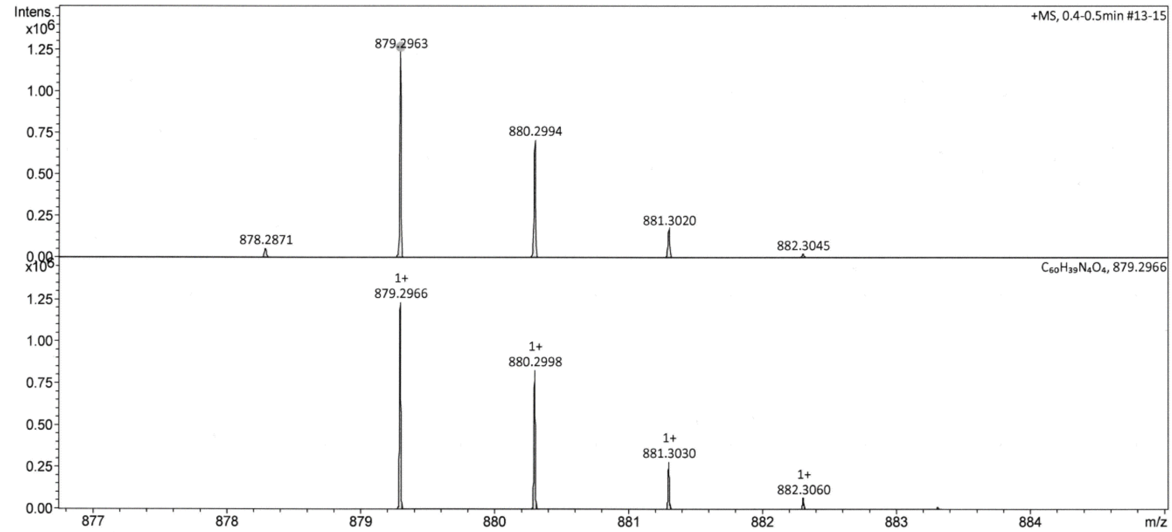

Figure S94. HRMS (APPI, toluene/DMSO)) of **20**.

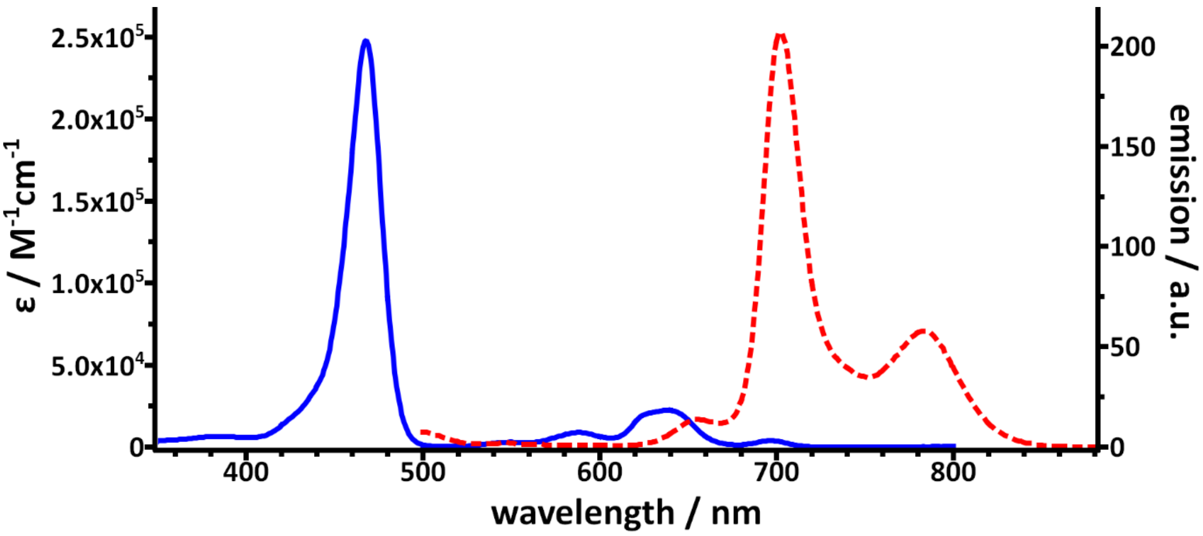

Figure S95. absorption (blue line) and emission spectrum of **20** (dashed red line; excitation at 468 nm) measured in CH_2Cl_2 + 1% NEt_3 at rt.

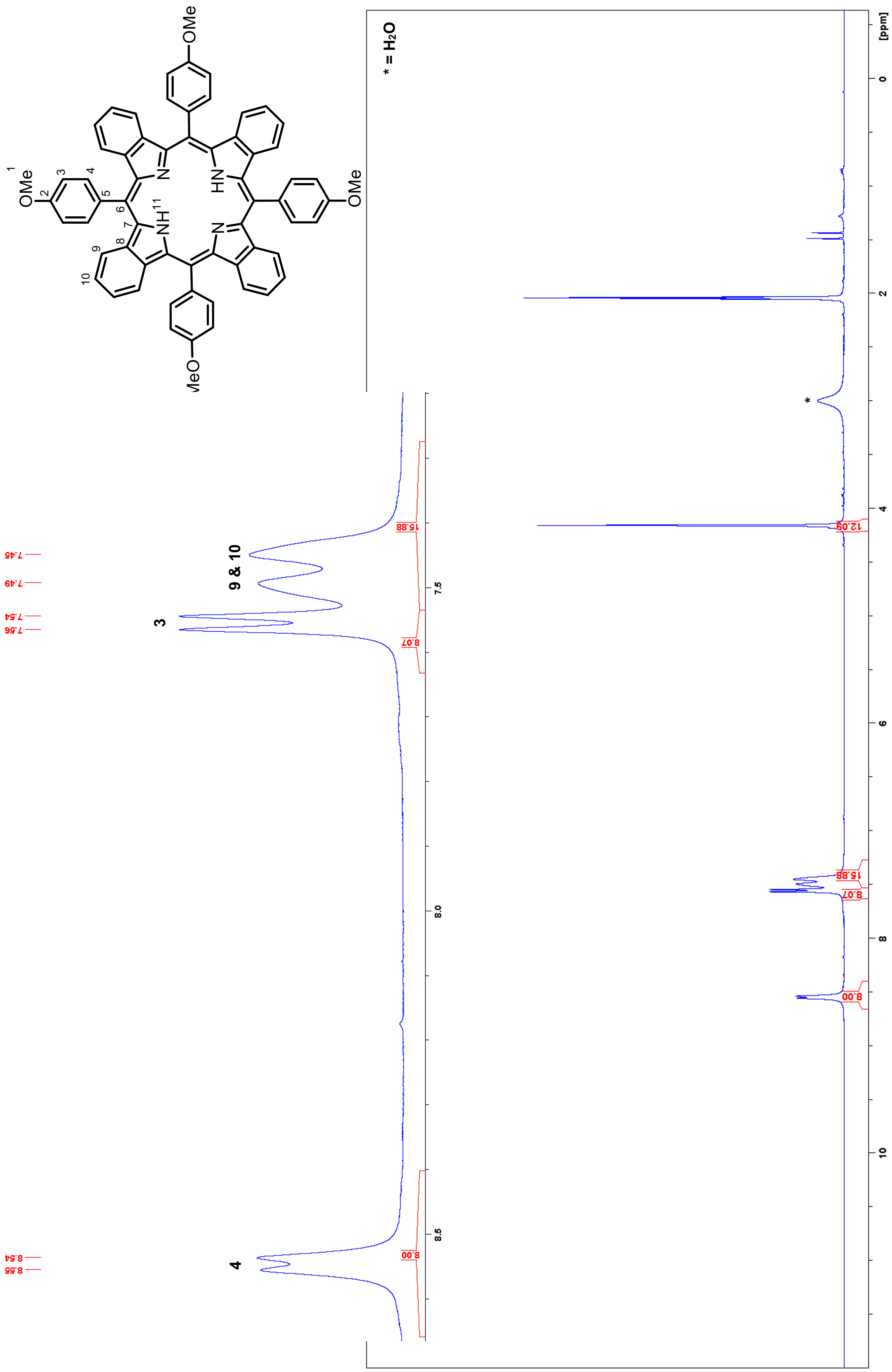

Figure S96. ^{1}H NMR (400 MHz, acetone-d_6, rt) of **21**.

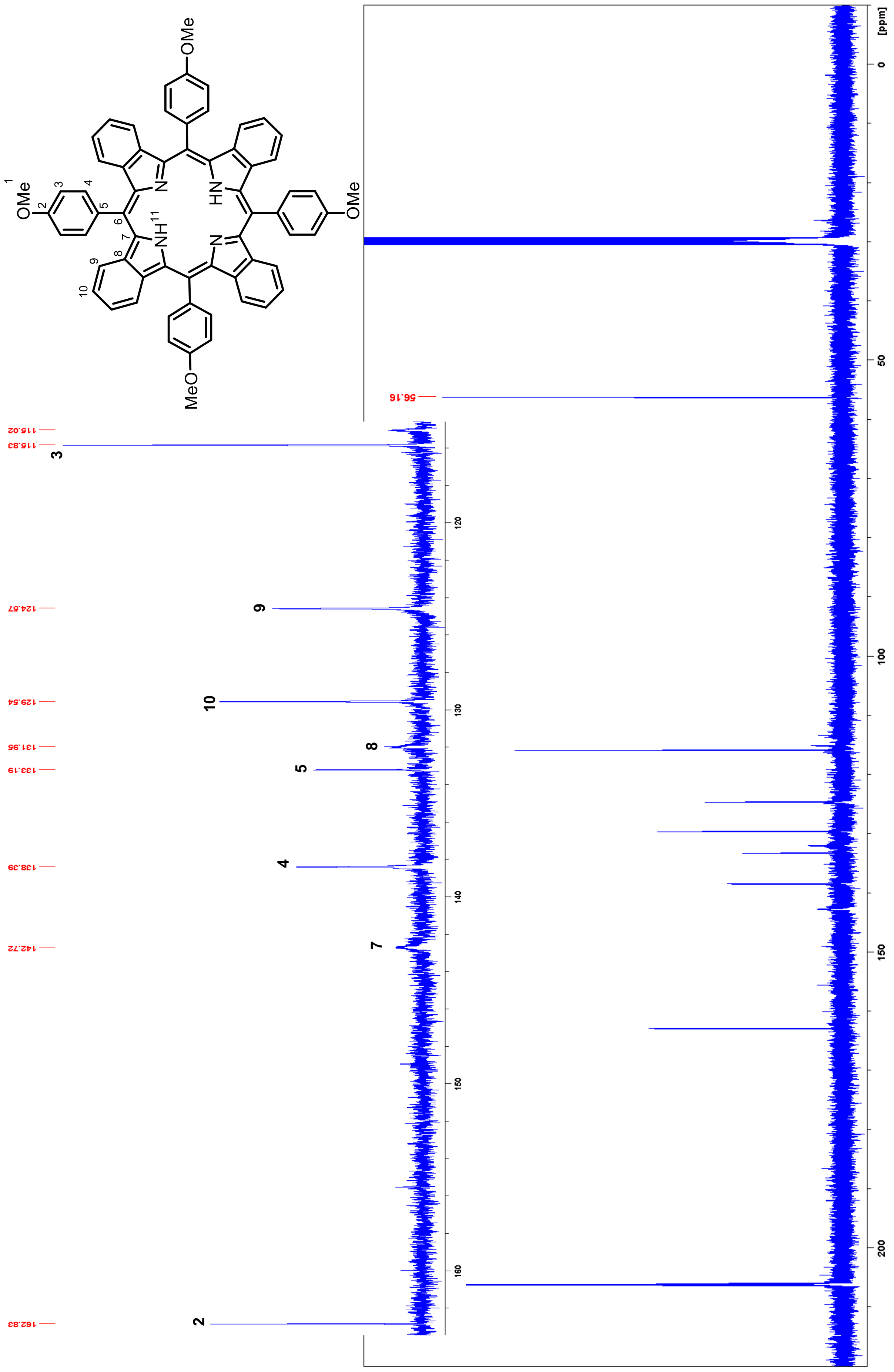

Figure S97. ^{13}C NMR (100 MHz, acetone-d_6, rt) of **21**.

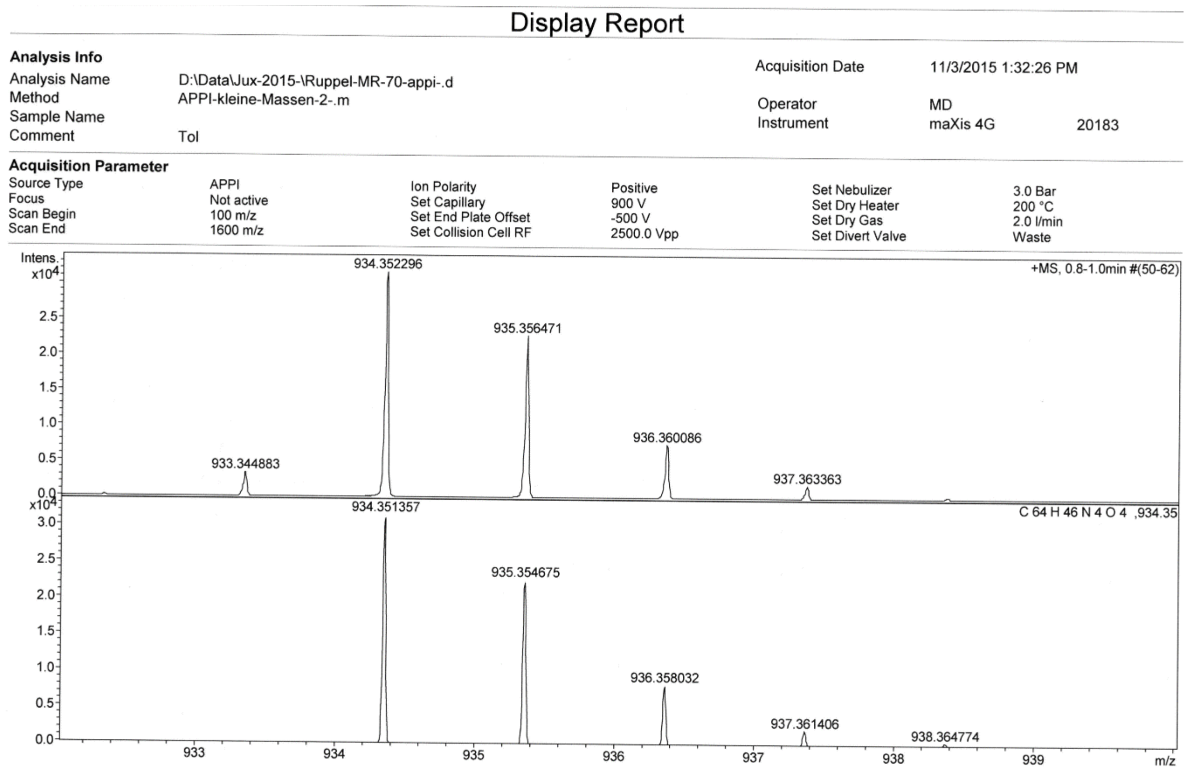

Figure S98. HRMS (APPI) of **21**.

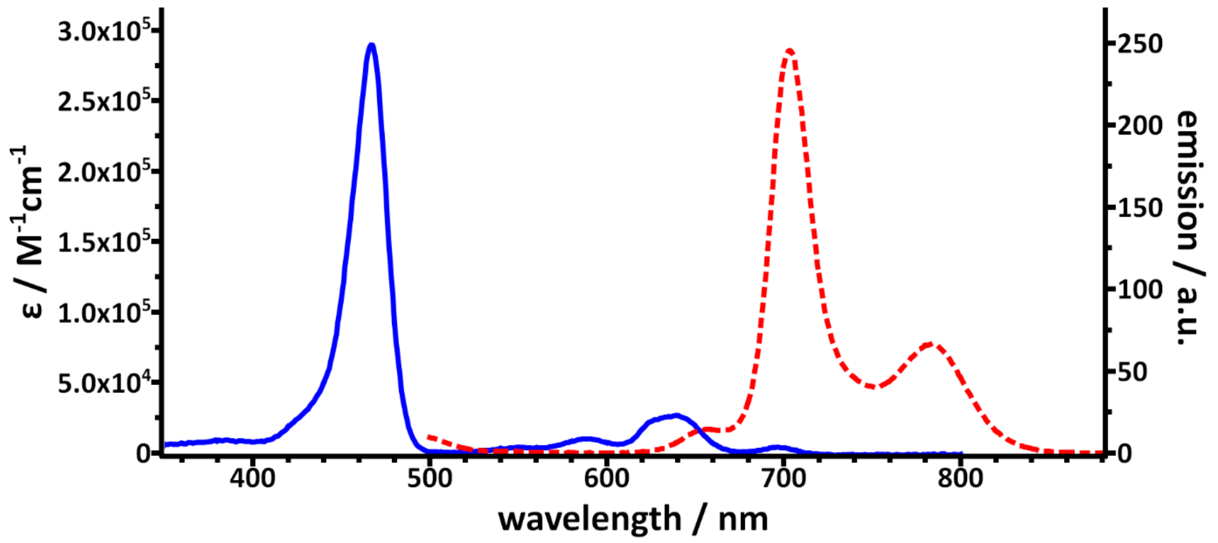

Figure S99. absorption (blue line) and emission spectrum of **21** (dashed red line; excitation at 467 nm) measured in CH_2Cl_2 + 1% NEt_3 at rt.

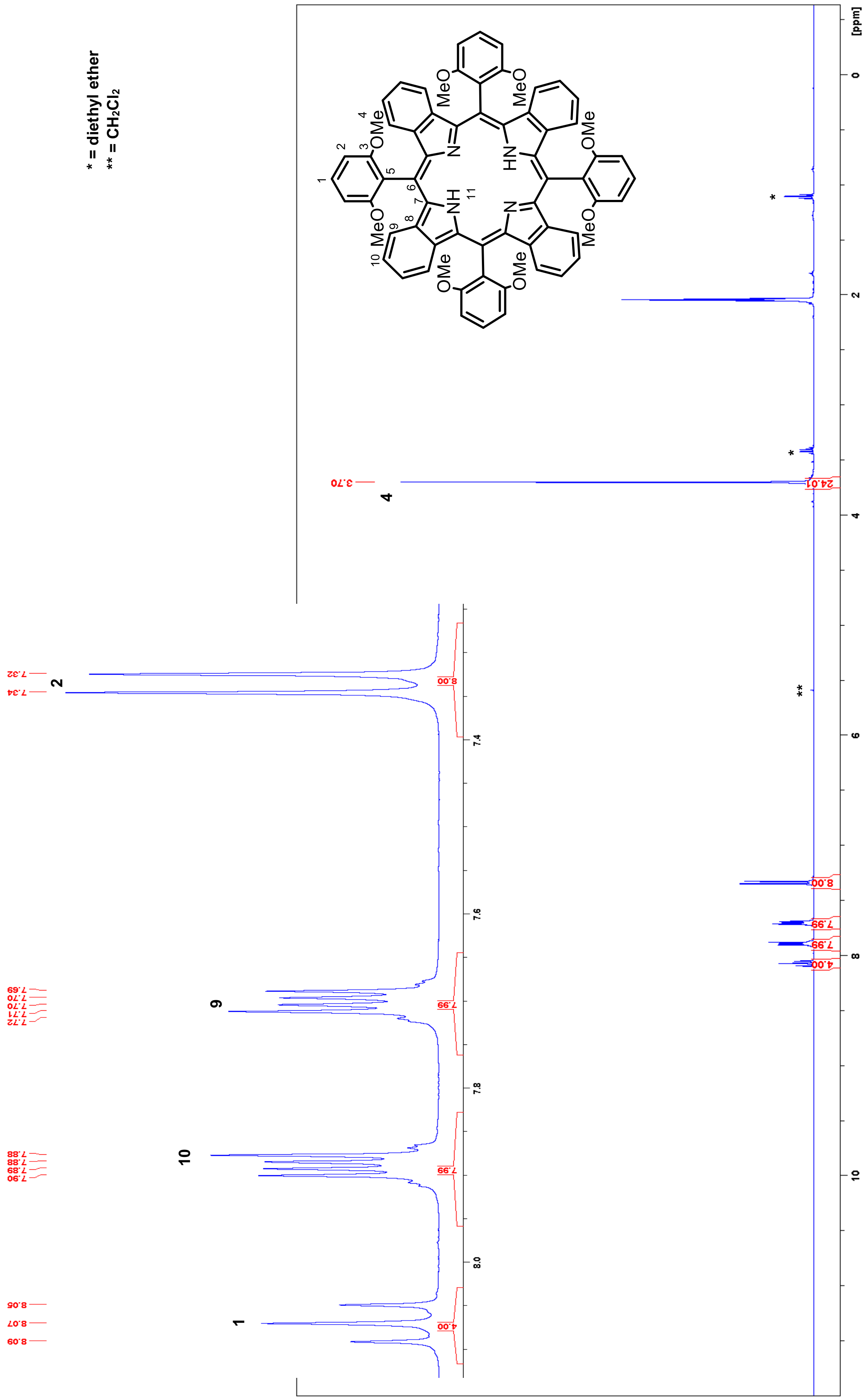

Figure S100. ^{1}H NMR (400 MHz, acetone-d_6/TFA-d_1, rt) of **22**.

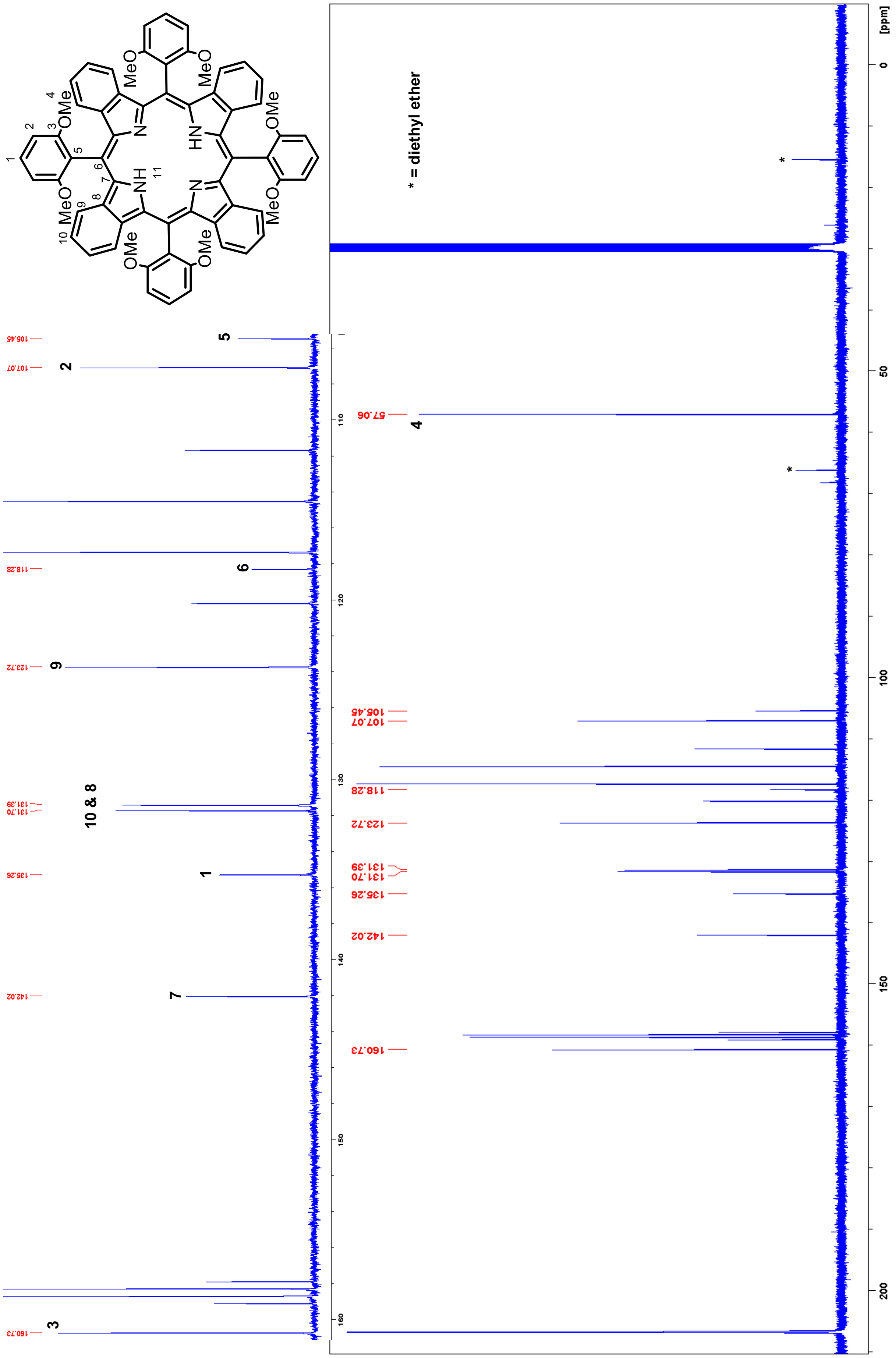

Figure S101. ^{13}C NMR (100 MHz, acetone-d_6/TFA-d_1, rt) of **22**.

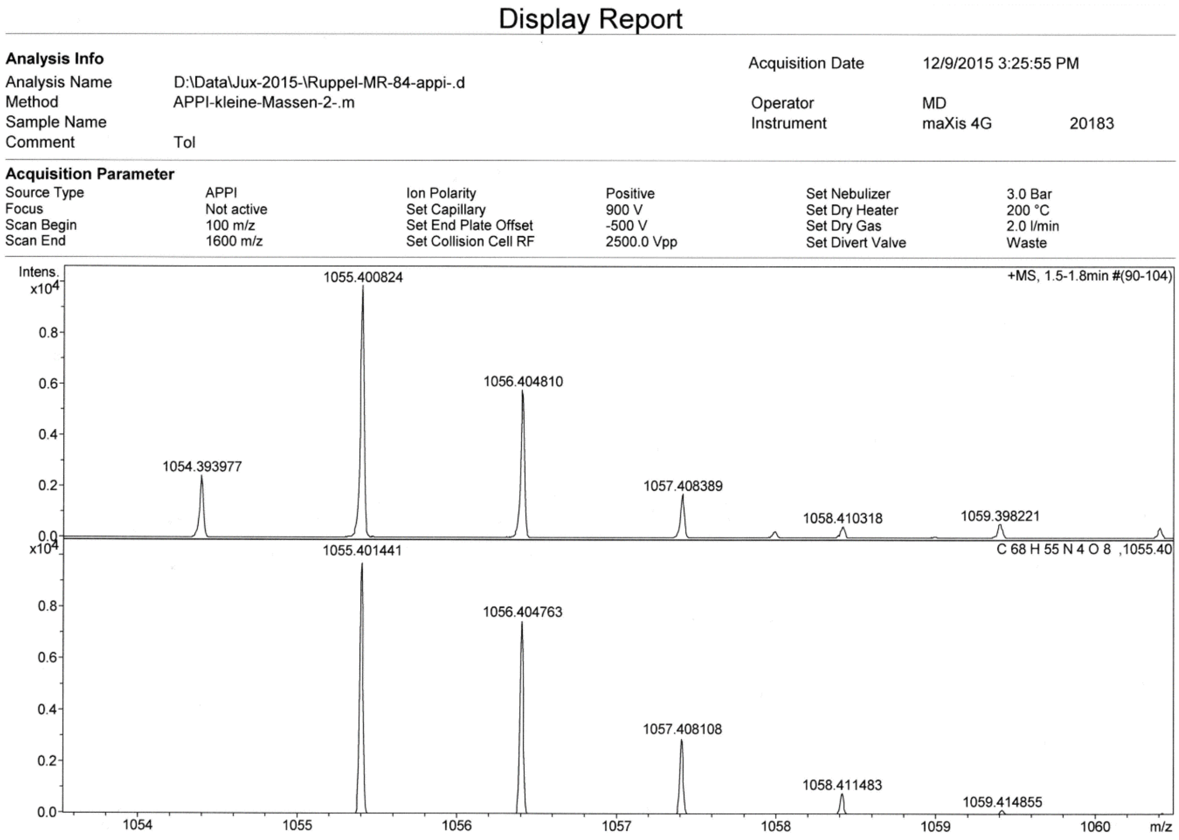

Figure S102. HRMS (APPI) of **22**.

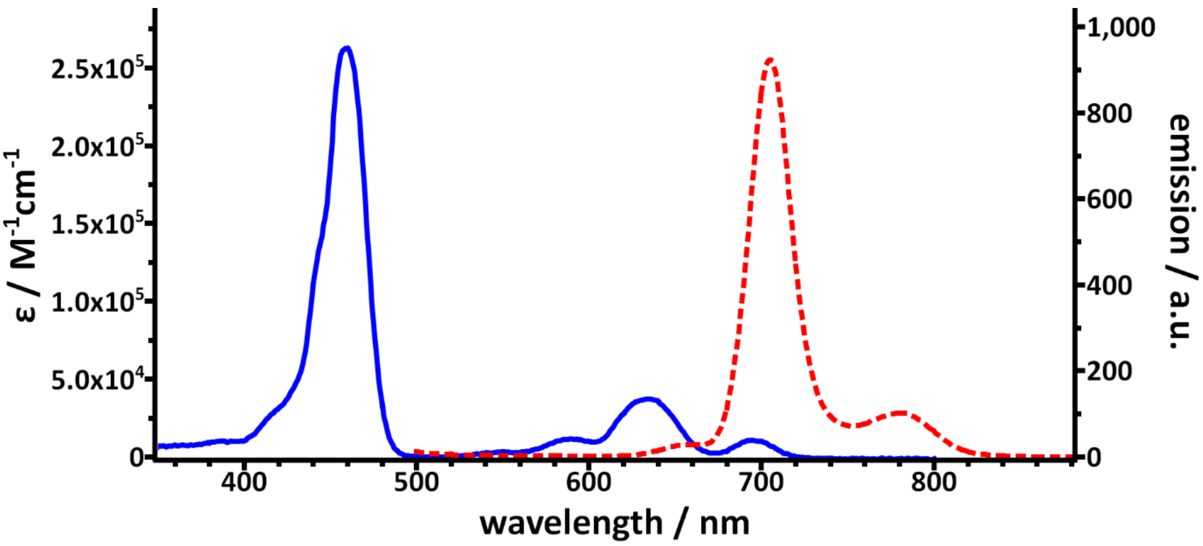

Figure S103. absorption (blue line) and emission spectrum of **22** (dashed red line; excitation at 459 nm) measured in CH_2Cl_2 + 1% NEt_3 at rt.

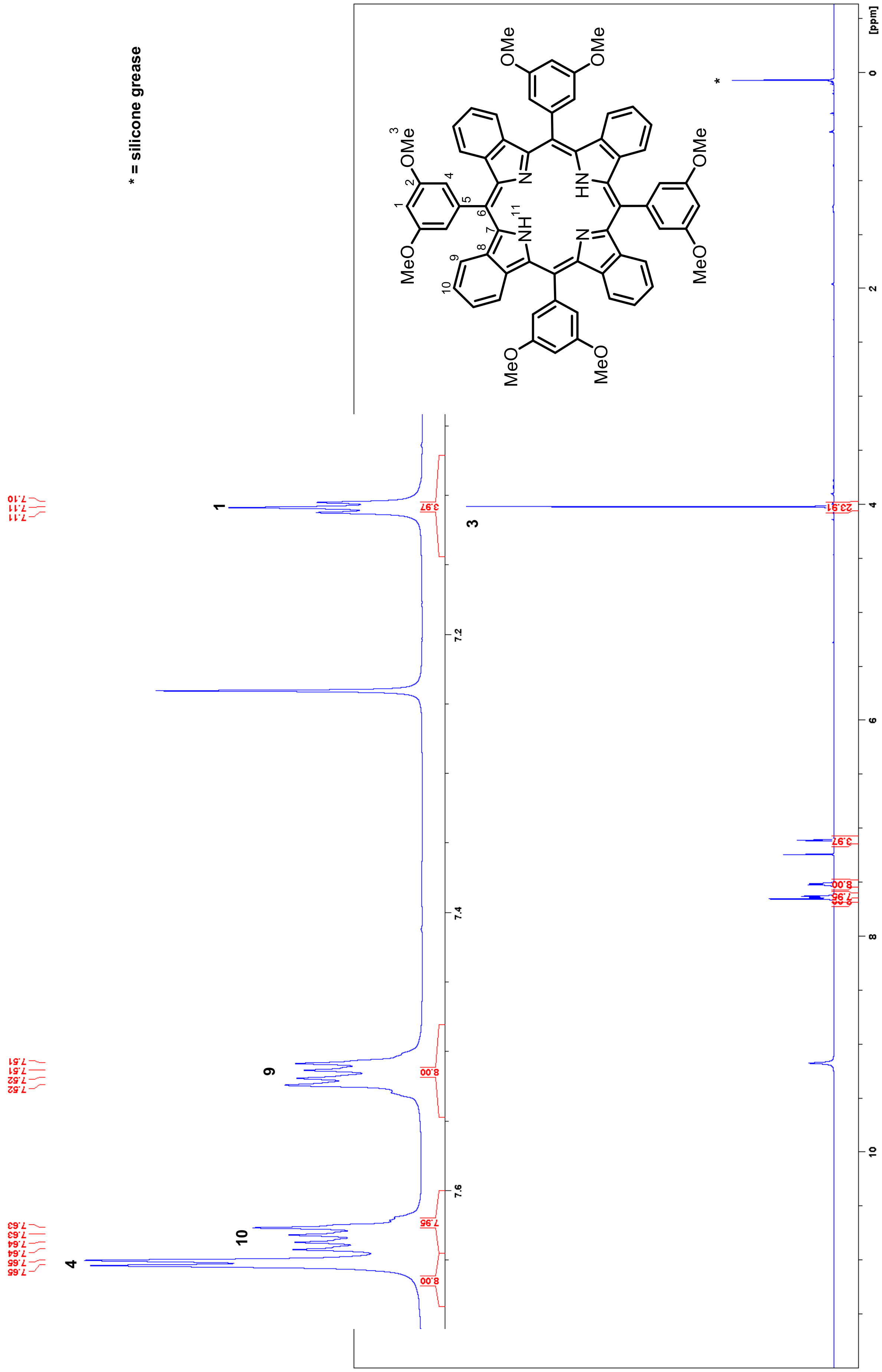

Figure S104. ^{1}H NMR (600 MHz, $CDCl_3$/TFA-d_1, rt) of **23**.

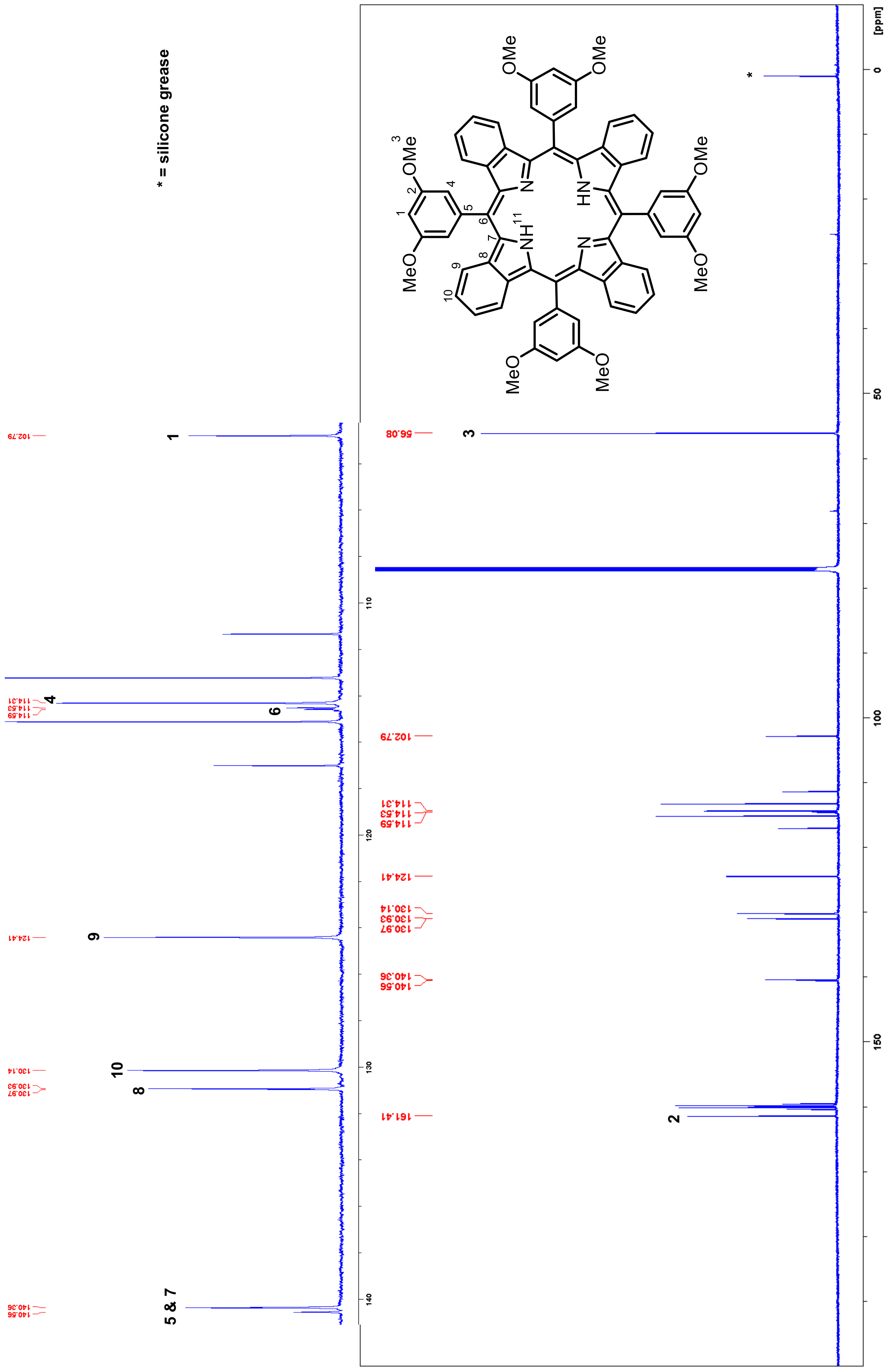

Figure S105. ^{13}C NMR (150 MHz, $CDCl_3$/TFA-d_1, rt) of **23**.

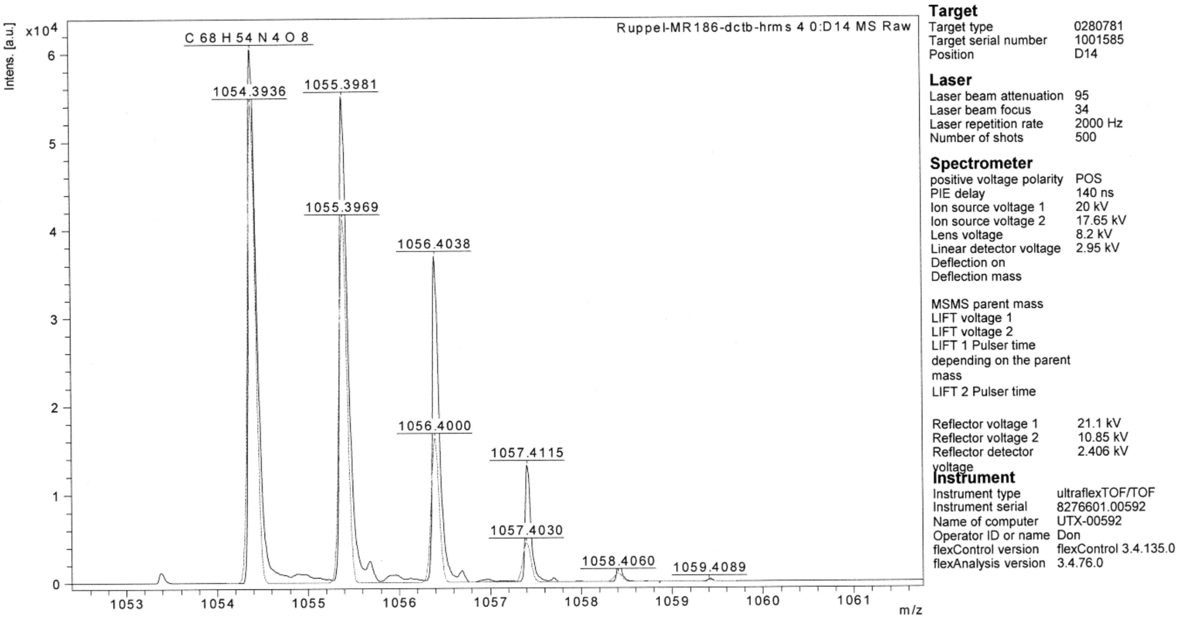

Figure S106. HRMS (MALDI) of **23**.

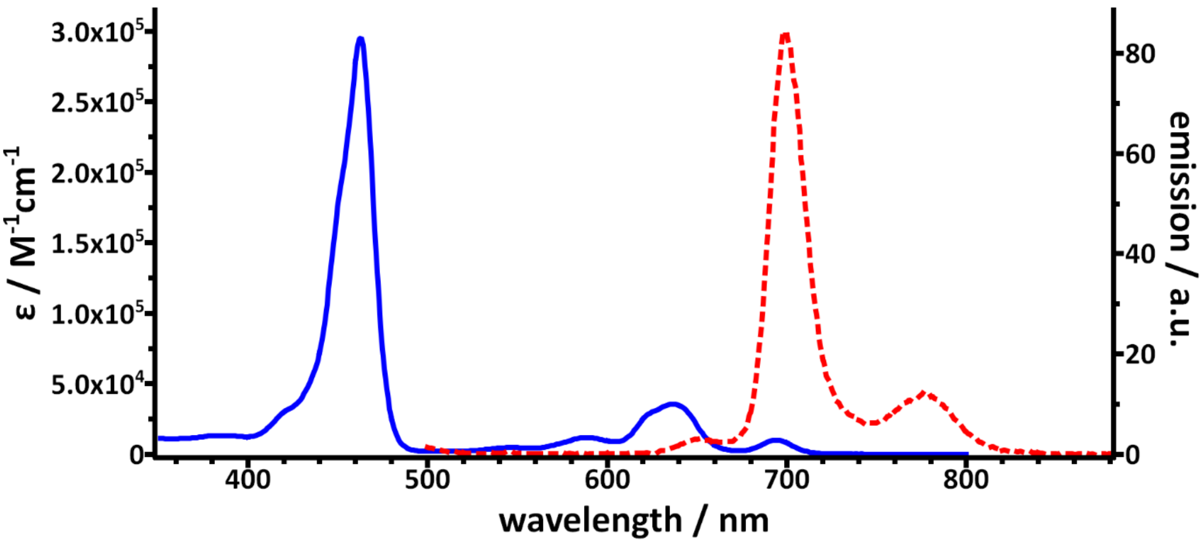

Figure S107. absorption (blue line) and emission spectrum of **23** (dashed red line; excitation at 463 nm) measured in CH_2Cl_2 + 1% NEt_3 at rt.

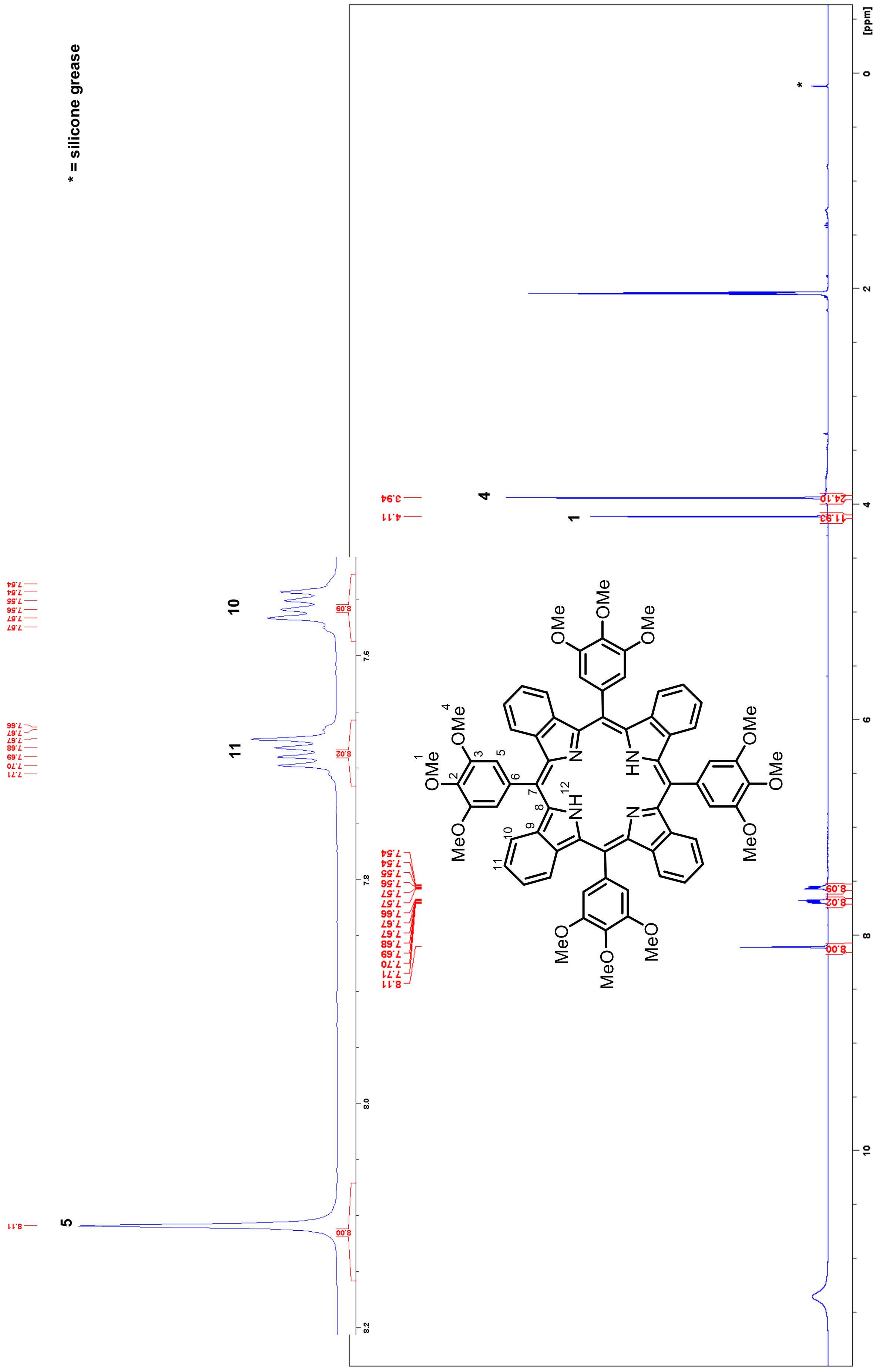

Figure S108. ^{1}H NMR (400 MHz, acetone-d_6/TFA-d_1, rt) of **24**.

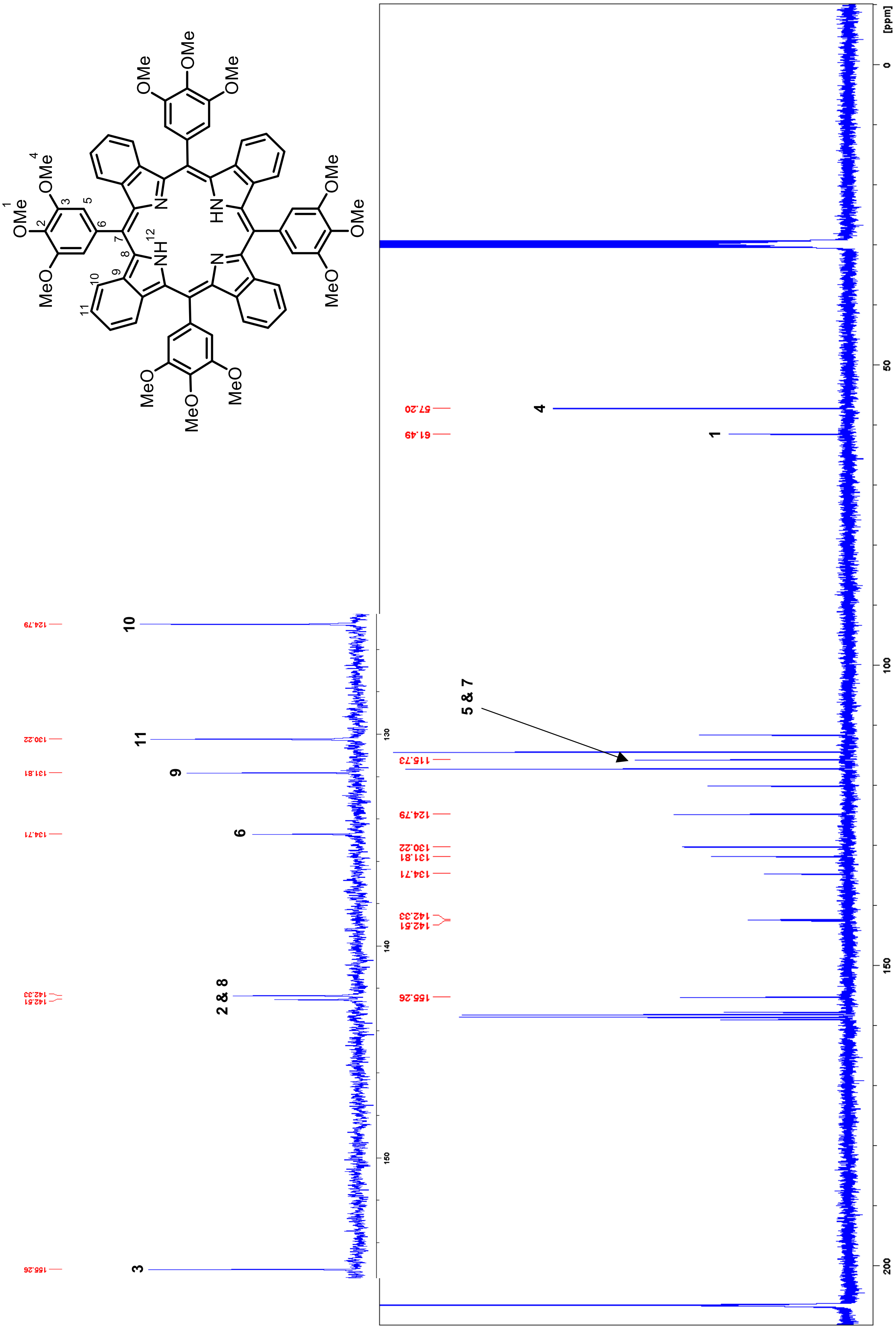

Figure S109. ^{1}H NMR (100 MHz, acetone-d$_6$/TFA-d$_1$, rt) of **24**.

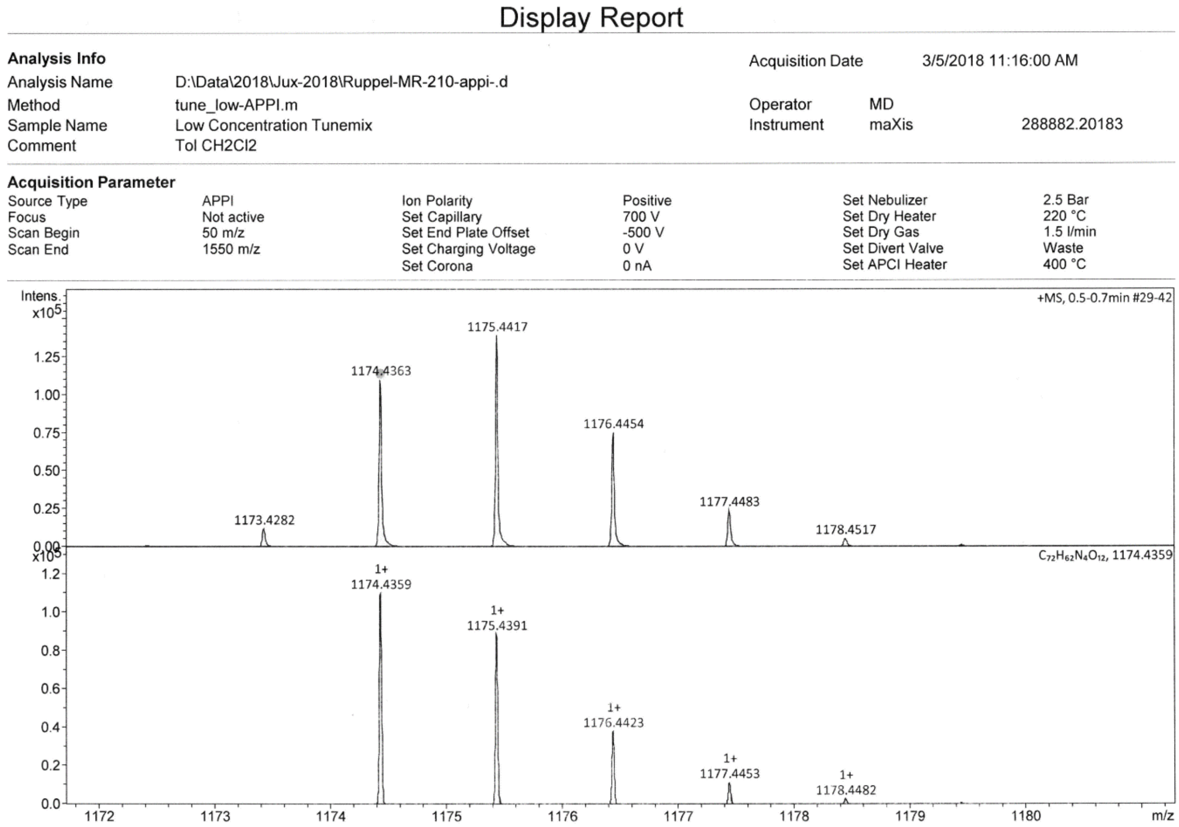

Figure S110. HRMS (APPI, toluene/CH_2Cl_2) of **24**.

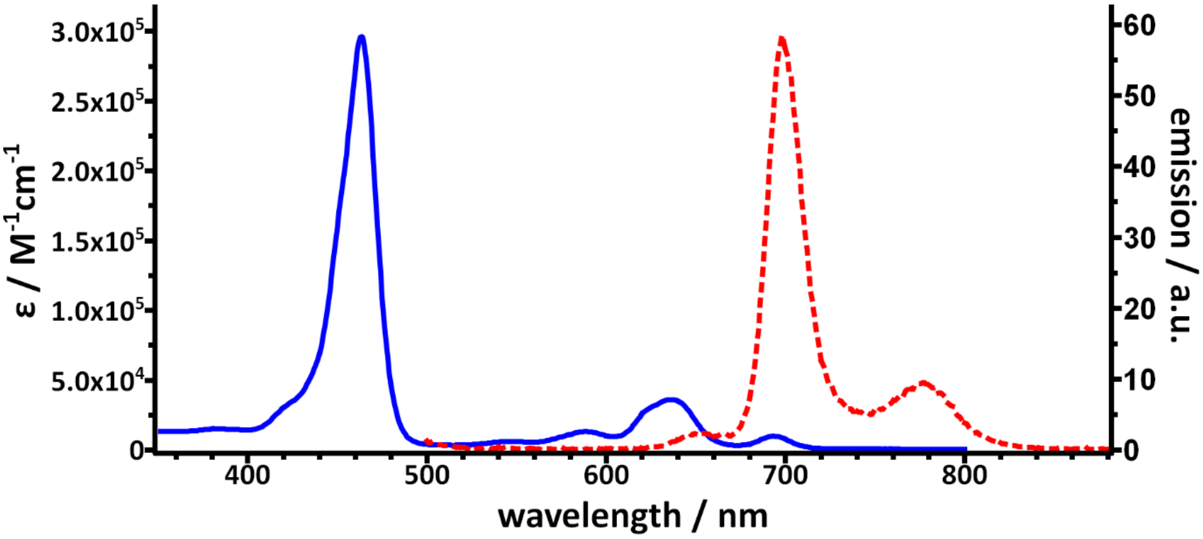

Figure S111. absorption (blue line) and emission spectrum of **24** (dashed red line; excitation at 463 nm) measured in CH_2Cl_2 + 1% NEt_3 at rt.

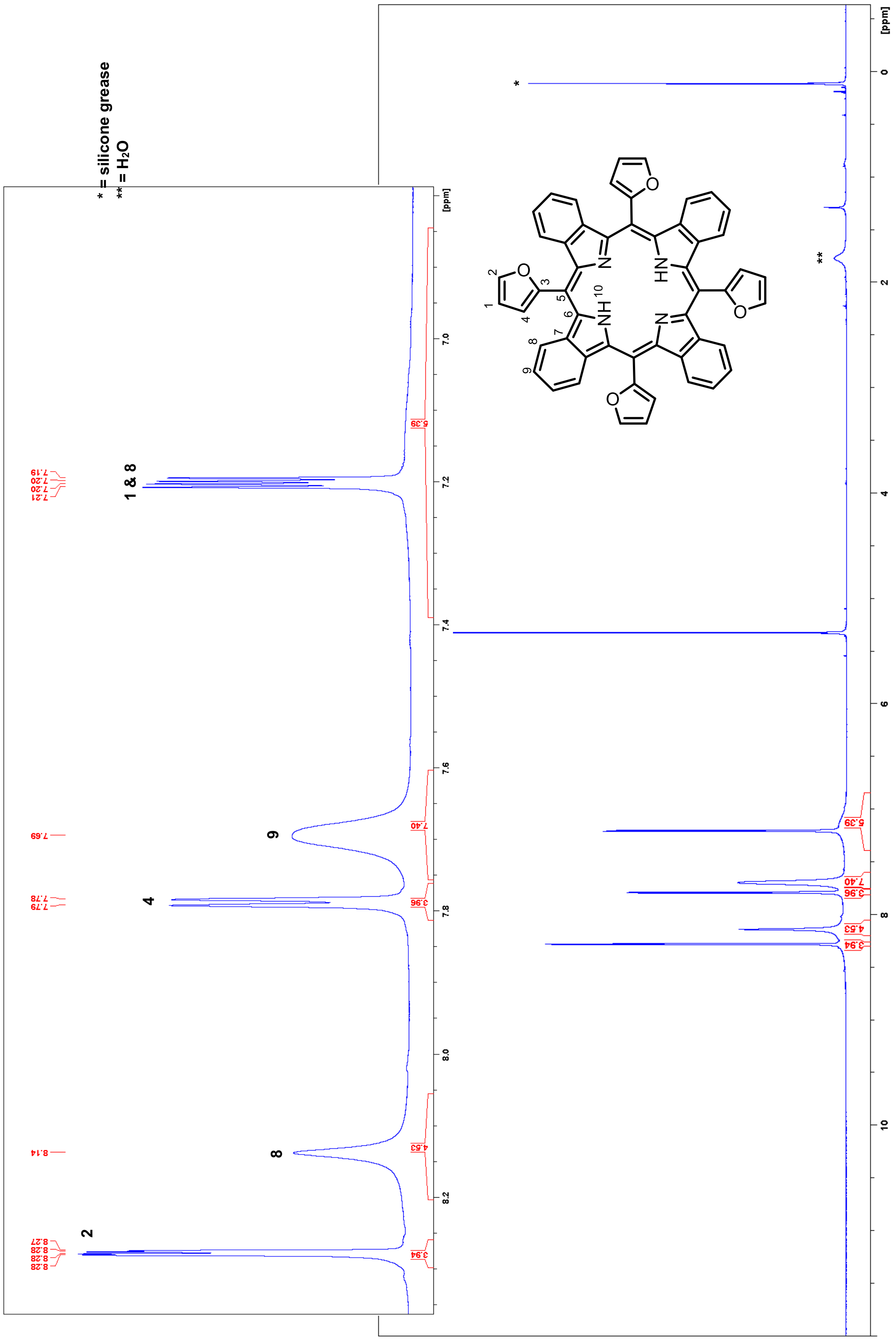

Figure S112. ^{1}H NMR (400 MHz, CD_2Cl_2/TFA-d$_1$, rt) of **25**.

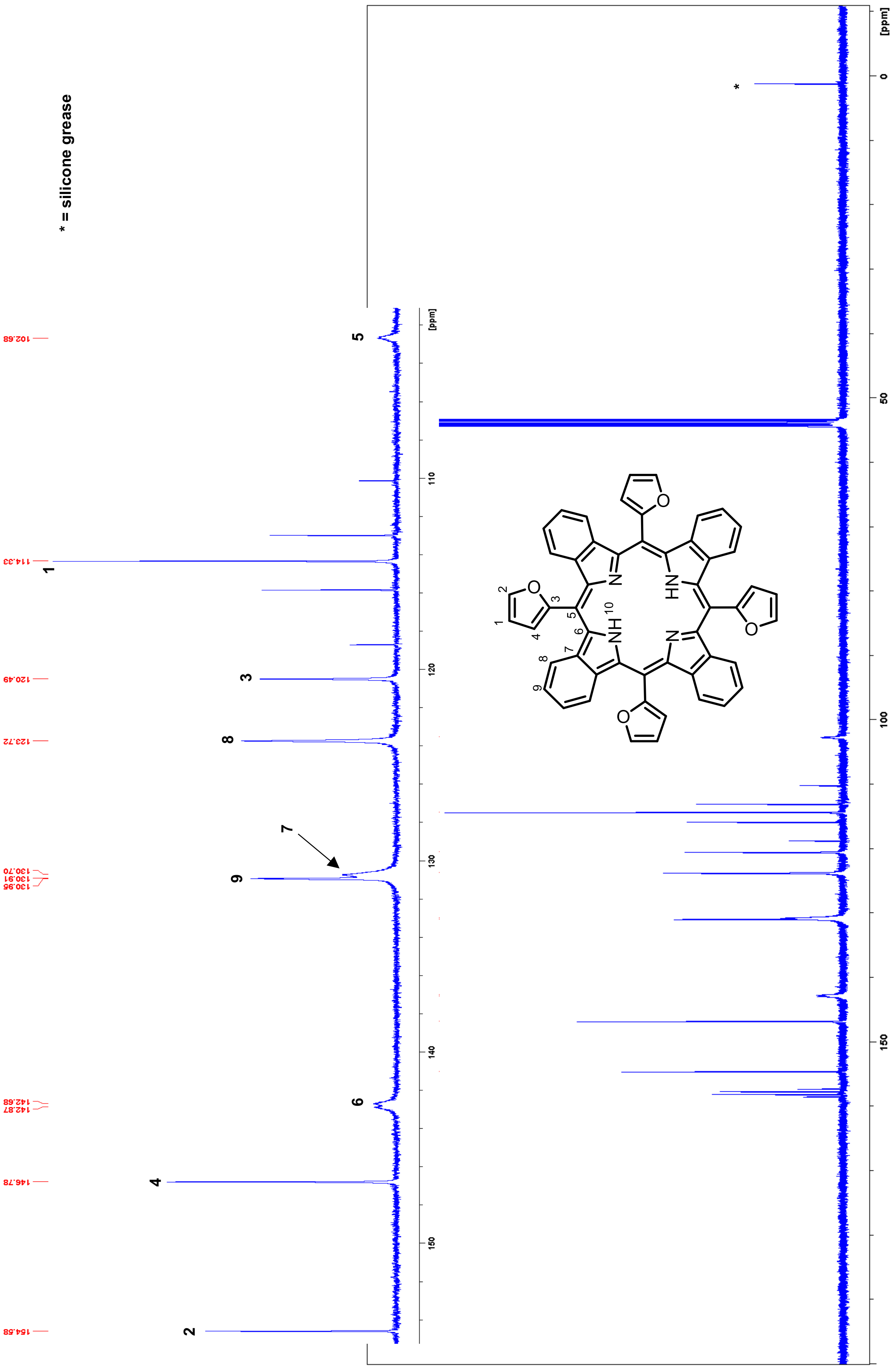

Figure S113. ^{1}H NMR (100 MHz, CD_2Cl_2/TFA-d_1, rt) of **25**.

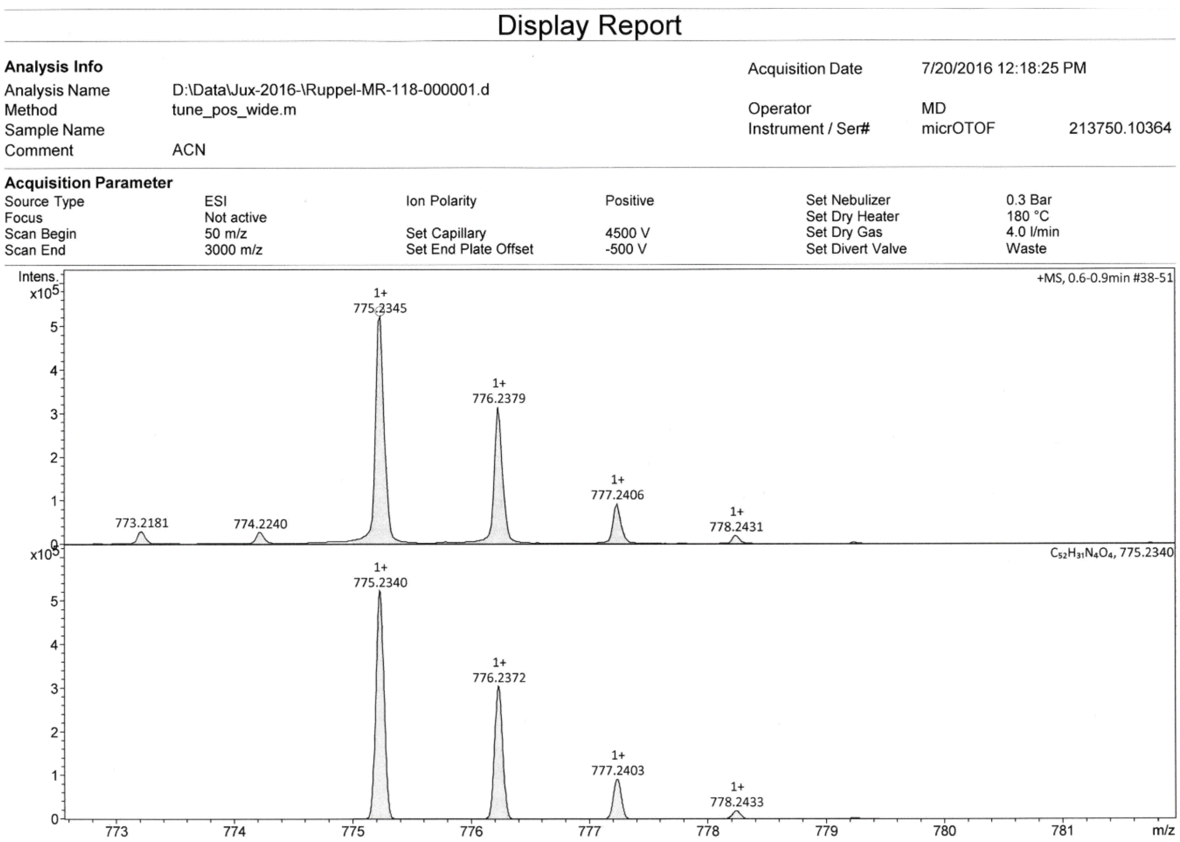

Figure S114. HRMS (ESI, MeCN) of **25**.

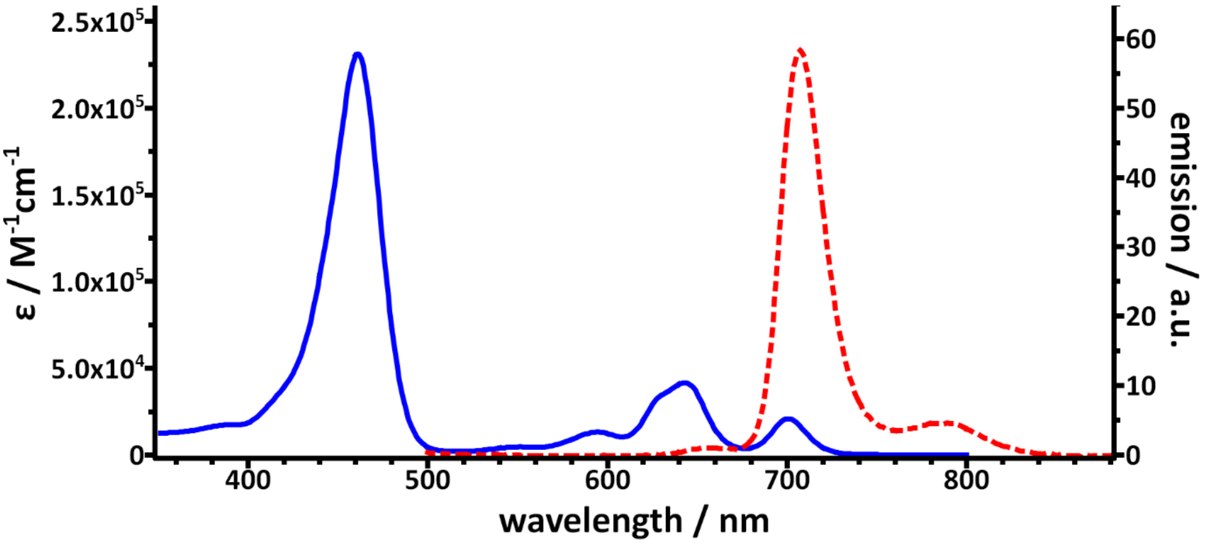

Figure S115. absorption (blue line) and emission spectrum of **25** (dashed red line; excitation at 461 nm) measured in CH_2Cl_2 + 1% NEt_3 at rt.

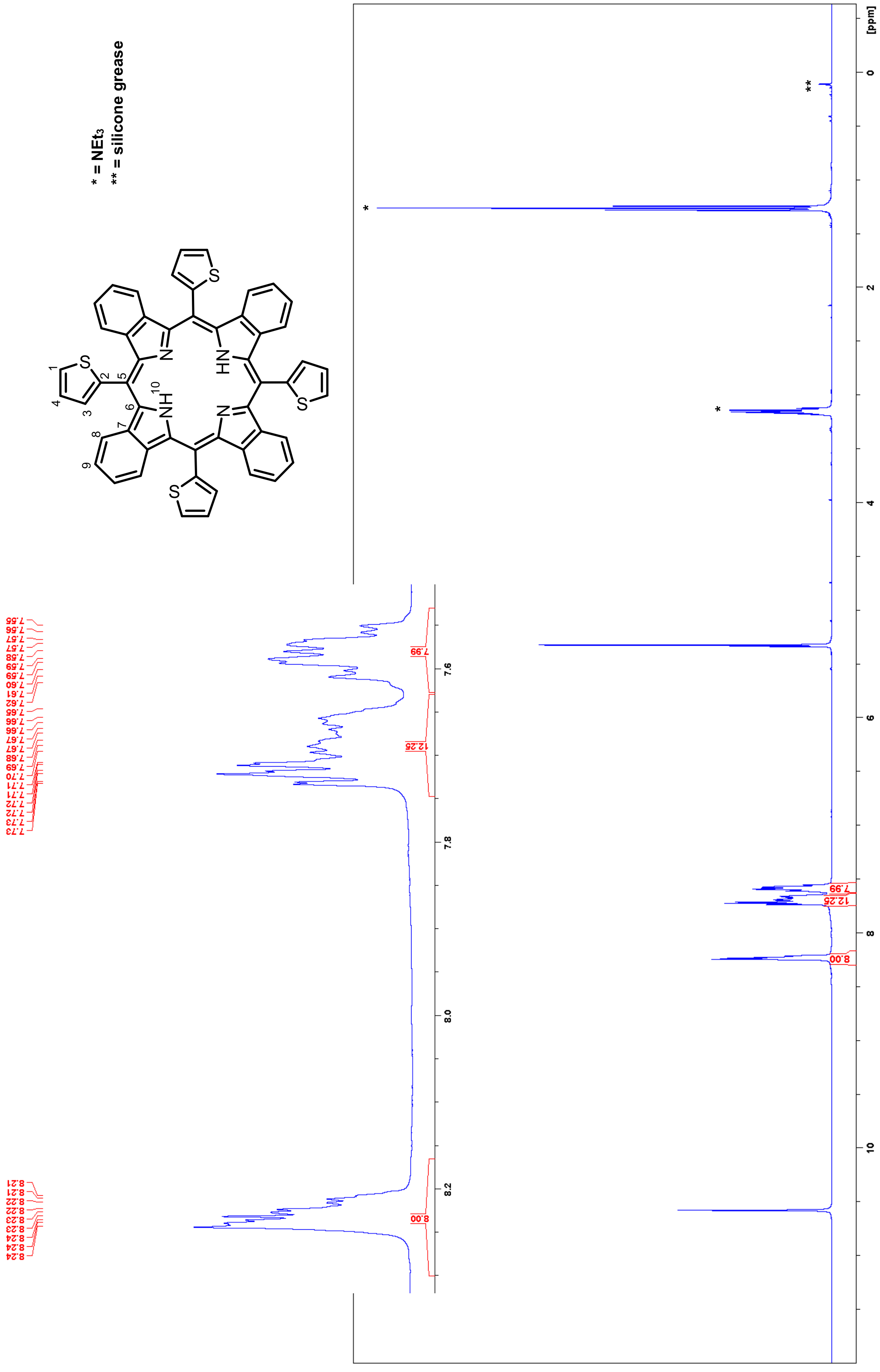

Figure S116. ^{1}H NMR (400 MHz, CD_2Cl_2/TFA-d_1, rt) of **26**.

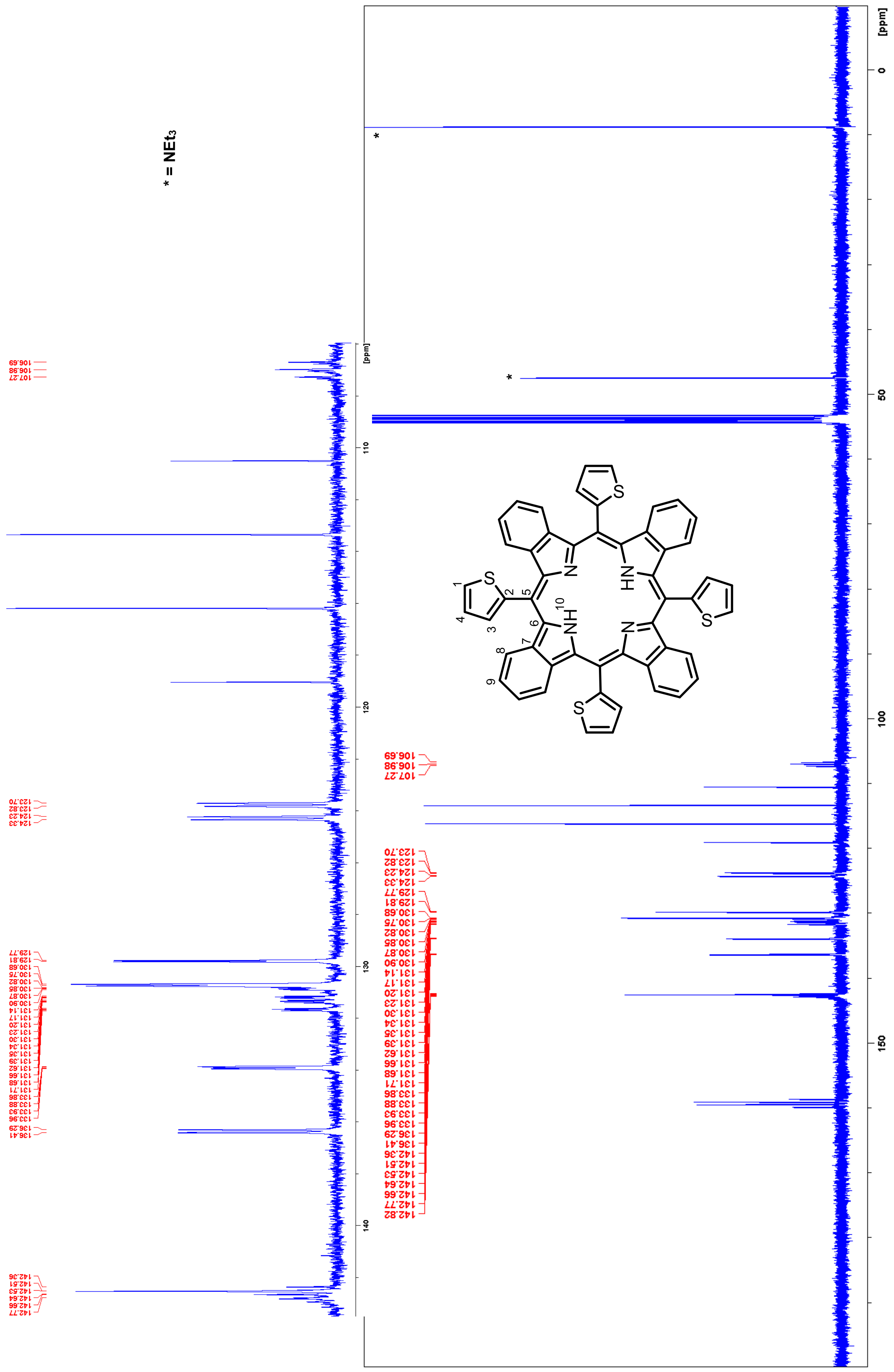

Figure S117. ^{13}C NMR (100 MHz, CD_2Cl_2/TFA-d_1, rt) of **26**.

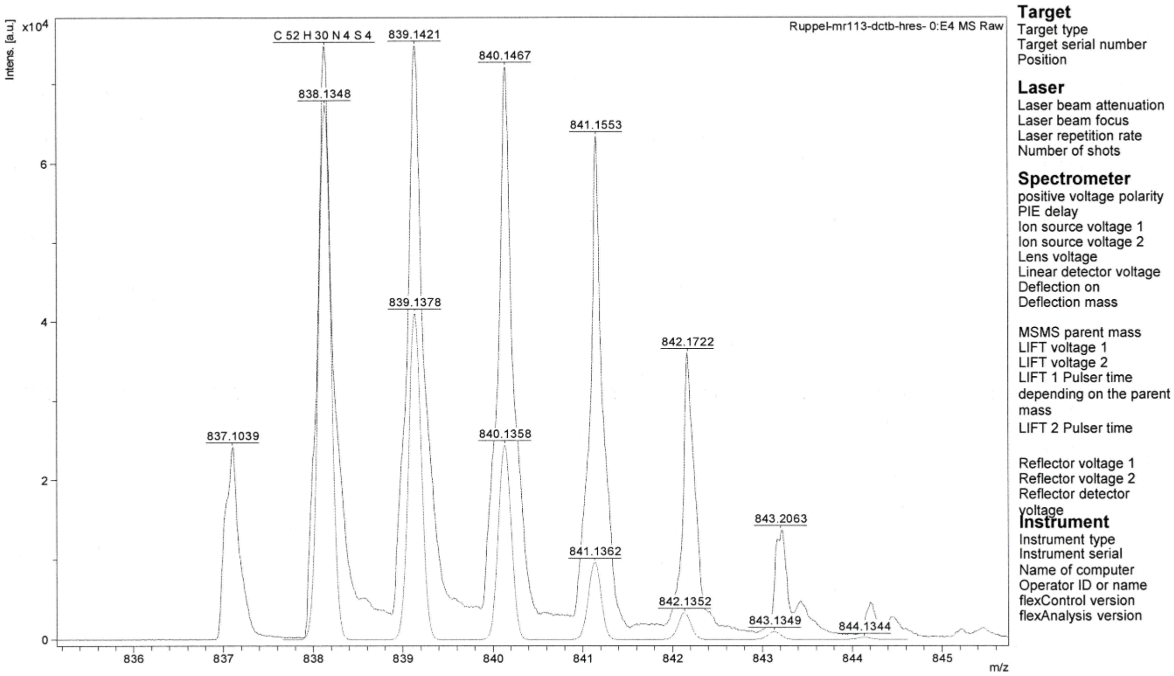

Figure S118. HRMS (MALDI) of **26**.

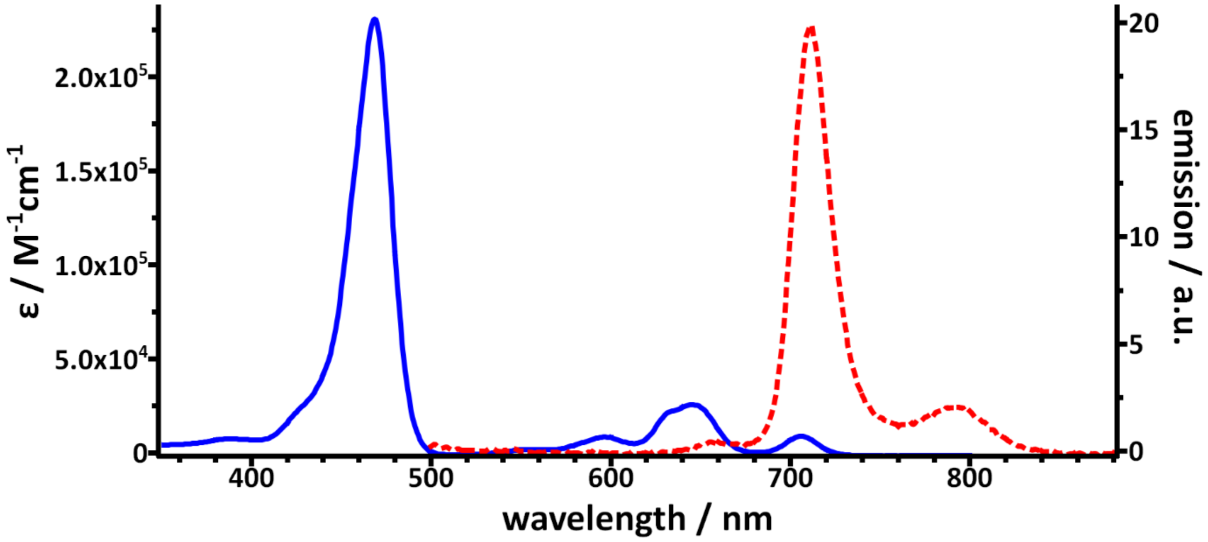

Figure S119. absorption (blue line) and emission spectrum of **26** (dashed red line; excitation at 469 nm) measured in CH_2Cl_2 + 1% NEt_3 at rt.

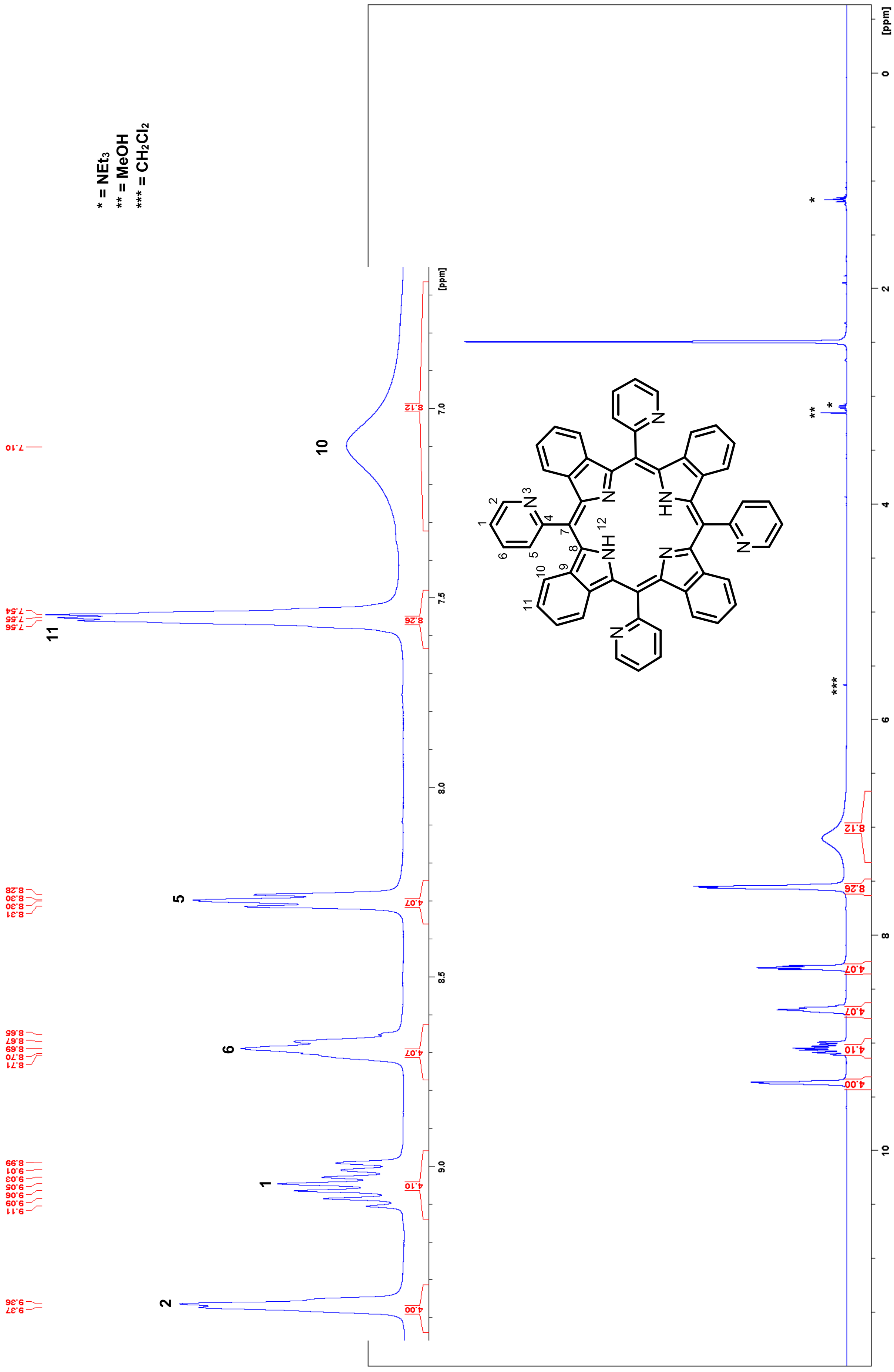

Figure S120. ^{1}H NMR (400 MHz, DMSO-d$_6$/TFA-d$_1$, rt) of **27**.

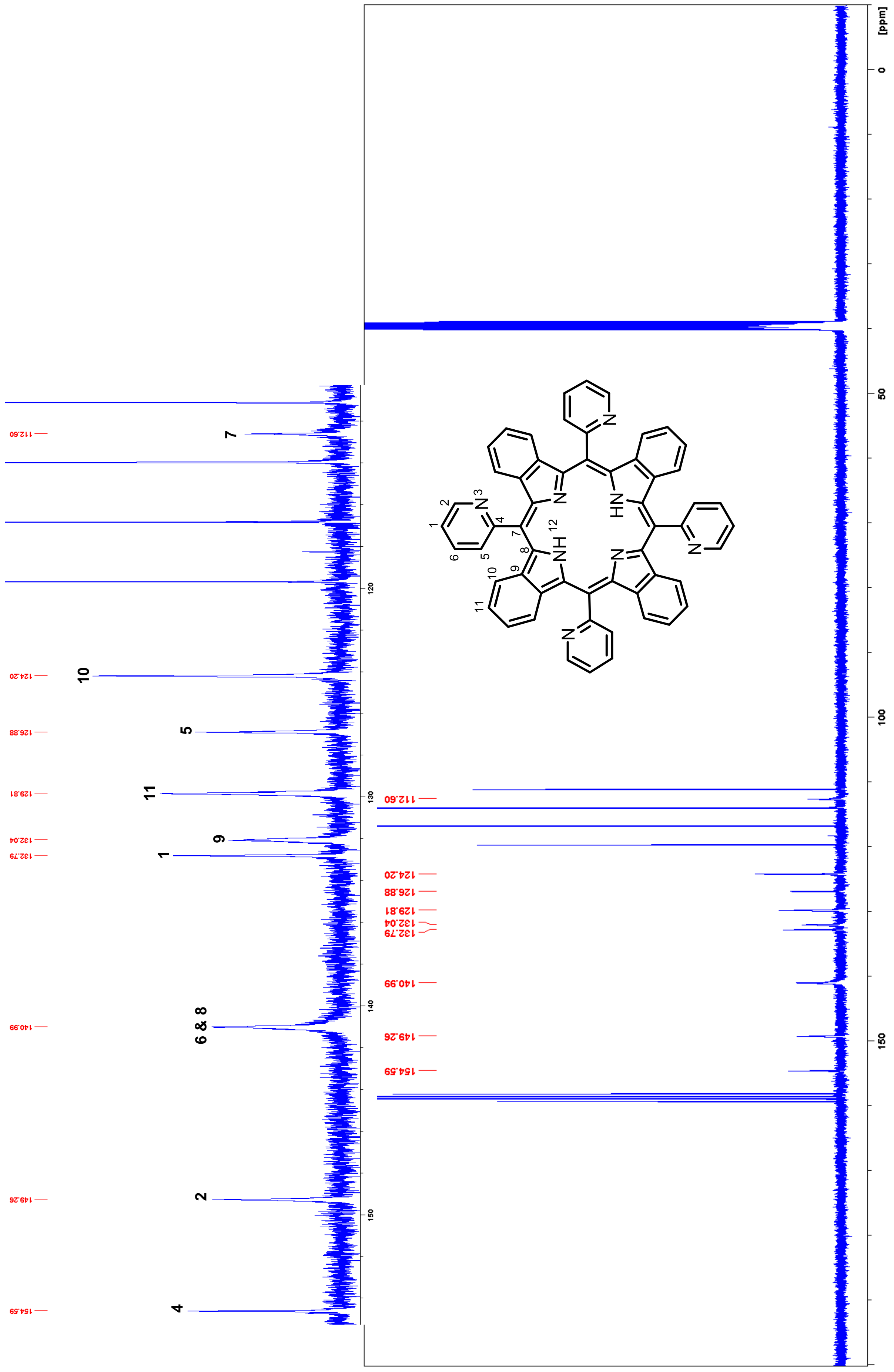

Figure S121. ^{13}C NMR (100 MHz, DMSO-d_6/TFA-d_1, rt) of **27**.

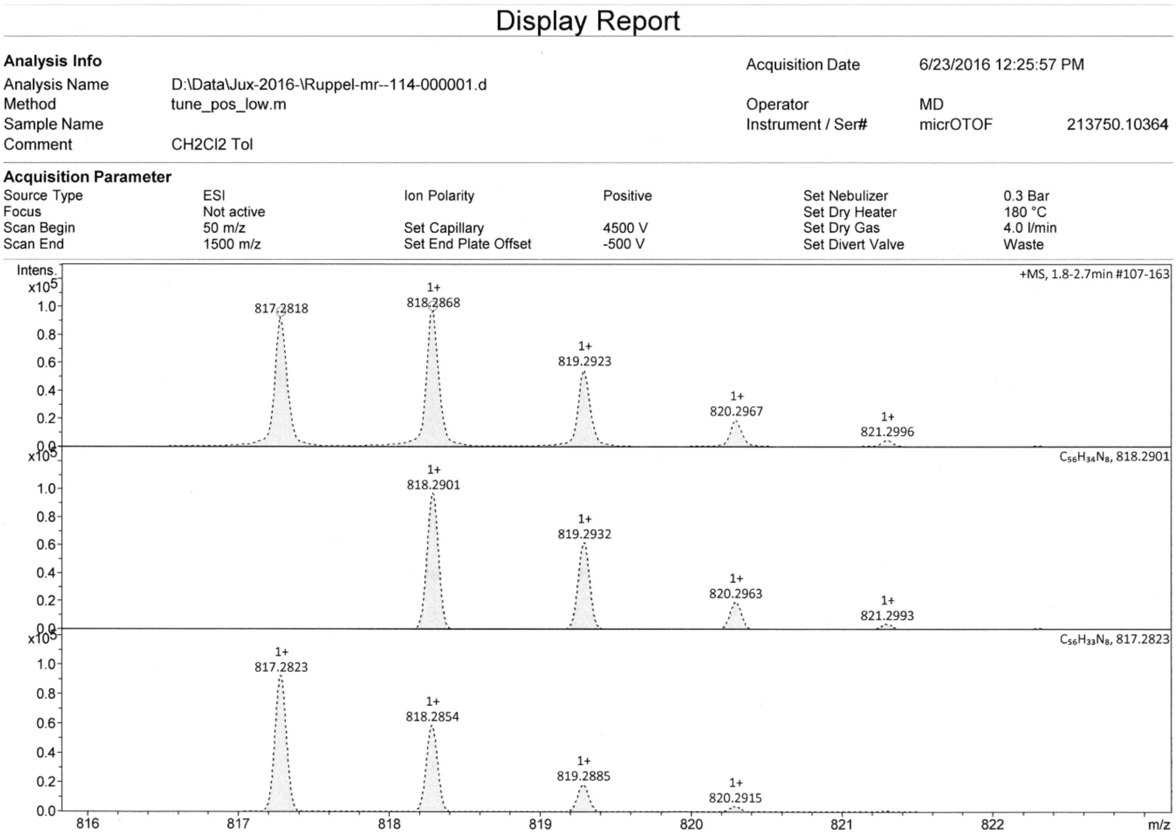

Figure S122. HRMS (ESI, toluene/CH_2Cl_2) of **27**.

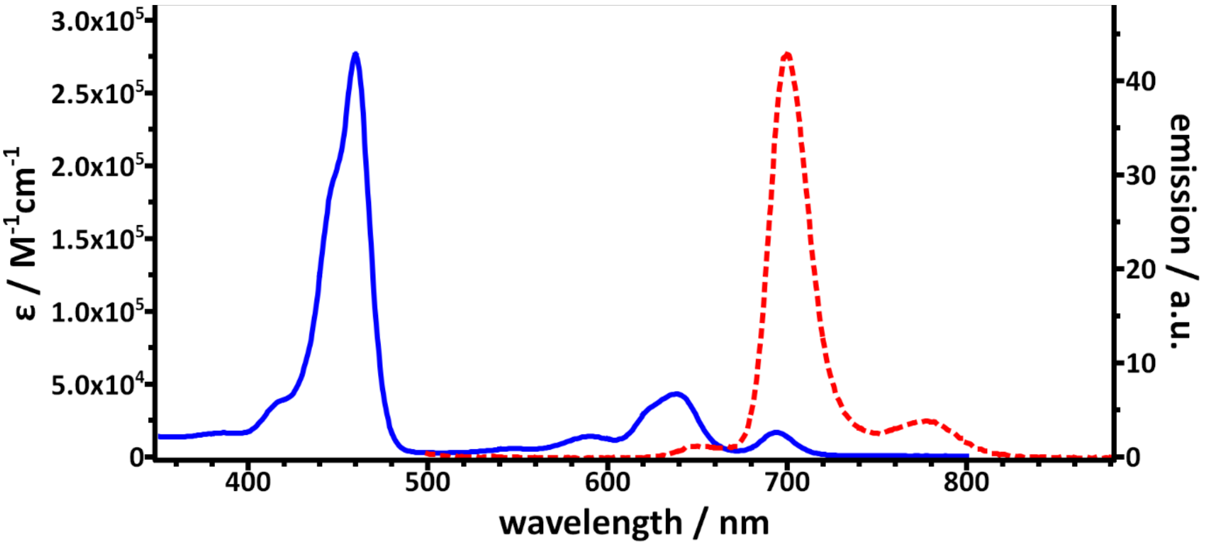

Figure S123. absorption (blue line) and emission spectrum of **27** (dashed red line; excitation at 460 nm) measured in CH_2Cl_2 + 1% NEt_3 at rt.

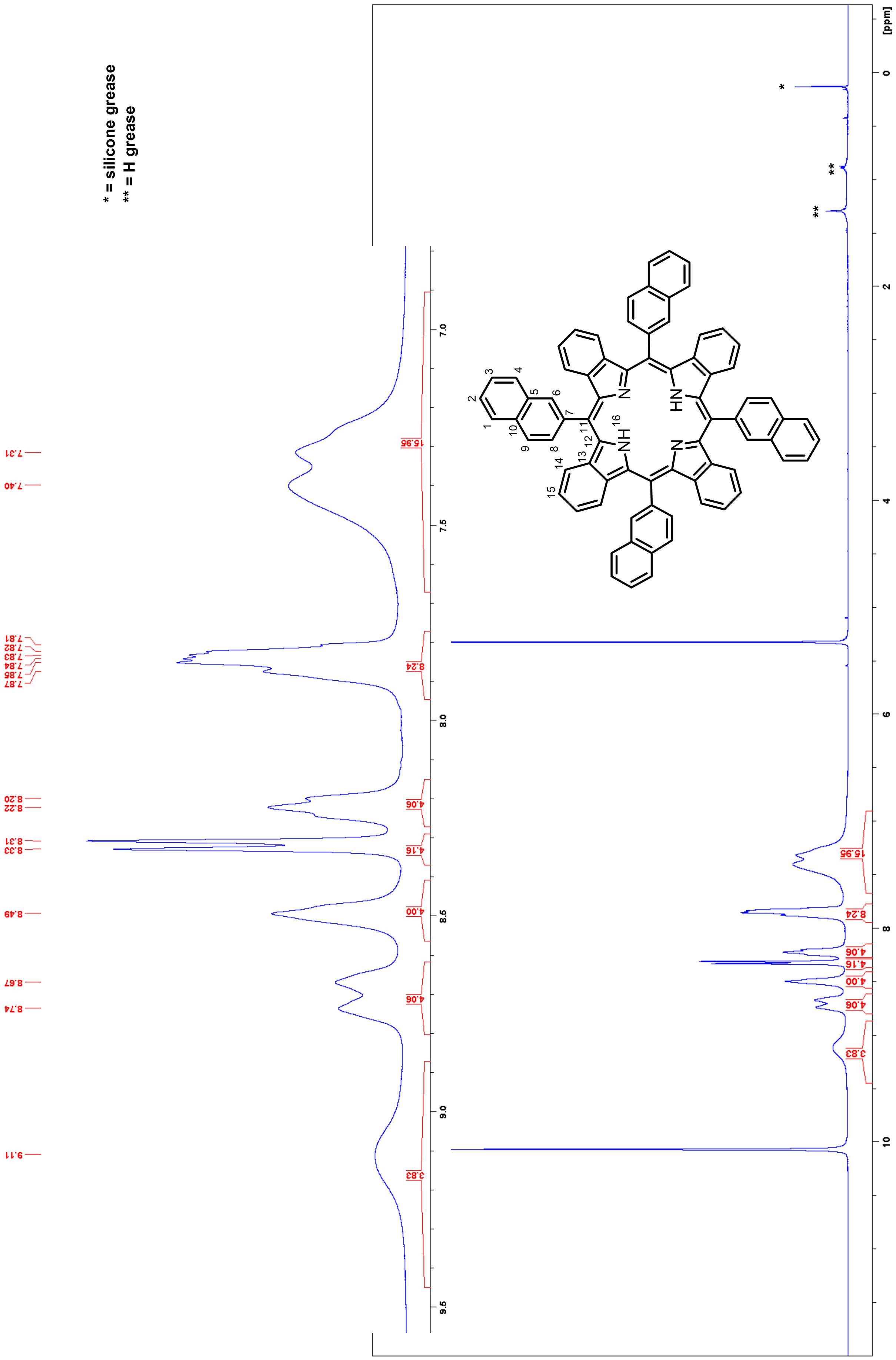

Figure S124. ^1H NMR (400 MHz, CD_2Cl_2/TFA-d_1, rt) of **28**.

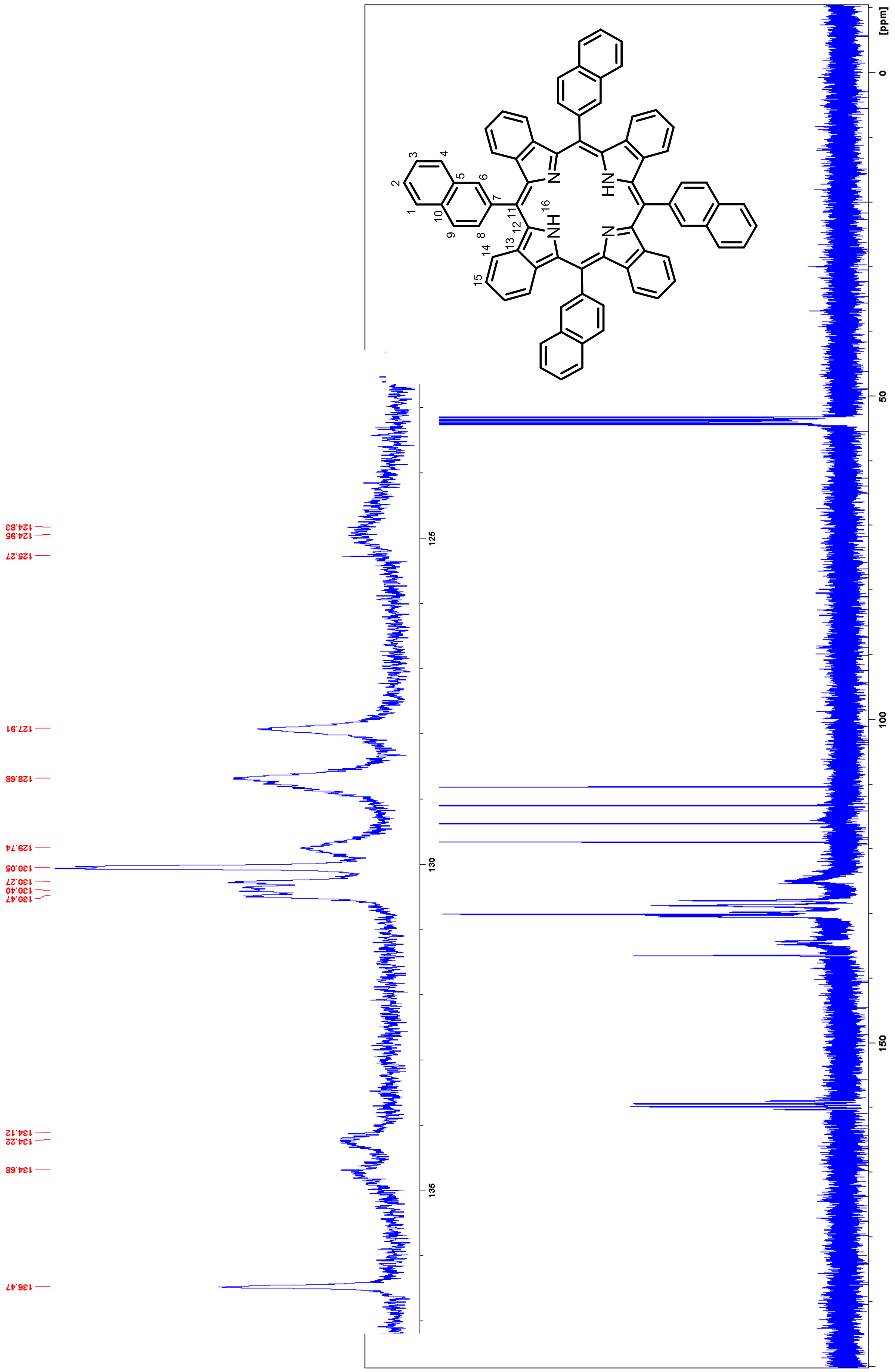

Figure S125. ^{13}C NMR (100 MHz, CD_2Cl_2/TFA-d_1, rt) of **28**.

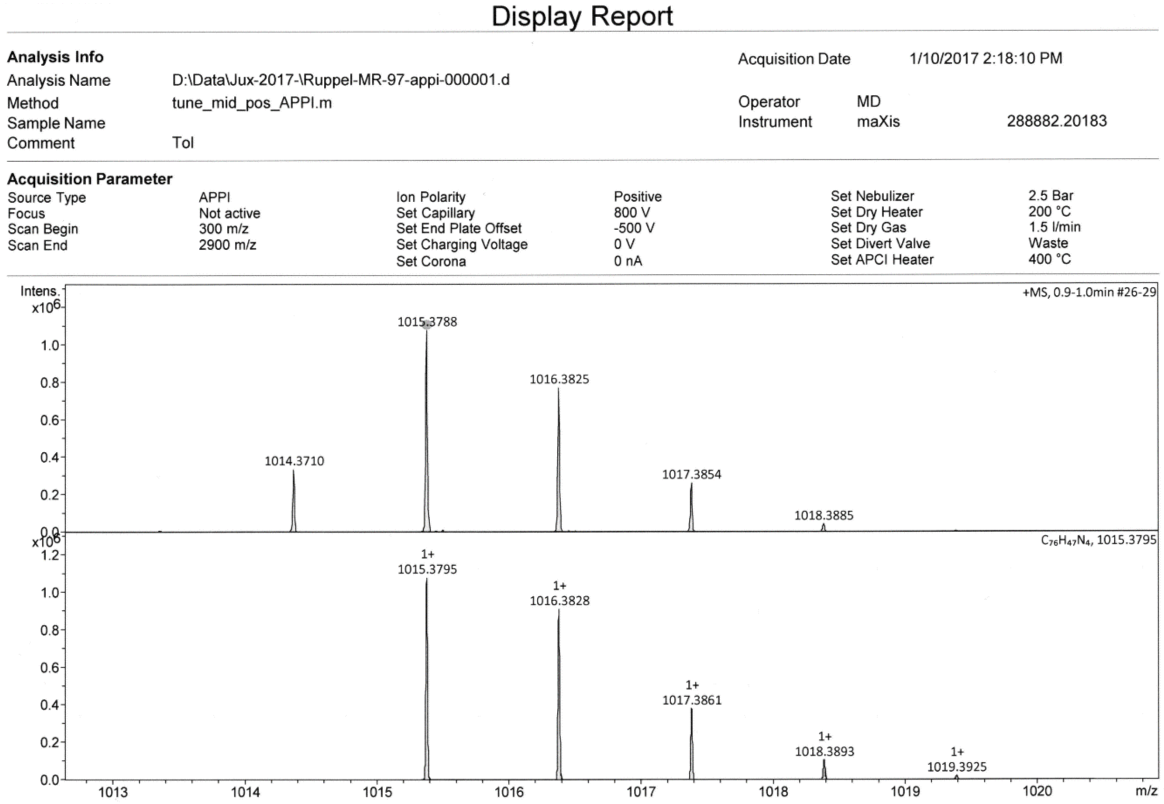

Figure S126. HRMS (APPI, toluene) of **28**.

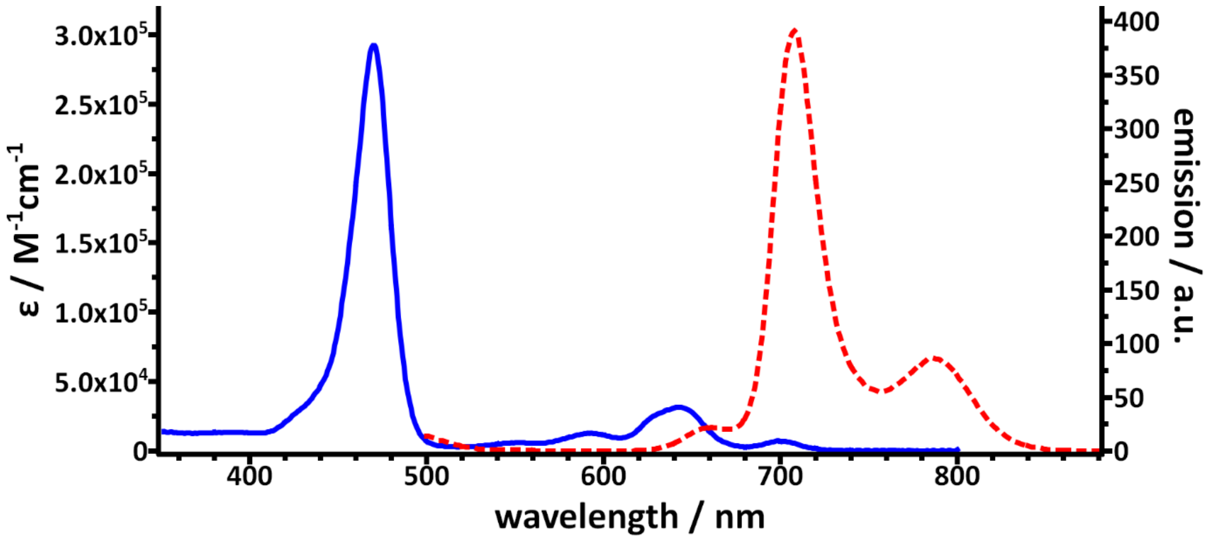

Figure S127. absorption (blue line) and emission spectrum of **28** (dashed red line; excitation at 470 nm) measured in CH_2Cl_2 + 1% NEt_3 at rt.

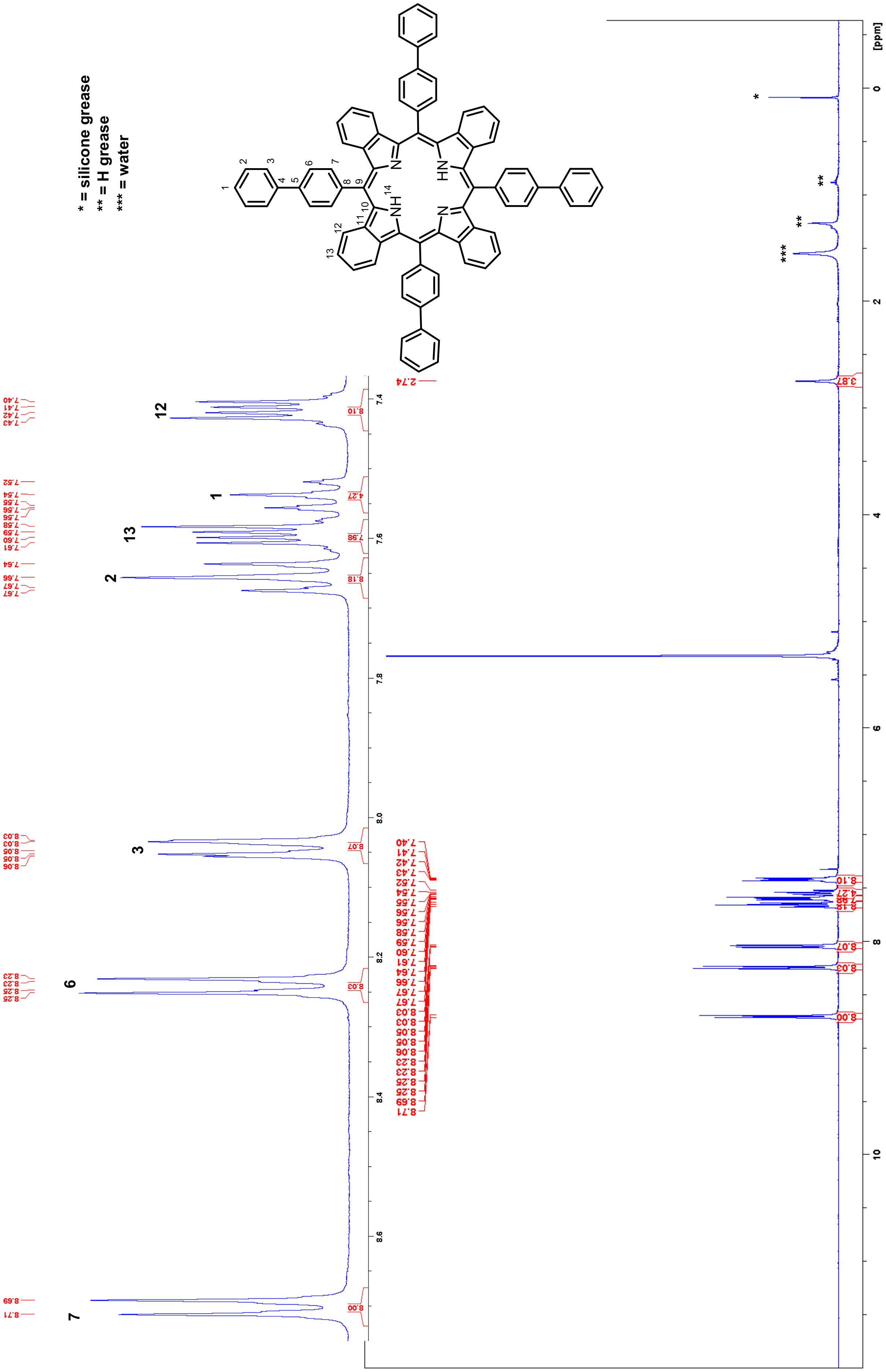

Figure S128. ^{1}H NMR (400 MHz, CD_2Cl_2, rt) of **29**.

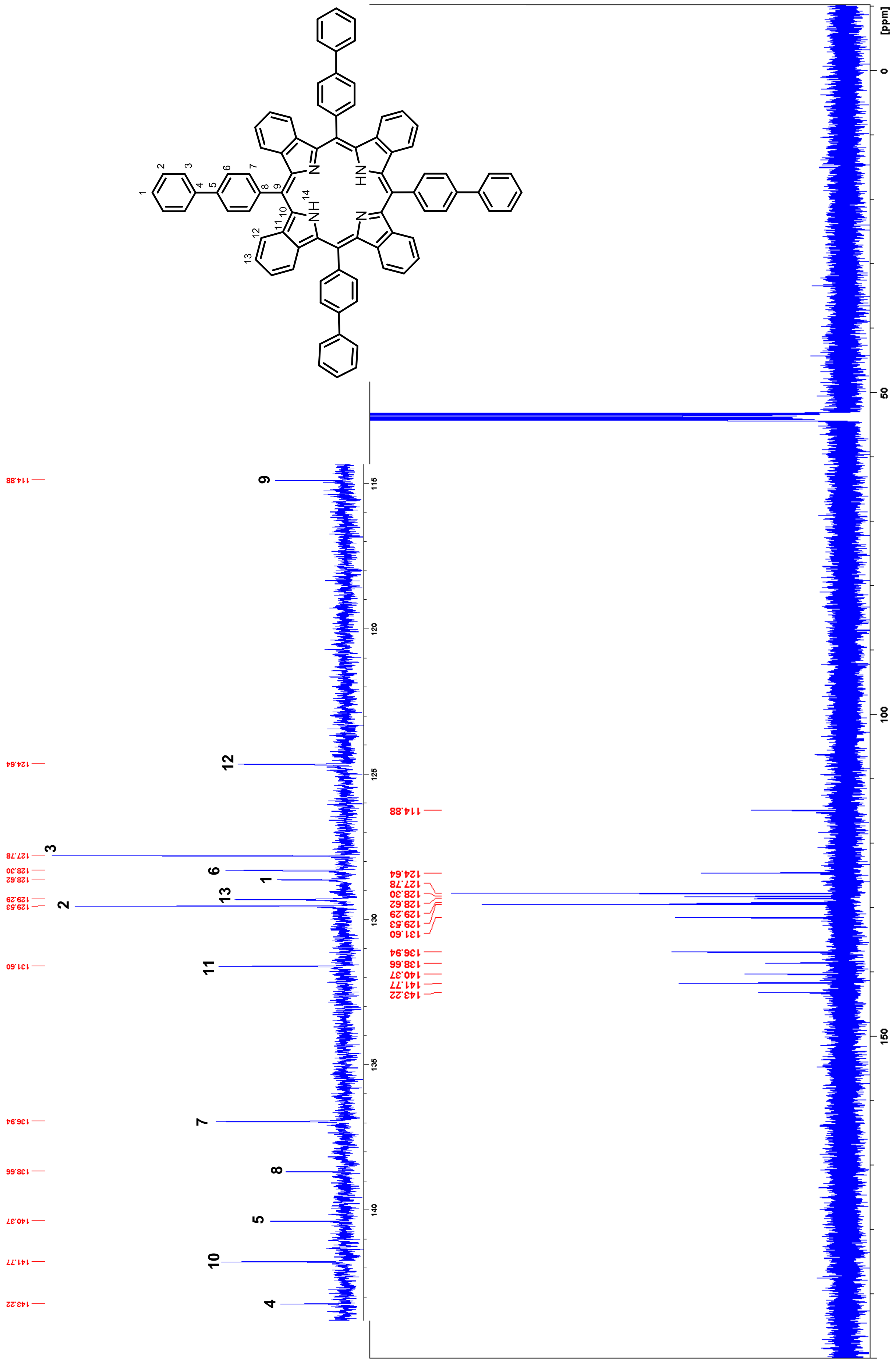

Figure S129. ^{13}C NMR (100 MHz, CD_2Cl_2, rt) of **29**.

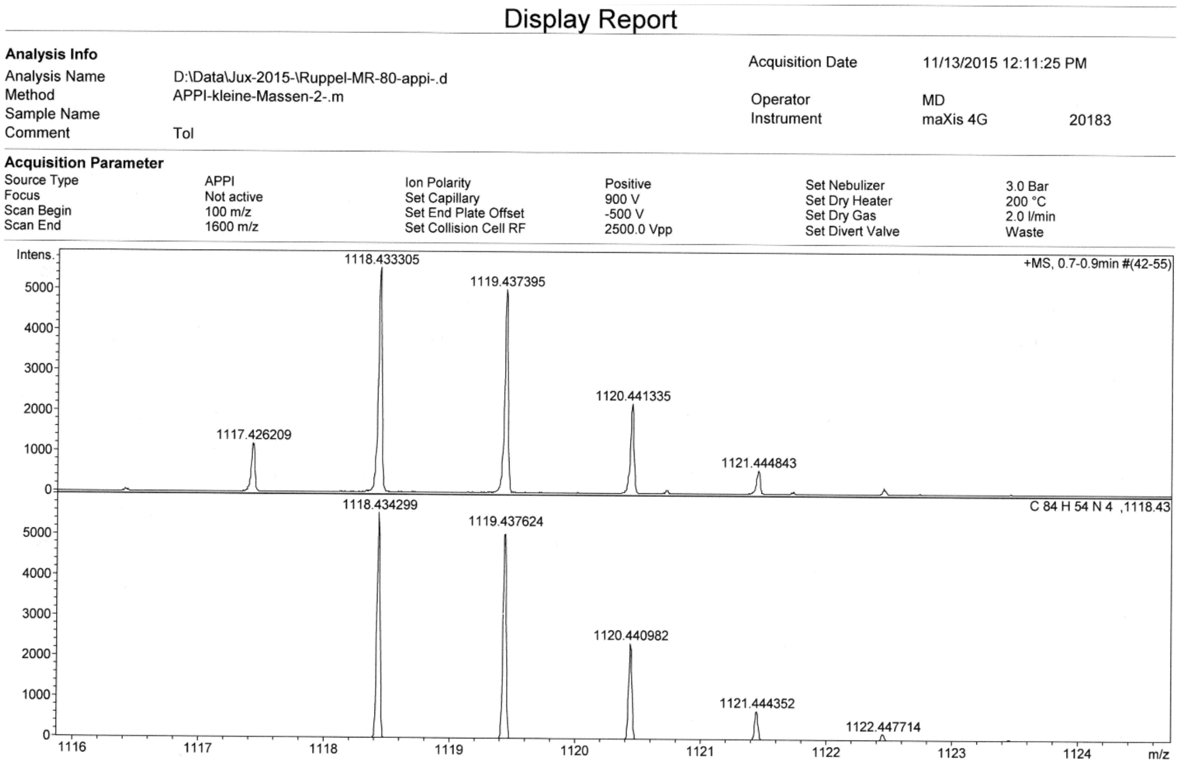

Figure S130. HRMS (APPI) of **29**.

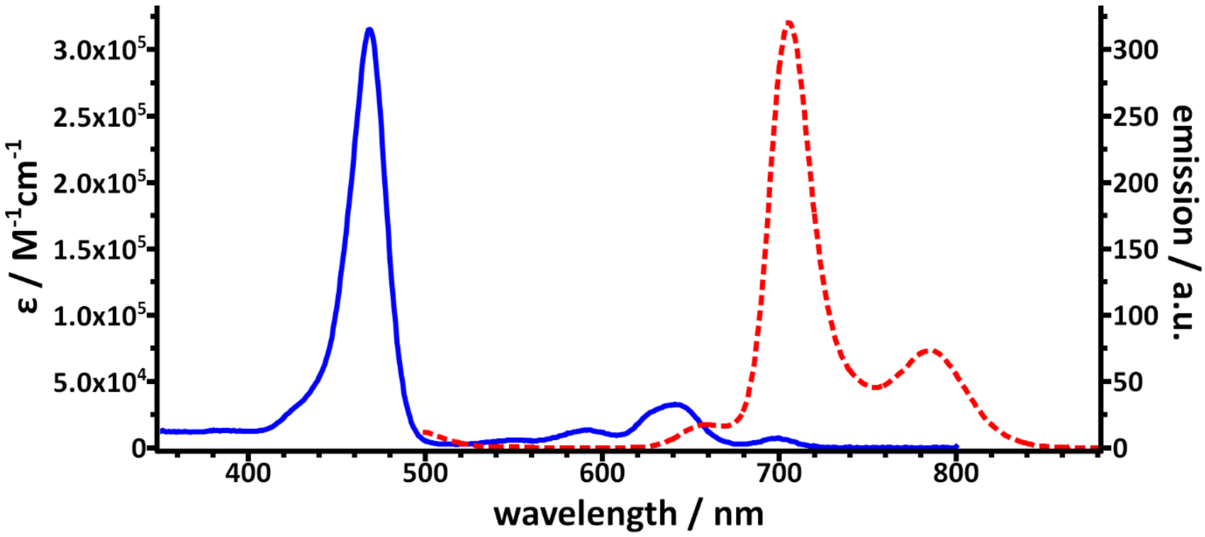

Figure S131. absorption (blue line) and emission spectrum of **29** (dashed red line; excitation at 468 nm) measured in CH_2Cl_2 + 1% NEt_3 at rt.

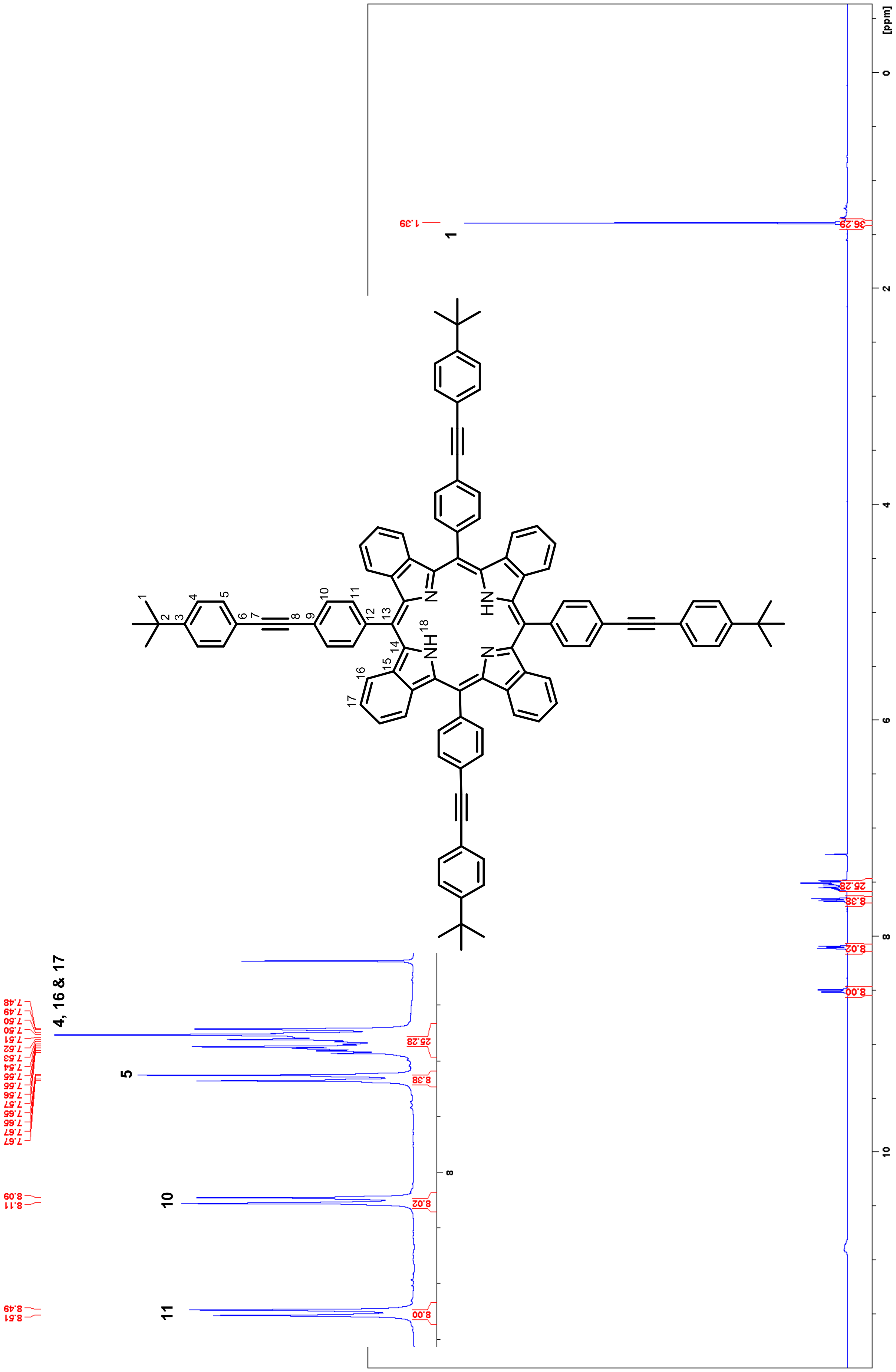

Figure S132. ^{1}H NMR (400 MHz, CDCl3/TFA-d$_1$, rt) of **30**.

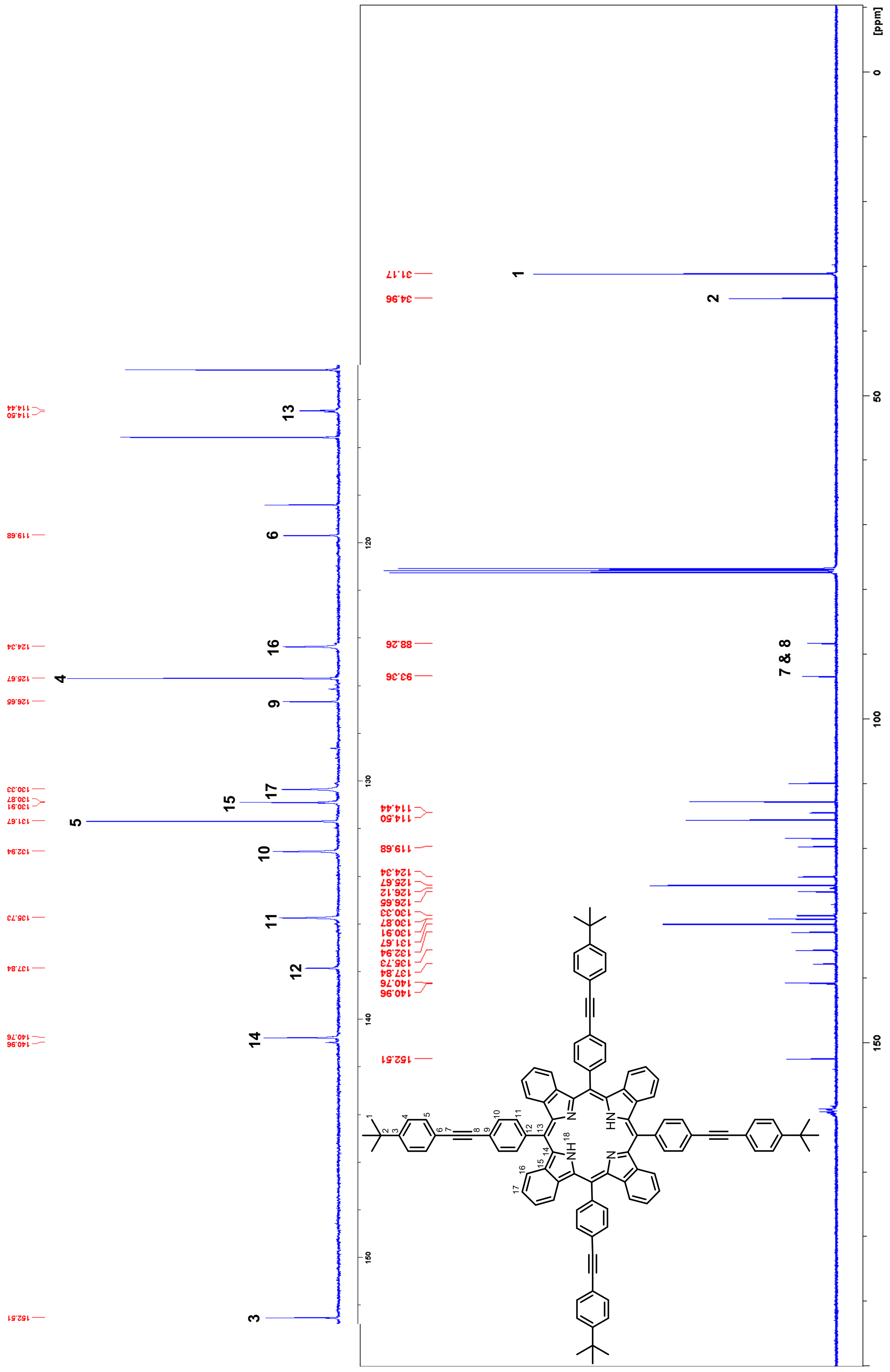

Figure S133. ^{13}C NMR (100 MHz, $CDCl_3$/TFA-d_1, rt) of **30**.

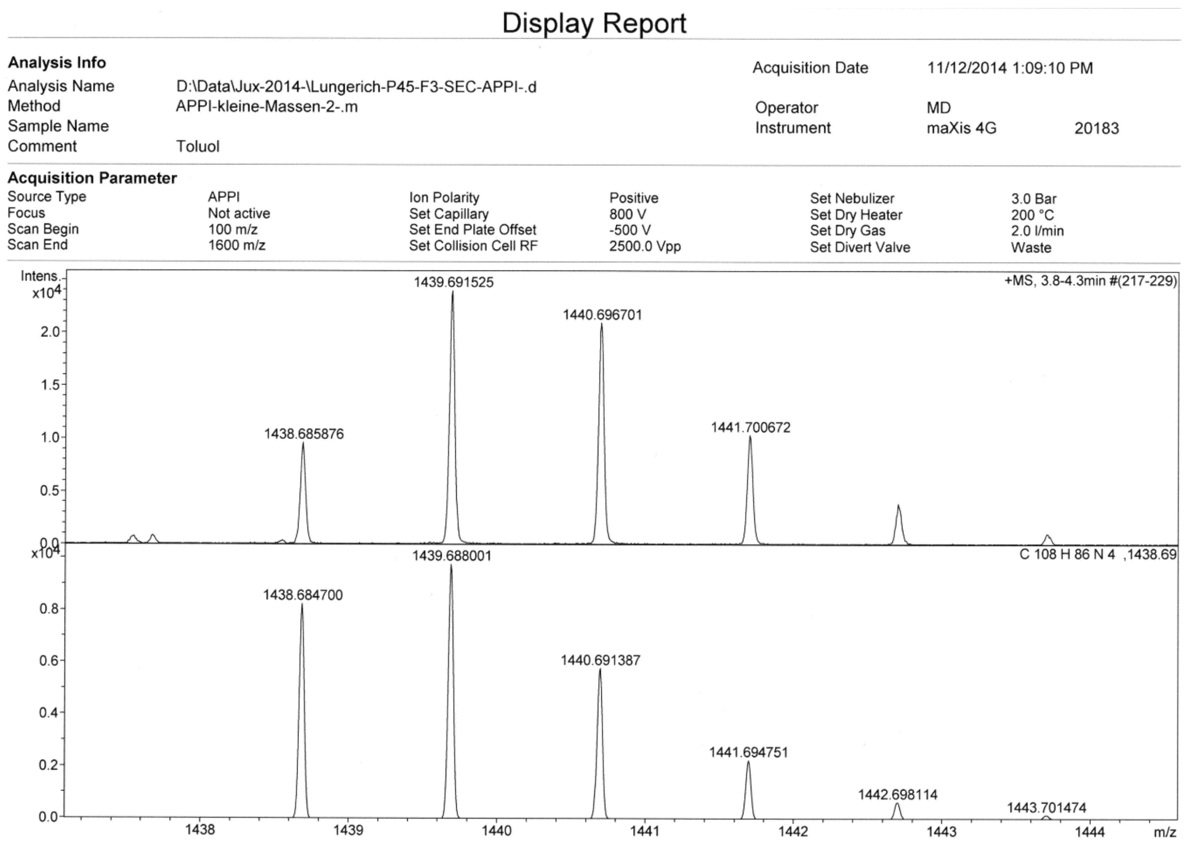

Figure S134. HRMS (APPI, toluene) of **30**.

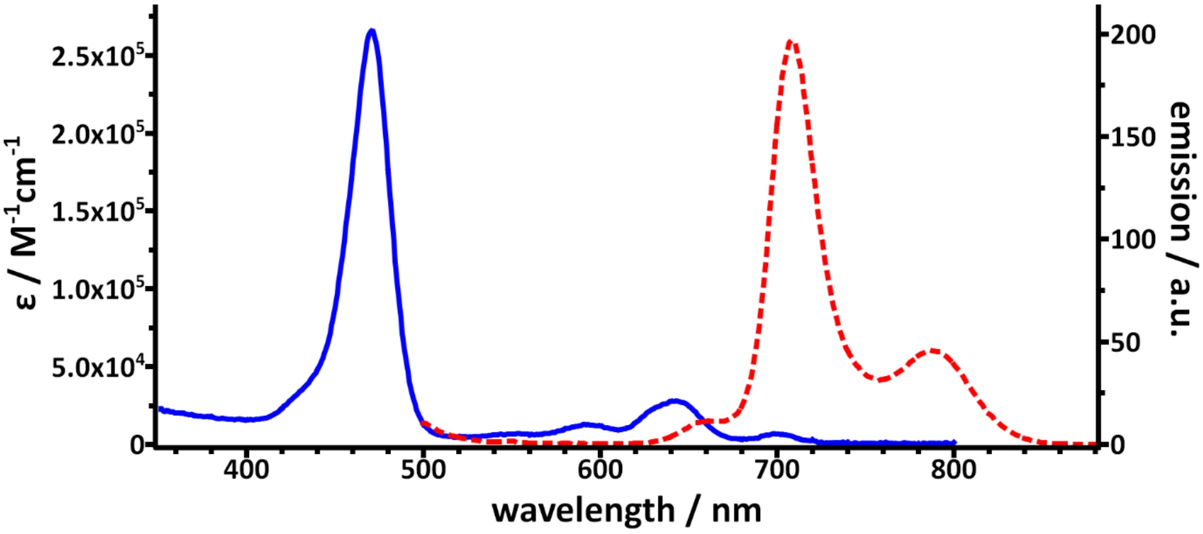

Figure S135. absorption (blue line) and emission spectrum of **30** (dashed red line; excitation at 471 nm) measured in CH_2Cl_2 + 1% NEt_3 at rt.

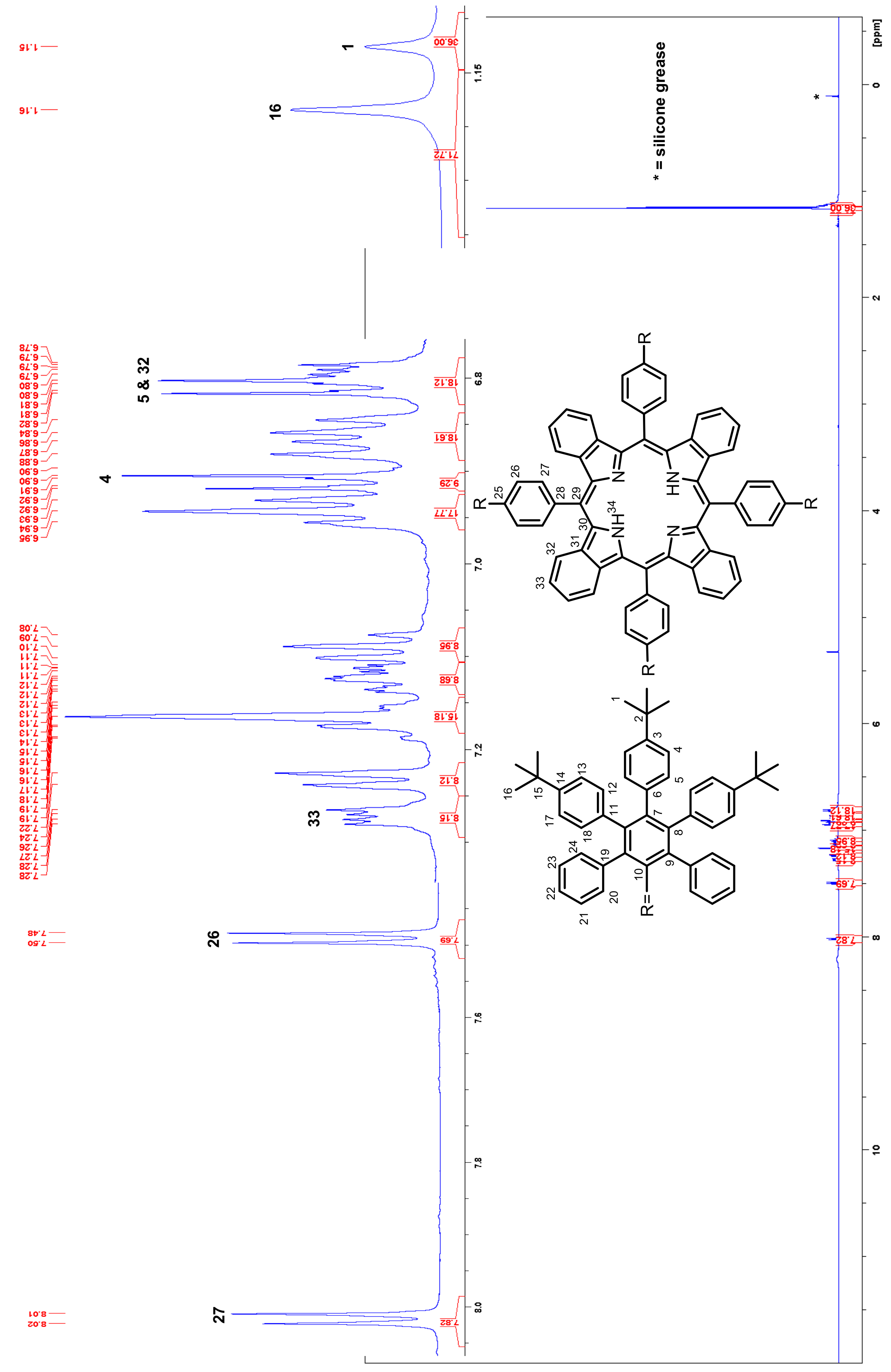

Figure S136. ^{1}H NMR (600 MHz, CD_2Cl_2/TFA-d_1, rt) of **31**.

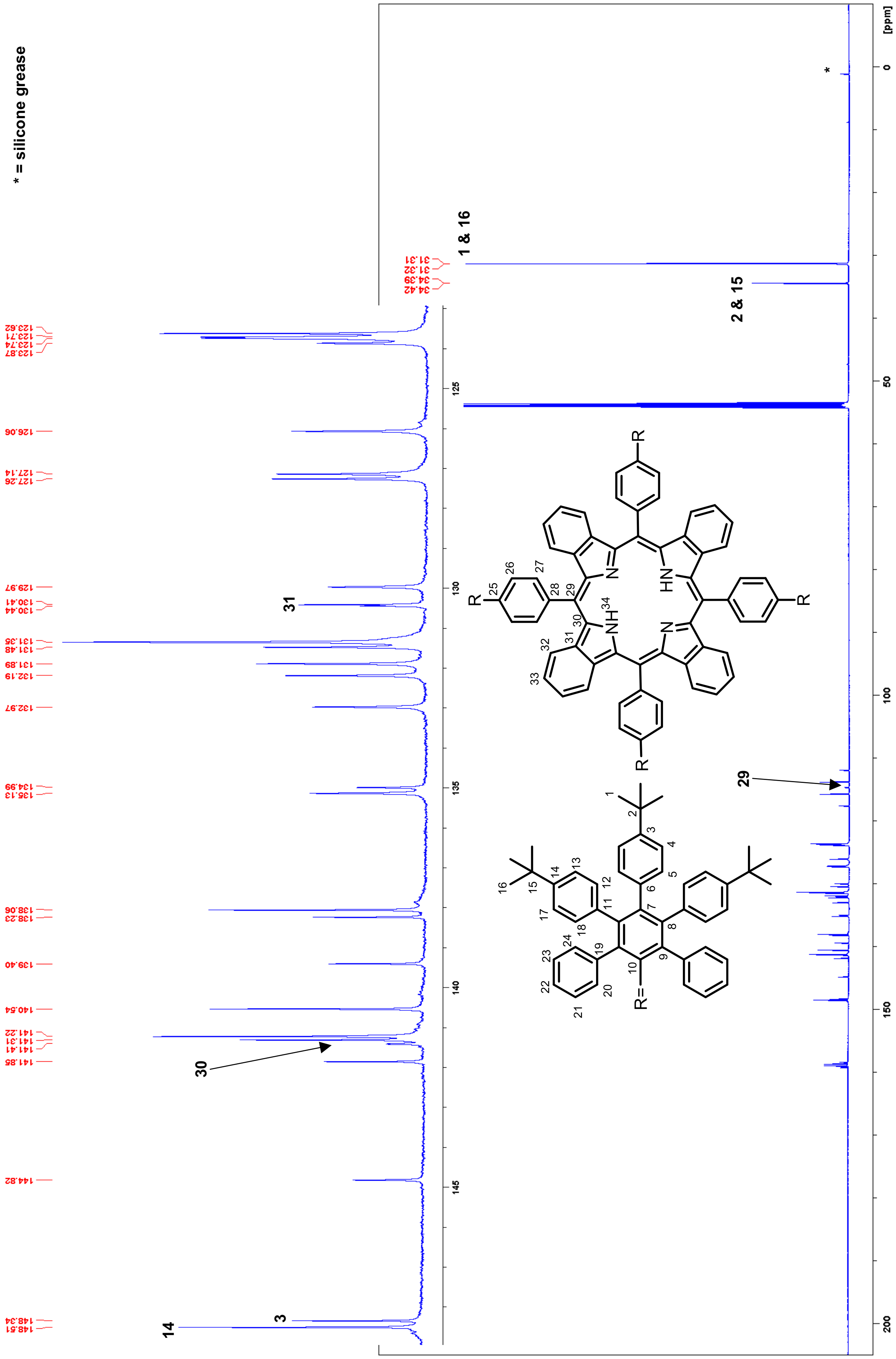

Figure S137. ^{13}C NMR (150 MHz, CD_2Cl_2/TFA-d_1, rt) of **31**.

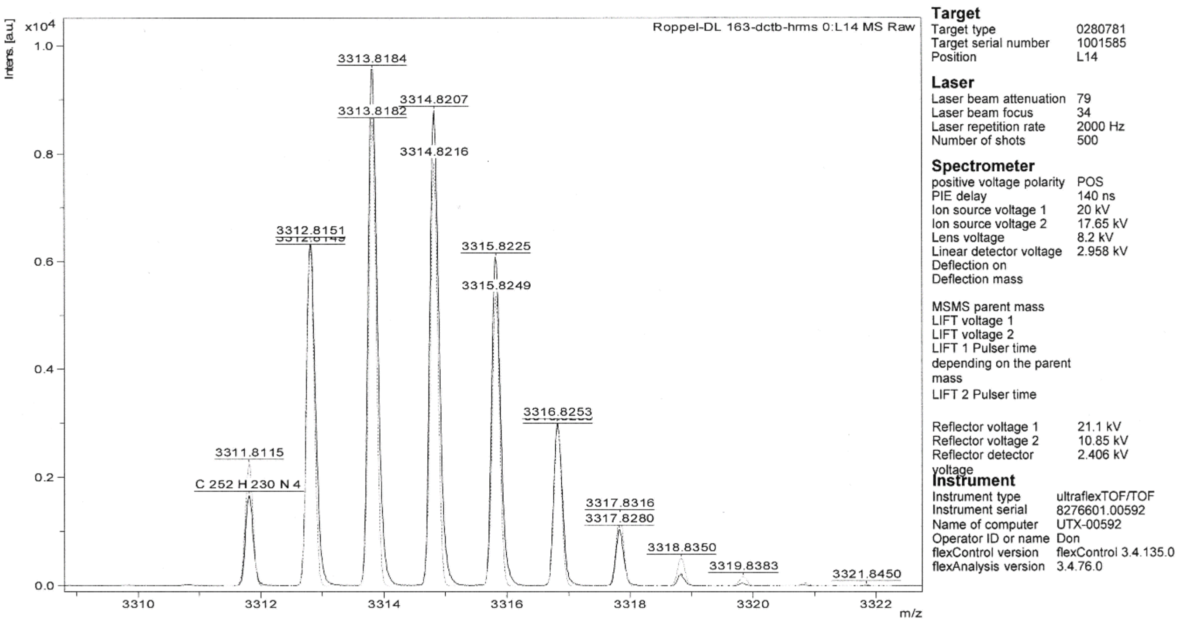

Figure S138. HRMS (MALDI) of **31**.

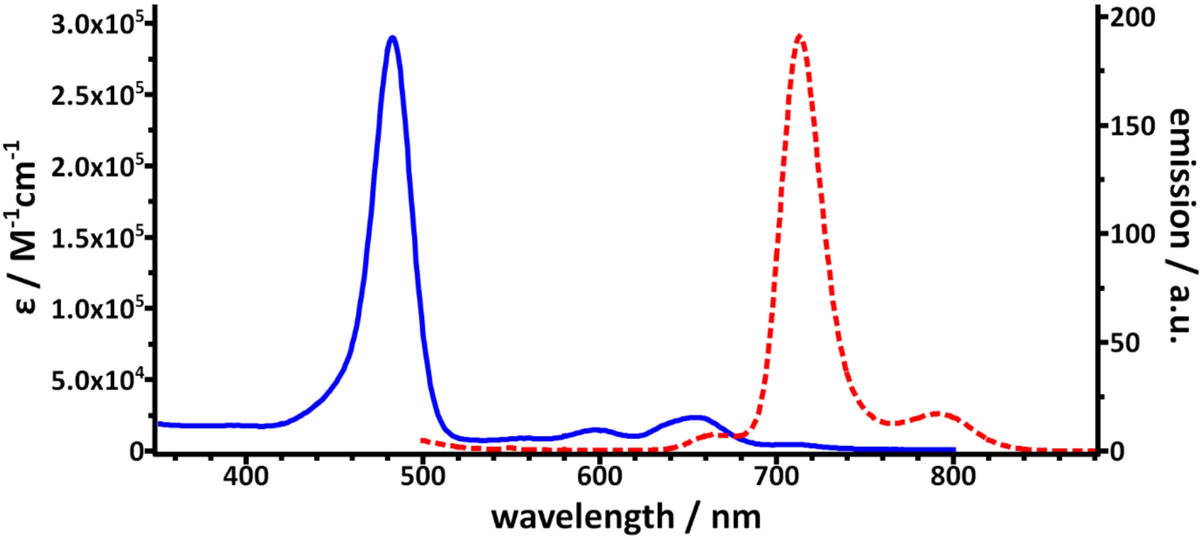

Figure S139. absorption (blue line) and emission spectrum of **31** (dashed red line; excitation at 468 nm) measured in CH_2Cl_2 + 1% NEt_3 at rt.

Table S7. UV-vis data for TATBP measured in CH_2Cl_2 + 1% NEt_3.

compound	Soret band / nm	Q bands[a] / nm	compound	Soret band / nm	Q bands[a] / nm
**2**	463	588 (0.04), 637 (0.11), 696 (0.03)	**17**	470	600 (0.8), 648 (0.18), 707 (0.07)
**3**	464	588 (0.04), 637 (0.10), 696 (0.02)	**18**	466	597 (0.07), 644 (0.17), 707 (0.07)
**4**	462	587 (0.04), 634 (0.11), 691 (0.03)	**19**	467	593 (0.05), 643 (0.12), 700 (0.03)
**5**	460	591 (0.05), 639 (0.14), 696 (0.06)	**20**	468	588 (0.04), 638 (0.09), 696 (0.02)
**6**	463	589 (0.04), 638 (0.11), 697 (0.03)	**21**	467	590 (0.04), 639 (0.09), 697 (0.02)
**7**	457	593 (0.06), 641 (0.17), 699 (0.09)	**22**	459	588 (0.05), 633 (0.14), 695 (0.04)
**8**	463	593 (0.05), 641 (0.14), 698 (0.05)	**23**	463	589 (0.04), 636 (0.11), 694 (0.03)
**9**	463	593 (0.05), 642 (0.14), 699 (0.05)	**24**	463	588 (0.04), 636 (0.12), 693 (0.03)
**10**	458	598 (0.06), 647 (0.21), 704 (0.14)	**25**	461	594 (0.06), 643 (0.18), 700 (0.09)
**11**	460	593 (0.06), 642 (0.17), 700 (0.09)	**26**	469	596 (0.04), 645 (0.11), 706 (0.04)
**12**	463	596 (0.06), 645 (0.17), 703 (0.09)	**27**	460	590 (0.05), 638 (0.15), 694 (0.06)
**13**	464	593 (0.05), 642 (0.14), 700 (0.06)	**28**	470	593 (0.04), 643 (0.11), 700 (0.02)
**14**	468	600 (0.05), 647 (0.17), 706 (0.09)	**29**	468	590 (0.04), 641 (0.10), 698 (0.02)
**15**	464	593 (0.04), 642 (0.12), 700 (0.04)	**30**	471	593 (0.05), 643 (0.10), 699 (0.03)
**16**	468	596 (0.04), 645 (0.13), 702 (0.04)	**31**	483	598 (0.05), 654 (0.08), 707 (0.02)

[a] Numbers in parentheses show the relative intensities of Q-bands normalized by intensities of the Soret bands.

7 SPECTRAL APPENDIX (CYCLIC VOLTAMMOGRAMS, DPV)

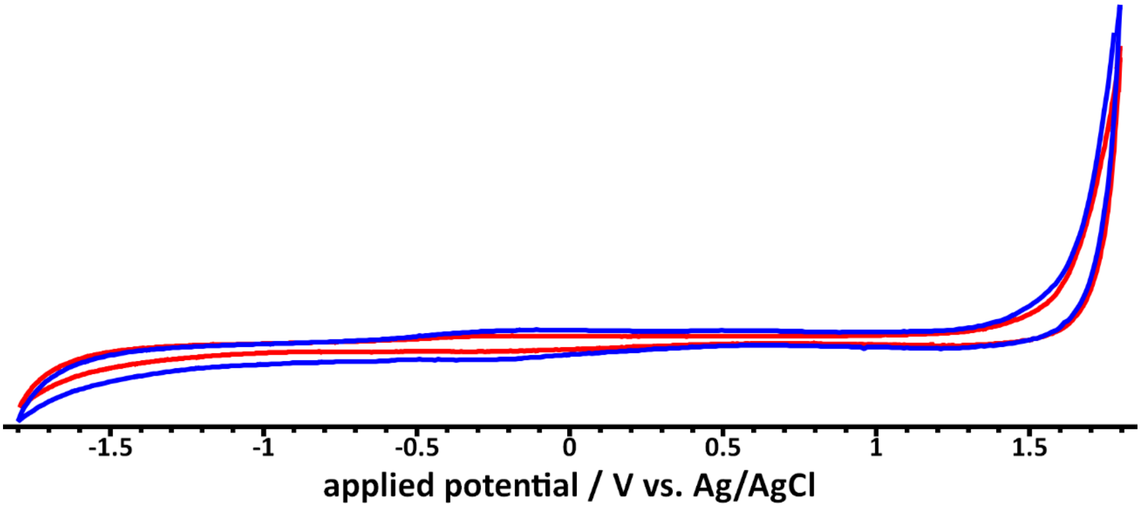

Figure S140. Cyclic voltammogram of CH_2Cl_2 containing 0.1 M $TBAPF_6$ with a scan rate of 100 mV/s (blue line) or 50 mV/s (red line).

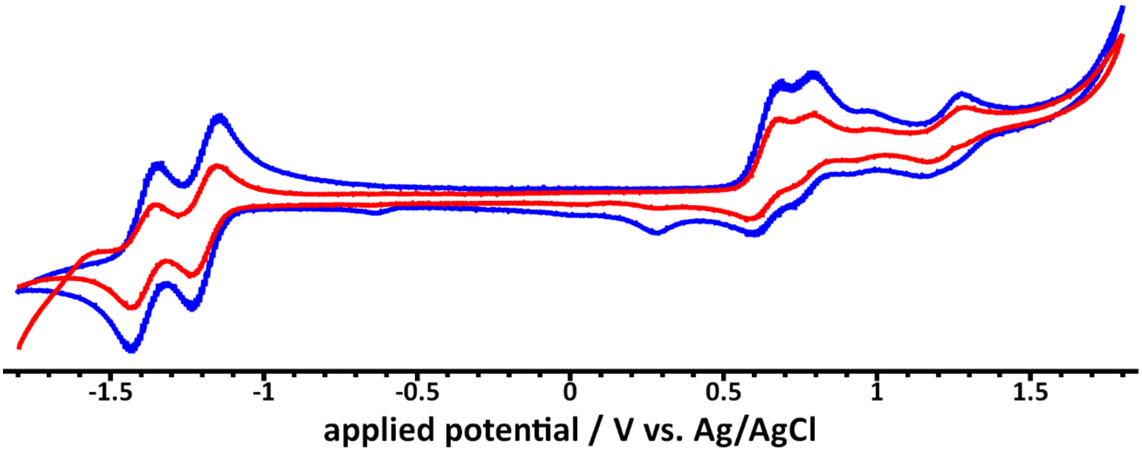

Figure S141. Cyclic voltammogram of **2** in CH_2Cl_2 containing 0.1 M $TBAPF_6$ with a scan rate of 100 mV/s (blue line) or 50 mV/s (red line).

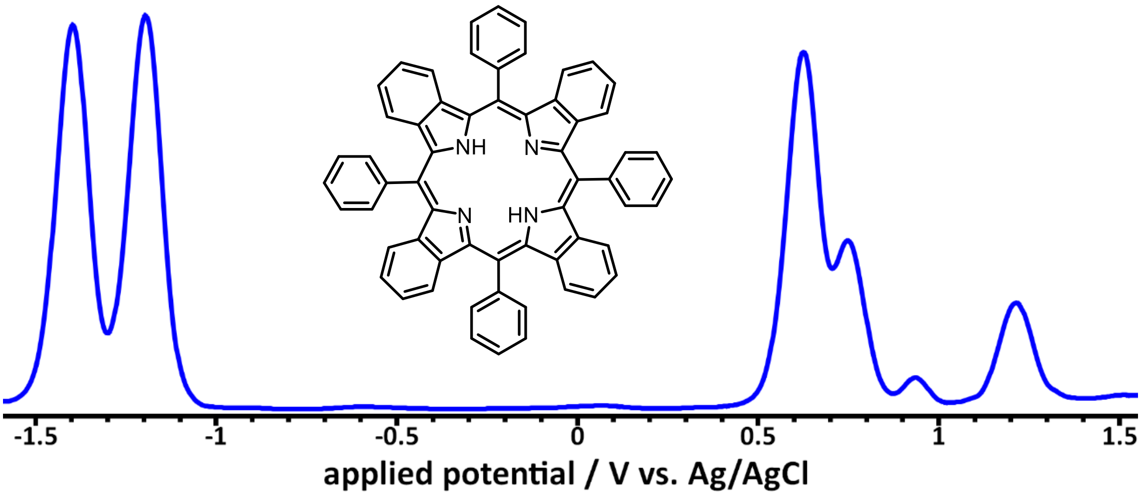

Figure S142. Differential pulse voltammogram of **2** in CH_2Cl_2 containing 0.1 M $TBAPF_6$ with a scan rate of 10 mV/s.

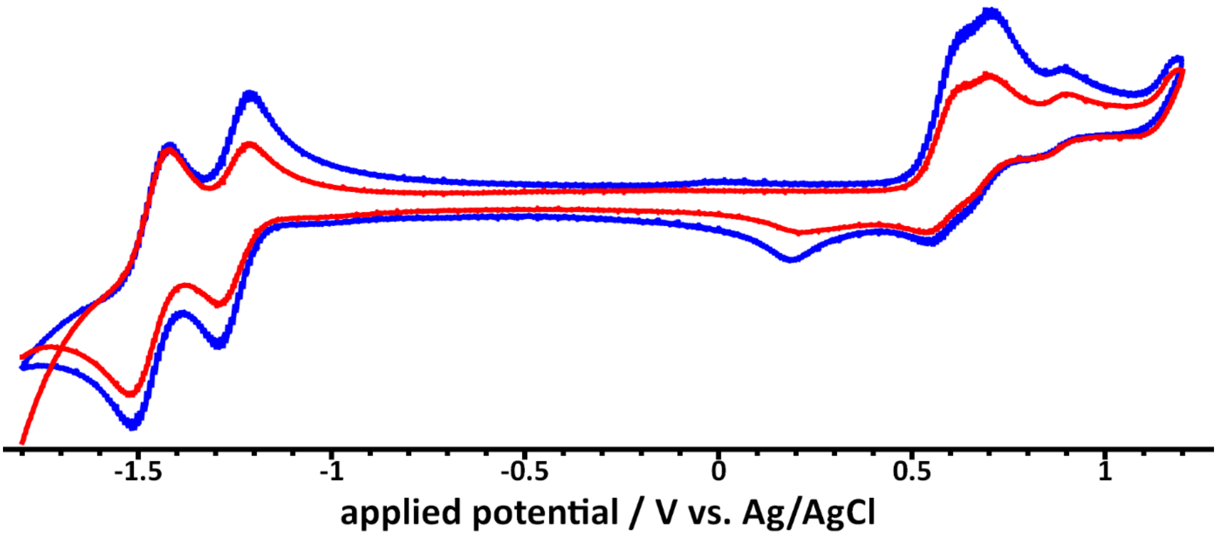

Figure S143. Cyclic voltammogram of **3** in CH_2Cl_2 containing 0.1 M $TBAPF_6$ with a scan rate of 100 mV/s (blue line) or 50 mV/s (red line).

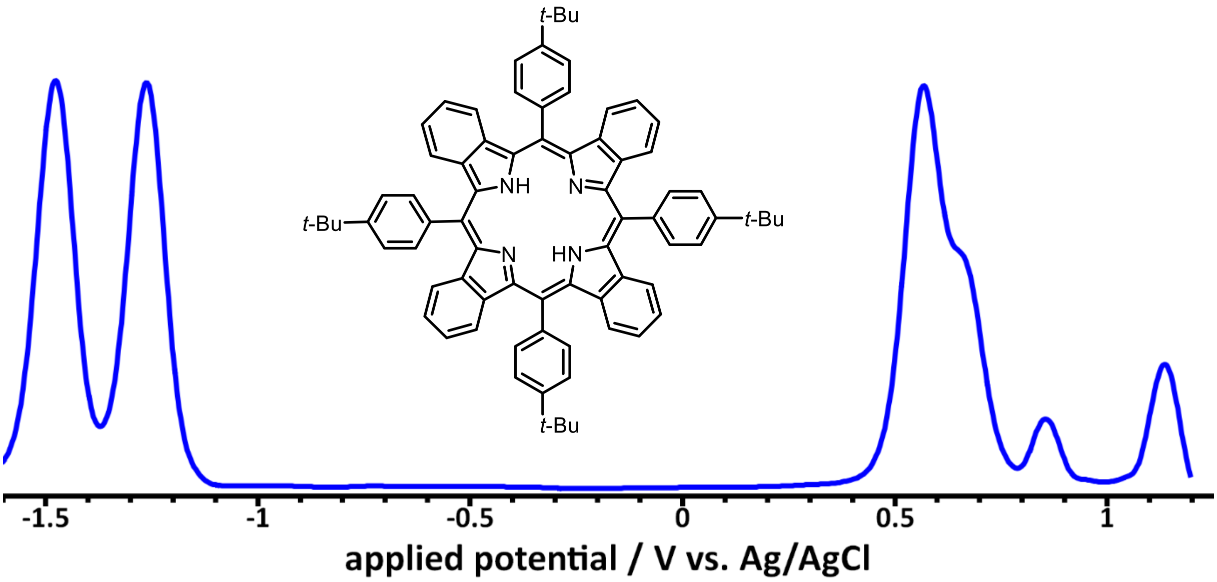

Figure S144. Differential pulse voltammogram of **3** in CH_2Cl_2 containing 0.1 M $TBAPF_6$ with a scan rate of 10 mV/s.

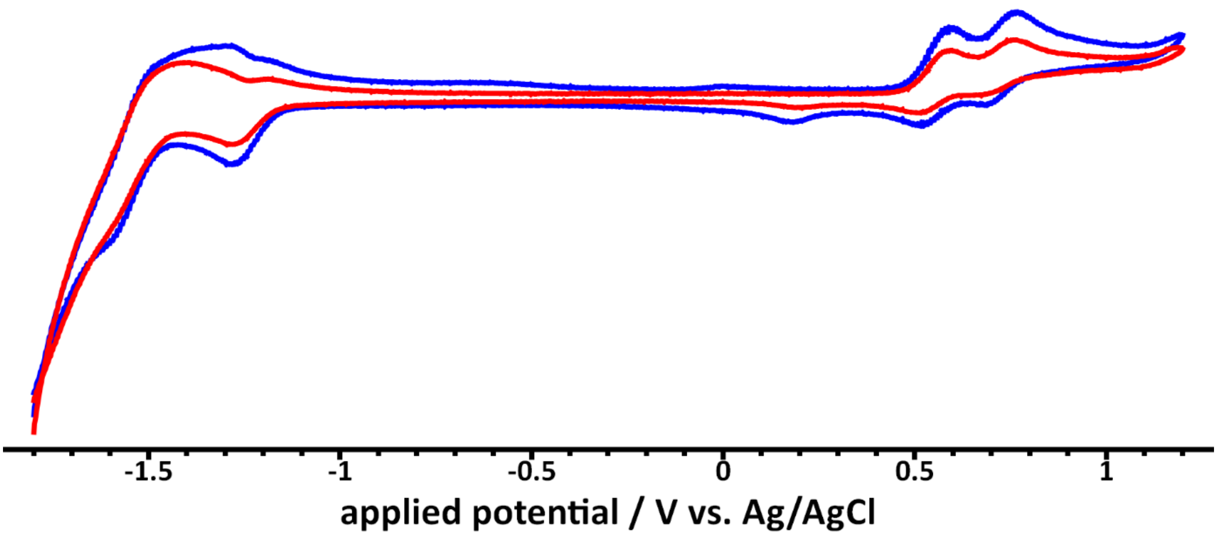

Figure S145. Cyclic voltammogram of **4** in CH_2Cl_2 containing 0.1 M $TBAPF_6$ with a scan rate of 100 mV/s (blue line) or 50 mV/s (red line).

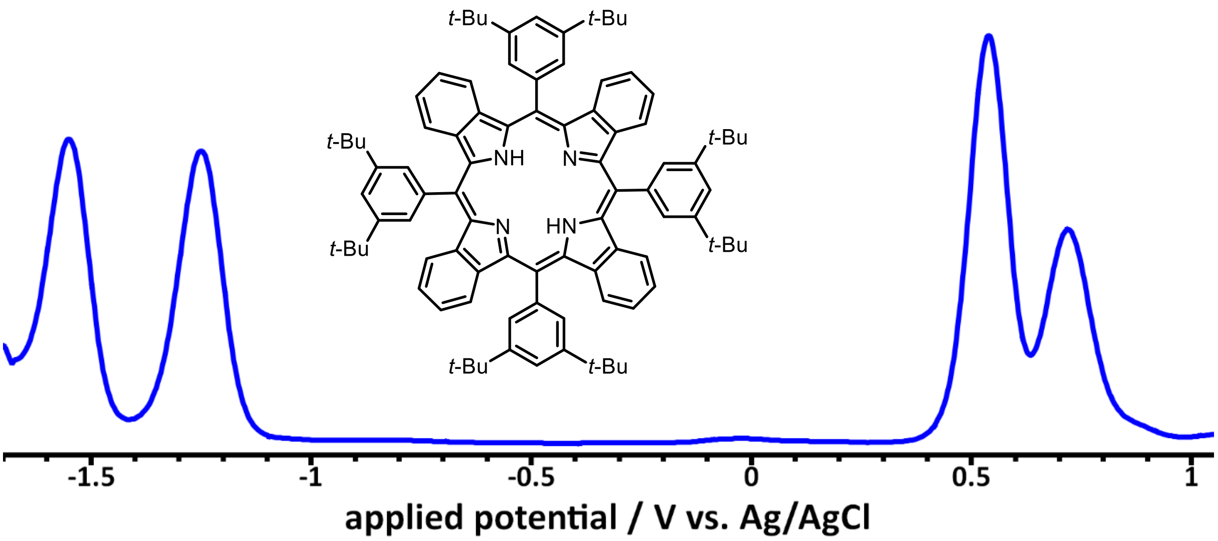

Figure S146. Differential pulse voltammogram of **4** in CH_2Cl_2 containing 0.1 M $TBAPF_6$ with a scan rate of 10 mV/s.

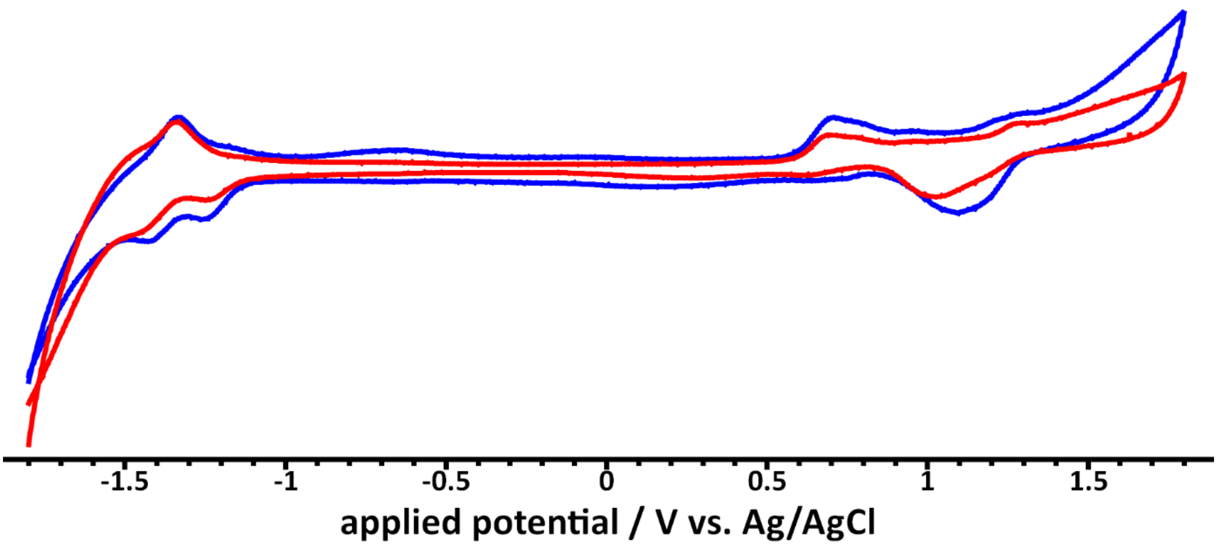

Figure S147. Cyclic voltammogram of **6** in CH_2Cl_2 containing 0.1 M $TBAPF_6$ with a scan rate of 100 mV/s (blue line) or 50 mV/s (red line).

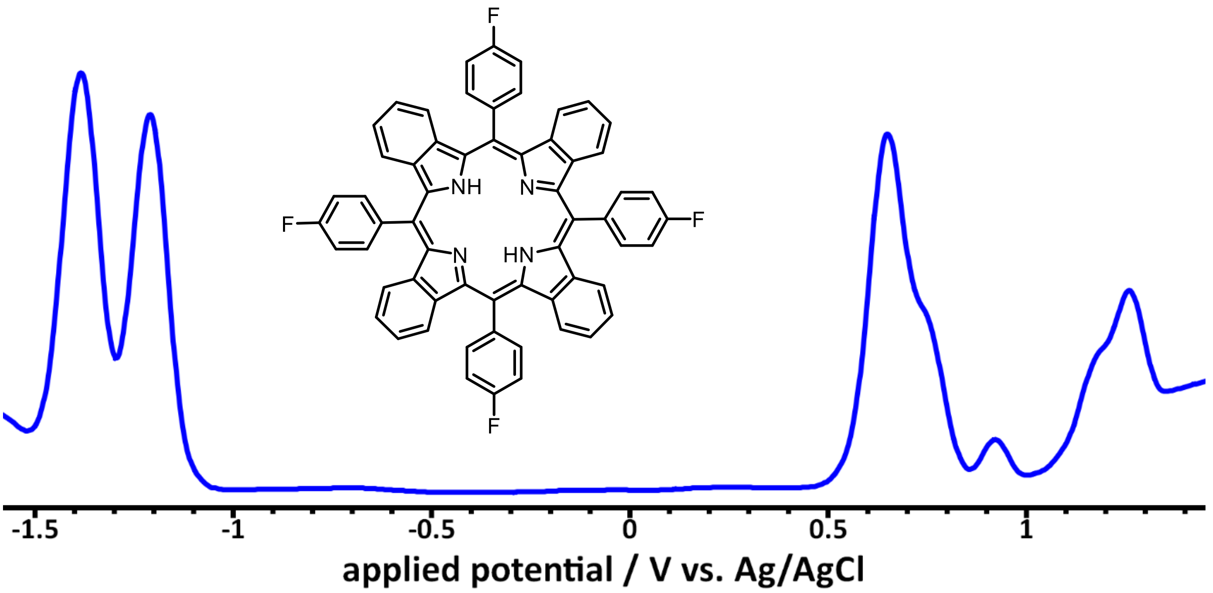

Figure S148. Differential pulse voltammogram of **6** in CH_2Cl_2 containing 0.1 M $TBAPF_6$ with a scan rate of 10 mV/s.

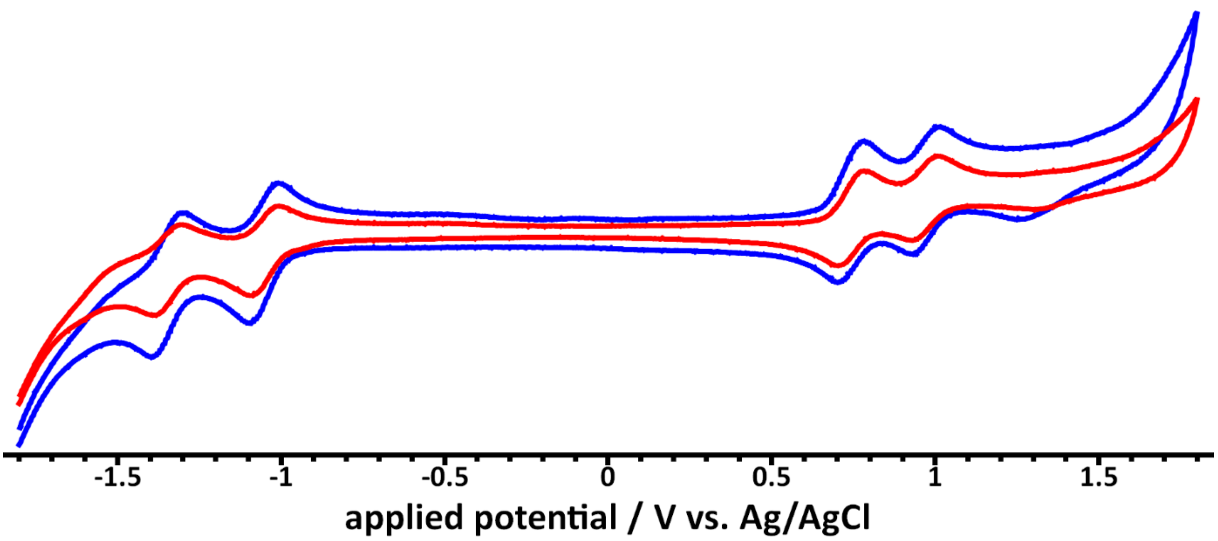

Figure S149. Cyclic voltammogram of **7** in CH_2Cl_2 containing 0.1 M $TBAPF_6$ with a scan rate of 100 mV/s (blue line) or 50 mV/s (red line).

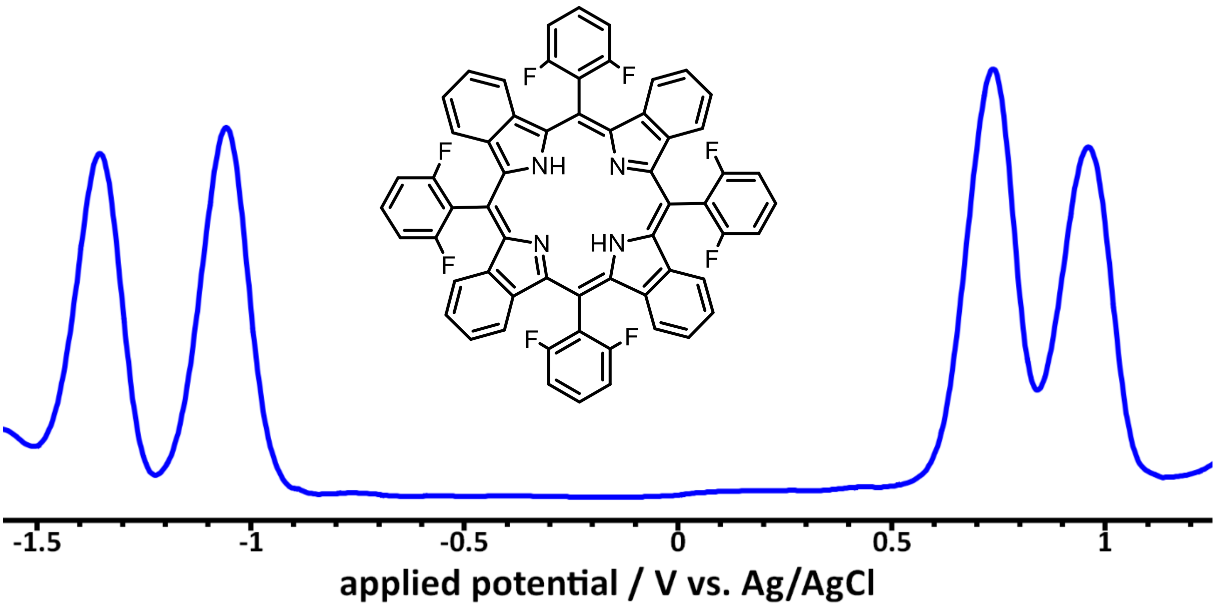

Figure S150. Differential pulse voltammogram of **7** in CH_2Cl_2 containing 0.1 M $TBAPF_6$ with a scan rate of 10 mV/s.

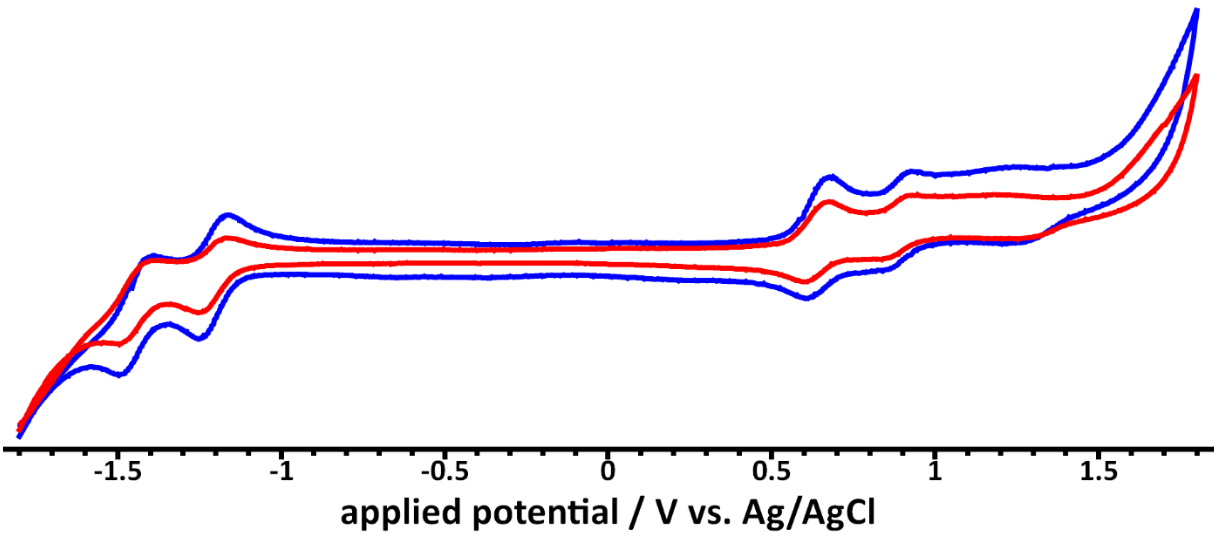

Figure S151. Cyclic voltammogram of **8** in CH_2Cl_2 containing 0.1 M $TBAPF_6$ with a scan rate of 100 mV/s (blue line) or 50 mV/s (red line).

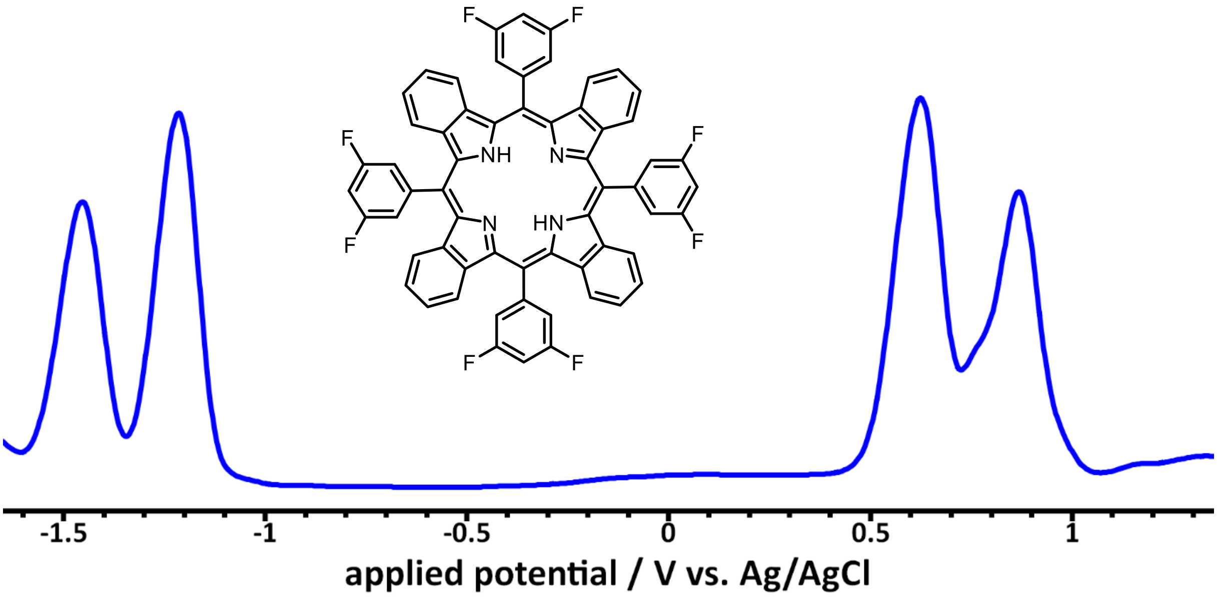

Figure S152. Differential pulse voltammogram of **8** in CH_2Cl_2 containing 0.1 M $TBAPF_6$ with a scan rate of 10 mV/s.

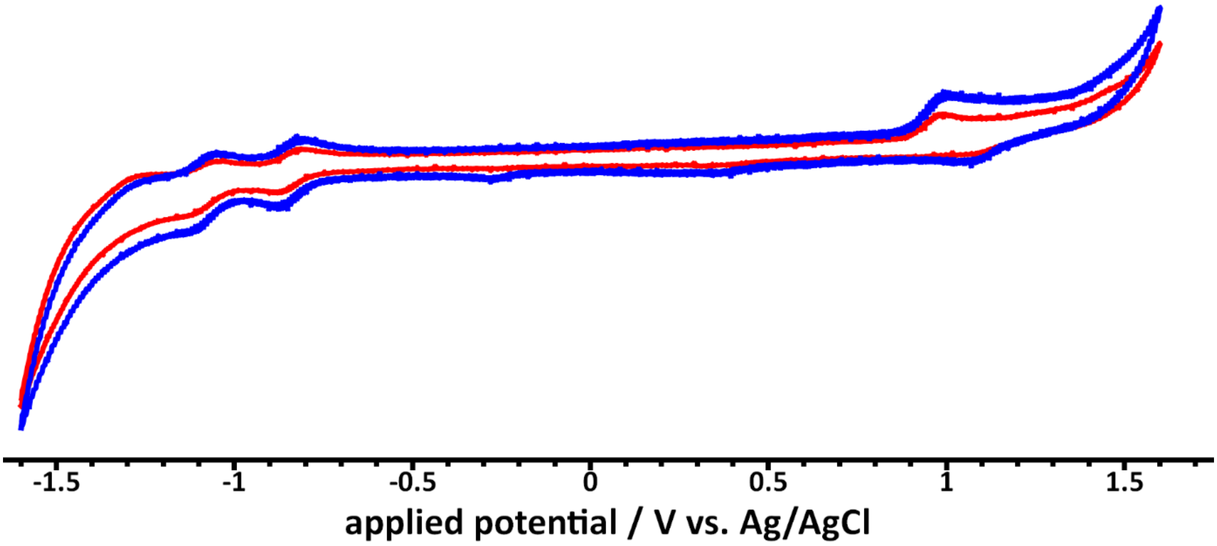

Figure S153. Cyclic voltammogram of **10** in CH_2Cl_2 containing 0.1 M $TBAPF_6$ with a scan rate of 100 mV/s (blue line) or 50 mV/s (red line).

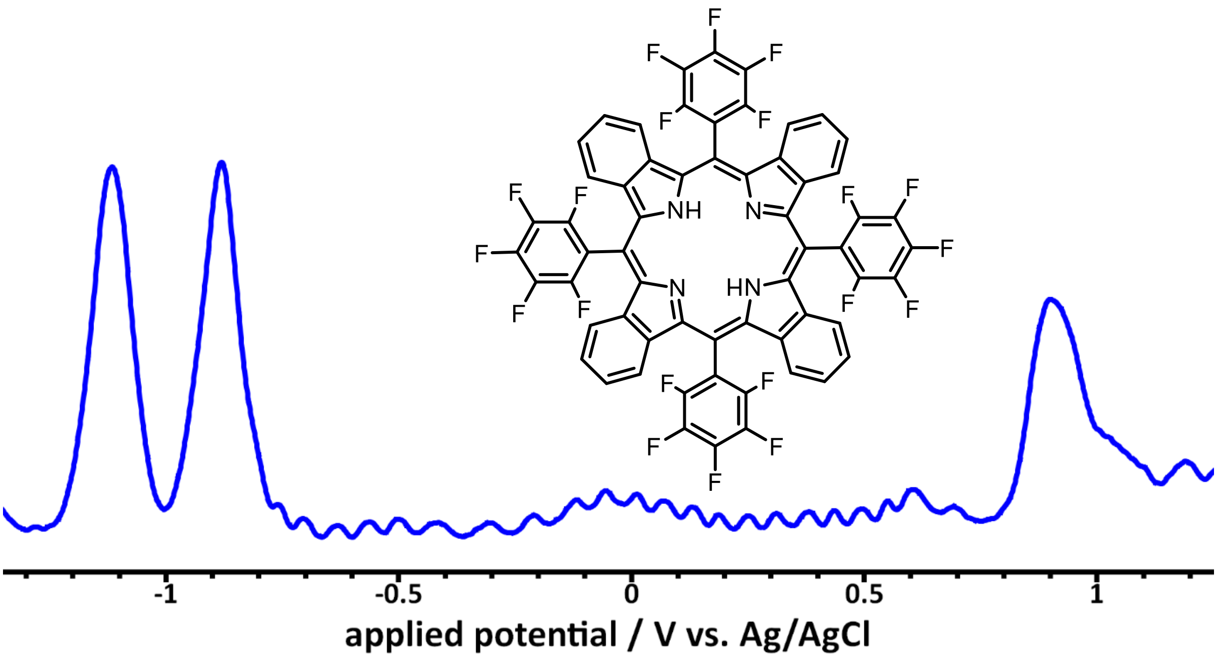

Figure S154. Differential pulse voltammogram of **10** in CH_2Cl_2 containing 0.1 M $TBAPF_6$ with a scan rate of 10 mV/s.

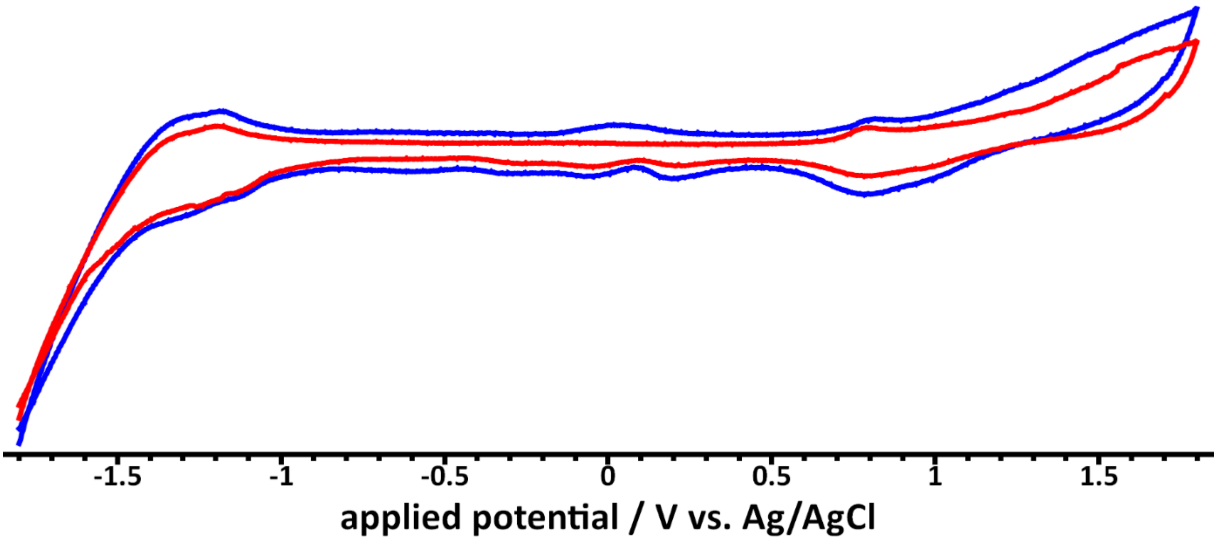

Figure S155. Cyclic voltammogram of **16** in CH_2Cl_2 containing 0.1 M $TBAPF_6$ with a scan rate of 100 mV/s (blue line) or 50 mV/s (red line).

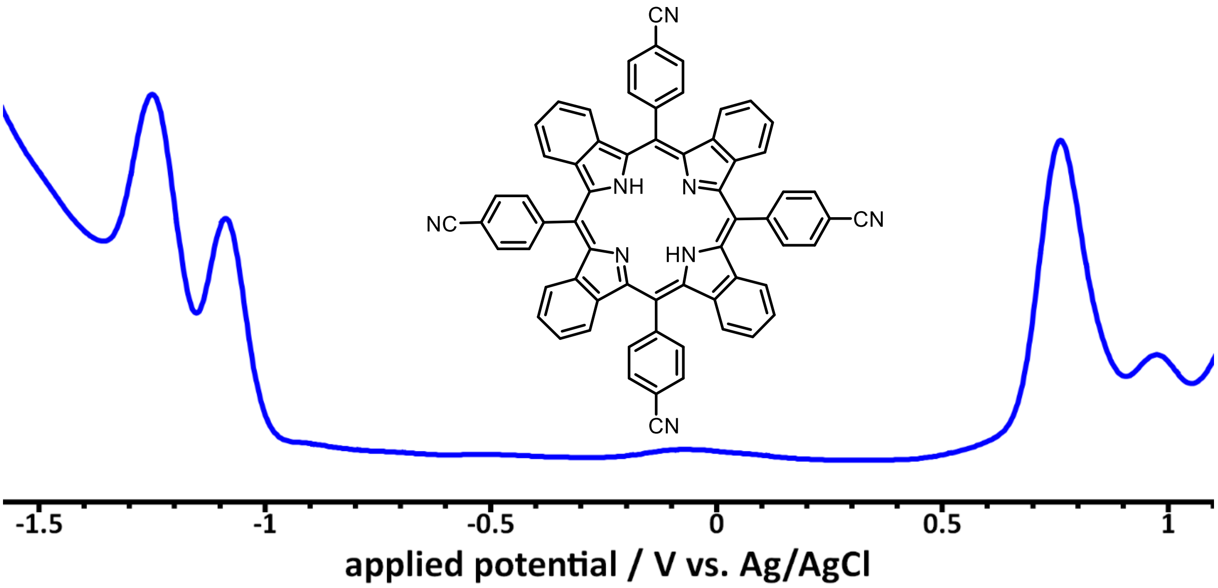

Figure S156. Differential pulse voltammogram of **16** in CH_2Cl_2 containing 0.1 M $TBAPF_6$ with a scan rate of 10 mV/s.

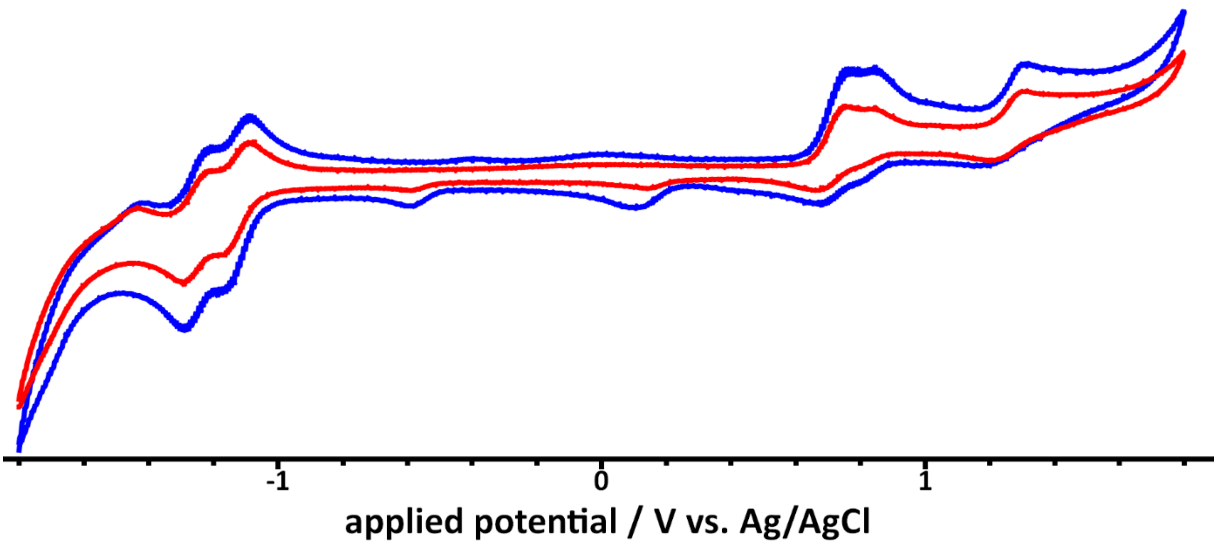

Figure S157. Cyclic voltammogram of **19** in CH_2Cl_2 containing 0.1 M $TBAPF_6$ with a scan rate of 100 mV/s (blue line) or 50 mV/s (red line).

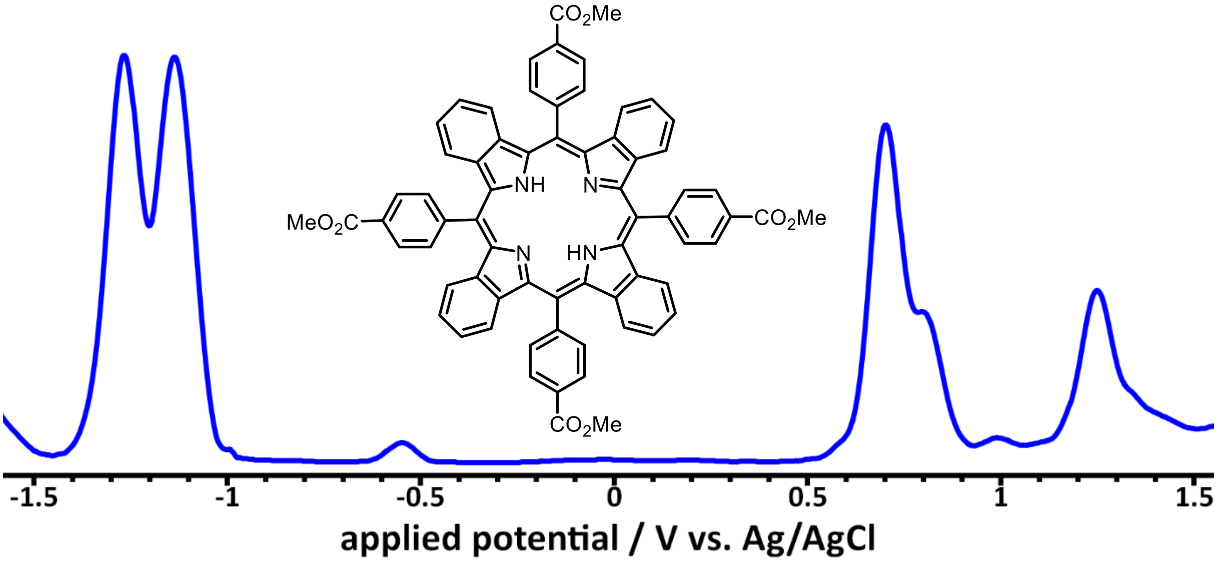

Figure S158. Differential pulse voltammogram of **19** in CH_2Cl_2 containing 0.1 M $TBAPF_6$ with a scan rate of 10 mV/s.

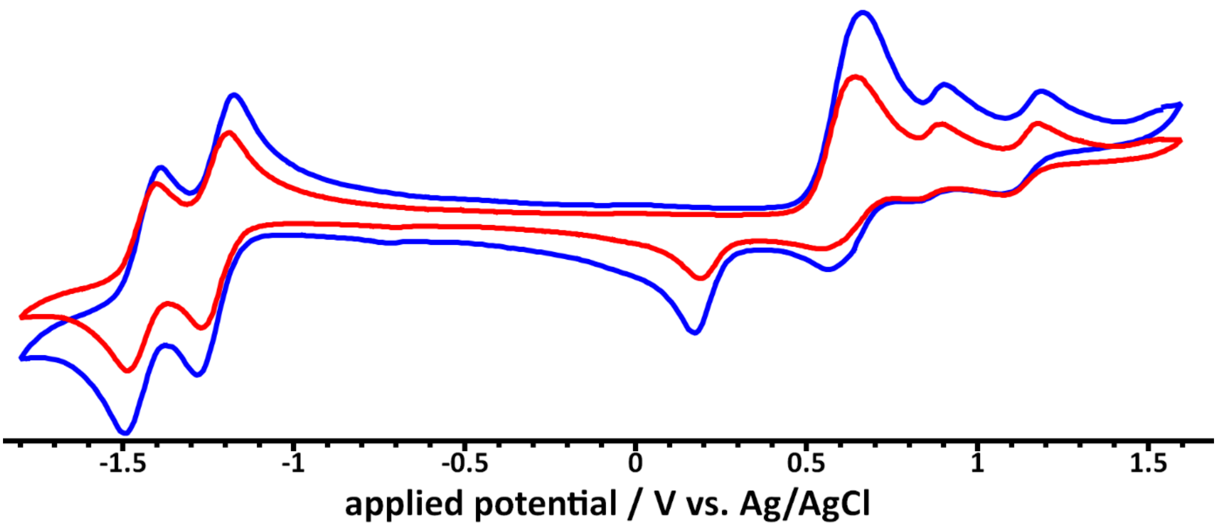

Figure S159. Cyclic voltammogram of **21** in CH_2Cl_2 containing 0.1 M $TBAPF_6$ with a scan rate of 100 mV/s (blue line) or 50 mV/s (red line).

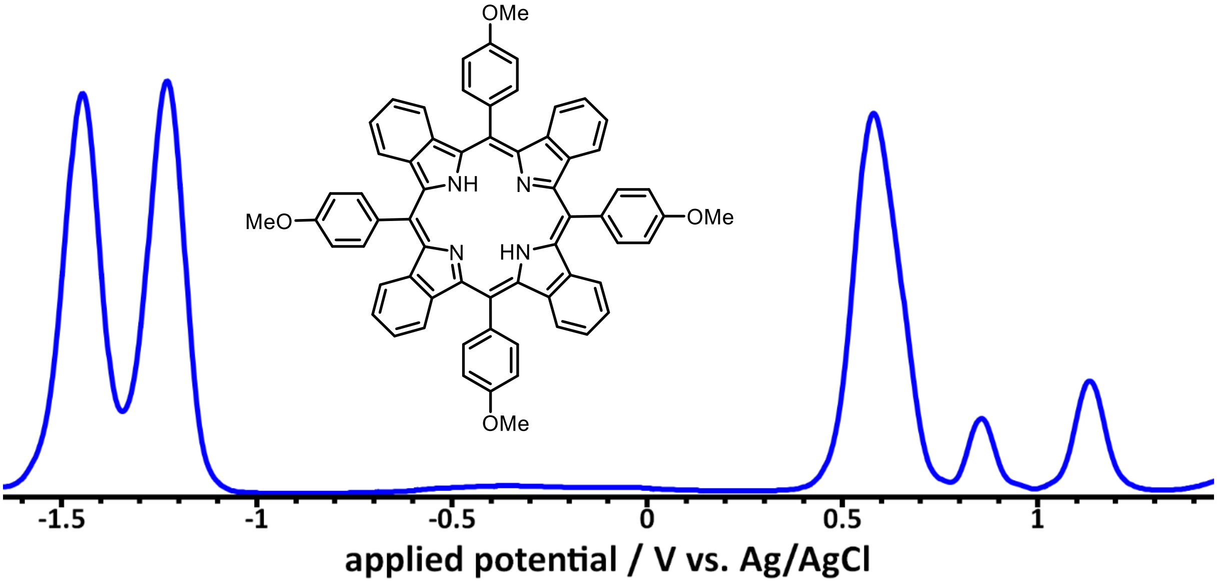

Figure S160. Differential pulse voltammogram of **21** in CH_2Cl_2 containing 0.1 M $TBAPF_6$ with a scan rate of 10 mV/s.

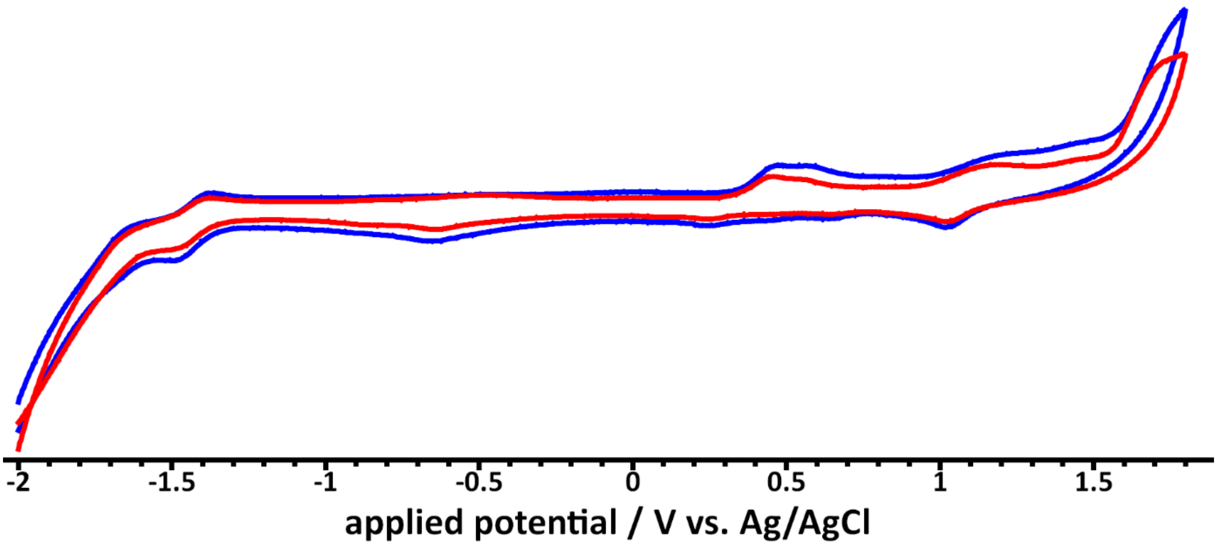

Figure S161. Cyclic voltammogram of **22** in CH_2Cl_2 containing 0.1 M $TBAPF_6$ with a scan rate of 100 mV/s (blue line) or 50 mV/s (red line).

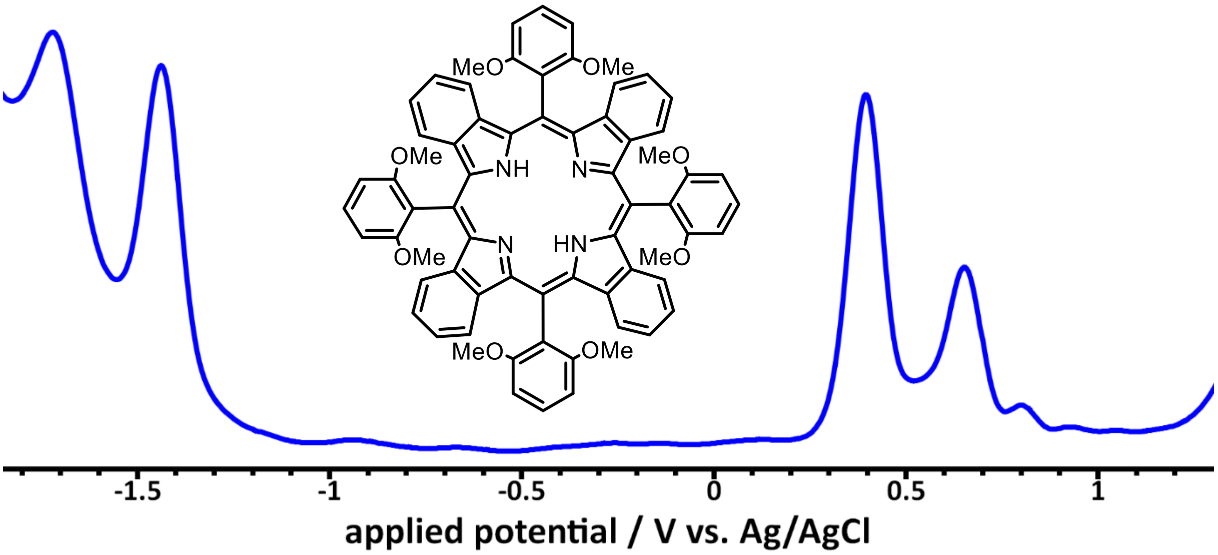

Figure S162. Differential pulse voltammogram of **22** in CH_2Cl_2 containing 0.1 M $TBAPF_6$ with a scan rate of 10 mV/s.

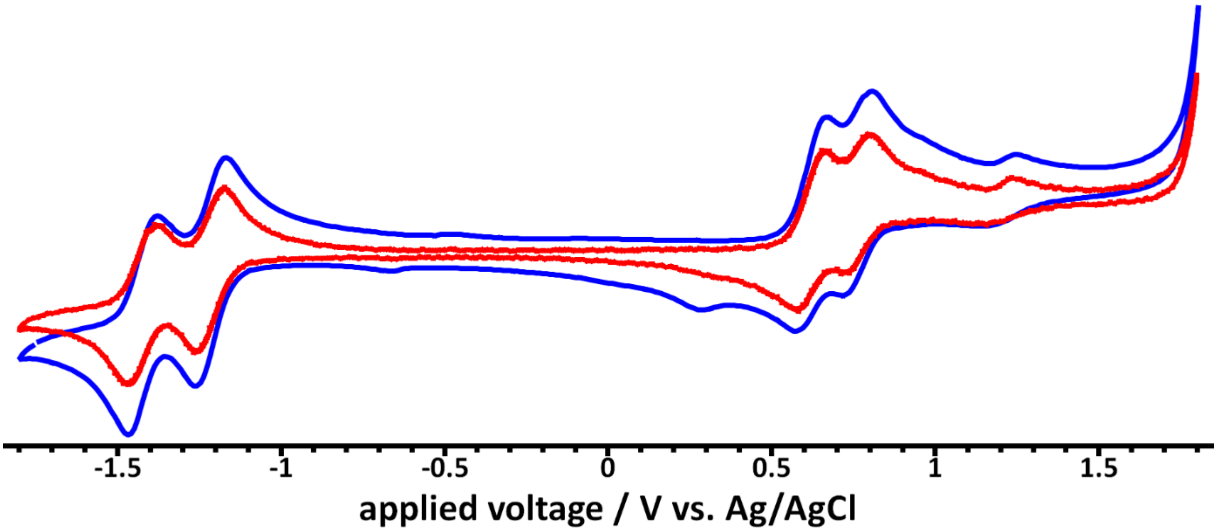

Figure S163. Cyclic voltammogram of **23** in CH_2Cl_2 containing 0.1 M $TBAPF_6$ with a scan rate of 100 mV/s (blue line) or 50 mV/s (red line).

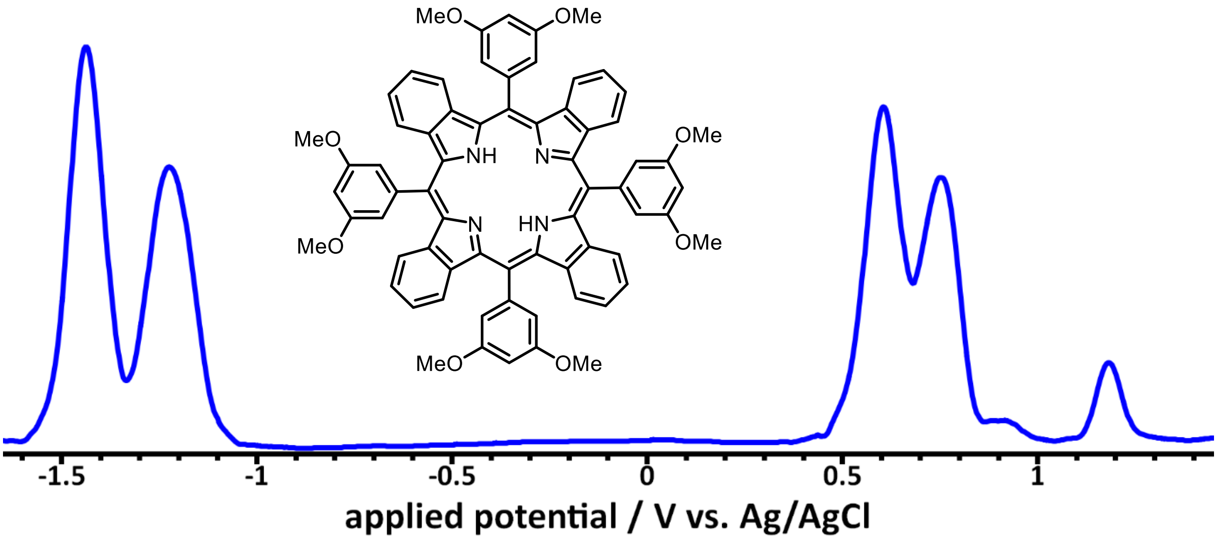

Figure S164. Differential pulse voltammogram of **23** in CH_2Cl_2 containing 0.1 M $TBAPF_6$ with a scan rate of 10 mV/s.

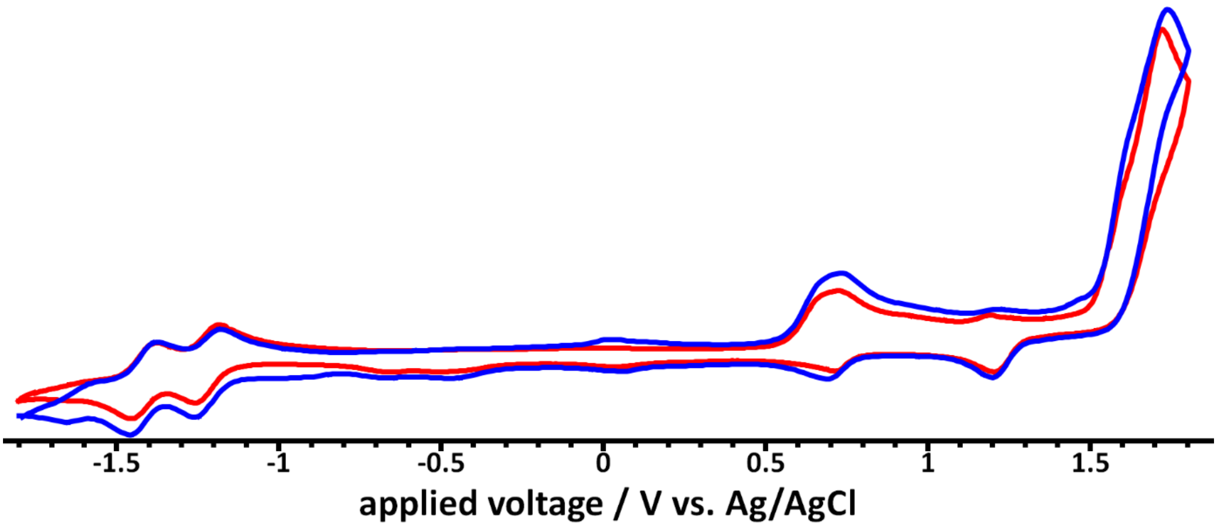

Figure S165. Cyclic voltammogram of **24** in CH_2Cl_2 containing 0.1 M $TBAPF_6$ with a scan rate of 100 mV/s (blue line) or 50 mV/s (red line).

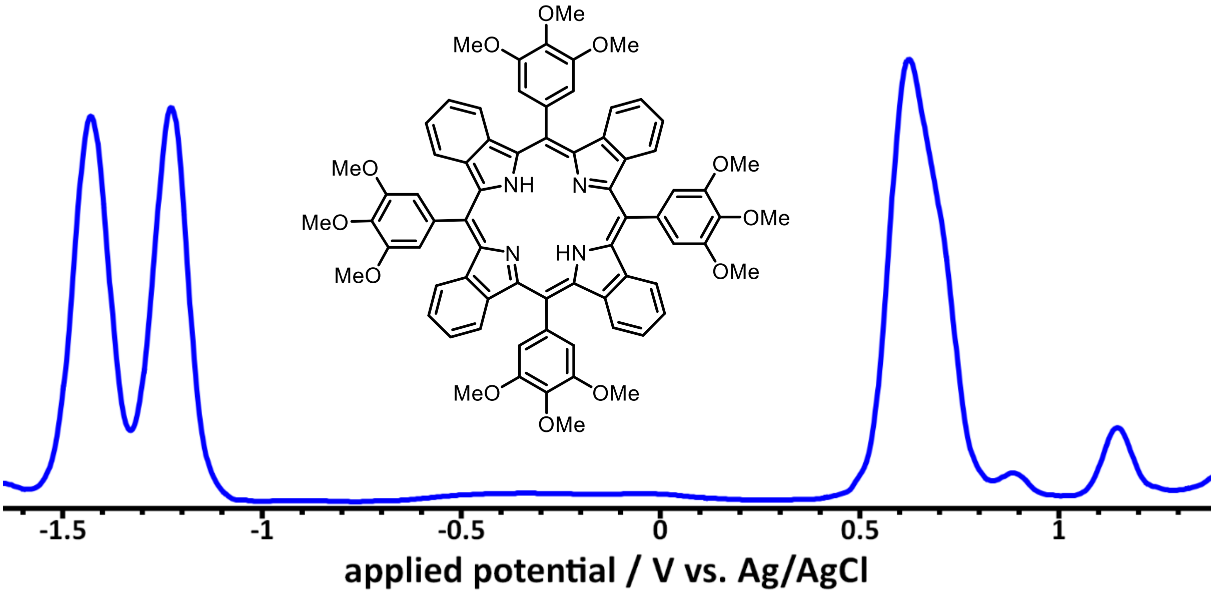

Figure S166. Differential pulse voltammogram of **24** in CH_2Cl_2 containing 0.1 M $TBAPF_6$ with a scan rate of 10 mV/s.

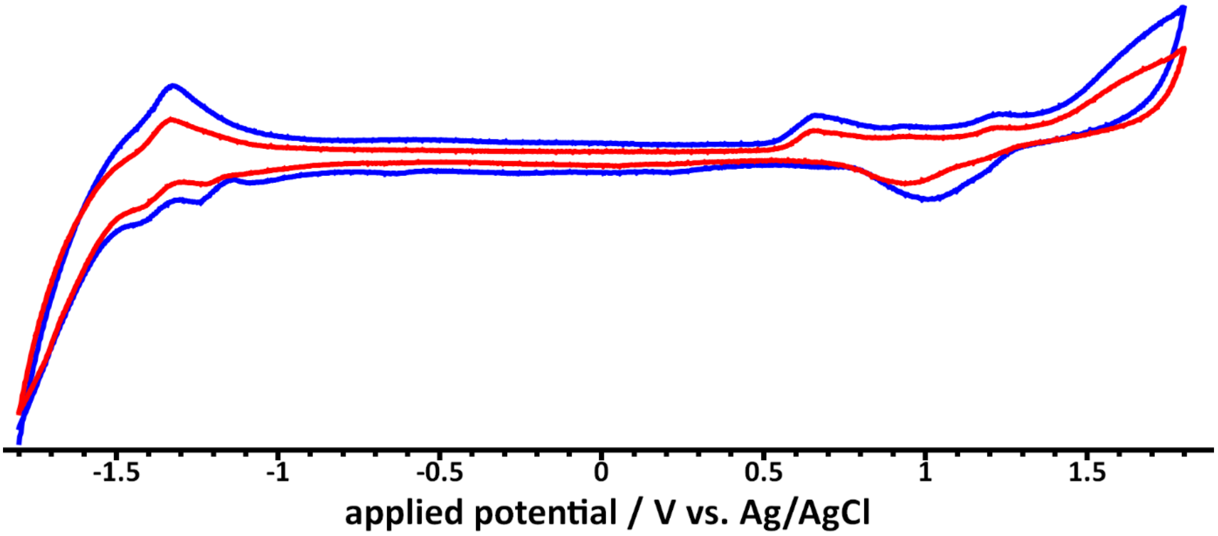

Figure S167. Cyclic voltammogram of **29** in CH_2Cl_2 containing 0.1 M $TBAPF_6$ with a scan rate of 100 mV/s (blue line) or 50 mV/s (red line).

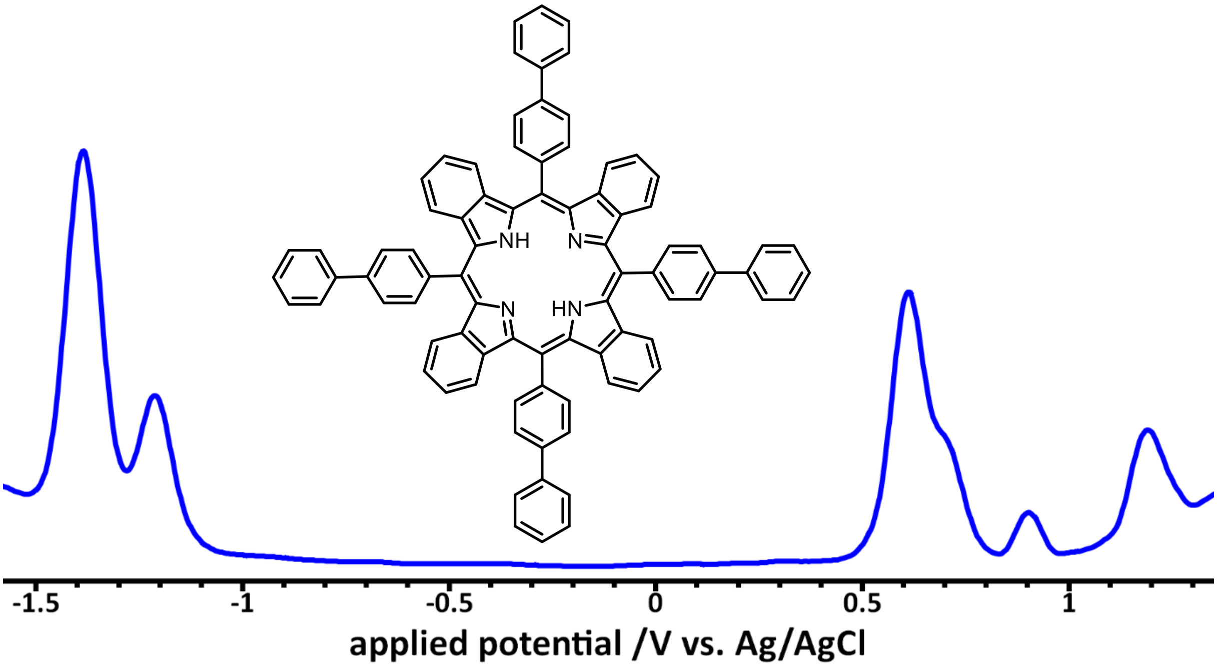

Figure S168. Differential pulse voltammogram of **29** in CH_2Cl_2 containing 0.1 M $TBAPF_6$ with a scan rate of 10 mV/s.

8 PHOTOSTABILITY MEASUREMENTS

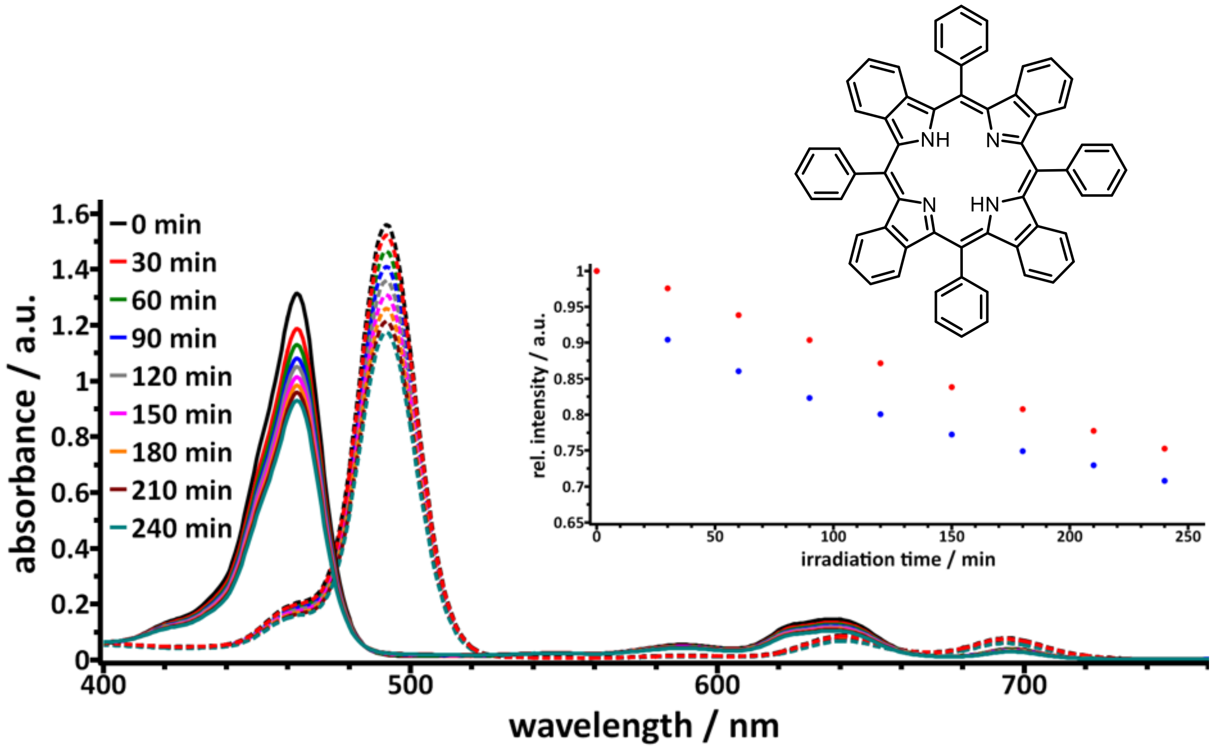

Figure S169. Photodegradation monitored by UV/vis absorption spectroscopy of **2** in CH_2Cl_2 + 1% NEt_3 (solid lines) and CH_2Cl_2 + 1% TFA (dashed lines) after different periods of irradiation time. Inset: decrease of the relative intensity of B-band versus irradiation time under basic (blue dots) and acidic conditions (red dots).

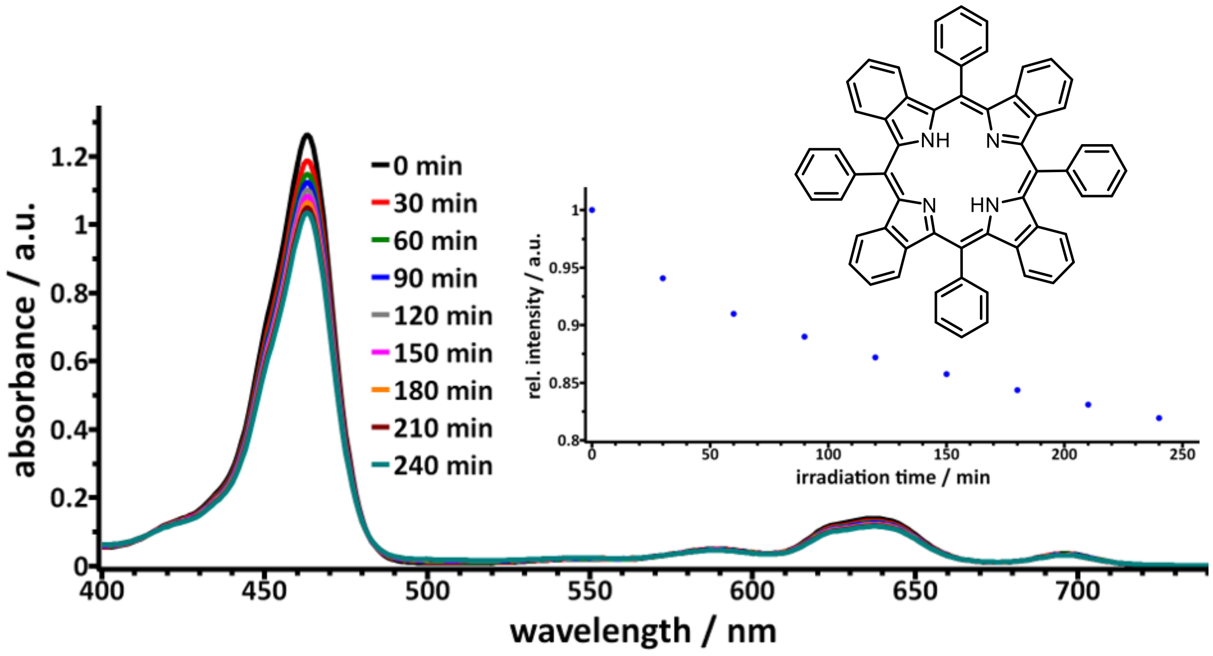

Figure S170. Photodegradation monitored by UV/vis absorption spectroscopy of **2** in CH_2Cl_2 + 1% NEt_3 (purged with Argon for 15 minutes before the measurement). Inset: decrease of the relative intensity of B-band versus irradiation time.

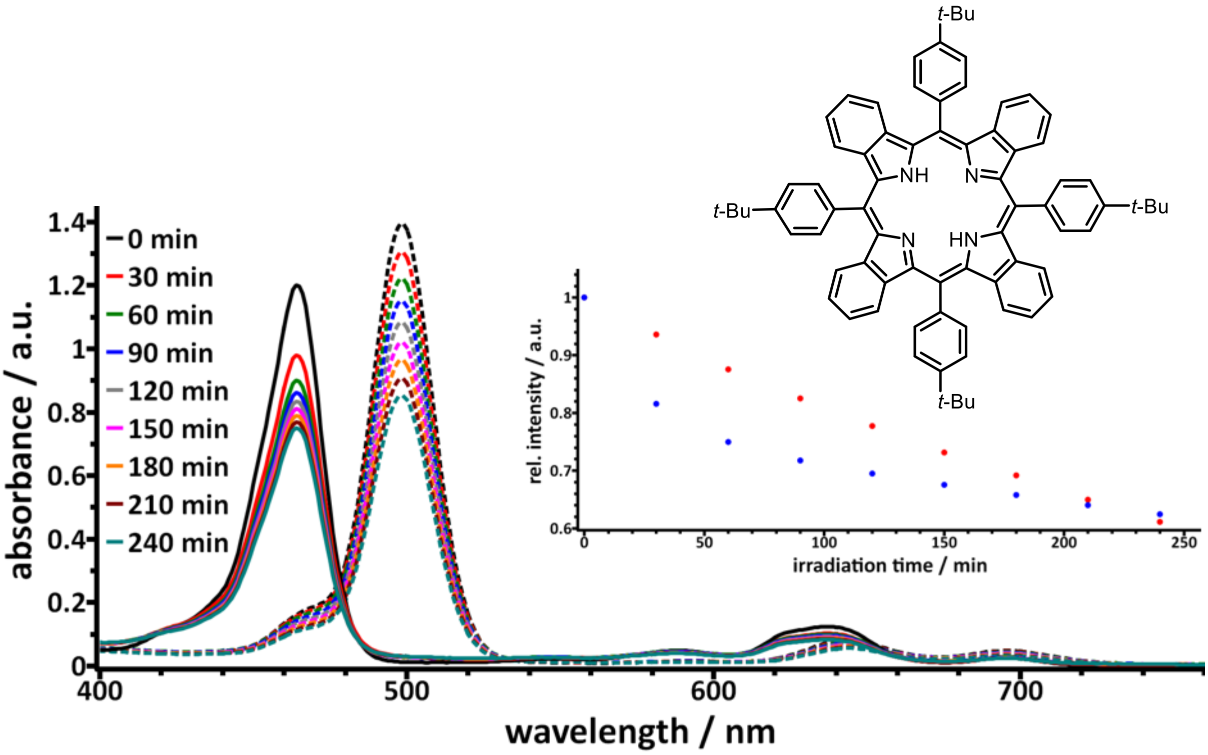

Figure S171. Photodegradation monitored by UV/vis absorption spectroscopy of **3** in CH_2Cl_2 + 1% NEt_3 (solid lines) and CH_2Cl_2 + 1% TFA (dashed lines) after different periods of irradiation time. Inset: decrease of the relative intensity of B-band versus irradiation time under basic (blue dots) and acidic conditions (red dots).

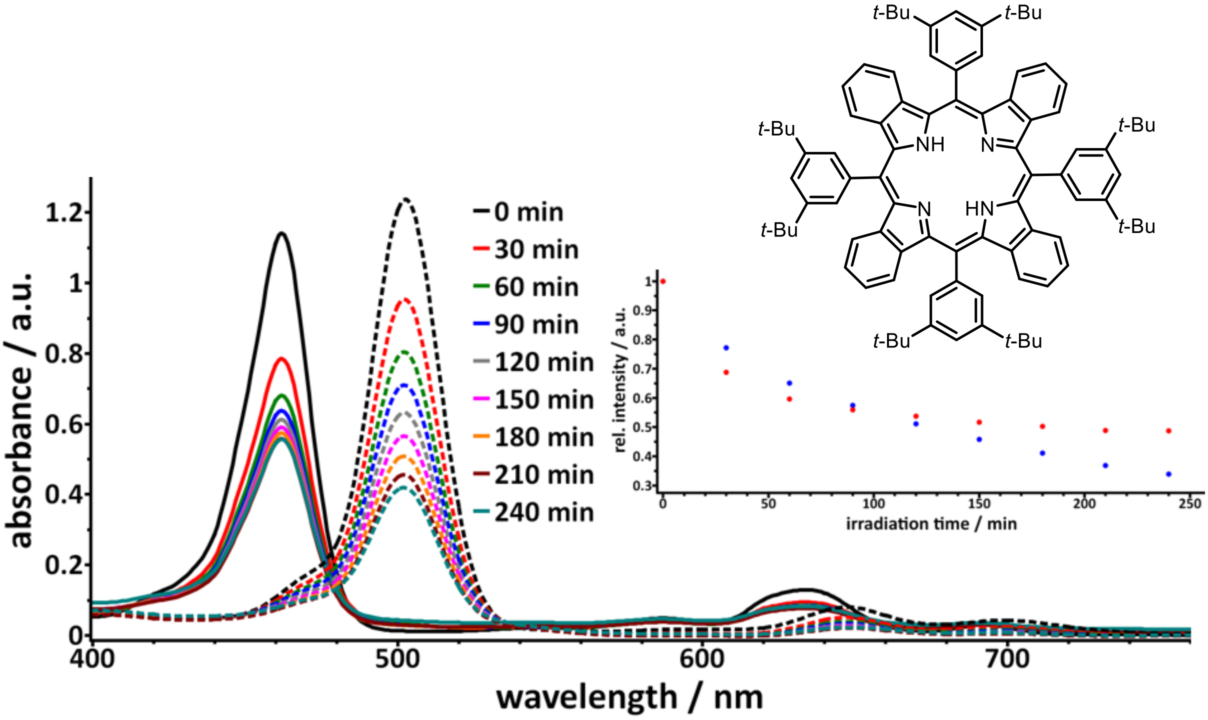

Figure S172. Photodegradation monitored by UV/vis absorption spectroscopy of **4** in CH_2Cl_2 + 1% NEt_3 (solid lines) and CH_2Cl_2 + 1% TFA (dashed lines) after different periods of irradiation time. Inset: decrease of the relative intensity of B-band versus irradiation time under basic (blue dots) and acidic conditions (red dots).

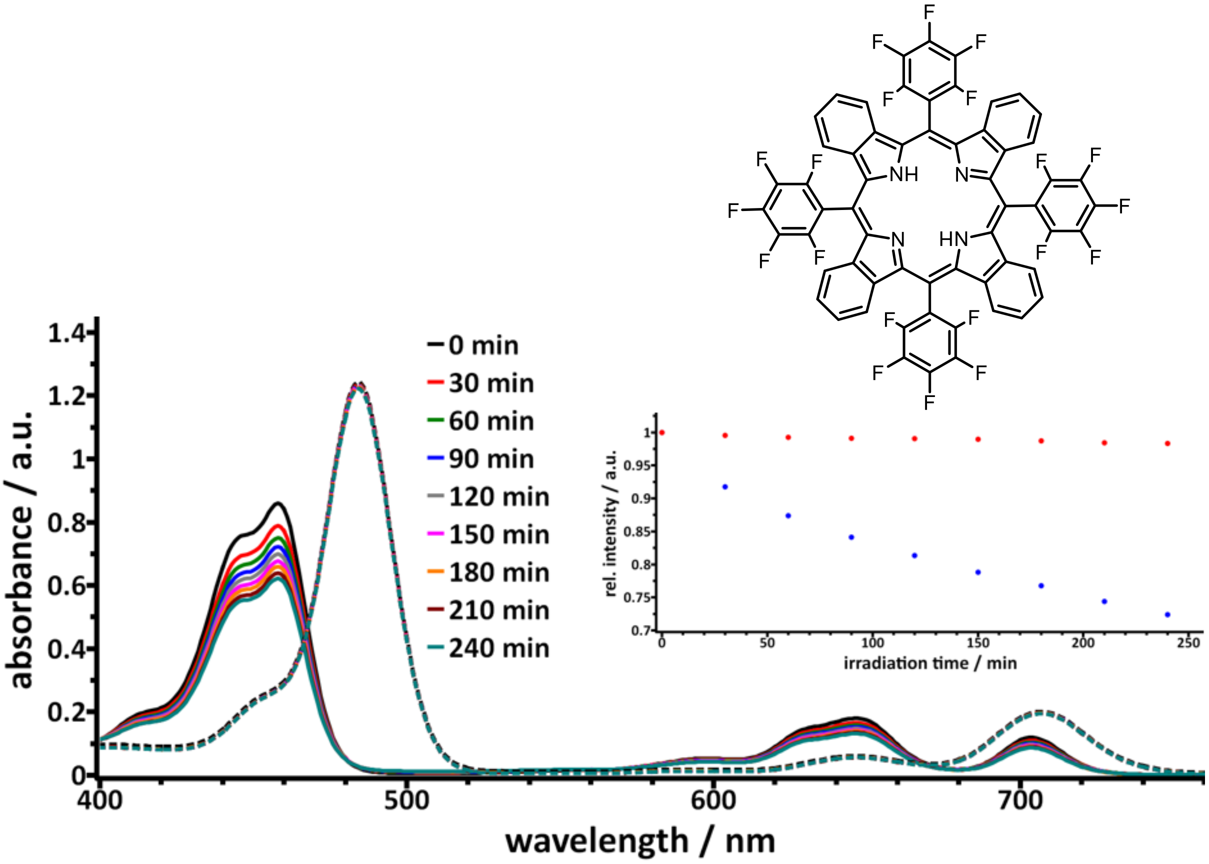

Figure S173. Photodegradation monitored by UV/vis absorption spectroscopy of **10** in CH_2Cl_2 + 1% NEt_3 (solid lines) and CH_2Cl_2 + 1% TFA (dashed lines) after different periods of irradiation time. Inset: decrease of the relative intensity of B-band versus irradiation time under basic (blue dots) and acidic conditions (red dots).

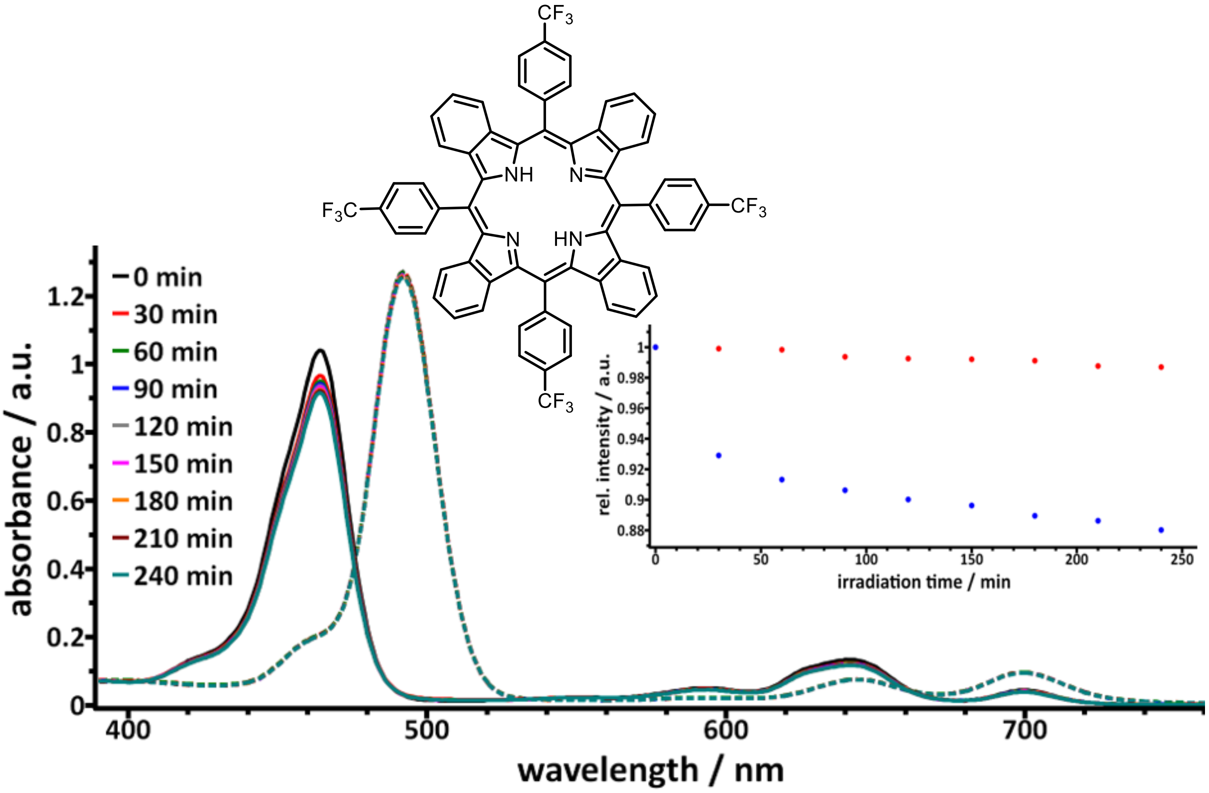

Figure S174. Photodegradation monitored by UV/vis absorption spectroscopy of **15** in CH_2Cl_2 + 1% NEt_3 (solid lines) and CH_2Cl_2 + 1% TFA (dashed lines) after different periods of irradiation time. Inset: decrease of the relative intensity of B-band versus irradiation time under basic (blue dots) and acidic conditions (red dots).

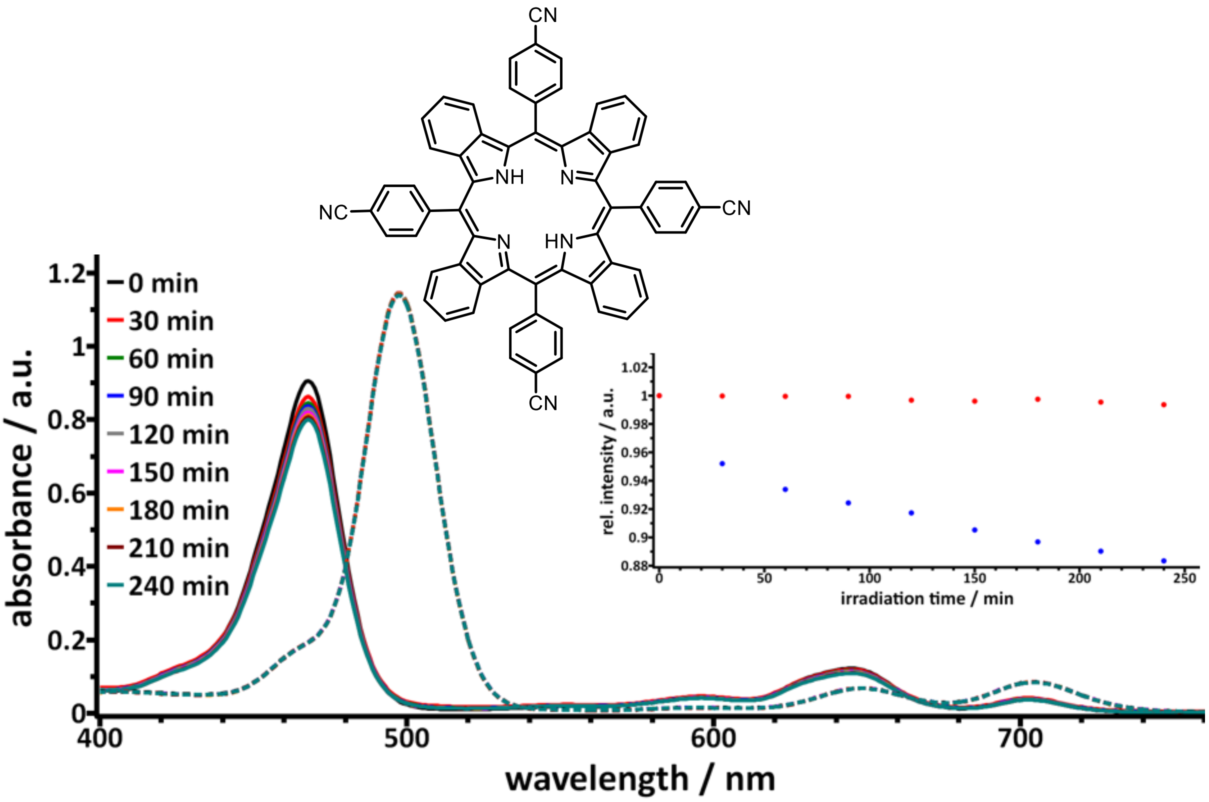

Figure S175. Photodegradation monitored by UV/vis absorption spectroscopy of **16** in CH_2Cl_2 + 1% NEt_3 (solid lines) and CH_2Cl_2 + 1% TFA (dashed lines) after different periods of irradiation time. Inset: decrease of the relative intensity of B-band versus irradiation time under basic (blue dots) and acidic conditions (red dots).

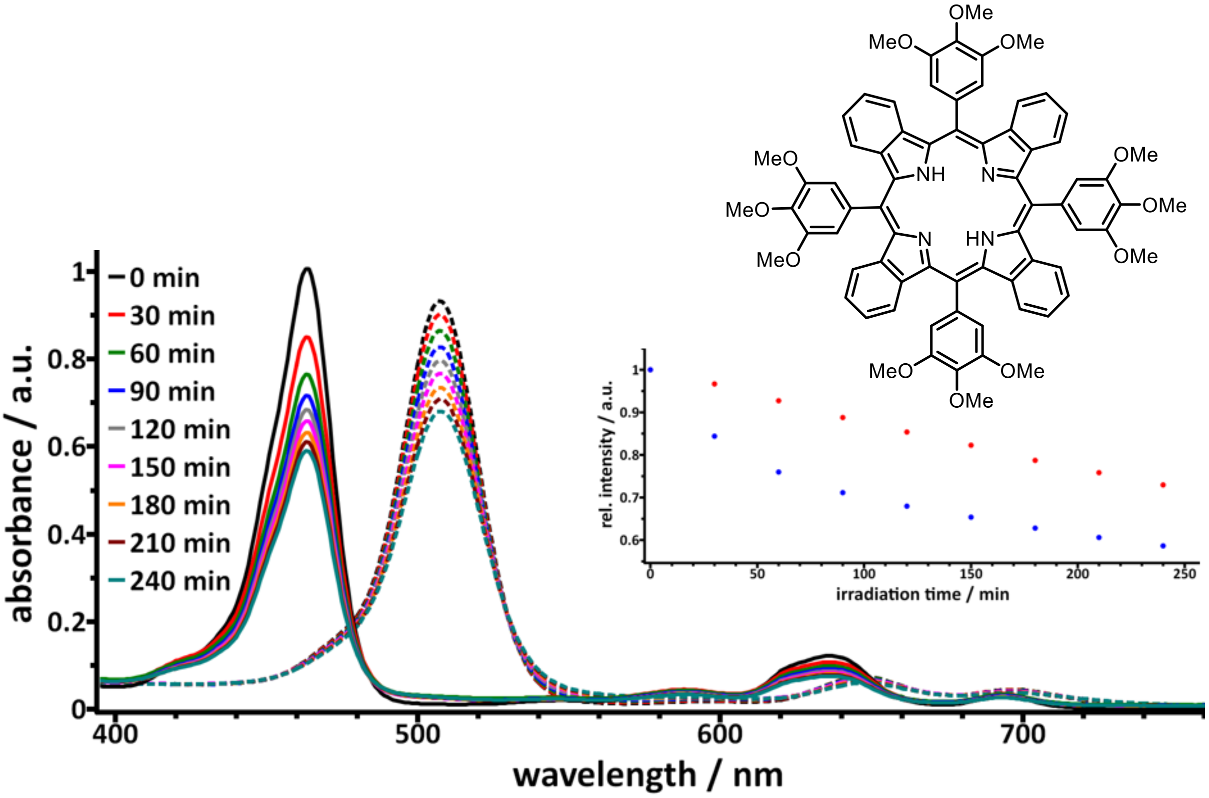

Figure S176. Photodegradation monitored by UV/vis absorption spectroscopy of **24** in CH_2Cl_2 + 1% NEt_3 (solid lines) and CH_2Cl_2 + 1% TFA (dashed lines) after different periods of irradiation time. Inset: decrease of the relative intensity of B-band versus irradiation time under basic (blue dots) and acidic conditions (red dots).

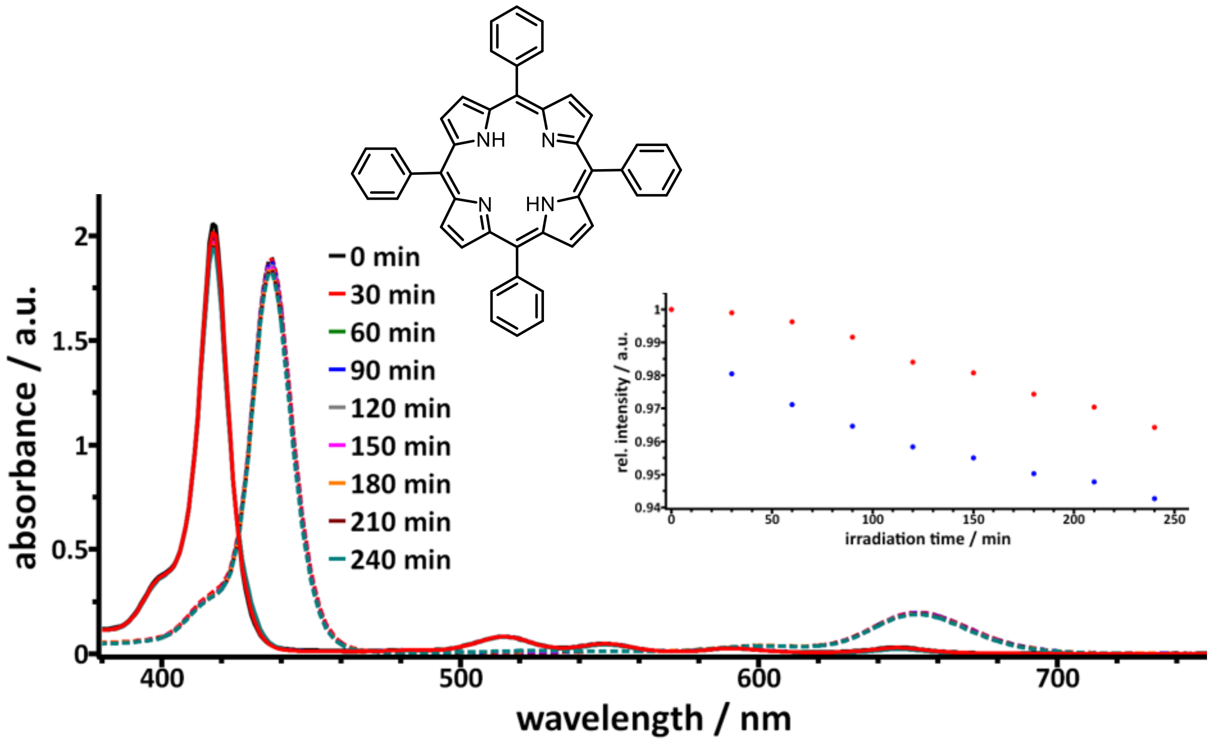

Figure S177. Photodegradation monitored by UV/vis absorption spectroscopy of **TPP** in CH_2Cl_2 + 1% NEt_3 (solid lines) and CH_2Cl_2 + 1% TFA (dashed lines) after different periods of irradiation time. Inset: decrease of the relative intensity of B-band versus irradiation time under basic (blue dots) and acidic conditions (red dots).

Table S8. Spectral data, photobleaching rate and efficiency of different TATBPs. [a]$\int \varepsilon$ is the absorbed light of TATBP in the region of cut-off filter transmittance $\int \varepsilon = \int_{620}^{800} \varepsilon_\lambda \, d\lambda$ [b]rate of photobleaching r_P after 4 h of irradiation. [c]η_P is the photobleaching efficiency $\eta_P = \frac{r_P \times S}{\varepsilon_{abs} \times V}$, where V is the reaction volume (3 cm^3) and S the irradiated area of the cell (0.32 cm^2).

Compound	$\int \varepsilon$[a] / 10^6 $M^{-1}cm^{-1}$	r_P[b]	η_P[c] / 10^{-8} M	Compound	$\int \varepsilon$[a] / 10^6 $M^{-1}cm^{-1}$	r_P[b]	η_P[c] / 10^{-8} M
**2**	1.43	0.29	2.16	**15**	1.55	0.12	0.82
**$H_2 2^{2+}$**	1.26	0.25	2.11	**$H_2 15^{2+}$**	2.08	0.01	0.05
**2** (Ar purged)	1.43	0.18	1.34	**16**	1.48	0.12	0.86
**3**	1.33	0.39	2.93	**$H_2 16^{2+}$**	1.93	0.01	0.05
**$H_2 3^{2+}$**	1.20	0.37	3.08	**24**	1.44	0.41	3.04
**4**	1.45	0.51	3.75	**$H_2 24^{2+}$**	1.59	0.27	1.81
**$H_2 4^{2+}$**	1.29	0.66	5.45	**TPP**	0.24	0.06	2.67
**10**	2.94	0.28	1.01	**H_2TPP^{2+}**	1.97	0.04	0.21
**$H_2 10^{2+}$**	3.03	0.02	0.07				

9 SINGLET OXYGEN PRODUCTION

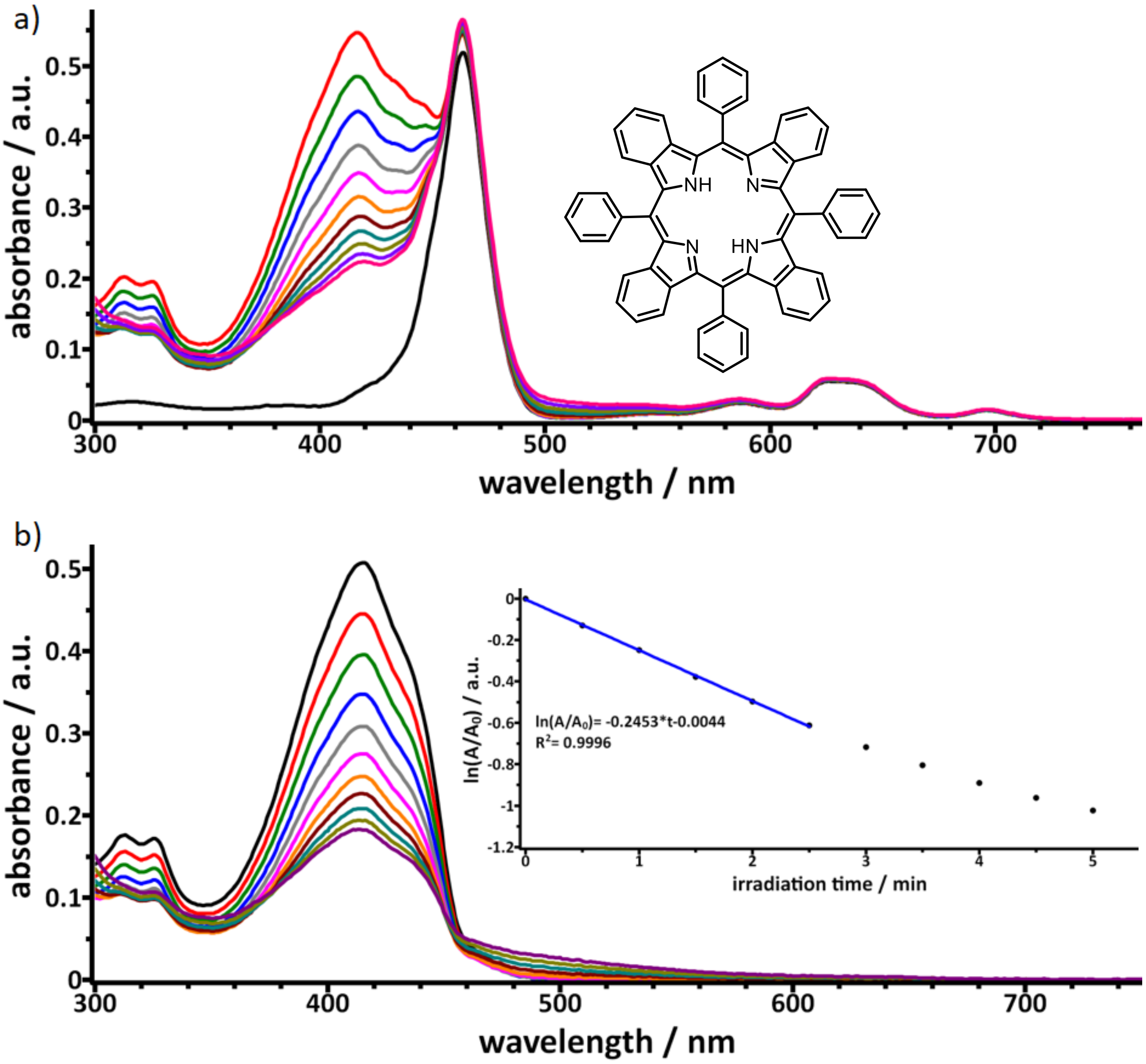

Figure S178. Singlet oxygen generation monitored by absorption spectroscopy using DPBF as singlet oxygen scavenger in DMF + 1% NEt_3. a) spectral changes upon irradiation in the presence of **2** (black line). b) decrease of DPBF absorption maximum at 415 nm after different irradiation periods. The spectrum of **2** was subtracted for clarity. inset: first-order kinetic of photooxidation of DPBF.

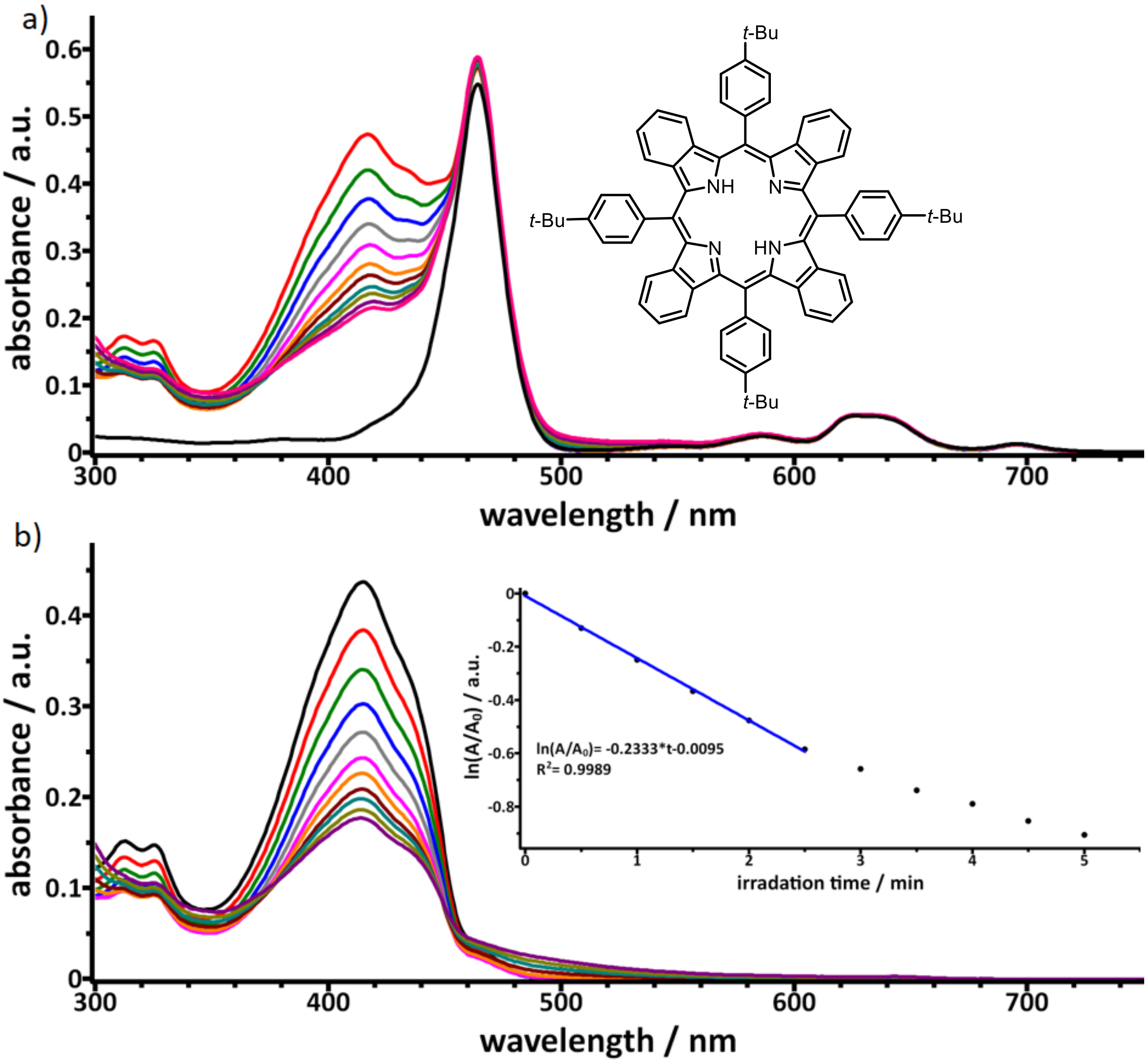

Figure S179. Singlet oxygen generation monitored by absorption spectroscopy using DPBF as singlet oxygen scavenger in DMF + 1% NEt_3. a) spectral changes upon irradiation in the presence of **3** (black line). b) decrease of DPBF absorption maximum at 415 nm after different irradiation periods. The spectrum of **3** was subtracted for clarity. inset: first-order kinetic of photooxidation of DPBF.

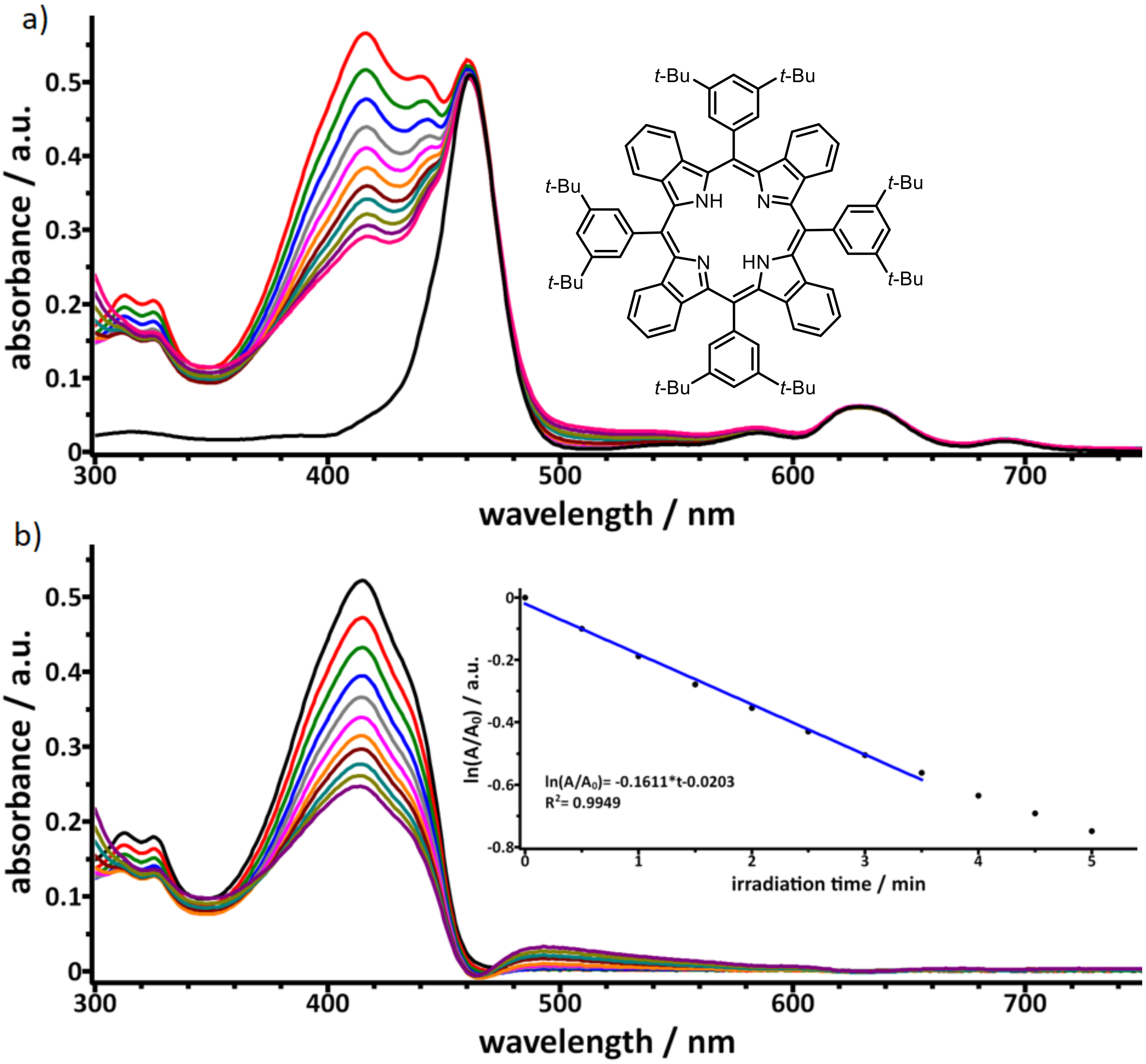

Figure S180. Singlet oxygen generation monitored by absorption spectroscopy using DPBF as singlet oxygen scavenger in DMF + 1% NEt_3. a) spectral changes upon irradiation in the presence of **4** (black line). b) decrease of DPBF absorption maximum at 415 nm after different irradiation periods. The spectrum of **4** was subtracted for clarity. inset: first-order kinetic of photooxidation of DPBF.

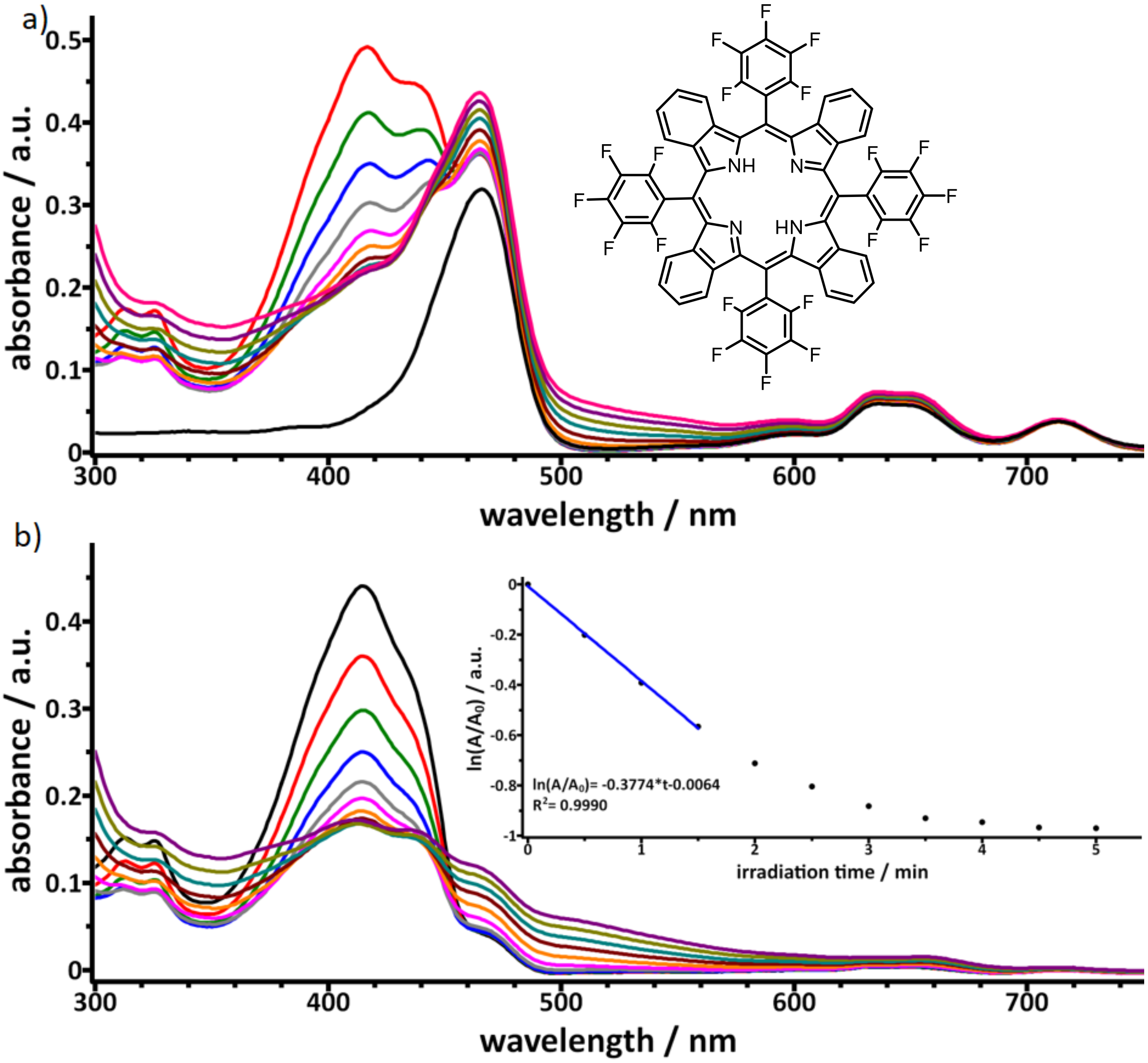

Figure S181. Singlet oxygen generation monitored by absorption spectroscopy using DPBF as singlet oxygen scavenger in DMF + 1% NEt_3. a) spectral changes upon irradiation in the presence of **10** (black line). b) decrease of DPBF absorption maximum at 415 nm after different irradiation periods. The spectrum of **10** was subtracted for clarity. inset: first-order kinetic of photooxidation of DPBF.

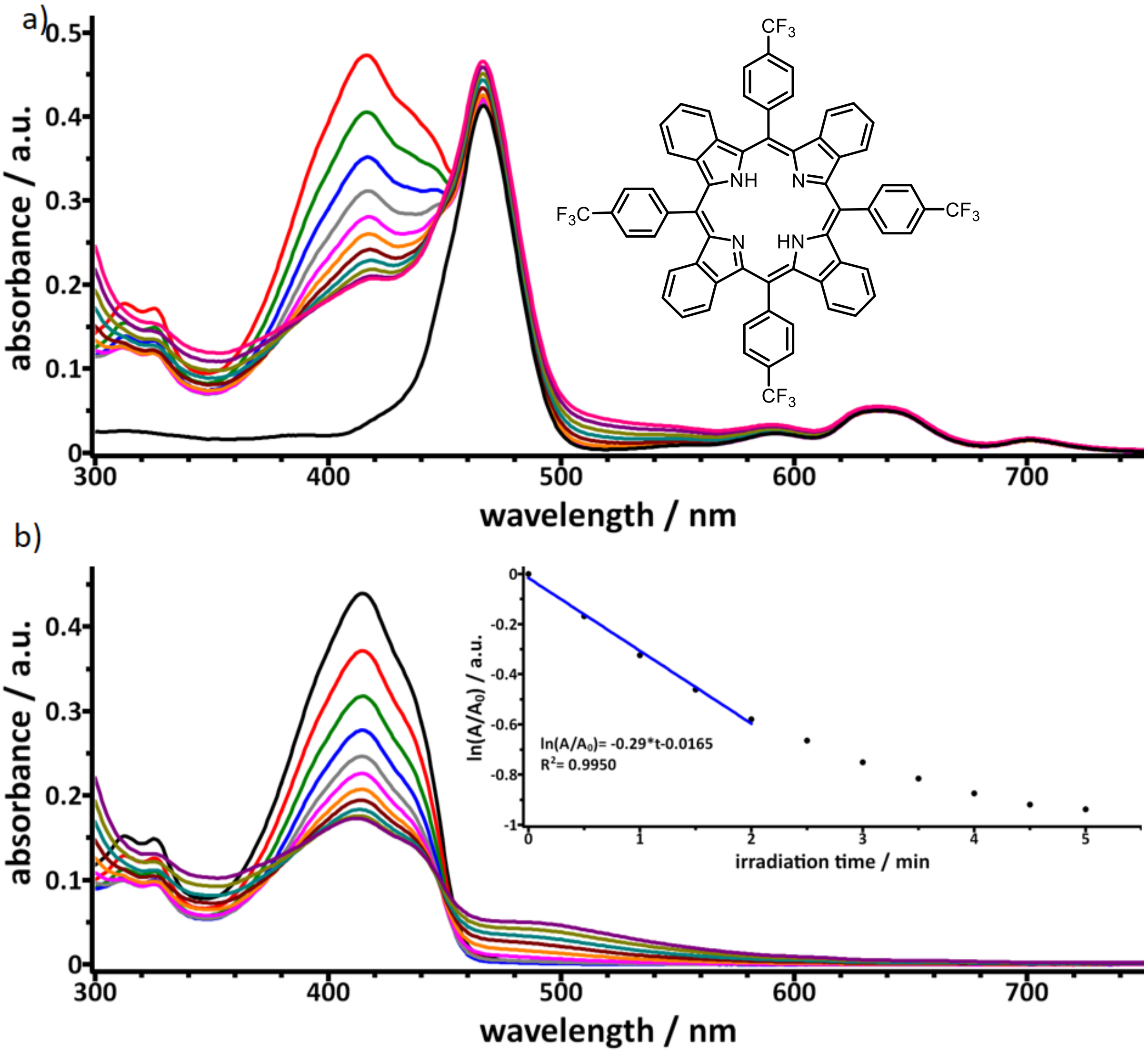

Figure S182. Singlet oxygen generation monitored by absorption spectroscopy using DPBF as singlet oxygen scavenger in DMF + 1% NEt_3. a) spectral changes upon irradiation in the presence of **15** (black line). b) decrease of DPBF absorption maximum at 415 nm after different irradiation periods. The spectrum of **15** was subtracted for clarity. inset: first-order kinetic of photooxidation of DPBF.

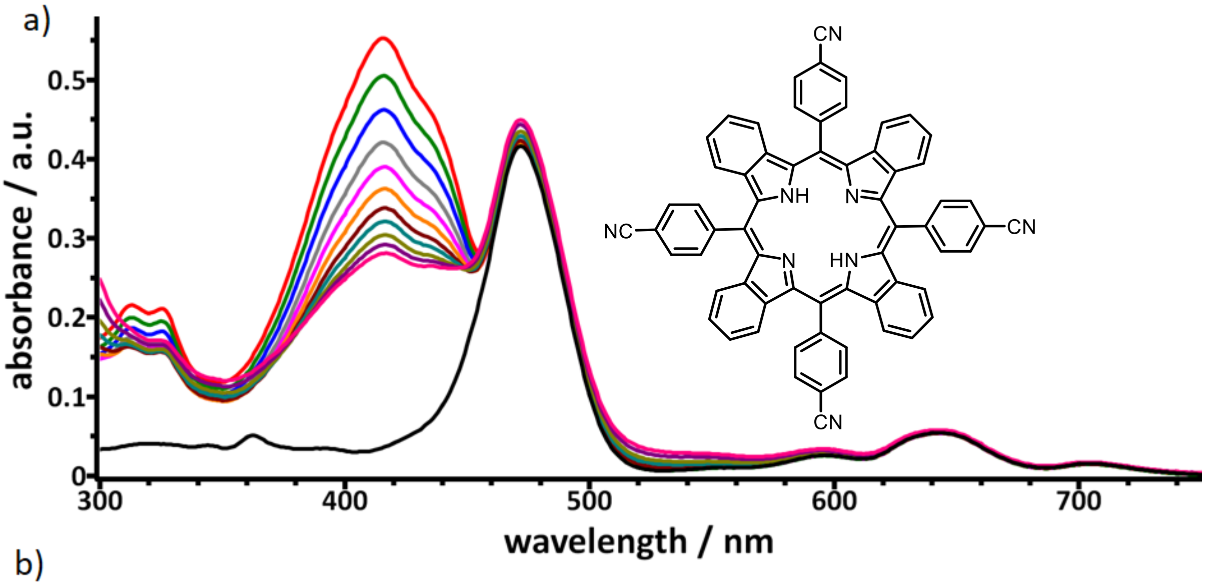

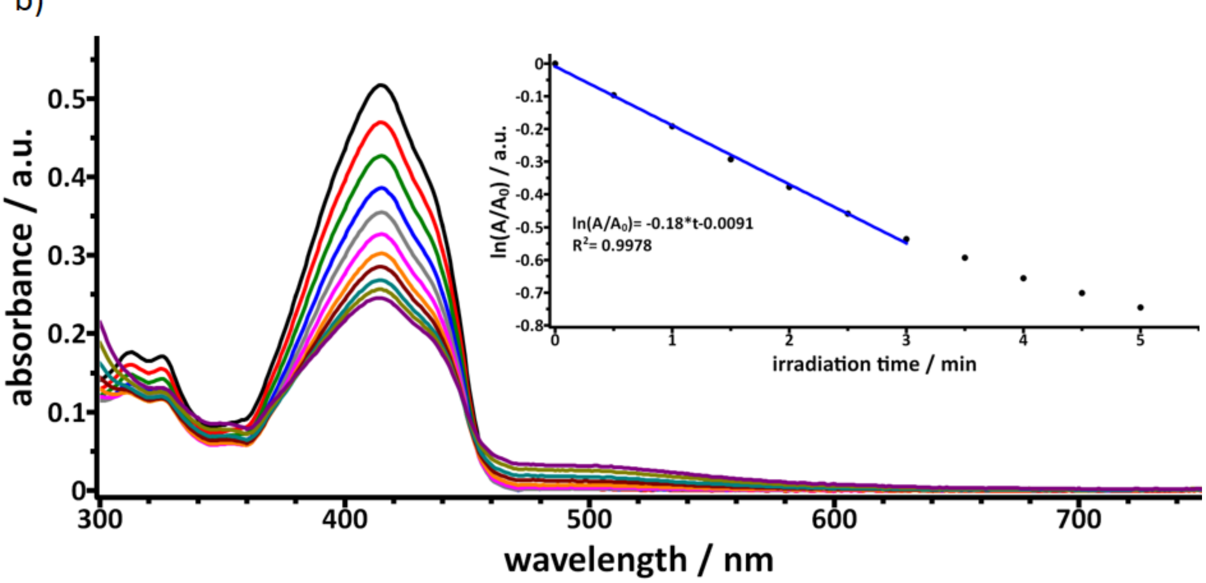

Figure S183. Singlet oxygen generation monitored by absorption spectroscopy using DPBF as singlet oxygen scavenger in DMF + 1% NEt_3. a) spectral changes upon irradiation in the presence of **16** (black line). b) decrease of DPBF absorption maximum at 415 nm after different irradiation periods. The spectrum of **16** was subtracted for clarity. inset: first-order kinetic of photooxidation of DPBF.

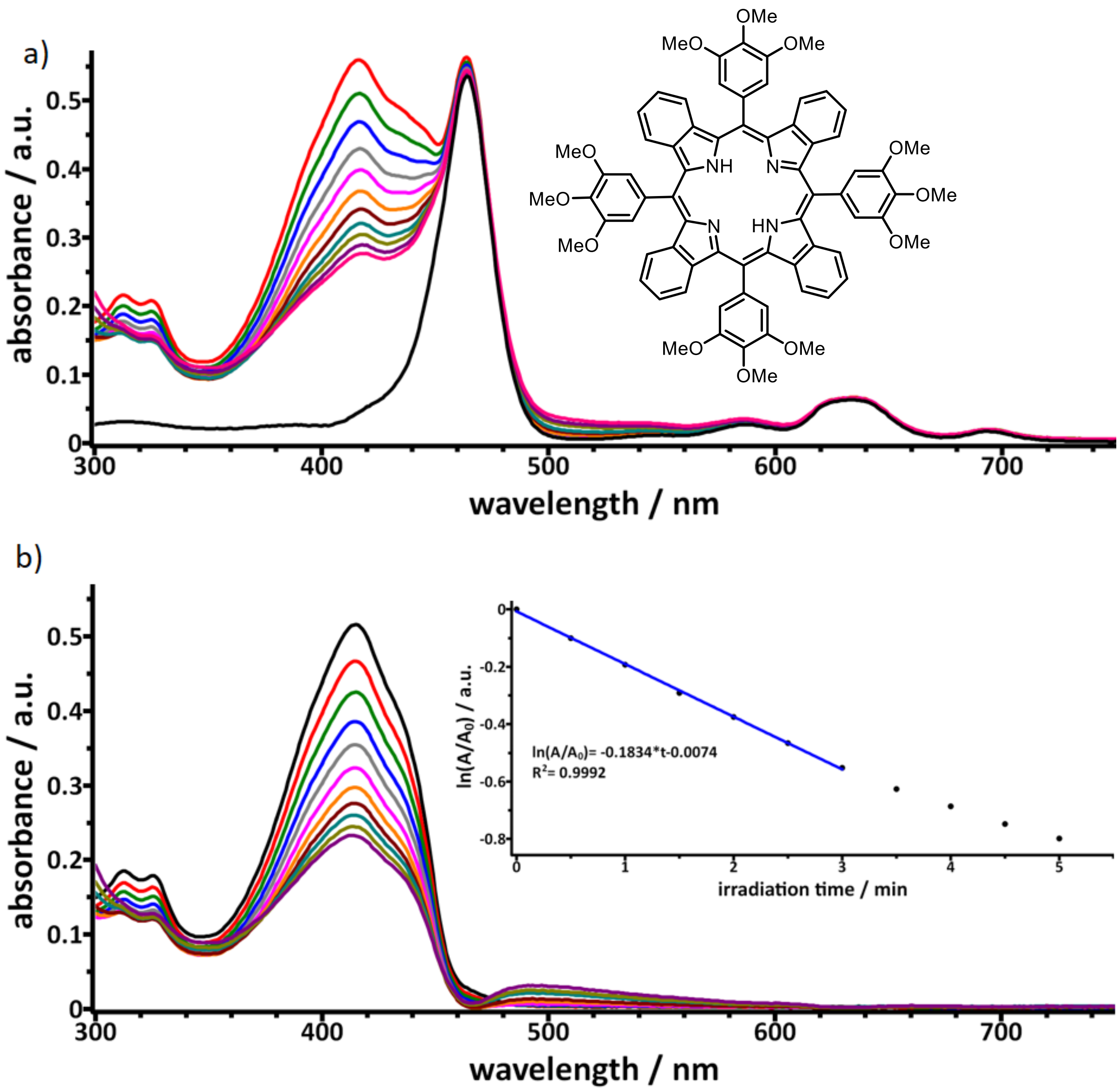

Figure S184. Singlet oxygen generation monitored by absorption spectroscopy using DPBF as singlet oxygen scavenger in DMF + 1% NEt_3. a) spectral changes upon irradiation in the presence of **24** (black line). b) decrease of DPBF absorption maximum at 415 nm after different irradiation periods. The spectrum of **24** was subtracted for clarity. inset: first-order kinetic of photooxidation of DPBF.

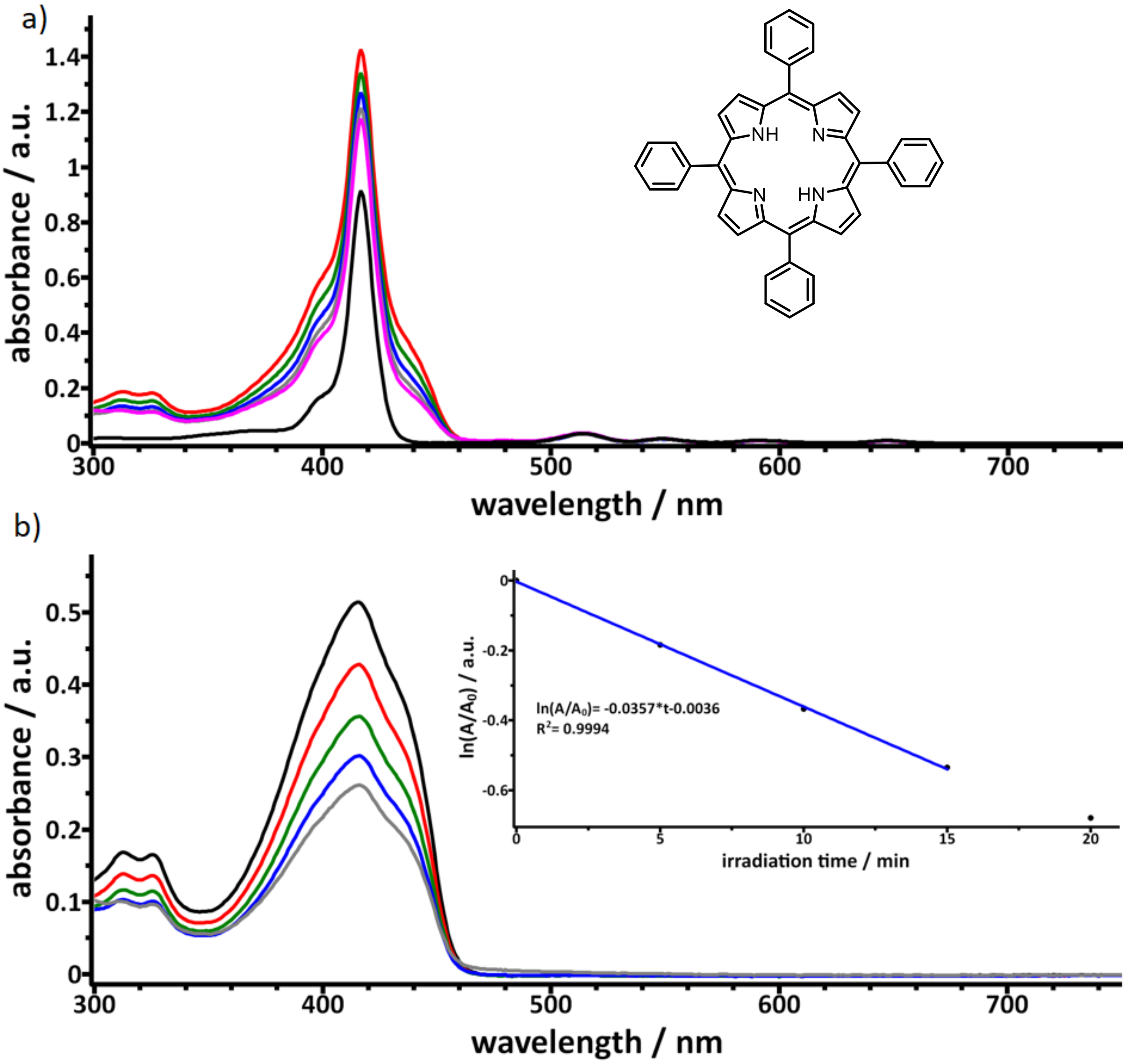

Figure S185. Singlet oxygen generation monitored by absorption spectroscopy using DPBF as singlet oxygen scavenger in DMF + 1% NEt_3. a) spectral changes upon irradiation in the presence of TPP (black line). b) decrease of DPBF absorption maximum at 415 nm after different irradiation periods. The spectrum of TPP was subtracted for clarity. inset: first-order kinetic of photooxidation of DPBF.

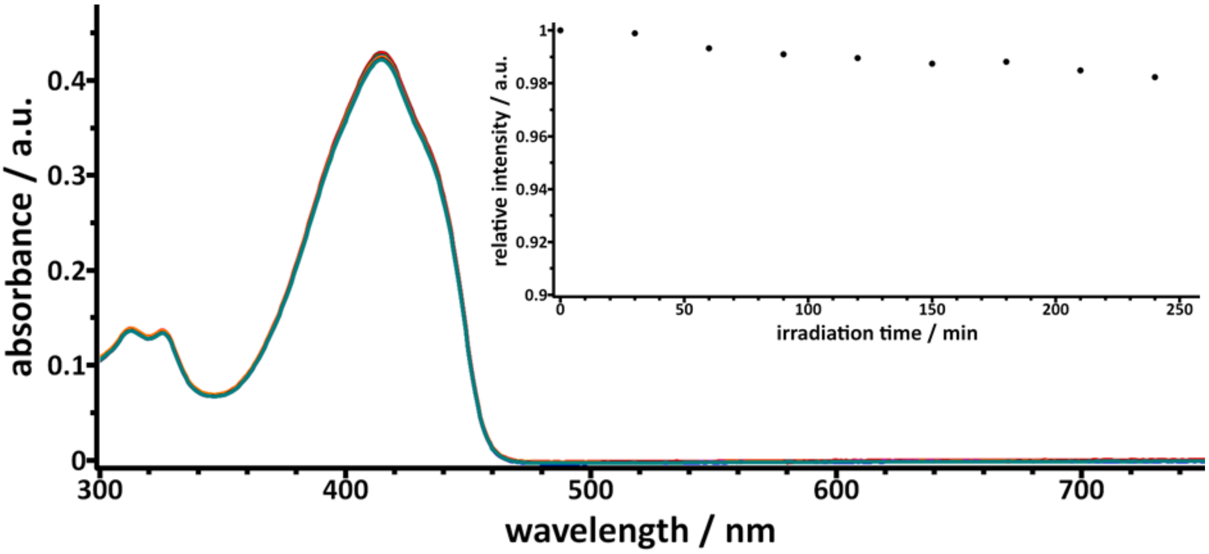

Figure S186. Photodegradation monitored by UV/vis absorption spectroscopy of DPBF in DMF + 1% NEt_3 after different periods of irradiation time. Inset: decrease of the relative intensity of absorption maximum at 415 nm versus irradiation time.

Table S9. Spectral data, photobleaching rate and efficiency of TATBPs. [a]$\int\varepsilon$ is the absorbed light of TATBP in the region of cut-off filter transmittance $\int \varepsilon = \int_{620}^{800} \varepsilon_\lambda \, d\lambda$. [b]$k_b$ is defined as the slope of the plot ln(A/A_0) of the DPBF absorption maximum vs irradiation time. [c]$\Delta_{rel}(^1O_2)$ is the relative singlet oxygen quantum yield and is defined as $\Delta_{rel}(^1O_2) = \Delta^{TPP} \frac{k_q^{TATBP} \times \int \varepsilon^{TPP}}{k_q^{TPP} \times \int \varepsilon^{TATBP}}$. Δ^{TPP} is defined as 1.

compound	$\int\varepsilon$[a] / 10^6 $M^{-1}cm^{-1}$	k_b[b] / min^{-1}	$\Delta_{rel}(^1O_2)$[c] / a.u.
**2**	1.24	-0.24	0.63
**3**	1.14	-0.23	0.65
**4**	1.27	-0.17	0.43
**10**	2.27	-0.38	0.54
**15**	1.27	-0.29	0.74
**16**	1.57	-0.18	0.37
**24**	1.43	-0.183	0.41
**TPP**	0.11	-0.034	1

10 COMPUTATIONAL DATA

Geometry optimized structures were obtained at the density functional theory B3LYP 6-311G** level of theory, using the SPARTAN ’16 (Win/64b) Release 2.0.0 work package.[13]

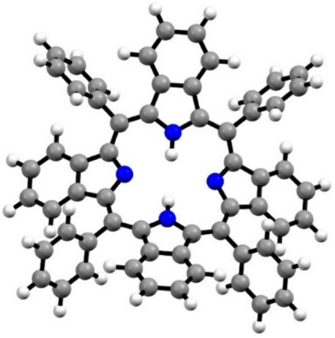

meso-Tetraphenyltetrabenzoporphyrin 2

cartesian coordinates (Angstroms)

Atom	X	Y	Z	Atom	X	Y	Z
N1	0.149556	-2.106269	0	H52	-1.532895	-2.488635	-5.188052
C2	0.366798	-2.830304	-1.148067	C53	-1.50706	1.409074	-5.174289
C3	0.913777	-4.103919	-0.709786	H54	-1.534265	2.487624	-5.188042
C4	0.913777	-4.103919	0.709787	C55	-2.062774	-0.700004	-6.23093
C5	0.366797	-2.830304	1.148068	H56	-2.509535	-1.239235	-7.05905
H6	-0.162762	-1.14313	0	C57	-2.063174	0.698714	-6.23091
C7	0.007513	-2.418093	2.439307	H58	-2.510241	1.237709	-7.059019
C8	0.007514	-2.418093	-2.439307	C59	0.013839	3.470576	-3.504336
C9	-0.355198	-1.102847	2.812011	C60	0.025529	5.445213	-5.497998
C10	-0.355197	-1.102847	-2.812011	C61	-0.959239	4.476761	-3.517822
N11	-0.110005	-0.000149	2.051881	C62	0.992761	3.4679	-4.504316
C12	-0.355602	1.102434	2.812042	C63	1.002587	4.451226	-5.490639
C13	-0.938279	-0.705697	4.105193	C64	-0.957914	5.453552	-4.510174
C14	-0.938627	0.705014	4.105188	H65	1.771203	4.438941	-6.255679
N15	-0.110003	-0.000148	-2.05188	H66	-1.723065	6.222179	-4.510879
C16	-0.355601	1.102435	-2.812042	H67	0.030007	6.207892	-6.268894
C17	-0.938276	-0.705696	-4.105193	C68	0.01384	3.470575	3.504336
C18	-0.938625	0.705015	-4.105188	C69	0.025533	5.445214	5.497996
C19	0.006658	2.417819	-2.439312	C70	0.992758	3.467896	4.504319
C20	0.365787	2.830102	-1.148064	C71	-0.959233	4.476765	3.517818
C21	0.006657	2.417818	2.439312	C72	-0.957907	5.453557	4.510168
N22	0.148759	2.105992	0	C73	1.002586	4.451222	5.490641
H23	-0.163245	1.14275	0	H74	-1.723054	6.222188	4.510871
C24	0.365787	2.830101	1.148064	H75	1.771199	4.438934	6.255683
C25	0.912411	4.103868	-0.709784	H76	0.030012	6.207894	6.268891
C26	0.91241	4.103868	0.709785	C77	0.014912	-3.470783	3.5044
C27	1.456957	5.19313	-1.412696	C78	0.026877	-5.445194	5.498286
H28	1.4869	5.203135	-2.49092	C79	0.993672	-3.467701	4.504539
C29	1.456954	5.19313	1.412698	C80	-0.957917	-4.477206	3.517895
H30	1.486896	5.203135	2.490922	C81	-0.956445	-5.453895	4.510344
C31	1.980207	6.258171	-0.702277	C82	1.003645	-4.450921	5.490967
H32	2.407137	7.098028	-1.238915	H83	-1.721388	-6.222728	4.511037
C33	1.980205	6.25817	0.702281	H84	1.772149	-4.438333	6.256115
H34	2.407135	7.098027	1.23892	H85	0.031469	-6.207793	6.26926
C35	-1.50628	-1.410066	5.174313	C86	0.014912	-3.470783	-3.5044
H36	-1.532899	-2.488635	5.188051	C87	0.026873	-5.445193	-5.498287
C37	-1.507061	1.409074	5.174289	C88	-0.957919	-4.477205	-3.517894
H38	-1.534265	2.487624	5.188043	C89	0.993671	-3.467702	-4.50454
C39	-2.062777	-0.700005	6.230929	C90	1.003642	-4.450922	-5.490968
H40	-2.509537	-1.239236	7.059049	C91	-0.956448	-5.453894	-4.510344
C41	-2.063175	0.698714	6.23091	H92	1.772146	-4.438335	-6.256117
H42	-2.510242	1.237709	7.059019	H93	-1.721392	-6.222726	-4.511036
C43	1.458589	-5.193054	1.412694	H94	0.031463	-6.207793	-6.269261
H44	1.48851	-5.203069	2.490916	H95	-1.722294	-4.487274	2.748971
C45	1.45859	-5.193054	-1.412693	H96	-1.722295	-4.487273	-2.74897
H46	1.488511	-5.203069	-2.490915	H97	1.75119	-2.692123	-4.50309
C47	1.982115	-6.257957	-0.702279	H98	-1.723701	4.486556	-2.748978
H48	2.409246	-7.097711	-1.238917	H99	1.75119	-2.692122	4.503089
C49	1.982114	-6.257957	0.70228	H100	-1.723693	4.486564	2.748971
H50	2.409245	-7.09771	1.238919	H101	1.750506	2.692541	4.502839
C51	-1.506277	-1.410066	-5.174313	H102	1.750512	2.692549	-4.502834

meso-tetraphenyltetrabenzoporphyrin dication

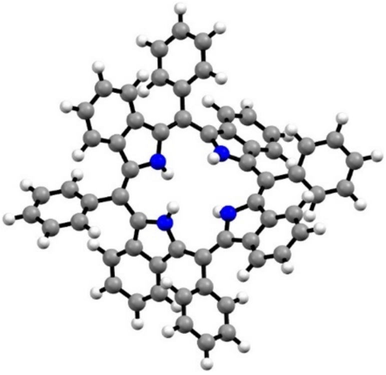

cartesian coordinates (Angstroms)

Atom	X	Y	Z	Atom	X	Y	Z
N1	1.731138	-1.093439	0.747672	C53	1.60646	1.786323	-4.947103
C2	2.900404	-0.371176	0.628893	H54	0.735038	2.271309	-5.363635
C3	3.465787	-0.346525	1.922608	C55	3.774046	0.742454	-5.253844
C4	2.606896	-1.109921	2.791822	H56	4.574692	0.437761	-5.91873
C5	1.526159	-1.586763	2.018829	C57	2.653537	1.403093	-5.774493
H6	1.162775	-1.357073	-0.043774	H58	2.594975	1.604175	-6.83847
C7	0.446678	-2.50991	2.347516	C59	-0.80206	3.383604	-3.515553
C8	3.411601	0.09786	-0.652479	C60	-1.480578	5.25453	-5.504605
C9	-0.818397	-2.3703	1.838451	C61	-2.017292	3.288545	-4.208924
C10	2.590063	0.600618	-1.62826	C62	0.058137	4.447098	-3.82707
N11	-1.270901	-1.209278	1.173306	C63	-0.275812	5.371144	-4.813471
C12	-2.674202	-1.219677	1.015975	C64	-2.350409	4.209116	-5.197344
C13	-1.960288	-3.2864	1.827725	H65	0.40282	6.187308	-5.037342
C14	-3.085453	-2.588021	1.33318	H66	-3.290837	4.11174	-5.727771
N15	1.246817	0.974701	-1.402916	H67	-1.742867	5.976054	-6.270307
C16	0.725587	1.7049	-2.49314	C68	-4.888758	-0.105093	0.77837
C17	2.824352	0.853741	-3.050351	C69	-7.685692	-0.018299	1.072888
C18	1.694135	1.521241	-3.575152	C70	-5.489966	-0.352592	2.021225
C19	-0.454936	2.400128	-2.450599	C71	-5.717965	0.205042	-0.309568
C20	-1.426049	2.236156	-1.377349	C72	-7.101313	0.242085	-0.165996
C21	-3.406422	-0.126966	0.62727	C73	-6.873709	-0.313011	2.166732
N22	-1.726799	1.012049	-0.816678	H74	-7.724347	0.476509	-1.022186
H23	-1.31629	0.145242	-1.13132	H75	-7.31774	-0.504574	3.137323
C24	-2.793814	1.064109	0.056666	H76	-8.763435	0.016508	1.186178
C25	-2.319809	3.156301	-0.785853	C77	0.786086	-3.617982	3.283712
C26	-3.174075	2.423356	0.113414	C78	1.457888	-5.69818	5.057107
C27	-2.464301	4.561318	-0.90831	C79	0.047518	-3.831634	4.457381
H28	-1.826371	5.121914	-1.579703	C80	1.883286	-4.454551	3.030977
C29	-4.152004	3.112613	0.874133	C81	2.211774	-5.487018	3.903477
H30	-4.79612	2.574585	1.558203	C82	0.377002	-4.862885	5.332656
C31	-3.42535	5.196361	-0.16194	H83	3.059169	-6.127529	3.683429
H32	-3.54777	6.270521	-0.24612	H84	-0.205924	-5.007895	6.23544
C33	-4.267039	4.472646	0.729241	H85	1.717524	-6.4994	5.739971
H34	-5.011413	5.011906	1.304934	C86	4.884887	0.010108	-0.853251
C35	-2.048093	-4.654328	2.109666	C87	7.673968	-0.153449	-1.19103
H36	-1.187341	-5.202088	2.465748	C88	5.568601	-1.193935	-0.626431
C37	-4.295244	-3.260019	1.122596	C89	5.632108	1.13518	-1.23252
H38	-5.153755	-2.74015	0.721914	C90	7.011239	1.054146	-1.402879
C39	-3.259212	-5.304591	1.915618	C91	6.946418	-1.276817	-0.79906
H40	-3.337365	-6.363937	2.132473	H92	7.569726	1.937605	-1.692337
C41	-4.374492	-4.6124	1.425325	H93	7.454273	-2.219125	-0.625128
H42	-5.307149	-5.141723	1.265933	H94	8.748577	-0.216896	-1.320363
C43	2.922242	-1.229698	4.16871	H95	2.478855	-4.293172	2.139715
H44	2.282861	-1.801794	4.829322	H96	5.009478	-2.068399	-0.313948
C45	4.618766	0.287058	2.450736	H97	5.122233	2.078901	-1.38874
H46	5.266682	0.87054	1.808927	H98	-2.700156	2.481209	-3.971756
C47	4.895937	0.149238	3.787888	H99	-0.788264	-3.178631	4.682559
H48	5.774345	0.628688	4.205842	H100	-5.269121	0.414358	-1.273615
C49	4.049701	-0.60917	4.645791	H101	-4.861157	-0.571817	2.8764
H50	4.304212	-0.691964	5.696762	H102	0.993882	4.545279	-3.288023
C51	3.864108	0.455135	-3.898516	H103	0.992433	1.273102	-0.47018
H52	4.721364	-0.077674	-3.511881	H104	-0.83138	-0.329343	1.411382

11 REFERENCES

[1] G. M. Sehldrick, *Acta Cryst. A* **2008**, *64*, 112–122.

[2] O. V. Dolomanov, L. J. Bourhis, R. J. Gildea, J. A. K. Howard, H. Puschmann, *J. Appl. Cryst.* **2009**, *42*, 339-341.

[3] G.M. Sheldrick, *Acta Cryst. A* **2015**, *64*, 112-122.

[4] G.M. Sheldrick, *Acta Cryst. A* **2015**, *71*, 3-8.

[5] G.M. Sheldrick, *Acta Cryst. C* **2015**, *71*, 3-8.

[6] S. D. Jeong, B. Min, S. Y. Cho, C. Lee, B. K. Park, K. S. An, J. Lim, *J. Org. Chem.* **2012**, *77*, 8329-8331.

[7] *NSDGUI*, Version 1.3 alpha version; Sandia National Laboratory: New Mexico, **2001**.

[8] W. Jentzen, X.-Z. Song, J. A. Shelnutt, *J. Phys. Chem. B* **1997**, *101*, 1684–1699.

[9] W. Jentzen, J.-G. Ma, J. A. Shelnutt, *Biophys. J.* **1998**, *74*, 753–763.

[10] J. A. Shelnutt, *J. Porphyrins Phthalocyanines* **2001**, *5*, 300–311.

[11] J. Schindler, S. Kupfer, A. A. Ryan, K. J. Flanagan, M. O. Senge, B. Dietzek, *Coord. Chem. Rev.* **2018**, *360*, 1–16.

[12] M. O. Senge, S. A. MacGowan, J. M. O'Brien, *Chem. Commun.* **2015**, *51*, 17031–17063.

[13] Y. Shao, L.F. Molnar, Y. Jung, J. Kussmann, C. Ochsenfeld, S.T. Brown, A.T.B. Gilbert, L.V. Slipchenko, S.V. Levchenko, D.P. O'Neill, R.A. DiStasio Jr., R.C. Lochan, T. Wang, G.J.O. Beran, N.A. Besley, J.M. Herbert, C.Y. Lin, T. Van Voorhis, S.H. Chien, A.Sodt, R.P. Steele, V.A. Rassolov, P.E. Maslen, P.P. Korambath, R.D. Adamson, B. Austin, J. Baker, E.F.C. Byrd, H. Dachsel, R.J. Doerksen, A. Dreuw, B.D. Dunietz, A.D. Dutoi, T.R. Furlani, S.R. Gwaltney, A. Heyden, S. Hirata, C-P. Hsu, G. Kedziora, R.Z. Khalliulin, P. Klunzinger, A.M. Lee, M.S. Lee, W.Z. Liang, I. Lotan, N. Nair, B. Peters, E.I. Proynov, P.A. Pieniazek, Y.M. Rhee, J. Ritchie, E. Rosta, C.D. Sherrill, A.C. Simmonett, J.E. Subotnik, H.L. Woodcock III, W. Zhang, A.T. Bell, A.K. Chakraborty, D.M. Chipman, F.J. Keil, A.Warshel, W.J. Hehre, H.F. Schaefer, J. Kong, A.I. Krylov, P.M.W. Gill and M. Head-Gordon, *Phys. Chem. Chem. Phys.* **2006**, *8*, 3172–3191.
